# *Velvet*-mediated repression of β-glucan synthesis in *Aspergillus nidulans* spores

**DOI:** 10.1038/srep10199

**Published:** 2015-05-11

**Authors:** Hee-Soo Park, Yeong Man Yu, Mi-Kyung Lee, Pil Jae Maeng, Sun Chang Kim, Jae-Hyuk Yu

**Affiliations:** 1 Department of Bacteriology, University of Wisconsin, Madison, WI, USA; 2 Department of Microbiology and Molecular Biology, Chungnam National University, Daejeon, Republic of Korea; 3 Department of Biological Sciences, Korea Advanced Institute of Science and Technology, Dae-Jon, Republic of Korea

## Abstract

Beta-glucans are a heterologous group of fibrous glucose polymers that are a major constituent of cell walls in Ascomycetes and Basidiomycetes fungi. Synthesis of β (1,3)- and (1,6)-glucans is coordinated with fungal cell growth and development, thus, is under tight genetic regulation. Here, we report that β-glucan synthesis in both asexual and sexual spores is turned off by the NF-kB like fungal regulators VosA and VelB in *Aspergillus nidulans*. Our genetic and genomic analyses have revealed that both VosA and VelB are necessary for proper down-regulation of cell wall biosynthetic genes including those associated with β-glucan synthesis in both types of spores. The deletion of *vosA* or *velB* results in elevated accumulation of β-glucan in asexual spores. Double mutant analyses indicate that VosA and VelB play an inter-dependent role in repressing β-glucan synthesis in asexual spores. *In vivo* chromatin immuno-precipitation analysis shows that both VelB and VosA bind to the promoter region of the β-glucan synthase gene *fksA* in asexual spores. Similarly, VosA is required for proper repression of β-glucan synthesis in sexual spores. In summary, the VosA-VelB hetero-complex is a key regulatory unit tightly controlling proper levels of β-glucan synthesis in asexual and sexual spores.

The cell wall is crucial for many aspects of fungal biology including growth, development, morphogenesis and pathogenicity^1^,^2^,^3^,^4^. It maintains the shape of the fugal cells and provides a protection against internal turgor pressure and various environmental factors^5^. The major components of the fungal cell wall are glycoproteins, chitin, α- glucans, β-glucans, mannans and melanin^6^,^7^. These components are cross-linked and form the fungal specific polysaccharide-based network^4^,^5^. Since the structure and components of cell wall are fungi-specific, the cell wall has been considered an attractive target for anti-fungal drug development^3^,^8^,^9^. In the model filamentous fungus *Aspergillus nidulans*, α-1,3-glucan, β-1,3-glucan and chitin are the main polysaccharides in the cell wall and associated genes include *agsA*, *agsB* (encoding α-1,3-glucan synthases), *fksA* (encoding β-1,3-glucan synthase) and *chsA* (encoding a chitin synthase)^10^,^11^,^12^,^13^,^14^.

Filamentous fungi produce asexual spores (conidia in higher fungi) as the main propagules and infection particles^15^,^16^. Conidia of *A. nidulans* are formed on the multicellular asexual reproductive structure conidiophore^15^,^17^. After conidia are formed from the phialide apex, it subsequently undergoes the maturation process consisting of three stages^18^,^19^,^20^. Stage I conidia contain walls consisting of C1 (outer) and C2 (inner) layers. At stage II, C1 becomes crenulated and C2 condenses. During stage III, two new wall layers, C3 (between C1 and C2) and C4 (the innermost wall) form and these two layers are required for conidial dormancy^19^. A key gene for conidia maturation in *A. nidulans* is *wetA*^19^. The *wetA6* mutant conidia are defective in the formation of the C3 and C4 wall layers and condensation of the C2 wall layer, resulting in the lack of spore pigmentation and lysis of conidia. Our studies further revealed that two *velvet* genes *vosA* and *velB* are also important for conidia maturation^21^,^22^,^23^.

The *velvet* proteins (VeA, VelB, VelC and VosA) are fungi-specific transcription factors and contain the *velvet* domain with the NF-kB-like DNA binding motif^21^,^24^,^25^,^26^. These proteins form different complexes, which play differential roles in regulating developmental processes and synthesis of secondary metabolites in many filamentous fungi^24^,^27^. Among them, the VosA-VelB complex acts as a functional unit controlling maturation (trehalose biogenesis), dormancy and germination of conidia^21^,^22^,^23^. In addition, the *vosA* deletion mutant conidia are defective in the formation of the electron-light layer of the conidial cell wall^21^, suggesting that the VosA protein may regulate proper spore wall formation.

To gain further insights into their role in the formation of spore wall, we have performed a transcriptome analysis using the wild type (WT) and Δ*vosA* mutant conidia. We have found that several genes involved in the synthesis of major cell wall polysaccharides are up-regulated by the lack of VosA in conidia. Further targeted studies have revealed that the absence of *vosA* or *velB* results in elevated mRNA accumulation of *fksA*, *gelA* and *gelB* involved in the biosynthesis of β-1,3-glucan. Indeed, levels of β-glucan in the mutant conidia were much higher than those in WT. In addition, *in vivo* protein-DNA interaction studies employing chromatin-immuno-precipitation (ChIP) using the VosA and VelB proteins tagged with the epitope FLAG specifically enriched an *fksA* promoter region which contains the consensus DNA sequence predicted to be recognized by VosA^25^. Moreover, the deletion of *vosA* causes increased levels of *fksA* mRNA and total β-glucan amount in sexual spores (ascospores), and VosA-FLAG-ChIP and VelB-FLAG-ChIP occupy an *fksA* promoter region. Taken together, we propose that the VosA-VelB complex represses β-glucan synthesis in fungal spores by directly binding to the promoter regions of *fksA* and other cell wall biosynthetic genes, thereby conferring proper spore wall formation during sporogenesis in *A. nidulans*.

## Results

### Genome-wide expression studies reveal that VosA is necessary for proper repression of cell wall-related genes in conidia

**Figure 1:**
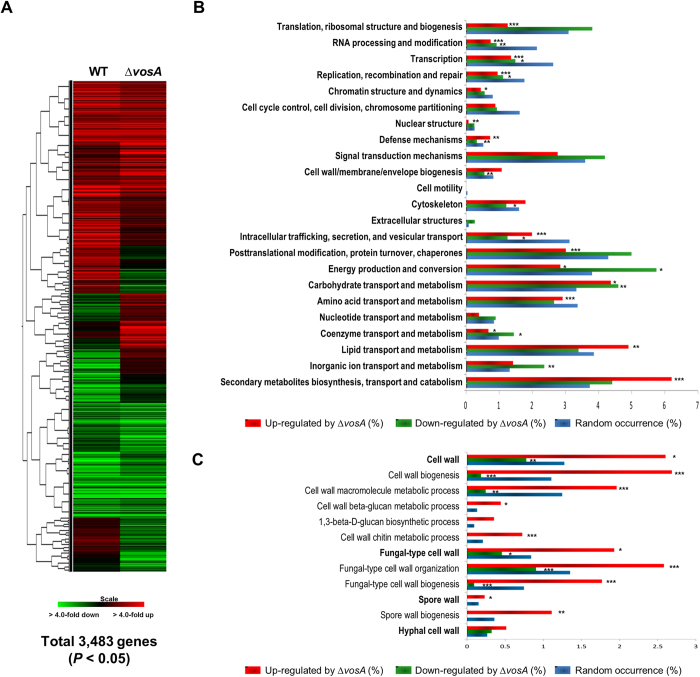
Genome wide analyses of *A. nidulans* genes whose expression is influenced by VosA in conidia. (**A**) Hierarchical clustering analysis of 3,483 genes which showed significant differential expression between the WT and ∆*vosA* conidia (P < 0.05, T-test) is presented. (**B**) Functional categories of up-, or down-regulated genes in the ∆*vosA* conidia compared to WT conidia are shown. Each group of genes was functionally categorized by KOG description (* P < 0.05; ** P < 0.01; *** P < 0.001). (**C**) Functional categories and expression responses of cell wall-, or sporulation-related genes in the ∆*vosA* conidia compared to WT conidia are presented. Each group of genes was functionally categorized by GO description (* P < 0.05; ** P < 0.01; *** P < 0.001).

To gain further insights into the complex regulatory roles of VosA during asexual sporogenesis, we carried out comparative transcriptome analysis of the WT and ∆*vosA* conidia. A total of 3,483 genes showed statistically significant differential expression between the ∆*vosA* and WT conidia (Fig. 1A, *P* < 0.05, T-test). A total of 2,117 genes exhibited more than two-fold reduction (1,104 genes), or induction (1,013 genes) in the ∆*vosA* conidia (Table S1). KOG (eukaryotic orthologous groups) analysis of genes up-regulated by VosA showed that the following categories of genes were overrepresented: cell wall/membrane/envelope biogenesis, cytoskeleton, lipid transport/metabolism, and secondary metabolites biogenesis/transport/catabolism (Fig. 1B). Further GO analyses demonstrated that a large number of the cell wall-related genes (the MIPS PEDANT 3 database, http://pedant.gsf.de/genomes.jsp?category=fungal) is differentially up-regulated in the ∆*vosA* conidia (Fig. 1C). Several genes involved in β-1,3-glucan synthesis including *fksA*, *gelA* and *gelB* (β-1,3-glucan-transglucosylase genes), and chitin synthesis including *chsA* (class II chitin synthase gene)^28^, *chsB* (class III chitin synthase gene)^29^,^30^, *csmA* (class V chitin synthase gene)^31^ and *gfaA* (glutamine-fructose 6-phosphate transaminase gene) were shown to be highly induced in the ∆*vosA* conidia compared to the WT conidia (Table 1). These indicate that VosA is involved in repression of cell wall-related genes in spores, and may govern the spore wall polysaccharides composition and structure.

### Both VelB and VosA are required for repression of spore wall-related genes

**Figure 2:**
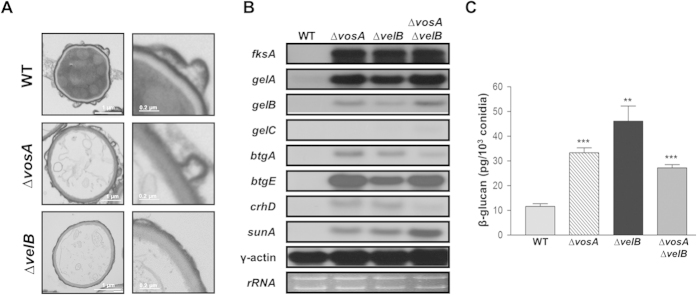
Requirement of *vosA* and *velB* in proper formation of conidial wall. (**A**) Transmission electron micrographs of WT (FGSC4), Δ*vosA* (THS15.1), Δ*velB* (THS16.1) mutant conidia are shown. All photomicrograph images were cropped (indicated by the red lines). (**B**) Levels of *fksA, gelA, gelB, gelC, btgA, btgE, crhD* and *sunA* mRNAs in conidia of WT (FGSC4), Δ*vosA* (THS15.1), *ΔvelB (*THS16.1) and Δ*velB* ∆*vosA* (THS14.1) strains. Equal loading of total RNA was confirmed by ethidium bromide staining of rRNA and hybridization with the γ-actin gene. All Northern blot and rRNA gel images were cropped (indicated by the red lines) to show the relevant data only. For each probe hybridization, equal amounts of total conidial RNA of four strains were separated in the same agarose gel, transferred to the one nylon membrane, and hybridized in the same bag. Thus, mRNA levels of each indicated gene can be directly compared among the four strains. (**C**) Amount of β-glucan (pg) per 10^3^ conidia in 2 day old conidia of WT (FGSC4), Δ*vosA* (THS15.1), Δ*velB* (THS16.1) and Δ*velB* ∆*vosA* (THS14.1) strains (measured in triplicates) (** P < 0.01; *** P < 0.001).

Previously we showed that VosA interacts with VelB, and forms the VosA-VelB complex which is a functional unit for spore maturation including trehalose biogenesis^22^,^23^. To test whether VelB plays a role in proper spore wall formation, we first examined 8 days old conidia of WT, ∆*velB* and ∆*vosA* strains using transmission electron microscopy (TEM) (Fig. 2A). We found that, similar to ∆*vosA*, the ∆*velB* conidia appear to lack cytoplasm and clear organelle structures. In addition, the *∆velB* conidia electron light layer was darker than that of WT. These results indicated that VelB, similar to VosA, plays a pivotal role in conidial wall formation and structure.

We then examined whether the absence of *velB* or *vosA* affected mRNA levels of key genes associated with synthesis and processing of β-1,3-glucan in conidia. We found that, while mRNA of 1,3-β-glucan synthetic genes including *fksA*, *gelA* and *gelB* was not detectable in WT conidia, mRNA levels of those genes were very high in the ∆*velB* and ∆*vosA* conidia (Fig. 2B). In accordance with the increased mRNA levels of β-1,3-glucan biosynthetic genes, β-glucan levels in the ∆*velB* and ∆*vosA* conidia were significantly higher than those of WT conidia (Fig. 2C). Moreover, the ∆*vosA* ∆*velB* double mutant conidia exhibited the essentially identical enhancement of mRNA accumulation patterns and increased β-glucan content in conidia (Fig. 2B,C). Taken together, these results suggest that both VelB and VosA are needed for proper repression of β-glucan synthesis in conidia, and they play an inter-dependent role.

### VosA and VelB bind to the *fksA* promoter

**Figure 3:**
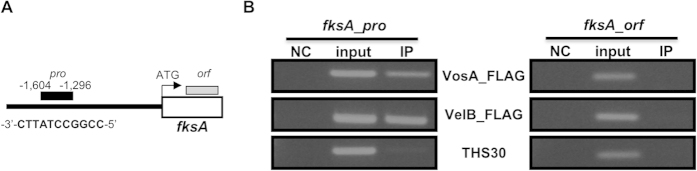
Both VosA and VelB bind to the *fksA* promoter in conidia. (**A**) Schematic presentation of the promoter region of *fksA*. The black box (the −1,296 ~ −1,604 region of the *fksA* promoter) and the gray box (a region of the *fksA* ORF) represents the regions subject to PCR (shown in B). The 11-nucleotide sequence predicted to be recognized by VosA is shown. (**B**) Results of VosA-ChIP-PCR: the PCR amplicons separated on a 2% agarose gel are shown in the bottom panel. The input DNA before immuno-precipitation (IP) was used as a positive control (input). The chromatin extract being incubated with bead only (without anti-FLAG antibody) was used as a negative control (NC). The samples of THS30 lacking FLAG-tagged VosA or VelB were used as negative controls, too. Representative results are shown (measured in duplicates). All gel images were cropped (indicated by the red lines) to show the relevant data only. The ChIP-PCR amplified samples were separated in the same agarose gel. The full-length gels are presented in Supplementary Fig. S1A online.

As mentioned, a key and common property of these multi-functional *velvet* regulators is that they are DNA-binding proteins and function as transcription factors. Their DNA-binding *velvet* domain is similar to NF-kB DNA binding motif^25^. Previous chromatin immune-precipitation followed by microarray (ChIP-chip) studies have revealed promoter regions of a high number of genes were enriched by VosA, including several cell-wall related genes (Table 1)^25^. One consensus DNA sequence predicted to be recognized by VosA is located at the promoter region of *fksA* (Fig. 3A). To test our hypothesis that the VelB and VosA hetero-complex is a functional unit for repression of β-glucan synthesis in conidia, we checked whether both VosA-ChIP and VelB-ChIP enriched the promoter regions of *fksA in vivo*. As shown Fig. 3B, PCR-amplification of the *fksA* promoter region using DNA fragments derived from VosA-FLAG-ChIP and VelB-FLAG-ChIP gave rise clear amplicons, whereas DNA from ChIP of THS30 lacking FLAG-tagged VosA or VelB failed to do so. Collectively, these results indicate that the VosA-VelB hetero-complex binds to the promoter region of *fksA* and negatively regulates expression of *fksA*, and many other conidial wall biosynthetic genes.

### VosA is required for β-glucan synthesis in ascospores

**Figure 4:**
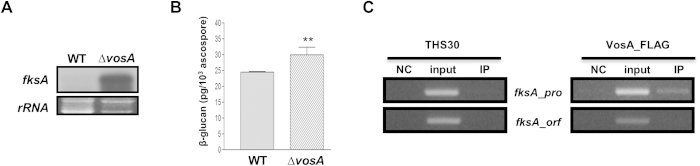
VosA represses β-glucan biogenesis in ascospores. (**A**) Levels of *fksA* transcript in ascospores of WT (FGSC4) and Δ*vosA* (THS15.1) strains. Equal loading of total RNA was confirmed by ethidium bromide staining of rRNA. (**B**) Amount of β-glucan (pg per 10^3^ ascospores) in 8 day old ascospores of WT (FGSC4) and Δ*vosA* (THS15.1) strains (measured in triplicates) (** P < 0.01). The Northern blot and rRNA gel images were cropped (indicated by the red lines). For *fksA* probe hybridization, equal amounts of total RNA of two strains’ ascospores were separated in the same agarose gel, transferred to the one nylon membrane, and hybridized in the same bag. (**C**) Results of VosA-ChIP-PCR: the PCR amplicons separated on a 2% agarose gel are shown in the bottom panel. The input DNA before immuno-precipitation (IP) was used as a positive control (input). The chromatin extract being incubated with bead only (without anti-FLAG antibody) was used as a negative control (NC). The samples of THS30 lacking FLAG-tagged VosA were also used as negative controls. Representative results are shown (measured in duplicates). All four gel images were cropped (indicated by the red lines) to show the relevant data only. The ChIP-PCR amplified samples were separated in the same agarose gel. The full-length gels are presented in Supplementary Fig. S1B online.

While asexual sporulation is the primary reproductive mode, *A. nidulans* can form sexual spores called ascospores. Our previous studies showed that, similar to conidia, the deletion of *vosA* caused the formation of defective ascospores with dis-jointed cell wall lacking long-term viability^21^. We envisioned that if VosA is generally required for proper conidial wall formation and structure, it would function similarly during sexual sporulation. The ∆*velB* mutant could not produce sexual fruiting bodies, and was excluded in ascospore analyses. To further test the roles of VosA in sexual fruiting, we examined mRNA levels of *fksA* and total content of β-1,3-glucan in WT and ∆*vosA* ascospores. As shown Fig. 4A, mRNA of *fksA* accumulated at high level in ∆*vosA*, but not in WT, ascospores. In addition, amount of β-glucan in ∆*vosA* ascospores was significantly higher than that of WT (Fig. 4B). Likewise in conidia, VosA-ChIP-PCR results show that VosA-ChIP specifically enriches the promoter regions of *fksA* in ascospores (Fig. 4C). Collectively, VosA represses expression of *fksA* and controls levels of β-1,3-glucan synthesis in ascospores.

## Discussion

The VosA and VelB regulators are fungi-specific transcription factors that play multifaceted roles in various biological processes including spore maturation, trehalose biogenesis, conidial germination and conidial wall integrity^21^,^22^,^25^. In this study, we have newly revealed following additional key roles of the VosA and VelB regulators during fungal sporogenesis. First, VosA negatively regulates expression of various genes including cell wall-related genes in conidia (Fig. 1, Table 1). Second, VosA and VelB directly bind to a promoter region of *fksA*, and thereby negatively control *fksA* expression and β-glucan synthesis in conidia (Figs. 2 and 3). Third, VosA plays a similar repressive role during sexual sporulation (Fig. 4). These results support that the VosA-VelB complex represses β-glucan synthesis in conidia and ascospores.

TEM results showed that 8 day old conidial wall of the *∆vosA* and *∆velB* mutant is different from that of WT. Our Northern blot analysis showed that *fksA* mRNA is detectable at relatively constant levels throughout the lifecycle of *A. nidulans* except in conidia (data not shown), suggesting that FksA may play a crucial role during fungal growth and development, but not in sporogenesis. While β-1,3-glucan is important for *A. nidulans* growth and development, the regulatory mechanism of β-1,3-glucan synthesis is largely unknown. Unlike *Saccharomyces cerevisiae*, transcription of *fksA* is regulated by non MpkA-RlmA pathway in *A. nidulans*^32^,^33^. We have revealed a new regulatory mechanism of *fksA* in *A. nidulans* that the VosA-VelB complex directly represses *fksA* expression in spores, thereby β-1,3-glucan synthesis.

Our discovery demonstrates that VosA plays a crucial role in both asexual and sexual spores. However, the role of VelB in *fksA* transcription and β-1,3-glucan synthesis could not be examined in sexual spores, because the ∆*velB* mutant fails to form any sexual fruiting bodies and sexual spores^23^,^34^. To predict the VelB’s role in β-1,3-glucan synthesis in sexual fruiting, we tested whether VelB-ChIP also enriches the promoter regions of *fksA* in ascospores. Our ChIP-PCR result showed that VelB-FLAG-ChIP enriched the *fksA* promoter region in ascospores (see Supplementary Fig. S1B online). Based on these results, we propose that the VosA-VelB hetero-complex directly regulates expression of *fksA* in both conidia and ascospores.

Beta-glucan is a fungal pathogen associated molecular pattern (PAMP) recognized by the pattern recognition receptors (PRRs) including Dectin-1 and Toll-like receptors (TLRs)^4^,^35^,^36^,^37^. This cell wall component triggers innate immune responses to fungal pathogens including *Aspergillus fumigatus*^38^,^39^,^40^. In *A. fumigatus*, the glucan synthase complex forms the catalytic and regulatory subunits^41^, where the *fks1* gene encodes the catalytic subunits. Previously, we have reported the conserved and distinct roles of the *velvet* genes in *A. fumigatus*^42^. To test whether VosA and VelB are involved in β-glucan synthesis in *A. fumigatus*, we briefly examined mRNA level of *fks1 A. fumigatus* conidia. Our preliminary Northern blot data indicated that deletion of *vosA* or *velB* caused increased *fks1* expression in *A. fumigatus* conidia (data not shown), suggesting that VosA and VelB may play a conserved repressive role in β-glucan synthesis in *A. fumigatus* spores.

Our microarray data also showed that expression levels genes associated with chitin synthesis, including *chsA*, *chsB, csmA* and *gfaA*, are up-regulated in the ∆*vosA* conidia compared to WT (Table 1). These genes are required for normal hyphal tip growth, septum formation, cell wall integrity and conidiophore development^31^,^43^,^44^,^45^,^46^. Previously we found that VosA-ChIP-chip enriched the promoter regions of *csmA* and *gfaA*^25^. In addition, expression of *csmA*, *gfaA*, and other chitin biosynthetic genes was ~2-3 fold elevated in the ∆*vosA* conidia compared to the WT (Table 1; see Supplementary Fig. S2 online). Based on these results, one can speculate that the VosA-VelB complex may also directly represses chitin biosynthetic genes and chitin levels in conidia. However, as some chitinase encoding genes (*chiB* and *chiC*) were also up-regulated in the ∆*vosA* conidia (Table 1), additional studies of the cell wall polysaccharides composition and structure in the ∆*vosA* and ∆*velB* conidia need to be carried out.

An additional discovery of our transcriptome analysis is that the deletion of *vosA* causes increased expression of genes associated with secondary metabolism in conidia. Particularly, the members of the sterigmatocystin (ST) biosynthesis gene cluster, including *stcW*, *stcV*, *stcT*, *stcQ*, *stcI*, *stcF*, *stcE*, *stcA*, and *stcL*, were up-regulated by mutation of *vosA*. It has been proposed that the dynamic formation of the *velvet* complexes may govern the determination of fungal cellular processes^47^. We speculate that the deletion of *vosA* results in increased formation of the VelB-VeA-LaeA trimeric complex, which is required for ST production.

**Figure 5:**
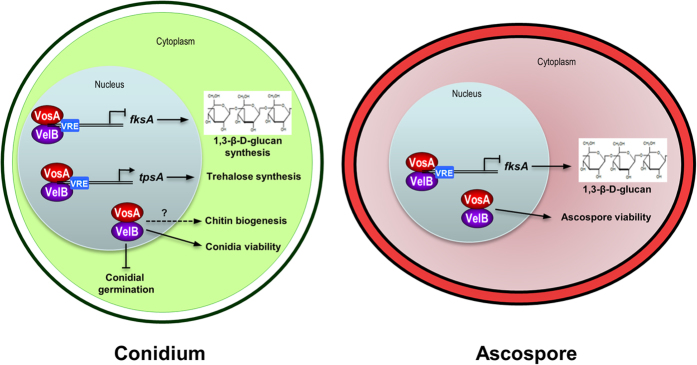
The roles of VosA and VelB in conidia and ascospores. A model depicting the regulatory roles of VosA and VelB in integrating morphological and chemical development during conidiogenesis and ascosporogenesis is shown (see Discussion).

Taken together, we propose a model for the proposed roles of the VosA-VelB complex in both asexual and sexual spores (Fig. 5). Previous studies demonstrated that the VosA-VelB complex directly and/or indirectly controls developmental regulatory genes or sporogenesis-related genes, thereby regulating spore maturation, spore-wall integrity, and trehalose biogenesis in fungal spores. In addition, this hetero-complex directly binds to the *fksA* promoter region and negatively regulates *fksA* expression in conidia and ascospores. Overall, our data further corroborate the idea that the VosA-VelB complex is a master regulatory unit for structure, metabolism and physiology in both asexual and sexual spores in *A. nidulans*.

## Methods

### Strains, media and culture conditions

*A. nidulans* strains used in this study are listed in Table 2. Strains were grown on solid minimal medium (MM), or sexual media (SM) with supplements as described previously^47^,^48^ at 37 ^o^C.

### Nucleic acid manipulation

The oligonucleotides used in this study are listed in Table 3. Total RNA isolation and Northern blot analyses were carried out as previously described^23^. The DNA probes were prepared by PCR-amplification of the coding regions of individual genes with appropriate oligonucleotide pairs using FGSC4 genomic DNA as a template.

### Microarray and data analysis

Two-day old conidia of ∆*vosA* (RNI14.1) and WT (FGSC4) strains were collected and ground in liquid N_2_ with a mortar and pestle. Total RNA was extracted by the acid-phenol method and harvested using CsCl density gradient ultracentrifugation. The fluorescent-labeled target cDNA probes were synthesized using the EZ start RNA linear amplification kit following manufacturer’s instructions (GenomicTree, Korea). Briefly, total RNA (1 μg) was reverse transcribed in the presence of 2 μg of 5′-CTACGCTGGGCCGACCGGGCGCGGGAC-3′-dT24 primer (Bioneer, Korea) at 42 °C for 2 h to generate single stranded cDNA. The reaction mixtures were further treated with RNase H (Invitrogen), and double stranded cDNAs were synthesized in the presence of *Escherichia coli* DNA ligase and DNA polymerase I (Invitrogen). Double stranded cDNAs were then purified by the Qiaquick PCR purification kit (Qiagen, USA), and further labeled with Aminoallyl-dUTP (Sigma, USA) in the presence of 5′-CTACGCTGGGCCGACCGGGCGCGGGAC-3′ primer (Bioneer) and Taq polymerase (Solgent). The amplified products were purified using a Qiaquick PCR purification kit (Qiagen), and further coupled with monofunctional cyanine dye as recommended by manufacturer (Amersham; Buckinghamshire, UK). The cDNAs synthesized from reference RNA were labeled with Cy3-dUTP, and cDNAs synthesized from total RNA taken from indicated time points were labeled with Cy5-dUTP. The labeled cDNA targets were purified using a Qiaquick purification kit (Qiagen), and dissolved in 100 μl of hybridization solution containing 5 × SSC, 0.1% SDS, 30% formamide, and 20 μg of salmon sperm DNA (Invitrogen). The hybridization mixtures were heated at 100 °C for 3 min and directly applied onto the 70-mer glass slide DNA microarrays (Tiger-PFGRC, USA). Two biological replicates were performed using cultures grown in parallel.

After hybridization, microarray slides were imaged using the laser scanner (Axon 4000B; Axon, USA). The signal and background fluorescence intensities were calculated for each probe spot by averaging the intensities of every pixel inside the target region using GenePix Pro 4.0 software (Axon). The signal intensity for each spot is the difference between the median pixel signal intensity and the median local background intensity. Spots exhibiting obvious abnormalities were excluded from analysis. All data normalization, statistical analyses and cluster analyses were performed using the GeneSpring 7.3.1 (Agilent, USA). Genes were further filtered according to their intensity in both channels, and thus the spots with average intensity of both channels ≥10 after normalization were considered as reliable ones.

The information of each *A. nidulans* gene that was downloaded from AspGD (http://www.aspergillusgenome.org/), the chromosomal feature of *A. nidulans* FGSC A4 was updated on 2 March, 2014. The functional categories of *A. nidulans* FGSC A4 were assigned using the MIPS PEDANT 3 database (http://pedant.gsf.de/genomes.jsp?category=fungal)^49^, KOGs database^50^ was from the version p3_p130_Asp_nidul (calculation date 11 August, 2007), GO database was from the version p3_r40559_Asp_nidul (calculation date 19 September, 2013).

### Transmission electron microscopy (TEM)

Samples were prepared following the methods as described^21^. Briefly, conidia were collected from the 8 day cultures on solid media. Samples were fixed in Karnovsky’s fixative, post –fixed in 2% osmium tetroxide, dehydrated in graded ethanol series. Polymerized samples were sectioned on a Leica UC6 ultramicrotome (80 nm) and stained with uranyl acetate and lead citrate. The stained sections were viewed on a JEOL 100CX transmission electron microscope, and documented with a SIS (Soft Imaging Systems, Lakewood, CO) MegaView III digital side mount camera.

### β-glucan analysis

The β-1,3-glucan amounts in spores were determined by the Glucatell^®^ assay (Associates of Cape Cod, USA) following the manufacturer’s instructions^51^. Briefly, two-day old conidia, or eight-day old ascospores of WT and mutants were collected in ddH_2_O. Samples of spores suspension (10^2 ^~ 10^5^) were prepared and resuspended in 25 μl of ddH_2_O. Each spores sample was mixed with 25 μl of Glucatell^®^ reagent and incubated at 37 ^o^C for 30 minutes. After incubation, diazo-reagents were added to stop the reaction, and optical density at 540 nm was determined. This test was performed in triplicate.

### Chromatin Immuno-precipitation followed by PCR (ChIP-PCR)

Samples were prepared following the methods as described^25^. Briefly, ChIP-PCR analysis was performed according to the manufacturer’s instructions with a minor modification using MAGnify Chromatin Immuno-precipitation System (Invitrogen). Two-day-old conidia or eight-day-old ascospores (1 × 10^9^) of each strain were cross-linked, washed and homogenized by a mini-bead beater. The cell lysates were sonicated for four cycles (30 s on, 60 s off) with a sonifier. The sonicated cell lysates were cleared by centrifugation. The diluted chromatin extracts were incubated with 2 μg of anti-FLAG antibody-Dynabeads complex and washed with the IP buffer. The input control and chromatin samples were eluted from the beads at 55 °C for 15 min with a reverse crosslinking buffer with Proteinase K. Enriched DNA was purified by DNA purification Magnetic Beads (Invitrogen). For amplification by PCR, the GO Taq DNA polymerase (Promega) was used. As negative controls, the chromatin extract being incubated with bead only (without anti-FLAG antibody) and samples of the fungal strain THS30 without FLAG-tagged VosA or VelB were used. Individual input DNA samples before immune-precipitation (IP) were used as positive controls. Two biological replicates have provided the essentially identical ChIP-PCR results. The primer sets used for PCR are shown in Table 3.

## Additional Information

**How to cite this article**: Park, H.-S. *et al*. *Velvet*-mediated repression of ß-glucan synthesis in *Aspergillus nidulans* spores. *Sci. Rep*. **5**, 10199; doi: 10.1038/srep10199 (2015).

## Supplementary Material

Supplementary Information

## Figures and Tables

**Table 1:** Relative expression of cell wall-related genes in Δ*vosA* and WT conidia.

Systematic name	Gene name	Description^a^	Expression ratio (∆*vosA*/WT)	Occupancy of VosA-ChIP^b^
**Genes involved in 1,3-β-glucan synthesis and processing**				
AN3729.3	*fksA*	1,3-beta-glucan synthase	5.01	O
AN7657.3	*gelA*	Putative 1,3-beta-transglycosidase	2.34	
AN0558.3	*gelB*	Putative 1,3-beta-transglycosidase	2.20	
AN3730.3	*gelC*	Putative 1,3-beta-transglycosidase	2.73	O
AN10150.3	*btgA*	Putative beta-transglucosylase	4.17	
AN1551.3	*btgE*	Putative beta-glucosidase	3.17	
AN3053.3	*crhD*	Putative transglycosidase	2.91	O
AN0550.3		Putative glucan 1,3-beta-glucosidase	0.22	
AN0779.3		Putative glucan 1,3-beta-glucosidase	5.97	
AN4825.3		Putative glucan 1,3-beta-glucosidase	2.43	
AN4852.3		Putative glucan 1,3-beta-glucosidase	2.67	
AN6697.3	*sunA*	Putative Sun-family protein	4.59	O

**Genes involved in 1,3-α-glucan synthesis and processing**				
AN5885.3	*agsA*	alpha-1,3 glucan synthase	6.11	
AN1604.3	*agnE*	Putative alpha-1,3-glucanase	2.22	
AN6324.3	*amyE*	Putative alpha-amylase	2.76	

**Genes involved in chitin synthesis and processing**				
AN7032.3	*chsA*	Class II chitin synthase	2.13	
AN2523.3	*chsB*	Class III chitin synthase	2.16	
AN6318.3	*csmA*	Class V chitin synthase	3.37	O
AN10709.3	*gfaA*	Glutamine-fructose-6-phosphate transaminase	2.43	O
AN4871.3	*chiB*	Class V chitinase	3.23	
AN9390.3	*chiC*	Putative endochitinase	11.33	
AN11059.3		Putative chitinase	2.30	
AN0221.3		Putative chitinase	0.43	
AN8481.3		Putative chitinase	0.31	
AN1502.3	*nagA*	Extracellular N-acetyl-beta-glucosaminidase	2.26	O

**Genes involved in other enzymatic function in cell wall biosynthesis**				
AN8444.3	*celA*	Protein with similarity to cellulose synthase	2.02	
AN3883.3		Putative glucanase	2.36	O
AN6472.3	*dfgF*	Putative endo-mannanase	4.07	
AN10345.3	*dfgA*	Putative endo-mannanase	2.00	
AN4390.3	*ecmA*	GPI-anchored cell wall organization protein	2.50	O

^a^ASPGD and de Groot *et al*., 2009.

^b^O represent genes bound by VosA and having VosA-responsive element (Ahmed *et al*., 2013).

**Table 2:** *Aspergillus* strains used in this study.

Strain name	Relevant genotype	References
FGSC4	*A. nidulans* wild type, *veA*^*+*^	FGSC^a^
RNI14.1	*biA1*; Δ*vosA*::*argB*^+^; *veA*^+^	Ni and Yu, 2007
THS14.1	*pyroA4*; Δ*vosA*::*pyroA*^+^; Δ*velB*::Afu*pyrG*^+^; *veA*^+^	Park et al., 2012a
THS15.1	*pyrG89*; *pyroA4*; Δ*vosA*::Afu*pyrG*^+^; *veA*^+^	Park et al., 2012a
THS16.1	*pyrG89*; *pyroA4*; Δ*velB*::Afu*pyrG*^+^; *veA*	Park et al., 2012a
THS20.1	*pyrG89*; *pyroA*::*velB*(p)::*velB*::FLAG_3x_::*pyroA*^b^; Δ*velB*::*AfupyrG*^+^; *veA*^+^	Park et al., 2012a
THS28.1	*pyrG89*; *pyroA*::*vosA*(p)::*vosA*::FLAG_3x_::*pyroA*^b^; Δ*vosA*::*AfupyrG*^+^; *veA*^+^	Park et al., 2012a
THS30.1	*pyrG89*; Afu*pyrG*^+^; *veA*^+^	This study

^a^Fungal Genetic Stock Center.

^b^The 3/4 *pyroA* marker causes targeted integration at the *pyroA* locus.

**Table 3:** Oligonucleotides used in this study

Name	Sequence (5′ → 3′)	Purpose
**OMN143**	ACTTATGCCAACGTTCTGCG	5′ *fksA* probe
**OMN144**	AAAGAGCGGGCAGCATAATG	3′ *fksA* probe
**OHS518**	GCTGCAAGCGTGACATTGCGAAG	5′ *gelA* probe
**OHS519**	GTACCGGCGTTCTGGCTACCAG	3′ *gelA* probe
**OHS520**	GTAAGTTCACCGAGGTTCAGGCC	5′ *gelB* probe
**OHS521**	AGGCAGGTAACCAGCACCGTTTG	3′ *gelB* probe
**OHS972**	ATGAAGTACTCTTTCGCCCTCACC	5′ *sunA* probe
**OHS973**	CTAGTAGAAGACGAAGGTAGCGTC	3′ *sunA* probe
**OHS974**	ATGACCCTTTTTCGAAACCTGGC	5′ *crhD* probe
**OHS975**	TTAGCGCACAGGCTGGTAGCC	3′ *crhD* probe
**OHS976**	ATGAGGGGAGCTATCCTGGCC	5′ *btgE* probe
**OHS977**	GTGCTGCAAGCAGCTCCAAC	3′ *btgE* probe
**OHS978**	ATGCGCGTCGCCGGGATTAT	5′ *btgA* probe
**OHS979**	CTAGTTCGGGCACGACAAATCG	3′ *btgA* probe
**OHS980**	ATGAAGTTCTCCAGCATCCTTGC	5′ *gelC* probe
**OHS981**	GATGCAGATGTGCCAGAGGGAC	3′ *gelC* probe
**OJA174**	CATTGAGCACGGTGTTGT	5′ *γ-actin* probe
**OJA175**	ATCCCTTGATCTCGTTTG	3′ *γ-actin* probe
**OHS622**	AGCCGAGCGATCAACTCTTG	5′ *fksA*_*pro*_ChIP-PCR
**OHS623**	CGATGTCTGGAACTGGTATGCAG	3′ *fksA*_*pro*_ChIP-PCR
**OHS624**	CTCGGTTGTGGATTCCTAATCTC	5′ *fksA*_*orf*_ChIP-PCR
**OHS625**	AATAGGCTCCATGCCCTTGG	3′ *fksA*_*orf*_ChIP-PCR
