# Supplementary material for: Velvet-mediated repression of β-glucan synthesis in Aspergillus nidulans spores
Source: Sci Rep. 2015 May 11;5:10199. doi: 10.1038/srep10199 (PMC4426670; doi:10.1038/srep10199)
Supplement: Supplementary Information [file srep10199-s1.pdf]

Supplementary Information

Velvet-mediated repression of β-glucan synthesis in *Aspergillus nidulans* spores

Hee-Soo Park[1], Yeong Man Yu[2], Mi-Kyung Lee[1], Pil Jae Maeng[2], Sun Chang Kim[3], and Jae-Hyuk Yu[1 *]

1 Department of Bacteriology, University of Wisconsin, Madison, WI, USA, **2** Department of Microbiology and Molecular Biology, Chungnam National University, Daejeon, Republic of Korea, **3** Department of Biological Sciences, Korea Advanced Institute of Science and Technology, Dae-Jon, Republic of Korea

[*] **Corresponding Author:**

Jae-Hyuk Yu

Departments of Bacteriology and Genetics

1550 Linden Drive, Madison, 53706, USA

Tel: 608-262-4696

Fax: 608-262-9865

Email: jyu1@wisc.edu

Running title: Repressing β-glucan synthesis in fungal spores.

Supplementary Figure S1. Results of VosA-ChIP-PCR and VelB-ChIP-PCR in spores.

Products of the VosA-FLAG-ChIP and VelB-FLAG-ChIP followed by PCR amplification of the *fksA* promoter (*fksA_pro*) or ORF (*fksA_orf*) regions from conidia (A), or ascospores (B) were separated in a 2% agarose gel. The input DNA before immuno-precipitation (IP) was used as a positive control (input). The chromatin extract being incubated with bead only (without anti-FLAG antibody) was used as a negative control (NC). The samples of THS30 lacking FLAG-tagged VosA or VelB were used as negative controls, too.

Supplementary Figure S2. Levels of *csmA*, *csmB*, *gfaA*, *agsB*, and *agsB* mRNAs in WT and mutants conidia.

Levels of *csmA, csmB, gfaA, agsA,* and *agsB* mRNAs in conidia of WT (FGSC4), Δ*vosA* (THS15.1), Δ*velB* (THS16.1) and Δ*velB* Δ*vosA* (THS14.1) strains are shown. Equal loading of total RNA was confirmed by ethidium bromide staining of rRNA. All Northern blot and rRNA gel images were cropped (indicated by the red lines) to show the relevant data only. For each probe hybridization, equal amounts of total conidial RNA of four strains were separated in the same agarose gel, transferred to the one nylon membrane, and hybridized in the same bag. Thus, mRNA levels of each indicated gene can be directly compared among the four strains.

Park *et al*. Supplementary Figure S1

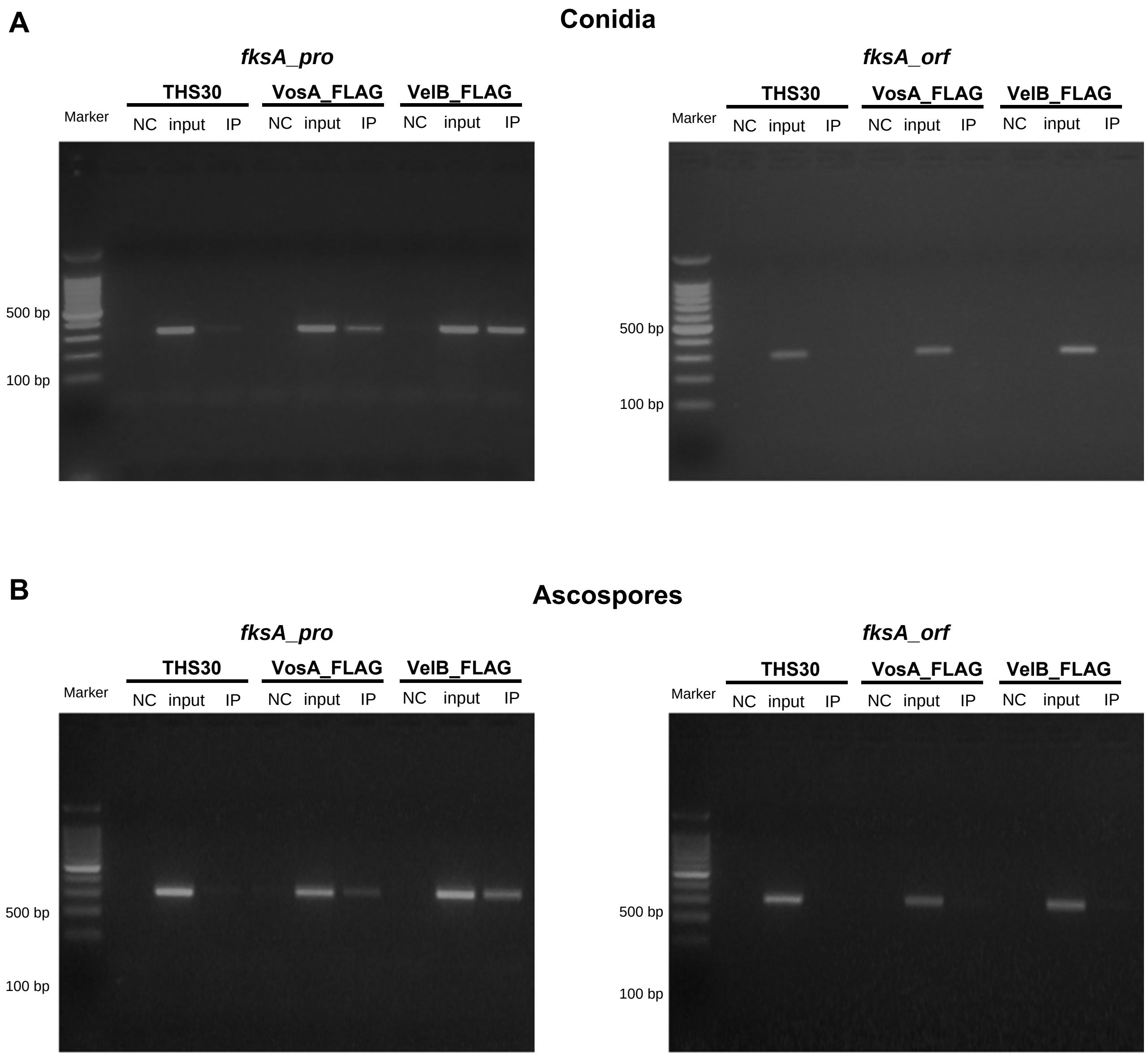

Park *et al*. Supplementary Figure S2

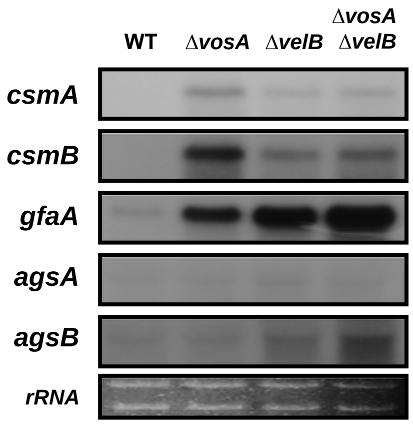

Supplementary Table 1.1. Genes exhibited more than twofold induction in the *ΔvosA* mutant conidia

: 1,013 genes

Systematic ID		Expession Ratio (Δ*vosA* /WT)	Description				Cell wall (GO Category from 'PEDANT3 - p3_r40559_Asp_nidul'[*4])												Sporulation (GO from 'PEDANT3 - p3_r40559_Asp_nidul'[*4])			
			Gene Name (AspGD[*1])	Transcription factor (DBD[*2])	Description (AspGD[*1])	KOGs[*3] Description & Category (PEDANT 3)	Cellular components GO:0005618 cell wall	Biological Process GO:0042546 cell wall biogenesis	Biological Process GO:0044036 cell wall macromolecule metabolic process	Biological Process GO:0034406 cell wall beta-glucan metabolic process	Biological Process GO:0006075 1,3-beta-D-glucan biosynthetic process	Biological Process GO:0006037 cell wall chitin metabolic process	Cellular compoment GO:0009277 fungal-type cell wall	Biological Process GO:0031505 fungal-type cell wall organization	Biological Process GO:0009272 fungal-type cell wall biogenesis	Cellular components GO:0031160 spore wall	Biological Process GO:0070590 spore wall biogenesis	Cellular compoment GO:0030446 hyphal cell wall	Biological Process GO:0009272 sporulation	Biological Process GO:0030436 asexual sporulation	Biological Process GO:0030293 sexual sporulation	Biological Process GO:0075259 spore-bearing organ development
AN7657.3	ANIA_07657	2.34	gelA		Putative 1,3-beta-transglycosidase with a predicted role in glucan processing; predicted glycosyl phosphatidylinositol (GPI)-anchor; palA-dependent expression independent of pH		cell wall	cell wall biogenesis	cell wall macromolecule metabolic process	cell wall beta-glucan metabolic process	(1->3)-beta-D-glucan biosynthetic process		fungal-type cell wall	fungal-type cell wall organization	fungal-type cell wall biogenesis		spore wall biogenesis	hyphal cell wall	sporulation		sexual sporulation	
AN7783.3	ANIA_07783	2.15			Ortholog(s) have Rho guanyl-nucleotide exchange factor activity	RhoGEF GTPase [T]		cell wall biogenesis	cell wall macromolecule metabolic process	cell wall beta-glucan metabolic process	(1->3)-beta-D-glucan biosynthetic process			fungal-type cell wall organization	fungal-type cell wall biogenesis		spore wall biogenesis		sporulation		sexual sporulation	
AN2687.3	ANIA_02687	2.59	rho4		Putative Rho-like GTPase	Ras-related small GTPase, Rho type [R]		cell wall biogenesis	cell wall macromolecule metabolic process	cell wall beta-glucan metabolic process	(1->3)-beta-D-glucan biosynthetic process			fungal-type cell wall organization	fungal-type cell wall biogenesis							
AN4782.3	ANIA_04782	2.63			Putative Rho-like GTPase	Ras-related small GTPase, Rho type [R]		cell wall biogenesis	cell wall macromolecule metabolic process	cell wall beta-glucan metabolic process	(1->3)-beta-D-glucan biosynthetic process			fungal-type cell wall organization	fungal-type cell wall biogenesis							
AN3729.3	ANIA_03729	5.01	fksA		Putative 1,3-beta-glucan synthase with a predicted role in glucan biosynthesis	1,3-beta-glucan synthase/callose synthase catalytic subunit [M]		cell wall biogenesis			(1->3)-beta-D-glucan biosynthetic process			fungal-type cell wall organization	fungal-type cell wall biogenesis		spore wall biogenesis		sporulation		sexual sporulation	
AN10150.3	ANIA_10150	4.17	btgA		Putative 1,3-beta-transglucosylase with a predicted role in glucan processing		cell wall	cell wall biogenesis	cell wall macromolecule metabolic process	cell wall beta-glucan metabolic process			fungal-type cell wall	fungal-type cell wall organization								
AN6318.3	ANIA_06318	3.37	csmA		Class V chitin synthase with a role in chitin biosynthesis; involved in hyphal tip growth; contains a myosin-like domain; mutation is synthetically lethal with csmB mutation; localized at hyphal tips and septa	Chitin synthase/hyaluronan synthase (glycosyltransferases) [M]		cell wall biogenesis	cell wall macromolecule metabolic process			cell wall chitin metabolic process	fungal-type cell wall	fungal-type cell wall organization	fungal-type cell wall biogenesis		spore wall biogenesis		sporulation	asexual sporulation	sexual sporulation	
AN2523.3	ANIA_02523	2.16	chsB		Class III chitin synthase with a role in chitin biosynthesis; required for normal growth and conidiation; constitutively expressed during vegetative growth, sexual and asexual development	Chitin synthase/hyaluronan synthase (glycosyltransferases) [M]		cell wall biogenesis	cell wall macromolecule metabolic process			cell wall chitin metabolic process		fungal-type cell wall organization	fungal-type cell wall biogenesis		spore wall biogenesis		sporulation	asexual sporulation	sexual sporulation	
AN7032.3	ANIA_07032	2.13	chsA		Class II chitin synthase with a role in chitin biosynthesis; involved with ChsC in septum formation and conidiation; transcript induced during asexual development; protein localizes to conidiophores	Chitin synthase/hyaluronan synthase (glycosyltransferases) [M]		cell wall biogenesis	cell wall macromolecule metabolic process			cell wall chitin metabolic process		fungal-type cell wall organization	fungal-type cell wall biogenesis		spore wall biogenesis		sporulation	asexual sporulation	sexual sporulation	
AN0799.3	ANIA_00799	2.02			Has domain(s) with predicted chitin synthase activity and role in chitin biosynthetic process	Chitin synthase/hyaluronan synthase (glycosyltransferases) [M]		cell wall biogenesis	cell wall macromolecule metabolic process			cell wall chitin metabolic process		fungal-type cell wall organization	fungal-type cell wall biogenesis		spore wall biogenesis			asexual sporulation	sexual sporulation	
AN10709.3	ANIA_10709	2.43	gfaA		Putative glutamine-fructose-6-phosphate transaminase	Glucosamine 6-phosphate synthetases, contain amidotransferase and phosphosugar isomerase domains [M]		cell wall biogenesis	cell wall macromolecule metabolic process			cell wall chitin metabolic process			fungal-type cell wall biogenesis							
AN5152.3	ANIA_05152	3.91			Ortholog of A. fumigatus Af293 : Afu1g07110, A. niger CBS 513.88 : An07g09650, A. oryzae RIB40 : AO090012000963, Aspergillus wentii : Aspwe1_0028295 and Aspergillus sydowii : Aspsy1_0091614			cell wall biogenesis	cell wall macromolecule metabolic process			cell wall chitin metabolic process										
AN3053.3	ANIA_03053	2.91	crhD		Putative transglycosidase with a predicted role in glucan processing		cell wall		cell wall macromolecule metabolic process			cell wall chitin metabolic process	fungal-type cell wall	fungal-type cell wall organization								
AN5586.3	ANIA_05586	2.78			Putative mannose-1-phosphate guanylyltransferase with a predicted role in mannose/mannitol, fructose, and sorbose/sorbitol metabolism	GDP-mannose pyrophosphorylase/mannose-1-phosphate guanylyltransferase [M]	cell wall	cell wall biogenesis	cell wall macromolecule metabolic process				fungal-type cell wall		fungal-type cell wall biogenesis							
AN0551.3	ANIA_00551	2.61			Putative mannosyl-oligosaccharide 1,2-alpha-mannosidase with a predicted role in mannose polymer metabolism	1, 2-alpha-mannosidase [G]		cell wall biogenesis	cell wall macromolecule metabolic process						fungal-type cell wall biogenesis							
AN7443.3	ANIA_07443	4.64			Ortholog of A. fumigatus Af293 : Afu2g06100, A. niger CBS 513.88 : An02g14710, A. oryzae RIB40 : AO090001000726, Aspergillus wentii : Aspwe1_0110234 and Aspergillus sydowii : Aspsy1_0152786			cell wall biogenesis	cell wall macromolecule metabolic process													
AN10496.3	ANIA_10496	2.95			Putative regulator of mannosylphosphorylation			cell wall biogenesis	cell wall macromolecule metabolic process													
AN3925.3	ANIA_03925	6.71			Has domain(s) with predicted catalytic activity, cation binding activity and role in carbohydrate metabolic process		cell wall		cell wall macromolecule metabolic process													
AN0542.3	ANIA_00542	2.15			Has domain(s) with predicted role in cell wall macromolecule catabolic process				cell wall macromolecule metabolic process													
AN8046.3	ANIA_08046	7.47			Putative triacylglycerol lipase with a predicted role in glycerolipid metabolism	Predicted lipase [I]			cell wall macromolecule metabolic process													
AN9458.3	ANIA_09458	3.40			Has domain(s) with predicted DNA binding, sequence-specific DNA binding RNA polymerase II transcription factor activity, zinc ion binding activity and role in regulation of transcription, DNA-dependent, transcription, DNA-templated				cell wall macromolecule metabolic process													
AN2810.3	ANIA_02810	3.17			Ortholog(s) have metallopeptidase activity	Meltrins, fertilins and related Zn-dependent metalloproteinases of the ADAMs family [O]	cell wall	cell wall biogenesis					fungal-type cell wall	fungal-type cell wall organization	fungal-type cell wall biogenesis	spore wall	spore wall biogenesis		sporulation		sexual sporulation	
AN5885.3	ANIA_05885	6.11	agsA		Catalytic subunit of alpha-1,3 glucan synthase complex; plays a minor role in alpha-1,3 glucan synthesis compared to AgsB; locus contains the conserved upstream open reading frame (uORF) AN5885-uORF	Alpha-amylase [G]	cell wall	cell wall biogenesis					fungal-type cell wall	fungal-type cell wall organization	fungal-type cell wall biogenesis	spore wall	spore wall biogenesis		sporulation		sexual sporulation	
AN4390.3	ANIA_04390	2.50	ecmA		Putative GPI-anchored protein with similarity to Saccharomyces cerevisiae Ecm33p; predicted role in cell wall biosynthesis		cell wall	cell wall biogenesis					fungal-type cell wall	fungal-type cell wall organization	fungal-type cell wall biogenesis		spore wall biogenesis		sporulation		sexual sporulation	
AN1837.3	ANIA_01837	3.04			Putative hydrophobin; predicted glycosyl phosphatidylinositol (GPI)-anchor		cell wall	cell wall biogenesis					fungal-type cell wall				spore wall biogenesis		sporulation	asexual sporulation		
AN6697.3	ANIA_06697	4.59	sunA		Putative Sun-family protein		cell wall	cell wall biogenesis														
AN0558.3	ANIA_00558	2.20	gelB		Putative 1,3-beta-transglycosidase with a predicted role in glucan processing; predicted glycosyl phosphatidylinositol (GPI)-anchor			cell wall biogenesis					fungal-type cell wall	fungal-type cell wall organization	fungal-type cell wall biogenesis		spore wall biogenesis					
AN3719.3	ANIA_03719	4.34	mpkB		MAP kinase, component of a signaling module SteD-SteC-MkkB-MpkB that controls coordination of development and secondary metabolism; phosphorylates VeA in vitro; mutant has moderate growth defect and arrested sexual development	Mitogen-activated protein kinase [T]		cell wall biogenesis						fungal-type cell wall organization	fungal-type cell wall biogenesis				sporulation		sexual sporulation	spore-bearing organ development
AN4447.3	ANIA_04447	2.48	hk-9		Putative histidine-containing phosphotransfer protein	Sensory transduction histidine kinase [T]		cell wall biogenesis							fungal-type cell wall biogenesis							
AN8444.3	ANIA_08444	2.02	celA		Protein with similarity to cellulose synthase; predicted role in beta-glucan synthesis			cell wall biogenesis									spore wall biogenesis		sporulation			spore-bearing organ development
AN6736.3	ANIA_06736	2.81			Ortholog(s) have unfolded protein binding activity			cell wall biogenesis														
AN10345.3	ANIA_10345	2.00	dfgA		Putative endo-mannanase GH76 family protein			cell wall biogenesis														
AN3730.3	ANIA_03730	2.73	gelC		Putative 1,3-beta-transglycosidase with a predicted role in glucan processing		cell wall						fungal-type cell wall	fungal-type cell wall organization				hyphal cell wall				
AN1222.3	ANIA_01222	2.51	sasA		Putative S-adenosylmethionine synthetase; predicted role in methionine metabolism; expression reduced after exposure to farnesol; strongly expressed during vegetative growth, downregulated during development in asexual or sexual cultures	S-adenosylmethionine synthetase [H]	cell wall						fungal-type cell wall					hyphal cell wall	sporulation		sexual sporulation	spore-bearing organ development
AN1131.3	ANIA_01131	2.83			Putative cytosolic Cu/Zn superoxide dismutase; transcript repressed by light in developmentally competent mycelia		cell wall						fungal-type cell wall					hyphal cell wall				
AN1551.3	ANIA_01551	3.17	btgE		Putative beta-glucosidase with predicted role in degradation of glucans; covalently bound cell wall protein		cell wall						fungal-type cell wall					hyphal cell wall				
AN4443.3	ANIA_04443	2.82	metH		Putative methionine synthase with a predicted role in methionine metabolism; protein expressed at increased levels in a hapX mutant versus wild-type	Methionine synthase II (cobalamin-independent) [E]	cell wall						fungal-type cell wall					hyphal cell wall				
AN4674.3	ANIA_04674	3.71			Ortholog(s) have role in asexual sporulation resulting in formation of a cellular spore, cellular response to drug and cell septum, cell surface localization	beta-1,6-N-acetylglucosaminyltransferase, contains WSC domain [G, O]	cell wall						fungal-type cell wall						sporulation	asexual sporulation		
AN5660.3	ANIA_05660	2.42	wscA		Putative plasma membrane sensor-transducer; N- and O-glycosylated and localized in the cell wall and membrane; mutants display a high frequency of swollen hyphae under hypo-osmotic conditions; required for conidiation	beta-1,6-N-acetylglucosaminyltransferase, contains WSC domain [G, O]	cell wall						fungal-type cell wall						sporulation	asexual sporulation		
AN6927.3	ANIA_06927	2.13	wscB		Putative plasma membrane sensor-transducer; N- and O-glycosylated and localized in the cell wall and membrane	beta-1,6-N-acetylglucosaminyltransferase, contains WSC domain [G, O]	cell wall						fungal-type cell wall						sporulation	asexual sporulation		
AN4604.3	ANIA_04604	3.22			Has domain(s) with predicted amino acid transmembrane transporter activity, role in amino acid transmembrane transport and membrane localization	Amino acid transporters [E]	cell wall						fungal-type cell wall									
AN7619.3	ANIA_07619	2.42	calA		Secreted thaumatin-like protein; role in early conidial germination; localized to the cell wall of germinating conidia but not to germ tubes; null appears wild-type, shows severe conidial germination defects with cetA calA double mutant		cell wall						fungal-type cell wall									
AN11059.3	ANIA_11059	2.30			Putative chitinase; glycoside hydrolase family 18 (GH18) protein with a predicted role in chitin hydrolysis	Chitinase [M]	cell wall						fungal-type cell wall									
AN6472.3	ANIA_06472	4.07	dfgF		Putative endo-mannanase GH76 family protein		cell wall							fungal-type cell wall organization								
AN1877.3	ANIA_01877	2.80			Ortholog of A. nidulans FGSC A4 : AN7228, A. fumigatus Af293 : Afu2g17490, A. niger CBS 513.88 : An04g09550, A. oryzae RIB40 : AO090102000061 and Neosartorya fischeri NRRL 181 : NFIA_092910	Flavin-containing monooxygenase [Q]	cell wall															

**AN6401.3**	**ANIA_06401**	**2.49**			Putative hydrophobin		cell wall					
**AN6685.3**	**ANIA_06685**	**3.15**			Ortholog(s) have role in cellular bud site selection, cellular protein localization, hyphal growth, positive regulation of establishment of bipolar cell polarity regulating cell shape		cell wall					
**AN7817.3**	**ANIA_07817**	**2.67**			Putative protein with a predicted role in sterigmatocystin/aflatoxin biosynthesis	Reductases with broad range of substrate specificities [R]	cell wall					
**AN8898.3**	**ANIA_08898**	**2.13**			Putative flavin-containing monooxygenase; expression upregulated after exposure to farnesol	Flavin-containing monooxygenase [Q]	cell wall					
**AN11121.3**	**ANIA_11121**	**2.66**			Protein of unknown function; transcript repressed by nitrate		cell wall					
**AN0973.3**	**ANIA_00973**	**3.09**	brlA	Transcription factor	C2H2 zinc finger transcription factor, fphA- and lreB-dependent light induced regulator of conidiophore development; locus has 2 overlapping transcriptional units called brlA alpha and brlA beta; see 5' brlA micro-ORF, AN0974			fungal-type cell wall organization	sporulation	asexual sporulation		spore-bearing organ development
**AN1394.3**	**ANIA_01394**	**3.06**	aspD		Putative septin	Septin CDC10 and related P-loop GTPases [D, T, Z]		fungal-type cell wall organization	sporulation	asexual sporulation		
**AN2290.3**	**ANIA_02290**	**2.44**	steA	Transcription factor	STE-like transcription factor with homeobox and zinc finger domains; null mutation blocks sexual cycle but not asexual development, forms Hulle cells but no ascogenous tissue nor cleistothecia	C2H2-type Zn-finger protein [K]		fungal-type cell wall organization				spore-bearing organ development
**AN1573.3**	**ANIA_01573**	**2.55**			Putative aspartic-type protease; predicted glycosylphosphatidylinositol (GPI)-anchored protein	Aspartyl protease [O]		fungal-type cell wall organization				
**AN6293.3**	**ANIA_06293**	**2.14**	acuM		Protein with role in utilization of gluconeogenic carbon sources; contains a Zn(2)-Cys(6) DNA-binding motif; ortholog of S. cerevisiae Rds2p			fungal-type cell wall organization				
**AN6503.3**	**ANIA_06503**	**2.05**		Transcription factor	Has domain(s) with predicted nucleic acid binding, zinc ion binding activity and intracellular localization	C2H2-type Zn-finger protein [K]		fungal-type cell wall organization				
**AN10763.3**	**ANIA_10763**	**2.82**	adaB		Subunit of the SAGA transcriptional regulatory complex with a role in nucleosome positioning	Histone acetyltransferase complex SAGA/ADA, subunit ADA2 [B]		fungal-type cell wall organization				
**AN2059.3**	**ANIA_02059**	**2.10**	velC		Velvet family protein with homology to VeA				sporulation	asexual sporulation	sexual sporulation	
**AN5028.3**	**ANIA_05028**	**6.17**	ppoC		Fatty acid oxygenase that plays a role in oxylipin biosynthesis; responsible for the formation of the psi-factor component psiB-beta; expression reduced after exposure to farnesol	Peroxidase/oxygenase [R]			sporulation	asexual sporulation	sexual sporulation	
**AN5730.3**	**ANIA_05730**	**2.31**			Ortholog of A. fumigatus Af293 : Afu1g06930, A. oryzae RIB40 : AO090003000083, Aspergillus wentii : Aspwe1_0109001, Aspergillus sydowii : Aspsy1_0043701 and Aspergillus terreus NIH2624 : ATET_02925				sporulation	asexual sporulation	sexual sporulation	
**AN0422.3**	**ANIA_00422**	**2.16**	abaA	Transcription factor	TEA/ATTS domain transcriptional activator involved in regulation of conidiation; required for phialide differentiation	TEF-1 and related K factor, TEAD family [K]			sporulation	asexual sporulation		spore-bearing organ development
**AN5836.3**	**ANIA_05836**	**2.88**	stuA	Transcription factor	APSES domain transcription factor involved in regulation of conidiophore development; represses abaA and other developmentally regulated genes; locus consists of stuA-alpha and stuA-beta transcriptional units; stuA-alpha contains a uORF				sporulation	asexual sporulation		spore-bearing organ development
**AN0453.3**	**ANIA_00453**	**3.43**	pclA		Putative G1/S cyclin; physically interacts with nimX; expression is transcriptionally regulated by BrlA and AbaA; mutants produce abnormal conidiophores with extra layers of phialides	Cyclin [R]			sporulation	asexual sporulation		
**AN1037.3**	**ANIA_01037**	**2.02**	odeA		Oleate delta-12 desaturase; transcriptionally regulated by low temperature and by light				sporulation	asexual sporulation		
**AN3129.3**	**ANIA_03129**	**2.02**	prpA		Poly(ADP-ribose) polymerase (PARP); predicted to function during the DNA damage response; transcript induced during asexual development and in response to the DNA damaging agents MMS, BLEO, 4-NQO and camptothecin	NAD+ ADP-ribosyltransferase Parp, required for poly-ADP ribosylation of nuclear proteins [K, L, O]			sporulation	asexual sporulation		
**AN5046.3**	**ANIA_05046**	**3.11**			Anisin-1; secreted defensin-like peptide with a predicted role in defense response; high homology to the mosquito defensin AaDefA1				sporulation	asexual sporulation		
**AN5829.3**	**ANIA_05829**	**4.46**			Has domain(s) with predicted phospholipid binding activity, role in cortical protein anchoring and cell cortex localization	Myosin class II heavy chain [Z]			sporulation	asexual sporulation		
**AN5893.3**	**ANIA_05893**	**2.49**	flbA		RGS (regulator of G-protein signaling) family member; negative regulator of G-protein signaling promoting conidiophore development; required for light-dependent activation of brlA transcription	G protein signaling regulators [T]			sporulation	asexual sporulation		
**AN6635.3**	**ANIA_06635**	**2.50**	yA		Conidial laccase (p-diphenol oxidase) involved in dark green pigment production of conidium wall; regulated by abaA; transcript accumulates during conidiophore development; mutants produce yellow colonies; upregulated in during hypoxia	Multicopper oxidases [Q]			sporulation	asexual sporulation		
**AN8752.3**	**ANIA_08752**	**4.64**			Ortholog of A. fumigatus Af293 : Afu6g02845, A. niger CBS 513.88 : An12g07700, Aspergillus wentii : Aspwe1_0048539, Aspergillus sydowii : Aspsy1_0092097 and Aspergillus terreus NIH2624 : ATET_07166					asexual sporulation		
**AN3075.3**	**ANIA_03075**	**2.78**	oefC	Transcription factor	Zinc finger protein of unknown function; overexpression confers the "fluffy" phenotype				sporulation		sexual sporulation	
**AN3761.3**	**ANIA_03761**	**2.02**		Transcription factor	Ortholog(s) have Golgi apparatus, cytosol localization	K factor NF-X1, contains NFX-type Zn2+-binding and R3H domains [K]			sporulation		sexual sporulation	
**AN5099.3**	**ANIA_05099**	**2.86**			Ortholog(s) have role in chromatin remodeling and Rpd3L-Expanded complex, Rpd3S complex localization				sporulation		sexual sporulation	
**AN5874.3**	**ANIA_05874**	**3.68**	llmG		Putative LaeA-like methyltransferase				sporulation		sexual sporulation	
**AN6280.3**	**ANIA_06280**	**2.33**			Ortholog of A. fumigatus Af293 : Afu2g12520, A. niger CBS 513.88 : An02g04750, A. oryzae RIB40 : AO090026000847, Aspergillus wentii : Aspwe1_0044488 and Aspergillus sydowii : Aspsy1_0150855	SNF2 family DNA-dependent ATPase [R]			sporulation		sexual sporulation	
**AN7570.3**	**ANIA_07570**	**2.40**	tubB		Alpha-tubulin, forms a heterodimer with beta-tubulin that promotes microtubule assembly; non-essential subunit required for meiosis and formation of ascospores	Alpha tubulin [Z]			sporulation		sexual sporulation	
**AN10516.3**	**ANIA_10516**	**2.38**	grrA		Putative F-box protein with a role in meiosis; NeddH-associated protein; required for ascospore development; transcript is highly expressed during sexual development	F-box protein containing LRR [R]			sporulation		sexual sporulation	
**AN4998.3**	**ANIA_04998**	**2.61**	gapA		Putative Ras GTPase-activating protein; required for normal cell polarity and conidiophore development	Ras GTPase-activating protein family - IQGAP [T]			sporulation			spore-bearing organ development
**AN0779.3**	**ANIA_00779**	**5.97**			Putative glucan 1,3-beta-glucosidase with a predicted role in glucan metabolism				sporulation			
**AN1475.3**	**ANIA_01475**	**2.01**	clipA		Protein that plays a role in microtubule dynamics; localizes to the plus-ends of microtubules	Microtubule-associated protein dynactin DCTN1/Glued [D, Z]			sporulation			
**AN2419.3**	**ANIA_02419**	**2.02**			Ortholog(s) have role in ascospore-type prospore assembly	SNAP-25 (synaptosome-associated protein) component of SNARE complex [U]			sporulation			
**AN8751.3**	**ANIA_08751**	**3.00**	sidB		Septation initiation network (SIN) component; required for septation and conidiation; interacts with MobA; localizes to spindle pole bodies and sites of septation; S. pombe Mob1p is required for the activity of the sidB ortholog, Sid2p	NDR and related serine/threonine kinases [R]			sporulation			
**AN5444.3**	**ANIA_05444**	**3.44**			Putative tryptophan synthase with a predicted role in aromatic amino acid biosynthesis	Tryptophan synthase beta chain [E]						spore-bearing organ development
**AN6150.3**	**ANIA_06150**	**2.48**	bud4		Cell polarity protein; involved in septum formation in hyphae and developing conidiophores; localizes to phialide-spore, vesicle-metulae and metulae-phialide junctions							spore-bearing organ development
**AN8182.3**	**ANIA_08182**	**4.21**	aspC		Septin, involved in development; prevents formation of inappropriate germ tubes and branches; required for formation of normal conidiophores	Septin family protein (P-loop GTPase) [D, Y, U]						spore-bearing organ development
**AN8340.3**	**ANIA_08340**	**2.03**			Has domain(s) with predicted role in transmembrane transport and integral to membrane localization	Sugar transporter/spinster transmembrane protein [G]						spore-bearing organ development
**AN10973.3**	**ANIA_10973**	**2.29**			Putative citrate synthase	Citrate synthase [C]						spore-bearing organ development
**AN0019.3**	**ANIA_00019**	**2.06**			Has domain(s) with predicted nucleotide binding activity	Flavonol reductase/cinnamoyl-CoA reductase [V]						
**AN0067.3**	**ANIA_00067**	**2.06**			Putative ribonucleotide reductase small subunit; transcript upregulated in response to camptothecin	Ribonucleotide reductase, beta subunit [F]						
**AN0100.3**	**ANIA_00100**	**2.18**			Has domain(s) with predicted nucleotide binding, oxidoreductase activity, transferase activity, transferring acyl groups other than amino-acyl groups, zinc ion binding activity and role in oxidation-reduction process	Alcohol dehydrogenase, class V [Q]						
**AN0122.3**	**ANIA_00122**	**2.73**			Has domain(s) with predicted ATP binding, ATP-dependent peptidase activity, serine-type endopeptidase activity and role in proteolysis	Mitochondrial ATP-dependent protease PIM1/LON [O]						
**AN0136.3**	**ANIA_00136**	**2.07**			Protein of unknown function							

**AN0137.3**	**ANIA_00137**	**2.85**			Ortholog(s) have glycerophosphocholine phosphodiesterase activity, role in cellular response to drug, glycerophospholipid catabolic process and cytosol, ribosome localization	Predicted starch-binding protein [R]
**AN0157.3**	**ANIA_00157**	**2.40**			Protein of unknown function	
**AN0167.3**	**ANIA_00167**	**2.73**			Ortholog(s) have enzyme regulator activity, guanyl-nucleotide exchange factor activity, translation initiation factor activity and role in regulation of translational initiation	Translation initiation factor 2B, alpha subunit (eIF-2Balpha/GCN3) [J]
**AN0169.3**	**ANIA_00169**	**2.25**			Protein of unknown function	
**AN0196.3**	**ANIA_00196**	**2.18**			Ortholog of A. fumigatus Af293 : Afu5g14200, Afu6g13810, Afu7g05110, Afu8g01720, A. niger CBS 513.88 : An13g02610, An15g02090, An04g09640 and A. oryzae RIB40 : AO090003001444	
**AN0230.3**	**ANIA_00230**	**2.69**			Ortholog of Neosartorya fischeri NRRL 181 : NFIA_102580, Aspergillus wentii : Aspwe1_0182725, Aspergillus versicolor : Aspve1_0035599 and Aspergillus zonatus : Aspzo1_0019331	
**AN0231.3**	**ANIA_00231**	**10.61**	ivoB		Conidiophore-specific phenol oxidase; mutant conidiophores, metulae and phialides lack pigmentation; ivoB mutants accumulate the substrate N-acetyl-6-hydroxytryptophan (AHT); repressed by light in developmentally competent mycelia	
**AN0260.3**	**ANIA_00260**	**3.86**			Putative DNA primase, large subunit; ortholog of S. cerevisiae PRI2; expression reduced after exposure to farnesol	Eukaryotic-type DNA primase, large subunit [L]
**AN0271.3**	**ANIA_00271**	**3.85**			Putative dUTP pyrophosphatase with a predicted role in pyrimidine metabolism; transcript upregulated in response to camptothecin	dUTPase [F]
**AN0273.3**	**ANIA_00273**	**2.69**			Putative transcription factor; transcript upregulated in response to camptothecin	
**AN0274.3**	**ANIA_00274**	**2.93**			Has domain(s) with predicted nucleic acid binding activity	
**AN0300.3**	**ANIA_00300**	**2.34**			Putative asparaginase with a predicted role in asparagine metabolism	Asparaginase [E]
**AN0301.3**	**ANIA_00301**	**5.15**			Ortholog of A. fumigatus Af293 : Afu1g02770, A. niger CBS 513.88 : An01g05370, A. oryzae RIB40 : AO090005000817, Aspergillus wentii : Aspwe1_0169626 and Aspergillus sydowii : Aspsy1_0144698	
**AN0307.3**	**ANIA_00307**	**3.12**			Putative F-box protein	
**AN0313.3**	**ANIA_00313**	**2.10**			Has domain(s) with predicted hydrolase activity and role in metabolic process	Arylacetamide deacetylase [V]
**AN0316.3**	**ANIA_00316**	**2.21**	tubA		Alpha-tubulin, forms a heterodimer with beta-tubulin that promotes microtubule assembly	Alpha tubulin [Z]
**AN0334.3**	**ANIA_00334**	**2.48**			Ortholog of A. fumigatus Af293 : Afu1g02340, A. oryzae RIB40 : AO090005000866, Aspergillus wentii : Aspwe1_0169667 and Aspergillus sydowii : Aspsy1_0145773	
**AN0338.3**	**ANIA_00338**	**2.07**	CYP680A1		Putative cytochrome P450	Cytochrome P450 CYP11/CYP12/CYP24/CYP27 subfamilies [Q]
**AN0370.3**	**ANIA_00370**	**2.70**			Has domain(s) with predicted catalytic activity and role in metabolic process	Enoyl-CoA hydratase [I]
**AN0404.3**	**ANIA_00404**	**2.16**			Has domain(s) with predicted ATP binding, ATPase activity, coupled to transmembrane movement of substances activity, role in transmembrane transport and integral to membrane localization	Multidrug/pheromone exporter, ABC superfamily [Q]
**AN0416.3**	**ANIA_00416**	**2.11**			Ortholog(s) have Golgi apparatus localization	
**AN0435.3**	**ANIA_00435**	**2.32**			Ortholog of A. nidulans FGSC A4 : AN10356, A. fumigatus Af293 : Afu1g04510, Afu3g13080, A. niger CBS 513.88 : An01g03600 and A. oryzae RIB40 : AO090003000870, AO090012000821	
**AN0443.3**	**ANIA_00443**	**2.63**			Putative zinc containing alcohol dehydrogenase; protein expressed at decreased levels in a hapX mutant versus wild-type	Sorbitol dehydrogenase [Q]
**AN0461.3**	**ANIA_00461**	**3.50**			Ortholog of A. fumigatus Af293 : Afu1g04370, A. niger CBS 513.88 : An01g03230, A. oryzae RIB40 : AO090003000912, Aspergillus wentii : Aspwe1_0025386 and Aspergillus sydowii : Aspsy1_0055307	Vesicle coat complex AP-3, beta subunit [U]
**AN0466.3**	**ANIA_00466**	**2.59**			Ortholog of A. fumigatus Af293 : Afu1g04315, A. oryzae RIB40 : AO090003000918, Aspergillus wentii : Aspwe1_0037360 and Aspergillus sydowii : Aspsy1_0041113	
**AN0467.3**	**ANIA_00467**	**3.00**			Ortholog of A. nidulans FGSC A4 : AN0468, A. fumigatus Af293 : Afu1g01350, Afu1g04310, A. niger CBS 513.88 : An12g10460 and Aspergillus wentii : Aspwe1_0169394	
**AN0482.3**	**ANIA_00482**	**2.63**			Putative ubiquitin-conjugating enzyme; transcript repressed by nitrate	Predicted ubiquitin-conjugating enzyme [O]
**AN0497.3**	**ANIA_00497**	**3.88**			Has domain(s) with predicted role in transmembrane transport and integral to membrane localization	Permease of the major facilitator superfamily [G]
**AN0539.3**	**ANIA_00539**	**3.30**			Ortholog of Aspergillus kawachii : Aspka1_0181433 and Aspergillus acidus : Aspfo1_0142555	O-linked N-acetylglucosamine transferase OGT [G, O, T]
**AN0540.3**	**ANIA_00540**	**4.47**			Ortholog of Neosartorya fischeri NRRL 181 : NFIA_056010, Aspergillus versicolor : Aspve1_0065547 and Aspergillus sydowii : Aspsy1_0048566, Aspsy1_0081349	
**AN0546.3**	**ANIA_00546**	**7.76**			Protein of unknown function	
**AN0572.3**	**ANIA_00572**	**2.06**			Ortholog of A. fumigatus Af293 : Afu6g11290, A. niger CBS 513.88 : An08g07465, Aspergillus wentii : Aspwe1_0027109 and Aspergillus sydowii : Aspsy1_0086207	
**AN0585.3**	**ANIA_00585**	**4.40**			Has domain(s) with predicted sequence-specific DNA binding RNA polymerase II transcription factor activity, zinc ion binding activity, role in regulation of transcription, DNA-dependent and nucleus localization	
**AN0588.3**	**ANIA_00588**	**3.01**			Protein of unknown function	
**AN0606.3**	**ANIA_00606**	**5.19**	CYP5076A1		Putative cytochrome P450	Cytochrome P450 CYP4/CYP19/CYP26 subfamilies [Q, I]
**AN0638.3**	**ANIA_00638**	**3.03**			Ortholog of A. nidulans FGSC A4 : AN6319, A. fumigatus Af293 : Afu1g16870, A. oryzae RIB40 : AO090005000988, Aspergillus wentii : Aspwe1_0038696 and Aspergillus sydowii : Aspsy1_0129541, Aspsy1_0995740	
**AN0650.3**	**ANIA_00650**	**2.40**			Protein of unknown function	
**AN0664.3**	**ANIA_00664**	**2.85**	plcA		Putative 1-phosphatidylinositol-4,5-bisphosphate phosphodiesterase with a predicted role in phospholipid metabolism; directly linked to high-molecular-weight carbon source sensing	Phosphoinositide-specific phospholipase C [T]
**AN0666.3**	**ANIA_00666**	**2.77**			Has domain(s) with predicted hydrolase activity and role in metabolic process	Arylacetamide deacetylase [V]
**AN0683.3**	**ANIA_00683**	**2.42**		Transcription factor	Predicted mariner transposon-related ORF	
**AN0709.3**	**ANIA_00709**	**3.37**		Transcription factor	Putative zinc-finger protein; expression upregulated after exposure to farnesol	
**AN0722.3**	**ANIA_00722**	**2.36**			Ortholog(s) have AP-2 adaptor complex, cytosol, nucleus localization	Clathrin adaptor complex, small subunit [U]
**AN0732.3**	**ANIA_00732**	**2.49**			Putative transporter of the major facilitator superfamily (MFS); expression upregulated after exposure to farnesol	Synaptic vesicle transporter SVOP and related transporters (major facilitator superfamily) [R]
**AN0734.3**	**ANIA_00734**	**2.64**	H4.1		Histone H4.1, core histone protein; nearly identical to histone H4.2	Histone H4 [B]
**AN0736.3**	**ANIA_00736**	**6.00**			Ortholog of A. nidulans FGSC A4 : AN2423, A. fumigatus Af293 : Afu1g14230, Afu2g13800, A. niger CBS 513.88 : An01g12240, An02g05360 and A. oryzae RIB40 : AO090026000195	
**AN0781.3**	**ANIA_00781**	**4.31**			Ortholog of A. fumigatus Af293 : Afu1g14500, A. niger CBS 513.88 : An01g12470, A. oryzae RIB40 : AO090011000360, Aspergillus wentii : Aspwe1_0112044 and Aspergillus sydowii : Aspsy1_0145580	
**AN0783.3**	**ANIA_00783**	**2.03**			Has domain(s) with predicted carbon-nitrogen ligase activity, with glutamine as amido-N-donor, hydrolase activity, acting on carbon-nitrogen (but not peptide) bonds, in linear amides activity	Amidases [J, I, T]
**AN0785.3**	**ANIA_00785**	**2.18**	sodM		Putative manganese superoxide dismutase	Manganese superoxide dismutase [P]
**AN0787.3**	**ANIA_00787**	**3.37**	mns1B		Putative mannosyl-oligosaccharide 1,2-alpha-mannosidase with a predicted role in mannose polymer metabolism	Mannosyl-oligosaccharide alpha-1,2-mannosidase and related glycosyl hydrolases [G]

**AN0788.3**	**ANIA_00788**	**3.48**			Ortholog of A. niger CBS 513.88 : An09g02430, An03g05960, An09g00710, An09g00820, An09g01710 and A. oryzae RIB40 : AO090005000954, AO090003000250, AO090012000369, AO090010000570, AO090023000882, AO090120000022, AO090012000900	Soluble epoxide hydrolase [I]
**AN0789.3**	**ANIA_00789**	**2.99**			Has domain(s) with predicted adenyl nucleotide binding activity	Predicted membrane protein, contains two CBS domains [S]
**AN0790.3**	**ANIA_00790**	**2.17**			Ortholog(s) have intracellular localization	
**AN0791.3**	**ANIA_00791**	**2.83**			Protein of unknown function	
**AN0823.3**	**ANIA_00823**	**2.43**			Ortholog of A. fumigatus Af293 : Afu1g14830, A. niger CBS 513.88 : An01g12940, A. oryzae RIB40 : AO090005001249, Aspergillus wentii : Aspwe1_0171783 and Aspergillus sydowii : Aspsy1_0053537	Uncharacterized conserved protein [S]
**AN0826.3**	**ANIA_00826**	**2.25**		Transcription factor	Predicted DDE1 transposon-related ORF	
**AN0828.3**	**ANIA_00828**	**2.26**			Putative amidase; expression upregulated after exposure to farnesol	Amidases [J]
**AN0849.3**	**ANIA_00849**	**2.60**			Ortholog of A. nidulans FGSC A4 : AN6081, A. fumigatus Af293 : Afu1g15080, Afu2g09190, A. niger CBS 513.88 : An01g13240, An12g04840 and A. oryzae RIB40 : AO090005001220, AO090011000776	
**AN0854.3**	**ANIA_00854**	**2.81**			Ortholog of A. fumigatus Af293 : Afu1g15110, A. niger CBS 513.88 : An01g13270, A. oryzae RIB40 : AO090005001214, Neosartorya fischeri NRRL 181 : NFIA_010310 and Aspergillus wentii : Aspwe1_0051140	
**AN0860.3**	**ANIA_00860**	**2.48**			Ortholog of A. fumigatus Af293 : Afu1g15260, A. niger CBS 513.88 : An01g13370, A. oryzae RIB40 : AO090103000449, AO090005001207 and Aspergillus wentii : Aspwe1_0111296	
**AN0867.3**	**ANIA_00867**	**3.70**			Ortholog of A. nidulans FGSC A4 : AN4122, AN2881, AN10123, A. fumigatus Af293 : Afu1g13860, Afu1g15180, Afu3g11650 and A. niger CBS 513.88 : An08g07010, An02g07440, An01g13480	
**AN0875.3**	**ANIA_00875**	**2.05**			Ortholog of A. fumigatus Af293 : Afu1g15350, A. niger CBS 513.88 : An01g13580, A. oryzae RIB40 : AO090005001181, Aspergillus wentii : Aspwe1_0027582 and Aspergillus sydowii : Aspsy1_0053487	
**AN0890.3**	**ANIA_00890**	**2.14**			Ortholog(s) have fungal-type vacuole membrane localization	Permease of the major facilitator superfamily [R]
**AN0902.3**	**ANIA_00902**	**2.22**		Transcription factor	Has domain(s) with predicted sequence-specific DNA binding RNA polymerase II transcription factor activity, zinc ion binding activity, role in regulation of transcription, DNA-dependent and nucleus localization	
**AN0903.3**	**ANIA_00903**	**2.58**			Ortholog of A. fumigatus Af293 : Afu1g15690, A. niger CBS 513.88 : An01g14030, A. oryzae RIB40 : AO090005001138, Aspergillus wentii : Aspwe1_0027550 and Aspergillus sydowii : Aspsy1_0139000	
**AN0904.3**	**ANIA_00904**	**4.51**			Ortholog of A. fumigatus Af293 : Afu1g15700, A. niger CBS 513.88 : An01g14040, A. oryzae RIB40 : AO090005001136, Aspergillus wentii : Aspwe1_0027549 and Aspergillus sydowii : Aspsy1_0141245	
**AN0939.3**	**ANIA_00939**	**3.84**			Has domain(s) with predicted catalytic activity and role in metabolic process	
**AN0940.3**	**ANIA_00940**	**3.03**			Putative hydrophobin	
**AN0942.3**	**ANIA_00942**	**3.03**	ladA		Putative L-arabinitol 4-dehydrogenase with a predicted role in L-arabinose/arabitol and D-xylose/D,L-xylulose/xylitol metabolism; transcriptionally induced by growth on xylose	Sorbitol dehydrogenase [Q]
**AN0964.3**	**ANIA_00964**	**14.21**			Ortholog of A. fumigatus Af293 : Afu1g16640, A. niger CBS 513.88 : An01g10620, A. oryzae RIB40 : AO090005001050, Aspergillus wentii : Aspwe1_0039876 and Aspergillus sydowii : Aspsy1_0138532	
**AN0975.3**	**ANIA_00975**	**2.22**		Transcription factor	Predicted DDE1 transposon-related ORF	
**AN0977.3**	**ANIA_00977**	**2.58**			Putative peroxin with a predicted role in peroxisome biogenesis	
**AN0985.3**	**ANIA_00985**	**3.12**			Protein of unknown function	
**AN0986.3**	**ANIA_00986**	**2.12**			Ortholog of A. fumigatus Af293 : Afu1g16745, A. niger CBS 513.88 : An01g10200, A. oryzae RIB40 : AO090005001009, Aspergillus wentii : Aspwe1_0171600 and Aspergillus sydowii : Aspsy1_0140408	
**AN0992.3**	**ANIA_00992**	**2.61**			Has domain(s) with predicted nucleotide binding activity	
**AN1031.3**	**ANIA_01031**	**2.73**	afoB		Putative efflux pump; involved in asperfuranone biosynthesis	Predicted transporter (major facilitator superfamily) [R]
**AN1032.3**	**ANIA_01032**	**2.40**	afoC		Putative oxidoreductase; required for asperfuranone biosynthesis	Phospholipase/carboxyhydrolase [E]
**AN1035.3**	**ANIA_01035**	**7.52**	afoF		Putative FAD/FMN-dependent oxygenase; required for asperfuranone biosynthesis	Proteins containing the FAD binding domain [C]
**AN1055.3**	**ANIA_01055**	**2.93**			Ortholog(s) have eukaryotic initiation factor 4G binding, mRNA binding activity	Uncharacterized mRNA-associated protein RAP55 [U]
**AN1058.3**	**ANIA_01058**	**2.24**			Ortholog of A. fumigatus Af293 : Afu1g12350, A. oryzae RIB40 : AO090001000294, Aspergillus wentii : Aspwe1_0172072, Aspergillus sydowii : Aspsy1_0053315 and Aspergillus terreus NIH2624 : ATET_00433	Defense-related protein containing SCP domain [S]
**AN1062.3**	**ANIA_01062**	**2.04**			Has domain(s) with predicted fatty-acyl-CoA binding activity	Acyl-CoA-binding protein [I]
**AN1071.3**	**ANIA_01071**	**3.25**			Has domain(s) with predicted ADP binding, microtubule motor activity, role in nucleoside metabolic process and kinesin complex localization	Kinesin light chain [Z]
**AN1077.3**	**ANIA_01077**	**3.04**			Has domain(s) with predicted DNA binding, sequence-specific DNA binding RNA polymerase II transcription factor activity, zinc ion binding activity and role in regulation of transcription, DNA-dependent, transcription, DNA-templated	
**AN1080.3**	**ANIA_01080**	**3.11**			Has domain(s) with predicted translation initiation factor activity, role in translational initiation and cytoplasm localization	Translation initiation factor 4F, cap-binding subunit (eIF-4E) and related cap-binding proteins [J]
**AN1088.3**	**ANIA_01088**	**2.03**			Ortholog of A. fumigatus Af293 : Afu4g14470, Afu8g00430, A. niger CBS 513.88 : An07g00070, An07g00010, An02g08300, An11g06450 and A. oryzae RIB40 : AO090001000323	
**AN1130.3**	**ANIA_01130**	**2.05**			Ortholog of A. nidulans FGSC A4 : AN5168, A. fumigatus Af293 : Afu6g07030, Afu4g13872, A. niger CBS 513.88 : An15g06750, An07g01930 and A. oryzae RIB40 : AO090701000916	Ankyrin [M]
**AN1137.3**	**ANIA_01137**	**2.38**	qutB		Putative quinate 5-dehydrogenase with a predicted role in aromatic amino acid biosynthesis	
**AN1144.3**	**ANIA_01144**	**3.00**			Has domain(s) with predicted peptidase activity	
**AN1156.3**	**ANIA_01156**	**4.68**			Ortholog of A. fumigatus Af293 : Afu1g11450, A. niger CBS 513.88 : An08g03570, A. oryzae RIB40 : AO090038000280, Aspergillus wentii : Aspwe1_0068767 and Aspergillus sydowii : Aspsy1_0191007	Myosin class II heavy chain [Z]
**AN1160.3**	**ANIA_01160**	**4.86**			Predicted glycosylphosphatidylinositol (GPI)-anchored protein; S. cerevisiae ortholog Srl1p has role in cell wall organization	
**AN1173.3**	**ANIA_01173**	**2.08**			Ortholog of A. fumigatus Af293 : Afu1g11060, A. niger CBS 513.88 : An08g03320, Aspergillus wentii : Aspwe1_0024838, Aspergillus sydowii : Aspsy1_0038077 and Aspergillus terreus NIH2624 : ATET_00305	Uncharacterized conserved protein [S]
**AN1182.3**	**ANIA_01182**	**2.63**	benA		Beta-tubulin, highly conserved component of microtubules; A. nidulans has two beta-tubulin genes, benA and tubC; temperature sensitive mutants are blocked in mitosis and in nuclear division	Beta tubulin [Z]
**AN1197.3**	**ANIA_01197**	**3.39**			Has domain(s) with predicted catalytic activity	
**AN1202.3**	**ANIA_01202**	**2.12**	ndxA		Conserved nudix hydrolase isozyme	NADH pyrophosphatase I of the Nudix family of hydrolases [L]
**AN1242.3**	**ANIA_01242**	**3.28**			Putative nonribosomal peptide synthase (NRPS) involved in nidulanin A biosynthesis; predicted backbone enzyme of the secondary metabolite gene cluster	Non-ribosomal peptide synthetase/alpha-aminoadipate reductase and related enzymes [Q]
**AN1250.3**	**ANIA_01250**	**7.49**			Ortholog of A. fumigatus Af293 : Afu1g10220, A. niger CBS 513.88 : An08g02090, Neosartorya fischeri NRRL 181 : NFIA_015430 and Aspergillus versicolor : Aspve1_0119420	
**AN1251.3**	**ANIA_01251**	**5.44**		Transcription factor	Has domain(s) with predicted nucleic acid binding, zinc ion binding activity and intracellular localization	

**AN1258.3**	**ANIA_01258**	**2.35**			Ortholog of A. fumigatus Af293 : Afu1g10210, A. niger CBS 513.88 : An08g02080, A. oryzae RIB40 : AO090038000405, Aspergillus wentii : Aspwe1_0103837 and Aspergillus sydowii : Aspsy1_0083479	
**AN1261.3**	**ANIA_01261**	**2.82**			Protein of unknown function; transcript is induced by nitrate	
**AN1263.3**	**ANIA_01263**	**2.06**			Putative adenosylhomocysteinase with a predicted role in methionine metabolism; expression reduced after exposure to farnesol	S-adenosylhomocysteine hydrolase [H]
**AN1279.3**	**ANIA_01279**	**2.00**			Ortholog of A. fumigatus Af293 : Afu1g09880, A. niger CBS 513.88 : An08g01690, Neosartorya fischeri NRRL 181 : NFIA_015750 and Aspergillus versicolor : Aspve1_0068298	
**AN1321.3**	**ANIA_01321**	**2.11**			Ortholog of Aspergillus versicolor : Aspve1_0068355	
**AN1322.3**	**ANIA_01322**	**2.33**			Has domain(s) with predicted GTP binding, GTPase activity	Vacuolar sorting protein VPS1, dynamin, and related proteins [U, R]
**AN1424.3**	**ANIA_01424**	**2.25**			Ortholog of A. fumigatus Af293 : Afu8g04140, A. niger CBS 513.88 : An16g08950, A. oryzae RIB40 : AO090103000029, Aspergillus wentii : Aspwe1_0043821 and Aspergillus sydowii : Aspsy1_0041972	Myosin class II heavy chain [Z]
**AN1426.3**	**ANIA_01426**	**4.34**			Ortholog(s) have serine-type carboxypeptidase activity	Serine carboxypeptidases (lysosomal cathepsin A) [E, O]
**AN1437.3**	**ANIA_01437**	**2.34**		Transcription factor	Has domain(s) with predicted zinc ion binding activity and intracellular localization	
**AN1438.3**	**ANIA_01438**	**3.90**			Ortholog of A. fumigatus Af293 : Afu8g04290, Aspergillus fumigatus A1163 : AFUB_083260 and Aspergillus sydowii : Aspsy1_0142928	
**AN1443.3**	**ANIA_01443**	**3.13**			Ortholog of A. fumigatus Af293 : Afu8g04310, A. niger CBS 513.88 : An16g08760, A. oryzae RIB40 : AO090103000048, Aspergillus wentii : Aspwe1_0118145 and Aspergillus sydowii : Aspsy1_0028793	Myosin class II heavy chain [Z]
**AN1449.3**	**ANIA_01449**	**2.21**			Predicted glycosylphosphatidylinositol (GPI)-anchored protein	
**AN1465.3**	**ANIA_01465**	**5.08**			Ortholog of A. nidulans FGSC A4 : AN8786, A. fumigatus Af293 : Afu8g01300, Afu8g04560, A. niger CBS 513.88 : An16g08420 and Aspergillus wentii : Aspwe1_0030845	
**AN1472.3**	**ANIA_01472**	**3.24**			Ortholog(s) have malate:hydrogen symporter activity, malonic acid uptake transmembrane transporter activity, succinate:hydrogen symporter activity	
**AN1492.3**	**ANIA_01492**	**2.26**			Protein of unknown function	
**AN1493.3**	**ANIA_01493**	**2.05**			Predicted glycosylphosphatidylinositol (GPI)-anchored protein	
**AN1500.3**	**ANIA_01500**	**4.89**			Has domain(s) with predicted nucleic acid binding activity	C2H2-type Zn-finger protein [K]
**AN1501.3**	**ANIA_01501**	**2.27**			Protein of unknown function	
**AN1502.3**	**ANIA_01502**	**2.26**	nagA		Extracellular N-acetyl-beta-glucosaminidase with a predicted role in chitin hydrolysis	Beta-N-acetylhexosaminidase [G]
**AN1508.3**	**ANIA_01508**	**3.22**			Ortholog of A. fumigatus Af293 : Afu8g05110, A. niger CBS 513.88 : An16g07640, Aspergillus wentii : Aspwe1_0044079, Aspergillus versicolor : Aspve1_0037294 and Aspergillus niger ATCC 1015 : 45562-mRNA	Uncharacterized conserved protein [S]
**AN1515.3**	**ANIA_01515**	**3.19**			Ortholog of A. nidulans FGSC A4 : AN2922, AN7536, A. niger CBS 513.88 : An12g10030, A. oryzae RIB40 : AO090701000305, AO090102000038, AO090124000043 and Aspergillus wentii : Aspwe1_0030521	
**AN1519.3**	**ANIA_01519**	**4.66**	rsdA		Putative Argonaute protein involved in inverted repeat transgene (IRT)-induced RNA silencing	Translation initiation factor 2C (eIF-2C) and related proteins [J]
**AN1532.3**	**ANIA_01532**	**2.35**			Ortholog of A. fumigatus Af293 : Afu8g05410, A. niger CBS 513.88 : An16g07330, A. oryzae RIB40 : AO090005000610, Aspergillus wentii : Aspwe1_0045147 and Aspergillus sydowii : Aspsy1_0041815	
**AN1536.3**	**ANIA_01536**	**5.02**		Transcription factor	Ortholog(s) have role in ascospore release from ascus, conidiophore development, conidium formation, hyphal growth, sporocarp development involved in sexual reproduction	
**AN1543.3**	**ANIA_01543**	**2.69**			Putative succinate dehydrogenase	Fumarate reductase, flavoprotein subunit [C]
**AN1548.3**	**ANIA_01548**	**3.04**			Has domain(s) with predicted nucleotide binding activity	Reductases with broad range of substrate specificities [R]
**AN1566.3**	**ANIA_01566**	**2.27**			Ortholog(s) have cytosol, nucleus localization	Predicted methyltransferase [R]
**AN1584.3**	**ANIA_01584**	**3.41**			Ortholog(s) have transcription cofactor activity, role in regulation of arginine metabolic process and nucleus localization	
**AN1588.3**	**ANIA_01588**	**2.45**			Ortholog of A. fumigatus Af293 : Afu3g13740, Neosartorya fischeri NRRL 181 : NFIA_049880, NFIA_063630, Aspergillus wentii : Aspwe1_0042965 and Aspergillus fumigatus A1163 : AFUB_035450	
**AN1590.3**	**ANIA_01590**	**2.79**			Ortholog of A. niger CBS 513.88 : An07g04490, Neosartorya fischeri NRRL 181 : NFIA_047490, Aspergillus versicolor : Aspve1_0078671 and Aspergillus niger ATCC 1015 : 131149-mRNA	
**AN1604.3**	**ANIA_01604**	**2.22**	agnE		Putative alpha-1,3-glucanase; predicted glycosyl phosphatidylinositol (GPI)-anchor	
**AN1606.3**	**ANIA_01606**	**3.48**			Has domain(s) with predicted calcium ion binding, calcium-dependent phospholipid binding activity	Annexin [U]
**AN1611.3**	**ANIA_01611**	**2.92**			Ortholog of A. nidulans FGSC A4 : AN8351, A. fumigatus Af293 : Afu4g09220, A. niger CBS 513.88 : An04g04270, A. oryzae RIB40 : AO090023000614 and Aspergillus wentii : Aspwe1_0101354	Flavin-containing monooxygenase [Q]
**AN1627.3**	**ANIA_01627**	**2.44**			Protein of unknown function	
**AN1646.3**	**ANIA_01646**	**3.36**			Ortholog of Aspergillus versicolor : Aspve1_0064838 and Aspergillus sydowii : Aspsy1_0059989	
**AN1647.3**	**ANIA_01647**	**2.63**			Protein of unknown function	
**AN1649.3**	**ANIA_01649**	**2.01**			Protein of unknown function; transcript repressed by nitrate	
**AN1656.3**	**ANIA_01656**	**2.40**			Ortholog of A. fumigatus Af293 : Afu6g03025, Neosartorya fischeri NRRL 181 : NFIA_049360, Aspergillus wentii : Aspwe1_0175985 and Aspergillus versicolor : Aspve1_0037520	
**AN1671.3**	**ANIA_01671**	**3.62**			Putative diacylglycerol pyrophosphate phosphatase with a predicted role in phospholipid metabolism	Lipid phosphate phosphatase and related enzymes of the PAP2 family [I]
**AN1673.3**	**ANIA_01673**	**2.06**			Ortholog(s) have 3-deoxy-7-phosphoheptulonate synthase activity	
**AN1674.3**	**ANIA_01674**	**2.37**			Ortholog of A. nidulans FGSC A4 : AN5649, AN0384, AN1268, AN7262, A. fumigatus Af293 : Afu1g01700, Afu2g17000, Afu4g08730, Afu4g13630, Afu1g10012 and A. niger CBS 513.88 : An01g09380, An01g06520, An04g09080, An04g03600	
**AN1677.3**	**ANIA_01677**	**3.59**			Short-chain dehydrogenase; transcriptionally induced by growth on xylose	Reductases with broad range of substrate specificities [R]
**AN1688.3**	**ANIA_01688**	**3.41**			Ortholog of S. cerevisiae : YBR053C, A. fumigatus Af293 : Afu4g08610, A. niger CBS 513.88 : An04g03420, A. oryzae RIB40 : AO090023000696 and Aspergillus wentii : Aspwe1_0034438	Ca2+-binding protein Regucalcin/SMP30 [P, T]
**AN1742.3**	**ANIA_01742**	**2.40**	mndA		Putative beta-1,4-mannosidase with a predicted role in polysaccharide degradation	Predicted beta-mannosidase [G]
**AN1748.3**	**ANIA_01748**	**2.15**	CYP666A1		Putative cytochrome P450	Cytochrome P450 CYP3/CYP5/CYP6/CYP9 subfamilies [Q]
**AN1763.3**	**ANIA_01763**	**3.25**			Has domain(s) with predicted nucleotide binding, oxidoreductase activity and role in metabolic process	Reductases with broad range of substrate specificities [R]
**AN1776.3**	**ANIA_01776**	**2.48**			Ortholog(s) have mRNA binding activity, role in mRNA splice site selection and U1 snRNP, U2-type prespliceosome, cytosol localization	Spliceosome subunit [A]
**AN1788.3**	**ANIA_01788**	**16.56**			Has domain(s) with predicted metal ion transmembrane transporter activity, role in metal ion transport, transmembrane transport and membrane localization	
**AN1799.3**	**ANIA_01799**	**2.12**			Ortholog(s) have lipase activity, role in lipid catabolic process and extracellular region localization	
**AN1809.3**	**ANIA_01809**	**9.08**			Has domain(s) with predicted fumarylacetoacetase activity and role in aromatic amino acid family metabolic process	Fumarylacetoacetase [G]

AN1810.3	ANIA_01810	2.02	otaA		Ornithine transaminase, involved in utilization of arginine as a proline source; arginine-induced expression and activity; subject to carbon-catabolite and nitrogen-metabolite repression; regulated by CreA and AreA; stress-induced protein	Ornithine aminotransferase [E]
AN1814.3	ANIA_01814	2.13			Protein of unknown function	
AN1817.3	ANIA_01817	2.00			Ortholog of A. niger CBS 513.88 : An04g08430, Neosartorya fischeri NRRL 181 : NFIA_033170, Aspergillus wentii : Aspwe1_0178646 and Aspergillus versicolor : Aspve1_0037631, Aspve1_0037735	
AN1822.3	ANIA_01822	2.21			Has domain(s) with predicted nucleotide binding, oxidoreductase activity and role in metabolic process	Reductases with broad range of substrate specificities [R]
AN1839.3	ANIA_01839	2.45			Protein with homology to GPR1/FUN34/YaaH family members	
AN1841.3	ANIA_01841	2.83			Has domain(s) with predicted nucleotide binding activity	
AN1846.3	ANIA_01846	2.43			Ortholog of A. fumigatus Af293 : Afu4g09670, A. oryzae RIB40 : AO090003001272, Aspergillus wentii : Aspwe1_0102762, Aspergillus sydowii : Aspsy1_0026263 and Aspergillus terreus NIH2624 : ATET_00776	
AN1855.3	ANIA_01855	2.53			Has domain(s) with predicted phosphoric diester hydrolase activity and role in lipid metabolic process	
AN1870.3	ANIA_01870	4.01			Putative beta-1,4-xylosidase	
AN1880.3	ANIA_01880	3.66			Ortholog of A. fumigatus Af293 : Afu2g04350, A. niger CBS 513.88 : An11g03040, A. oryzae RIB40 : AO090003000221, Aspergillus wentii : Aspwe1_0098739 and Aspergillus sydowii : Aspsy1_0126133	
AN1888.3	ANIA_01888	2.12			Putative ketose-1,6-bisphosphate aldolase	Fructose 1,6-bisphosphate aldolase [G]
AN1894.3	ANIA_01894	9.07			Transcript induced in response to calcium dichloride in a CrzA-dependent manner	
AN1897.3	ANIA_01897	2.96	hmgA		Homogentisate 1,2-dioxygenase, enzyme in phenylalanine catabolism; required for growth on phenylalanine or phenylacetate as the sole carbon source; mutation in human ortholog results in alkaptonuria	Homogentisate 1,2-dioxygenase [E]
AN1905.3	ANIA_01905	2.22	hepA		Putative HP1 chromatin modifier protein; N-terminal chromo-domain followed by a C-terminal chromo-shadow domain; S. cerevisiae does not contain a HP1 protein	
AN1920.3	ANIA_01920	3.04			Has domain(s) with predicted solute:hydrogen antiporter activity, role in cation transport, transmembrane transport and integral to membrane localization	Na+/H+ antiporter [P]
AN1924.3	ANIA_01924	3.09			Has domain(s) with predicted DNA binding, copper ion binding, sequence-specific DNA binding transcription factor activity, role in regulation of transcription, DNA-dependent and nucleus localization	
AN1926.3	ANIA_01926	2.49			Protein of unknown function	
AN1941.3	ANIA_01941	5.21			Predicted glycosylphosphatidylinositol (GPI)-anchored protein	
AN1948.3	ANIA_01948	4.32			Ortholog(s) have role in DNA methylation	
AN1952.3	ANIA_01952	4.06			Ortholog of A. fumigatus Af293 : Afu4g13380, A. niger CBS 513.88 : An01g09090, Aspergillus wentii : Aspwe1_0022581, Aspergillus sydowii : Aspsy1_0142528 and Aspergillus terreus NIH2624 : ATET_03221	
AN1958.3	ANIA_01958	3.01			Protein of unknown function	
AN1962.3	ANIA_01962	2.28			Ortholog of A. fumigatus Af293 : Afu4g10820, A. niger CBS 513.88 : An04g05820, Aspergillus wentii : Aspwe1_0145558, Aspergillus sydowii : Aspsy1_0142548 and Aspergillus terreus NIH2624 : ATET_03987	HMG-box K factor [K]
AN1972.3	ANIA_01972	2.36			Ortholog of A. nidulans FGSC A4 : AN4371, A. fumigatus Af293 : Afu4g06510, A. niger CBS 513.88 : An04g05970, An08g04550 and A. oryzae RIB40 : AO090003001143, AO090023000935	
AN1992.3	ANIA_01992	2.07		Transcription factor	Ortholog(s) have role in mRNA cis splicing, via spliceosome and U4/U6 x U5 tri-snRNP complex localization	U4/U6U5 snRNP associated protein [A]
AN2013.3	ANIA_02013	2.00			Has domain(s) with predicted nucleotide binding, oxidoreductase activity	
AN2043.3	ANIA_02043	2.31			Has domain(s) with predicted amino acid transmembrane transporter activity, role in amino acid transmembrane transport and membrane localization	Amino acid transporters [E]
AN2044.3	ANIA_02044	2.72			Ortholog of A. nidulans FGSC A4 : AN3303, AN3346, AN3348, AN8727 and A. fumigatus Af293 : Afu4g10080, Afu7g00420, Afu8g06100, Afu4g01242	
AN2045.3	ANIA_02045	2.60			Has domain(s) with predicted calcium ion binding, mannosyl-oligosaccharide 1,2-alpha-mannosidase activity and membrane localization	1, 2-alpha-mannosidase [G]
AN2064.3	ANIA_02064	2.33			Putative nonribosomal peptide synthase (NRPS)-like enzyme	Acyl-CoA synthetase [I]
AN2099.3	ANIA_02099	4.70			Putative alternative oxidase	
AN2101.3	ANIA_02101	2.78			Ortholog of A. fumigatus Af293 : Afu2g05080, A. niger CBS 513.88 : An11g04830, A. oryzae RIB40 : AO090003000311, Aspergillus wentii : Aspwe1_0083536 and Aspergillus sydowii : Aspsy1_0120628	
AN2118.3	ANIA_02118	2.42			Has domain(s) with predicted catalytic activity and role in metabolic process	Predicted metal-dependent hydrolase of the TIM-barrel fold [R]
AN2122.3	ANIA_02122	2.29		Transcription factor	Has domain(s) with predicted DNA binding, sequence-specific DNA binding RNA polymerase II transcription factor activity, zinc ion binding activity and role in regulation of transcription, DNA-dependent, transcription, DNA-templated	
AN2138.3	ANIA_02138	2.54			Ortholog(s) have cytosol localization	ATP-dependent RNA helicase A [A]
AN2144.3	ANIA_02144	4.05			Ortholog(s) have cytosol, nucleus localization	
AN2157.3	ANIA_02157	12.51	pepAa		Putative aspartic endopeptidase	Aspartyl protease [O]
AN2187.3	ANIA_02187	2.74			Protein of unknown function	
AN2228.3	ANIA_02228	3.58			Ortholog of A. fumigatus Af293 : Afu5g07200, A. niger CBS 513.88 : An17g00620, A. oryzae RIB40 : AO090701000236, Neosartorya fischeri NRRL 181 : NFIA_080060 and Aspergillus wentii : Aspwe1_0167466	
AN2232.3	ANIA_02232	2.35			Ortholog of A. fumigatus Af293 : Afu5g07250, A. niger CBS 513.88 : An17g00680, A. oryzae RIB40 : AO090701000231, Aspergillus wentii : Aspwe1_0101724 and Aspergillus sydowii : Aspsy1_0054323	Predicted seven transmembrane receptor - rhodopsin family [T]
AN2237.3	ANIA_02237	2.17			Putative carboxypeptidase C; induced by carbon starvation-induced autophagy	Serine carboxypeptidases (lysosomal cathepsin A) [E, O]
AN2251.3	ANIA_02251	4.29	BEST1		Putative bestrophin-like protein; anion channel capable of transporting a wide range of organic anions across the plasma membrane; currents activated by elevated cytosolic Ca(2+)	
AN2259.3	ANIA_02259	2.15			Ortholog of A. fumigatus Af293 : Afu5g06550, A. niger CBS 513.88 : An17g01100, A. oryzae RIB40 : AO090701000188, Aspergillus wentii : Aspwe1_0023497 and Aspergillus sydowii : Aspsy1_0054289	Predicted calmodulin-binding protein [T]
AN2277.3	ANIA_02277	2.74			Ortholog(s) have cell division site, cytosol, nucleus localization	Lysophosphatidic acid acyltransferase endophilin/SH3GL, involved in synaptic vesicle formation [I, T]
AN2300.3	ANIA_02300	2.17	atrD		Putative ATP-binding cassette (ABC) transporter of the P-glycoprotein cluster; has a role in protection against cytotoxic agents, in antibiotic secretion and in the efflux of the azole-related fungicide fenarimol; upregulated by farnesol	Multidrug/pheromone exporter, ABC superfamily [Q]
AN2325.3	ANIA_02325	3.31			Ortholog of A. nidulans FGSC A4 : AN3054, A. fumigatus Af293 : Afu5g10520, A. niger CBS 513.88 : An14g04240, An16g02910, A. oryzae RIB40 : AO090010000486 and Aspergillus wentii : Aspwe1_0131128	
AN2360.3	ANIA_02360	3.98			Has domain(s) with predicted acid phosphatase activity, metal ion binding activity	Purple acid phosphatase [G]
AN2362.3	ANIA_02362	2.03			Protein of unknown function	
AN2366.3	ANIA_02366	2.03			Putative trypsin-like protease with a role in the proteolytic cleavage of NmrA	Trypsin [E]

**AN2370.3**	**ANIA_02370**	**2.62**			Ortholog of A. nidulans FGSC A4 : AN10160, AN0323, A. fumigatus Af293 : Afu1g02440, Afu4g02760, A. niger CBS 513.88 : An01g05750, An12g09350, An13g03290 and A. oryzae RIB40 : AO090020000719, AO090005000852	Uncharacterized conserved protein [S]
**AN2373.3**	**ANIA_02373**	**3.26**	ffkC		Has domain(s) with predicted ATP binding, protein kinase activity and role in protein phosphorylation	Ankyrin [M]
**AN2377.3**	**ANIA_02377**	**2.07**			Protein of unknown function	
**AN2383.3**	**ANIA_02383**	**6.09**			Protein of unknown function	
**AN2384.3**	**ANIA_02384**	**2.35**			Has domain(s) with predicted nucleic acid binding, zinc ion binding activity	
**AN2388.3**	**ANIA_02388**	**8.37**			Putative beta-1,4-endoglucanase	
**AN2391.3**	**ANIA_02391**	**2.50**			Ortholog of A. fumigatus Af293 : Afu2g14510, A. niger CBS 513.88 : An02g00660, An07g03040, A. oryzae RIB40 : AO090023000055, AO090026000077, AO090020000644 and Aspergillus wentii : Aspwe1_0163838	
**AN2396.3**	**ANIA_02396**	**2.92**			Has domain(s) with predicted NAD binding, oxidoreductase activity, acting on the aldehyde or oxo group of donors, NAD or NADP as acceptor activity and role in cellular amino acid metabolic process, oxidation-reduction process	
**AN2397.3**	**ANIA_02397**	**3.71**			Ortholog of A. fumigatus Af293 : Afu8g06960, Aspergillus flavus NRRL 3357 : AFL2T_05473, Neosartorya fischeri NRRL 181 : NFIA_099650 and Aspergillus versicolor : Aspve1_0026050	
**AN2412.3**	**ANIA_02412**	**2.43**	cmkA		Calcium/calmodulin-dependent protein kinase A; essential for growth and nuclear division; expression downregulated by farnesol; transcript upregulated by camptothecin; mutants have a moderate growth defect and increased pigment production	Ca2+/calmodulin-dependent protein kinase, EF-Hand protein superfamily [T]
**AN2417.3**	**ANIA_02417**	**2.04**			Ortholog(s) have role in chromatin remodeling, positive regulation of protein phosphorylation, signal transduction involved in mitotic DNA replication checkpoint and ASTRA complex, TORC1 complex, TORC2 complex, cytosol localization	Uncharacterized conserved protein [S]
**AN2436.3**	**ANIA_02436**	**2.19**	aclB		Putative ATP citrate synthase with a predicted role in TCA intermediate metabolism; transcript downregulated upon shift from glucose to ethanol and after exposure to farnesol	ATP-citrate lyase [C]
**AN2491.3**	**ANIA_02491**	**4.71**			Ortholog(s) have DNA helicase activity, DNA replication origin binding, chromatin binding, single-stranded DNA binding activity	DNA replication licensing factor, MCM2 component [L]
**AN2498.3**	**ANIA_02498**	**2.06**			Has domain(s) with predicted zinc ion binding activity and intracellular localization	
**AN2499.3**	**ANIA_02499**	**2.83**			Ortholog(s) have role in cellular response to cadmium ion, detoxification of cadmium ion and cytosol, nucleus localization	RNA polymerase II elongator associated protein [R]
**AN2507.3**	**ANIA_02507**	**2.90**			Has domain(s) with predicted ubiquitin thiolesterase activity and role in ubiquitin-dependent protein catabolic process	
**AN2518.3**	**ANIA_02518**	**3.27**			Ortholog(s) have cytosol localization	Vesicle trafficking protein Sly1 (Sec1 family) [U]
**AN2538.3**	**ANIA_02538**	**2.67**			Ortholog of Aspergillus versicolor : Aspve1_0026536 and Aspergillus sydowii : Aspsy1_0028980	
**AN2548.3**	**ANIA_02548**	**2.17**	easC		Putative acyltransferase; required for emericellamide biosynthesis; emericellamide (eas) biosynthetic gene cluster member	
**AN2549.3**	**ANIA_02549**	**4.18**	easD		Putative acyl-CoA ligase; required for emericellamide biosynthesis; emericellamide (eas) biosynthetic gene cluster member	Acyl-CoA synthetase [I]
**AN2561.3**	**ANIA_02561**	**5.66**			Putative methyltransferase; intracellular, menadione stress-induced protein	
**AN2562.3**	**ANIA_02562**	**2.10**			Ortholog of A. nidulans FGSC A4 : AN2699, AN10332, A. fumigatus Af293 : Afu3g15000, A. niger CBS 513.88 : An09g01110, An09g02880 and A. oryzae RIB40 : AO090010000620, AO090012000340	Uncharacterized conserved protein [S]
**AN2572.3**	**ANIA_02572**	**4.73**			Putative dipeptidyl-peptidase; transcript upregulated by nitrate limitation	Dipeptidyl aminopeptidase [O]
**AN2573.3**	**ANIA_02573**	**10.46**			Has domain(s) with predicted iron ion binding, oxidoreductase activity and role in fatty acid biosynthetic process, oxidation-reduction process	C-4 sterol methyl oxidase [I]
**AN2596.3**	**ANIA_02596**	**3.77**	CYP646A1		Putative cytochrome P450	Cytochrome P450 [Q]
**AN2602.3**	**ANIA_02602**	**2.92**			Putative lipase/esterase domain family protein; transcript is induced by nitrate	Arylacetamide deacetylase [V]
**AN2618.3**	**ANIA_02618**	**2.11**			Protein of unknown function	
**AN2622.3**	**ANIA_02622**	**2.46**	ipnA		Isopenicillin-N synthase with a role in penicillin biosynthesis; expression is negatively regulated by glucose and acidic pH	Iron/ascorbate family oxidoreductases [Q, R]
**AN2629.3**	**ANIA_02629**	**3.77**			Ortholog of A. nidulans FGSC A4 : AN8660, A. fumigatus Af293 : Afu6g01900, A. niger CBS 513.88 : An12g06490 and A. oryzae RIB40 : AO090038000052, AO090001000087, AO090009000516	Flavin-containing monooxygenase [Q]
**AN2636.3**	**ANIA_02636**	**2.32**			Putative F-box protein	
**AN2659.3**	**ANIA_02659**	**2.32**			Predicted glycosylphosphatidylinositol (GPI)-anchored protein	
**AN2669.3**	**ANIA_02669**	**2.96**			Has domain(s) with predicted role in response to stress and integral to membrane localization	
**AN2700.3**	**ANIA_02700**	**2.07**			Ortholog of A. nidulans FGSC A4 : AN7942, AN7872, AN5510, AN3420, AN9050, AN8973, AN11222, AN10911, AN1749 and A. fumigatus Af293 : Afu1g12450, Afu2g00880, Afu2g17800, Afu2g17880, Afu5g14720, Afu7g06590	
**AN2701.3**	**ANIA_02701**	**8.10**			Ortholog(s) have role in conidiophore development, hyphal growth, sporocarp development involved in sexual reproduction, syncytium formation by plasma membrane fusion	
**AN2702.3**	**ANIA_02702**	**3.30**			Protein of unknown function	
**AN2746.3**	**ANIA_02746**	**2.02**			Has domain(s) with predicted role in transmembrane transport and integral to membrane localization	Monocarboxylate transporter [G]
**AN2760.3**	**ANIA_02760**	**5.09**			Ortholog of A. fumigatus Af293 : Afu3g06020, A. niger CBS 513.88 : An11g10520, A. oryzae RIB40 : AO090020000072, Aspergillus wentii : Aspwe1_0052504 and Aspergillus sydowii : Aspsy1_0056680	
**AN2768.3**	**ANIA_02768**	**2.25**			Has domain(s) with predicted role in mitotic spindle organization in nucleus and DASH complex, spindle microtubule localization	
**AN2783.3**	**ANIA_02783**	**2.34**			Has domain(s) with predicted role in transmembrane transport and integral to membrane localization	Uncharacterized conserved protein [S]
**AN2792.3**	**ANIA_02792**	**2.89**			Ortholog of A. fumigatus Af293 : Afu8g02480, A. niger CBS 513.88 : An12g05730, Neosartorya fischeri NRRL 181 : NFIA_096170 and Aspergillus versicolor : Aspve1_0038333	
**AN2818.3**	**ANIA_02818**	**4.30**			Ortholog of A. niger CBS 513.88 : An12g03370, An18g00460, An07g00580, A. oryzae RIB40 : AO090103000478, Aspergillus wentii : Aspwe1_0058229, Aspwe1_0111514 and Aspergillus versicolor : Aspve1_0051884	
**AN2826.3**	**ANIA_02826**	**2.35**		Transcription factor	Transcript induced in response to calcium dichloride in a CrzA-dependent manner	
**AN2828.3**	**ANIA_02828**	**2.42**	bglL		Putative beta-glucosidase with a predicted role in polysaccharide degradation	
**AN2830.3**	**ANIA_02830**	**2.86**			Has domain(s) with predicted iron ion binding, oxidoreductase activity and role in fatty acid biosynthetic process, oxidation-reduction process	C-4 sterol methyl oxidase [I]
**AN2845.3**	**ANIA_02845**	**2.09**			Ortholog of A. fumigatus Af293 : Afu3g12260, A. oryzae RIB40 : AO090003000791, Aspergillus wentii : Aspwe1_0169211 and Aspergillus sydowii : Aspsy1_0087513	
**AN2862.3**	**ANIA_02862**	**2.17**			Predicted CAP-Gly domain protein with similarity to Saccharomyces cerevisiae Bim1p	Microtubule-binding protein involved in cell cycle control [D, Z]
**AN2876.3**	**ANIA_02876**	**2.85**			Ortholog of A. nidulans FGSC A4 : AN5876, A. fumigatus Af293 : Afu2g11370, Afu3g11770, Aspergillus wentii : Aspwe1_0653753 and Aspergillus sydowii : Aspsy1_0146596, Aspsy1_1176317	

**AN2895.3**	**ANIA_02895**	**5.34**			Has domain(s) with predicted ADP binding activity	Uncharacterized conserved protein [S]
**AN2899.3**	**ANIA_02899**	**2.54**			Ortholog of A. fumigatus Af293 : Afu3g11450, A. niger CBS 513.88 : An02g07290, A. oryzae RIB40 : AO090003000699, Aspergillus wentii : Aspwe1_0150280 and Aspergillus sydowii : Aspsy1_0056343	
**AN2913.3**	**ANIA_02913**	**2.16**			Ortholog of A. nidulans FGSC A4 : AN4375, A. fumigatus Af293 : Afu3g07740, Afu4g06610, A. niger CBS 513.88 : An04g00930 and A. oryzae RIB40 : AO090023000927, AO090020000420	
**AN2919.3**	**ANIA_02919**	**3.28**	sltA	Transcription factor	C2H2 zinc-finger transcription factor involved in cation homeostasis; involved in response to abiotic stress; transcript upregulated in response to camptothecin; necessary for growth on pH 8 medium	
**AN2921.3**	**ANIA_02921**	**4.15**			Has domain(s) with predicted nucleotide binding activity	
**AN2937.3**	**ANIA_02937**	**2.43**			Ortholog of A. fumigatus Af293 : Afu3g08210, A. niger CBS 513.88 : An02g11760, Aspergillus wentii : Aspwe1_0029179 and Aspergillus sydowii : Aspsy1_0042633	
**AN2941.3**	**ANIA_02941**	**3.01**			Ortholog of A. nidulans FGSC A4 : AN5437, A. fumigatus Af293 : Afu3g08250, Afu6g13630, A. niger CBS 513.88 : An02g11860 and A. oryzae RIB40 : AO090005001453, AO090103000325	
**AN2943.3**	**ANIA_02943**	**2.18**	rfeA		Putative regulator of secondary metabolism	Checkpoint kinase and related serine/threonine protein kinases [D]
**AN2947.3**	**ANIA_02947**	**2.76**			Putative 1-phosphatidylinositol-4,5-bisphosphate phosphodiesterase with a predicted role in phospholipid metabolism	Phosphoinositide-specific phospholipase C [T]
**AN2953.3**	**ANIA_02953**	**3.16**			Ortholog of A. fumigatus Af293 : Afu3g07890, A. niger CBS 513.88 : An02g11360, A. oryzae RIB40 : AO090005001486, Aspergillus wentii : Aspwe1_0041944 and Aspergillus sydowii : Aspsy1_0056308	
**AN2971.3**	**ANIA_02971**	**2.14**			Ortholog of A. fumigatus Af293 : Afu3g08320, A. oryzae RIB40 : AO090005001443, Aspergillus wentii : Aspwe1_0552305, Aspergillus sydowii : Aspsy1_0130422 and Aspergillus terreus NIH2624 : ATET_01636	
**AN2979.3**	**ANIA_02979**	**4.78**			Ortholog of A. fumigatus Af293 : Afu3g08450, A. niger CBS 513.88 : An02g12090, A. oryzae RIB40 : AO090005001429, Aspergillus wentii : Aspwe1_0551961 and Aspergillus sydowii : Aspsy1_0056256	
**AN2983.3**	**ANIA_02983**	**3.42**	msb1		Putative Rho GTPase-activating protein (Rho-GAP); null mutants form normal septa	
**AN2993.3**	**ANIA_02993**	**2.71**			Ortholog of A. fumigatus Af293 : Afu3g08610, A. niger CBS 513.88 : An02g12330, A. oryzae RIB40 : AO090005001411, Aspergillus wentii : Aspwe1_0550058 and Aspergillus sydowii : Aspsy1_0042547	Vesicle coat complex COPII, subunit SFB3 [U]
**AN2994.3**	**ANIA_02994**	**3.08**			Ortholog of A. niger CBS 513.88 : An02g12350, Aspergillus tubingensis : Asptu1_0118593, Aspergillus glaucus : Aspgl1_0925168 and Aspergillus flavus NRRL 3357 : AFL2T_01318	Ankyrin repeat and DHHC-type Zn-finger domain containing proteins [R]
**AN3021.3**	**ANIA_03021**	**5.36**			Ortholog of A. fumigatus Af293 : Afu3g08920, A. niger CBS 513.88 : An16g02420, A. oryzae RIB40 : AO090005001345, Aspergillus wentii : Aspwe1_0052288 and Aspergillus sydowii : Aspsy1_0147269	
**AN3037.3**	**ANIA_03037**	**2.13**			Has domain(s) with predicted cholinesterase activity	Acetylcholinesterase/Butyrylcholinesterase [T]
**AN3048.3**	**ANIA_03048**	**2.01**		Transcription factor	Predicted mariner transposon-related ORF	
**AN3062.3**	**ANIA_03062**	**3.19**	pcpA		Putative pericentrin-related protein involved in microtubule organization and hyphal polarity	Myosin class II heavy chain [Z]
**AN3085.3**	**ANIA_03085**	**2.34**			Ortholog(s) have role in cellular response to biotic stimulus, cellular response to starvation and filamentous growth of a population of unicellular organisms in response to biotic stimulus, more	Predicted phosphatase [R]
**AN3124.3**	**ANIA_03124**	**2.21**			Kinesin-family protein	Kinesin-like protein [Z]
**AN3132.3**	**ANIA_03132**	**3.40**			Ortholog(s) have role in N-acylethanolamine metabolic process, N-acylphosphatidylethanolamine metabolic process and integral to mitochondrial inner membrane localization	Predicted Zn-dependent hydrolase (beta-lactamase superfamily) [R]
**AN3141.3**	**ANIA_03141**	**11.02**			Predicted glycosylphosphatidylinositol (GPI)-anchored protein	
**AN3162.3**	**ANIA_03162**	**3.34**	ngn4		Putative GNAT-type acetyltransferase	
**AN3165.3**	**ANIA_03165**	**2.23**			Ortholog of A. fumigatus Af293 : Afu3g13420, A. niger CBS 513.88 : An02g09290, A. oryzae RIB40 : AO090012000789, Aspergillus wentii : Aspwe1_0170053 and Aspergillus sydowii : Aspsy1_0087149	
**AN3188.3**	**ANIA_03188**	**3.52**			Putative phosphatidylserine decarboxylase with a predicted role in phospholipid metabolism	Phosphatidylserine decarboxylase [I]
**AN3191.3**	**ANIA_03191**	**2.66**			Ortholog(s) have lipase activity	Arylacetamide deacetylase [V]
**AN3225.3**	**ANIA_03225**	**4.84**	CYP631B1		Putative cytochrome P450; aspernidine A secondary metabolism gene cluster member	Cytochrome P450 CYP4/CYP19/CYP26 subfamilies [Q, I]
**AN3226.3**	**ANIA_03226**	**7.71**	pkfC		Has domain protein; aspernidine A secondary metabolism gene cluster member; protein levels decrease in response to farnesol	Dehydrogenases with different specificities (related to short-chain alcohol dehydrogenases) [Q]
**AN3228.3**	**ANIA_03228**	**2.37**	pkfE		Predicted prenyltransferase; aspernidine A secondary metabolism gene cluster member	Para-hydroxybenzoate-polyprenyl transferase [H]
**AN3234.3**	**ANIA_03234**	**4.80**			Has domain(s) with predicted nucleotide binding, oxidoreductase activity and role in metabolic process	Predicted short chain-type dehydrogenase [R]
**AN3241.3**	**ANIA_03241**	**2.35**			Ortholog of A. nidulans FGSC A4 : AN6413, AN6419, AN6946, AN5312, AN5664, AN8328, AN10369, AN1930, AN2386, AN1738, AN11159, AN8984, AN8951, AN1540, AN8971, AN9387, AN9266, AN9306, AN1317, AN10886, AN7232, AN7774, AN12202	
**AN3256.3**	**ANIA_03256**	**6.53**	CYP648A1		Putative cytochrome P450; predicted secondary metabolism gene cluster member	Cytochrome P450 CYP4/CYP19/CYP26 subfamilies [Q, I]
**AN3259.3**	**ANIA_03259**	**2.59**			Ortholog of A. oryzae RIB40 : AO090011000035, Aspergillus flavus NRRL 3357 : AFL2T_04841, Aspergillus versicolor : Aspve1_0040331 and Aspergillus sydowii : Aspsy1_0029969	
**AN3262.3**	**ANIA_03262**	**6.51**			Ortholog of Aspergillus glaucus : Aspgl1_0062845, Aspgl1_0155124, Aspergillus versicolor : Aspve1_0149729 and Aspergillus sydowii : Aspsy1_0067279	
**AN3265.3**	**ANIA_03265**	**12.57**	apyA		Arrestin domains and PY motif-containing protein with homology to Saccharomyces cerevisiae Rod1p and Rog3p proteins	Thioredoxin binding protein TBP-2/VDUP1 [R]
**AN3287.3**	**ANIA_03287**	**2.39**			Has domain(s) with predicted amino acid transmembrane transporter activity, role in amino acid transmembrane transport and membrane localization	Amino acid transporters [E]
**AN3298.3**	**ANIA_03298**	**2.65**			Has domain(s) with predicted protein disulfide oxidoreductase activity	
**AN3303.3**	**ANIA_03303**	**4.47**			Ortholog of A. nidulans FGSC A4 : AN3346, AN3348, AN2044, AN8727 and A. fumigatus Af293 : Afu4g10080, Afu7g00420, Afu8g06100, Afu4g01242	
**AN3304.3**	**ANIA_03304**	**5.23**			Putative GABA transporter; transcript repressed by light in developmentally competent mycelia	Amino acid transporters [E]
**AN3333.3**	**ANIA_03333**	**4.23**			Has domain(s) with predicted nucleotide binding, oxidoreductase activity	Predicted oxidoreductase [R]
**AN3342.3**	**ANIA_03342**	**2.16**			Ortholog of A. nidulans FGSC A4 : AN10302, AN4609, A. fumigatus Af293 : Afu7g06670, A. niger CBS 513.88 : An04g04320, An16g05670, An04g03250 and A. oryzae RIB40 : AO090011000012	
**AN3345.3**	**ANIA_03345**	**2.60**			Has domain(s) with predicted amino acid transmembrane transporter activity, role in amino acid transmembrane transport and membrane localization	Amino acid transporters [E]
**AN3346.3**	**ANIA_03346**	**2.21**			Ortholog of A. nidulans FGSC A4 : AN3303, AN3348, AN2044, AN8727 and A. fumigatus Af293 : Afu4g10080, Afu7g00420, Afu8g06100, Afu4g01242	
**AN3348.3**	**ANIA_03348**	**3.44**			Ortholog of A. nidulans FGSC A4 : AN3303, AN3346, AN2044, AN8727 and A. fumigatus Af293 : Afu4g10080, Afu7g00420, Afu8g06100, Afu4g01242	

**AN3351.3**	**ANIA_03351**	**2.33**			Has domain(s) with predicted UDP-N-acetylmuramate dehydrogenase activity, flavin adenine dinucleotide binding activity and role in oxidation-reduction process	Proteins containing the FAD binding domain [C]
**AN3356.3**	**ANIA_03356**	**4.41**		Transcription factor	Has domain(s) with predicted DNA binding, sequence-specific DNA binding RNA polymerase II transcription factor activity, zinc ion binding activity and role in regulation of transcription, DNA-dependent, transcription, DNA-templated	
**AN3377.3**	**ANIA_03377**	**2.18**			Ortholog(s) have endopeptidase activity, role in proteolysis, zymogen activation and extracellular region localization	
**AN3379.3**	**ANIA_03379**	**4.08**			Ortholog of A. fumigatus Af293 : Afu7g01170, A. niger CBS 513.88 : An01g01580, An12g01970, An01g00420, A. oryzae RIB40 : AO090010000104, AO090038000140 and Aspergillus wentii : Aspwe1_0112566	
**AN3421.3**	**ANIA_03421**	**2.76**			Has domain(s) with predicted nucleotide binding activity	
**AN3438.3**	**ANIA_03438**	**5.16**			Has domain(s) with predicted ARF guanyl-nucleotide exchange factor activity, phospholipid binding activity, role in regulation of ARF protein signal transduction and intracellular localization	Guanine nucleotide exchange factor [U]
**AN3472.3**	**ANIA_03472**	**2.46**			Protein of unknown function	Multidrug resistance-associated protein/mitoxantrone resistance protein, ABC superfamily [Q]
**AN3488.3**	**ANIA_03488**	**2.04**			Has domain(s) with predicted nucleotide binding activity	Flavin-containing monooxygenase [Q]
**AN3490.3**	**ANIA_03490**	**2.48**			Predicted acetyl CoA synthase; inp secondary metabolite gene cluster member	Acyl-CoA synthetase [I]
**AN3493.3**	**ANIA_03493**	**2.29**			Predicted threonine-type endopeptidase; inp secondary metabolite gene cluster member	20S proteasome, regulatory subunit beta type PSMB1/PRE7 [O]
**AN3497.3**	**ANIA_03497**	**3.17**	CYP623B2		Putative cytochrome P450	Cytochrome P450 CYP4/CYP19/CYP26 subfamilies [Q, I]
**AN3546.3**	**ANIA_03546**	**2.03**			Has domain(s) with predicted DNA binding, zinc ion binding activity, role in DNA integration, DNA recombination and intracellular localization	
**AN3551.3**	**ANIA_03551**	**2.45**			Protein of unknown function	CAATT-binding K factor/60S ribosomal subunit biogenesis protein [J, K]
**AN3552.3**	**ANIA_03552**	**7.45**			Protein of unknown function	
**AN3554.3**	**ANIA_03554**	**4.25**			Protein of unknown function	
**AN3557.3**	**ANIA_03557**	**2.80**			Ortholog of Aspergillus versicolor : Aspve1_0145302 and Aspergillus aculeatus ATCC16872 : Aacu16872_053773	
**AN3563.3**	**ANIA_03563**	**3.12**			Putative oxalate decarboxylase with a predicted role in oxalic acid metabolism	
**AN3574.3**	**ANIA_03574**	**2.38**			Protein of unknown function	
**AN3581.3**	**ANIA_03581**	**3.23**	trxR		Thioredoxin reductase with a predicted role in pyrimidine metabolism; putative flavoprotein; intracellular, menadione stress-induced protein; transcripts of two different sizes have been detected	Thioredoxin reductase [O]
**AN3585.3**	**ANIA_03585**	**4.38**			Transcript induced in response to calcium dichloride in a CrzA-dependent manner	
**AN3590.3**	**ANIA_03590**	**2.45**			Ortholog of A. nidulans FGSC A4 : AN7906, A. fumigatus Af293 : Afu4g12880, A. niger CBS 513.88 : An01g08440, A. oryzae RIB40 : AO090009000315 and Aspergillus wentii : Aspwe1_0033843	
**AN3592.3**	**ANIA_03592**	**2.90**	clxA		Putative calnexin with a predicted role in protein folding and protein quality control on the endoplasmic reticulum (ER) membrane	Calnexin [O]
**AN3615.3**	**ANIA_03615**	**2.34**			Has domain(s) with predicted ATP binding, nucleoside-triphosphatase activity	AAA+-type ATPase [O]
**AN3648.3**	**ANIA_03648**	**2.74**	nimE		B-type cyclin, mitosis-specific cyclin required for G2-M transition; regulates activity of cyclin-dependent protein kinase NimX	Cyclin B and related kinase-activating proteins [D]
**AN3664.3**	**ANIA_03664**	**2.45**			Ortholog(s) have cell surface localization	Endocytic adaptor protein intersectin [T, U]
**AN3670.3**	**ANIA_03670**	**2.27**			Ortholog of A. fumigatus Af293 : Afu4g12380, A. niger CBS 513.88 : An01g07790, A. oryzae RIB40 : AO090009000446, Aspergillus wentii : Aspwe1_0166560 and Aspergillus sydowii : Aspsy1_0032623	
**AN3674.3**	**ANIA_03674**	**4.26**			Has domain(s) with predicted phospholipid binding activity	
**AN3733.3**	**ANIA_03733**	**2.06**	mns1A		Putative alpha-1,2 mannosidase with a predicted role in mannose polymer metabolism	1, 2-alpha-mannosidase [G]
**AN3743.3**	**ANIA_03743**	**2.87**			Ortholog of A. nidulans FGSC A4 : AN5603, A. fumigatus Af293 : Afu4g11320, Afu7g04400, A. niger CBS 513.88 : An13g00980, An04g05280 and A. oryzae RIB40 : AO090005000127	Vesicle coat complex COPII, subunit SFB3 [U]
**AN3752.3**	**ANIA_03752**	**3.39**			Has domain(s) with predicted Rab GTPase activator activity, role in regulation of Rab GTPase activity and intracellular localization	Uncharacterized conserved protein, contains TBC domain [T, R]
**AN3753.3**	**ANIA_03753**	**2.20**			Ortholog(s) have RNA binding, telomeric template RNA reverse transcriptase activity, role in telomere maintenance via telomerase and telomerase catalytic core complex localization	Telomerase catalytic subunit/reverse transcriptase TERT [L, B]
**AN3796.3**	**ANIA_03796**	**3.76**			Ortholog of Neosartorya fischeri NRRL 181 : NFIA_080720, Aspergillus wentii : Aspwe1_0110971, Aspergillus versicolor : Aspve1_0083942 and Aspergillus clavatus NRRL 1 : ACLA_090600	
**AN3798.3**	**ANIA_03798**	**2.38**			Has domain(s) with predicted heme binding activity	Flavohemoprotein b5+b5R [C]
**AN3804.3**	**ANIA_03804**	**2.66**			Ortholog(s) have IgE binding activity	
**AN3821.3**	**ANIA_03821**	**3.97**			Ortholog of A. fumigatus Af293 : Afu2g03650, A. niger CBS 513.88 : An07g08220, A. oryzae RIB40 : AO090120000220, Aspergillus wentii : Aspwe1_0172303 and Aspergillus sydowii : Aspsy1_0030705	Equilibrative nucleoside transporter protein [F]
**AN3856.3**	**ANIA_03856**	**2.25**			Protein of unknown function	
**AN3866.3**	**ANIA_03866**	**2.72**			Putative dehydratase with a predicted role in glycine, serine, and threonine metabolism	Threonine/serine dehydratases [E]
**AN3883.3**	**ANIA_03883**	**2.36**			Putative glucanase with a predicted role beta-glucan hydrolysis	
**AN3889.3**	**ANIA_03889**	**2.41**			Has domain(s) with predicted nucleotide binding, oxidoreductase activity and role in metabolic process	Mitochondrial/plastidial beta-ketoacyl-ACP reductase [I]
**AN3895.3**	**ANIA_03895**	**2.13**			Has domain(s) with predicted catalytic activity and role in metabolic process	Predicted L-carnitine dehydratase/alpha-methylacyl-CoA racemase [I]
**AN3941.3**	**ANIA_03941**	**2.62**	nimT		Protein tyrosine phosphatase involved in cell cycle control; required for progression through G2; homolog of S. pombe cdc25	M-phase inducer phosphatase [D]
**AN3959.3**	**ANIA_03959**	**4.72**			Has domain(s) with predicted metalloendopeptidase activity and role in proteolysis	
**AN3961.3**	**ANIA_03961**	**4.01**			Ortholog of Aspergillus versicolor : Aspve1_0054191	Putative cytochrome C oxidase assembly protein [C]
**AN3983.3**	**ANIA_03983**	**3.61**			Ortholog of Aspergillus wentii : Aspwe1_0168245, Aspergillus clavatus NRRL 1 : ACLA_062140 and Aspergillus aculeatus ATCC16872 : Aacu16872_043723	
**AN3996.3**	**ANIA_03996**	**2.65**			Has domain(s) with predicted methyltransferase activity and role in metabolic process	Predicted protein carboxyl methylase [R]
**AN4008.3**	**ANIA_04008**	**3.16**			Has domain(s) with predicted O-methyltransferase activity	Hydroxyindole-O-methyltransferase and related SAM-dependent methyltransferases [R]
**AN4019.3**	**ANIA_04019**	**3.70**			Has domain(s) with predicted role in transmembrane transport and integral to membrane localization	Permease of the major facilitator superfamily [R]
**AN4042.3**	**ANIA_04042**	**2.18**	CYP61A1		Putative C-22 sterol desaturase with a predicted role in sterol metabolism; putative cytochrome P450	Cytochrome P450 CYP4/CYP19/CYP26 subfamilies [Q, I]
**AN4063.3**	**ANIA_04063**	**2.55**			Ortholog of A. fumigatus Af293 : Afu1g05380, Neosartorya fischeri NRRL 181 : NFIA_019370, Aspergillus wentii : Aspwe1_0037253 and Aspergillus versicolor : Aspve1_0054332	
**AN4068.3**	**ANIA_04068**	**2.27**			Ortholog(s) have UDP-galactose transmembrane transporter activity, role in UDP-galactose transmembrane transport, UDP-glucose transport, regulation of protein folding in endoplasmic reticulum and endoplasmic reticulum localization	UDP-galactose transporter related protein [G]
**AN4077.3**	**ANIA_04077**	**3.18**			Has domain(s) with predicted DNA binding activity	
**AN4078.3**	**ANIA_04078**	**3.72**			Protein of unknown function	
**AN4081.3**	**ANIA_04081**	**2.12**			Ortholog(s) have role in L-cysteine catabolic process to pyruvate, using cysteine dioxygenase	Cysteine dioxygenase CDO1 [E]
**AN4102.3**	**ANIA_04102**	**2.05**	bglA		Putative beta-glucosidase; induced by carbon starvation-induced autophagy	

**AN4110.3**	**ANIA_04110**	**2.18**			Has domain(s) with predicted N,N-dimethylaniline monooxygenase activity, NADP binding, flavin adenine dinucleotide binding activity and role in oxidation-reduction process	Flavin-containing monooxygenase [Q]
**AN4114.3**	**ANIA_04114**	**2.37**			Has domain(s) with predicted nucleotide binding, oxidoreductase activity and role in oxidation-reduction process	Aldo/keto reductase family proteins [R]
**AN4126.3**	**ANIA_04126**	**3.32**			Putative dehydrogenase with a predicted role in metabolism or penicillin biosynthesis	Aldehyde dehydrogenase [C]
**AN4148.3**	**ANIA_04148**	**2.03**			Sugar transporter; transcriptionally induced by growth on xylose	Predicted transporter (major facilitator superfamily) [R]
**AN4170.3**	**ANIA_04170**	**2.23**	creD		Arrestin domains and PY motif-containing protein with homology to Saccharomyces cerevisiae Rod1p and Rog3p proteins; expression reduced after exposure to farnesol	Thioredoxin binding protein TBP-2/VDUP1 [R]
**AN4172.3**	**ANIA_04172**	**2.12**			Ortholog of A. fumigatus Af293 : Afu6g07930, A. niger CBS 513.88 : An11g02850, A. oryzae RIB40 : AO090003000141, Aspergillus wentii : Aspwe1_0748330 and Aspergillus sydowii : Aspsy1_0059114	
**AN4181.3**	**ANIA_04181**	**2.50**			Ortholog of Aspergillus versicolor : Aspve1_0055365 and Aspergillus sydowii : Aspsy1_0205288	
**AN4190.3**	**ANIA_04190**	**3.52**			Predicted glycosylphosphatidylinositol (GPI)-anchored protein; palA-dependent expression independent of pH	
**AN4195.3**	**ANIA_04195**	**4.06**			Has domain(s) with predicted carbon-nitrogen ligase activity, with glutamine as amido-N-donor, hydrolase activity, acting on carbon-nitrogen (but not peptide) bonds, in linear amides activity	Amidases [J, I, T]
**AN4196.3**	**ANIA_04196**	**2.19**	ffkF		Has domain(s) with predicted ATP binding, protein tyrosine kinase activity and role in protein phosphorylation	Ribosomal protein S6 kinase and related proteins [R, T]
**AN4197.3**	**ANIA_04197**	**2.87**			Ortholog of Aspergillus versicolor : Aspve1_0138462 and Aspergillus sydowii : Aspsy1_0154012	
**AN4206.3**	**ANIA_04206**	**4.33**			Has domain(s) with predicted heat shock protein binding, unfolded protein binding activity and role in protein folding	Molecular chaperone (DnaJ superfamily) [O]
**AN4241.3**	**ANIA_04241**	**2.05**			Has domain(s) with predicted role in transmembrane transport and integral to membrane localization	Synaptic vesicle transporter SVOP and related transporters (major facilitator superfamily) [R]
**AN4246.3**	**ANIA_04246**	**3.43**			Ortholog of A. nidulans FGSC A4 : AN8484, AN4438, A. fumigatus Af293 : Afu1g06480, Afu2g05150/mp2, A. niger CBS 513.88 : An18g05510 and A. oryzae RIB40 : AO090001000450	
**AN4252.3**	**ANIA_04252**	**2.69**			Ortholog of A. nidulans FGSC A4 : AN10165, A. fumigatus Af293 : Afu1g06580/hel10, Afu1g11910, A. niger CBS 513.88 : An18g05640, An08g04120 and A. oryzae RIB40 : AO090001000456, AO090001000373	
**AN4255.3**	**ANIA_04255**	**2.15**	hxkC		Non-catalytic atypical hexokinase involved in regulation of extracellular proteases in response to carbon starvation; associates with mitochondria	Hexokinase [G]
**AN4264.3**	**ANIA_04264**	**6.14**			Ortholog(s) have role in syncytium formation by plasma membrane fusion	
**AN4265.3**	**ANIA_04265**	**2.77**			Ortholog(s) have role in DNA repair, mitotic chromosome condensation and condensin complex, cytosol, nucleus localization	Chromosome condensation complex Condensin, subunit H [B, D]
**AN4268.3**	**ANIA_04268**	**2.71**			Ortholog(s) have nitronate monooxygenase activity and role in denitrification pathway, detoxification of nitrogen compound	
**AN4273.3**	**ANIA_04273**	**2.07**			Ortholog of A. fumigatus Af293 : Afu7g03800, A. niger CBS 513.88 : An13g00220, A. oryzae RIB40 : AO090026000834, Aspergillus wentii : Aspwe1_0026252 and Aspergillus sydowii : Aspsy1_1088954	Predicted Ca2+-dependent phospholipid-binding protein [R]
**AN4302.3**	**ANIA_04302**	**2.78**			Ortholog of A. fumigatus Af293 : Afu4g05930, A. niger CBS 513.88 : An03g02750, Aspergillus wentii : Aspwe1_0099945, Aspergillus sydowii : Aspsy1_0036582 and Aspergillus terreus NIH2624 : ATET_06827	
**AN4312.3**	**ANIA_04312**	**2.58**			Ortholog of A. fumigatus Af293 : Afu4g06040, A. niger CBS 513.88 : An04g00270, A. oryzae RIB40 : AO090023000991, Aspergillus wentii : Aspwe1_0101375 and Aspergillus sydowii : Aspsy1_0036593	
**AN4314.3**	**ANIA_04314**	**2.91**			Ortholog of A. fumigatus Af293 : Afu4g06060, A. niger CBS 513.88 : An04g00300, A. oryzae RIB40 : AO090023000990, Aspergillus wentii : Aspwe1_0034783 and Aspergillus sydowii : Aspsy1_0073108	
**AN4339.3**	**ANIA_04339**	**3.10**			Protein of unknown function	Kinesin light chain [Z]
**AN4345.3**	**ANIA_04345**	**3.15**			Has domain(s) with predicted catalytic activity, magnesium ion binding, thiamine pyrophosphate binding activity	Thiamine pyrophosphate-requiring enzyme [E, H]
**AN4348.3**	**ANIA_04348**	**2.83**			Ortholog of A. fumigatus Af293 : Afu7g00760, Neosartorya fischeri NRRL 181 : NFIA_113420, Aspergillus wentii : Aspwe1_0044898, Aspwe1_0655650 and Aspergillus versicolor : Aspve1_0085199	Predicted haloacid-halidohydrolase and related hydrolases [R]
**AN4354.3**	**ANIA_04354**	**2.17**			Protein of unknown function	
**AN4357.3**	**ANIA_04357**	**2.77**			Has domain(s) with predicted exonuclease activity, nucleic acid binding activity and intracellular localization	3'-5' exonuclease [L]
**AN4363.3**	**ANIA_04363**	**2.27**			Has domain(s) with predicted UDP-N-acetylmuramate dehydrogenase activity, flavin adenine dinucleotide binding activity and role in oxidation-reduction process	Proteins containing the FAD binding domain [C]
**AN4375.3**	**ANIA_04375**	**2.37**			Ortholog of A. nidulans FGSC A4 : AN2913, A. fumigatus Af293 : Afu3g07740, Afu4g06610, A. niger CBS 513.88 : An04g00930 and A. oryzae RIB40 : AO090023000927, AO090020000420	
**AN4379.3**	**ANIA_04379**	**3.73**			Ortholog of A. fumigatus Af293 : Afu4g06670/aspf7, A. oryzae RIB40 : AO090023000918, Neosartorya fischeri NRRL 181 : NFIA_109540 and Aspergillus wentii : Aspwe1_0023301	Neurexin III-alpha [T]
**AN4394.3**	**ANIA_04394**	**3.85**			Ortholog(s) have role in asexual sporulation resulting in formation of a cellular spore, positive regulation of asexual sporulation resulting in formation of a cellular spore, regulation of transcription, DNA-dependent	
**AN4414.3**	**ANIA_04414**	**3.26**			Putative diphosphomevalonate decarboxylase with a predicted role in sterol metabolism	Mevalonate pyrophosphate decarboxylase [I]
**AN4481.3**	**ANIA_04481**	**3.39**			Ortholog(s) have role in secondary metabolite biosynthetic process and fungal-type vacuole membrane localization	Monocarboxylate transporter [G]
**AN4506.3**	**ANIA_04506**	**2.17**			Ortholog of A. fumigatus Af293 : Afu2g03250, A. niger CBS 513.88 : An07g07680, A. oryzae RIB40 : AO090120000262, Aspergillus wentii : Aspwe1_0059757 and Aspergillus sydowii : Aspsy1_0073302	
**AN4527.3**	**ANIA_04527**	**5.40**			Has domain(s) with predicted DNA binding, chromatin binding activity	
**AN4529.3**	**ANIA_04529**	**2.07**			Ortholog of A. niger CBS 513.88 : An07g07320, Aspergillus wentii : Aspwe1_0024851, Aspergillus versicolor : Aspve1_0739308 and Aspergillus clavatus NRRL 1 : ACLA_091870	
**AN4542.3**	**ANIA_04542**	**2.02**			Ortholog of A. fumigatus Af293 : Afu2g02730, A. niger CBS 513.88 : An07g07060, A. oryzae RIB40 : AO090026000848, Aspergillus wentii : Aspwe1_0168873 and Aspergillus sydowii : Aspsy1_0091100	
**AN4547.3**	**ANIA_04547**	**2.45**			Ortholog(s) have role in establishment or maintenance of cell polarity and cytosol localization	Vesicle coat complex COPI, gamma subunit [U]
**AN4571.3**	**ANIA_04571**	**2.07**			Ortholog(s) have Y-form DNA binding activity, role in intra-S DNA damage checkpoint, mitotic DNA replication checkpoint, nuclear division, replication fork protection and nuclear chromatin localization	HIV-1 Vpr-binding protein [D]
**AN4575.3**	**ANIA_04575**	**3.78**			Ortholog of A. oryzae RIB40 : AO090011000461, Aspergillus flavus NRRL 3357 : AFL2T_05228, Aspergillus versicolor : Aspve1_0042983 and Aspergillus sydowii : Aspsy1_0091136	
**AN4586.3**	**ANIA_04586**	**3.98**		Transcription factor	Has domain(s) with predicted nucleic acid binding, zinc ion binding activity and intracellular localization	

**AN4622.3**	**ANIA_04622**	**3.54**			Ortholog of A. fumigatus Af293 : Afu2g02380, A. niger CBS 513.88 : An07g06420, A. oryzae RIB40 : AO090011000518, Aspergillus wentii : Aspwe1_0168908 and Aspergillus sydowii : Aspsy1_0155773	
**AN4642.3**	**ANIA_04642**	**2.48**			Ortholog of A. nidulans FGSC A4 : AN6924, AN5943, AN8512, AN8548, AN0857, AN8661 and A. fumigatus Af293 : Afu1g15290, Afu3g00850, Afu4g08850, Afu7g00920	
**AN4650.3**	**ANIA_04650**	**3.38**			Ortholog of A. fumigatus Af293 : Afu2g01790, A. niger CBS 513.88 : An07g05410, A. oryzae RIB40 : AO090011000436, Aspergillus wentii : Aspwe1_0112486 and Aspergillus sydowii : Aspsy1_0059416	Predicted Ca2+-dependent phospholipid-binding protein [R]
**AN4653.3**	**ANIA_04653**	**2.04**			Ortholog(s) have cytoplasm, nucleus localization	Predicted phosphoglycerate mutase [G]
**AN4659.3**	**ANIA_04659**	**2.69**			Putative acyl-CoA synthetase/AMP-binding domain protein; has a predicted mitochondrial localization signal	Long chain fatty acid acyl-CoA ligase [I]
**AN4680.3**	**ANIA_04680**	**5.55**			Ortholog of A. fumigatus Af293 : Afu5g08990, A. niger CBS 513.88 : An07g04160, A. oryzae RIB40 : AO090020000471, Aspergillus wentii : Aspwe1_0048193 and Aspergillus sydowii : Aspsy1_0059440	
**AN4692.3**	**ANIA_04692**	**3.00**			Protein of unknown function	
**AN4698.3**	**ANIA_04698**	**5.26**			Ortholog of A. fumigatus Af293 : Afu5g08750, A. niger CBS 513.88 : An07g04700, Aspergillus wentii : Aspwe1_0278209, Aspergillus sydowii : Aspsy1_0155406 and Aspergillus terreus NIH2624 : ATET_06591	
**AN4699.3**	**ANIA_04699**	**3.24**			Ortholog of A. niger CBS 513.88 : An07g04660, A. oryzae RIB40 : AO090020000435, Aspergillus wentii : Aspwe1_0278331, Aspergillus versicolor : Aspve1_0053730 and Aspergillus clavatus NRRL 1 : ACLA_011760	
**AN4711.3**	**ANIA_04711**	**3.36**			Ortholog of A. fumigatus Af293 : Afu5g08650, A. niger CBS 513.88 : An07g04840, A. oryzae RIB40 : AO090020000424, Aspergillus wentii : Aspwe1_0064849 and Aspergillus sydowii : Aspsy1_1115966	
**AN4744.3**	**ANIA_04744**	**2.30**		Transcription factor	Has domain(s) with predicted DNA binding, sequence-specific DNA binding RNA polymerase II transcription factor activity, zinc ion binding activity and role in regulation of transcription, DNA-dependent, transcription, DNA-templated	
**AN4745.3**	**ANIA_04745**	**3.46**			Predicted Rho GTPase activating protein	Predicted Rho GTPase-activating protein [T]
**AN4795.3**	**ANIA_04795**	**4.38**			Has domain(s) with predicted guanyl-nucleotide exchange factor activity, role in small GTPase mediated signal transduction and intracellular localization	
**AN4801.3**	**ANIA_04801**	**4.81**			Protein of unknown function	
**AN4806.3**	**ANIA_04806**	**2.09**			Ortholog of A. fumigatus Af293 : Afu3g07000, A. niger CBS 513.88 : An02g13810, Aspergillus wentii : Aspwe1_0042056, Aspergillus sydowii : Aspsy1_0043180 and Aspergillus terreus NIH2624 : ATET_08722	
**AN4807.3**	**ANIA_04807**	**2.66**			Ortholog of A. niger CBS 513.88 : An02g13800, A. oryzae RIB40 : AO090020000293, Aspergillus wentii : Aspwe1_0052428, Aspergillus sydowii : Aspsy1_0087808 and Aspergillus terreus NIH2624 : ATET_08721	
**AN4809.3**	**ANIA_04809**	**4.24**	gtaA		Putative glutaminase A with a predicted role in glutamate and glutamine metabolism	
**AN4821.3**	**ANIA_04821**	**2.05**			Has domain(s) with predicted DNA binding, zinc ion binding activity, role in transcription, DNA-templated and nucleus localization	
**AN4825.3**	**ANIA_04825**	**2.43**			Putative glucan 1,3-beta-glucosidase with a predicted role in glucan metabolism	
**AN4838.3**	**ANIA_04838**	**2.59**			Ortholog of A. fumigatus Af293 : Afu3g07330, A. niger CBS 513.88 : An02g13390, Aspergillus wentii : Aspwe1_0173527, Aspergillus sydowii : Aspsy1_0087767 and Aspergillus terreus NIH2624 : ATET_01739	Uncharacterized conserved protein [S]
**AN4839.3**	**ANIA_04839**	**2.02**			Ortholog of A. fumigatus Af293 : Afu3g07340, A. niger CBS 513.88 : An02g13380, A. oryzae RIB40 : AO090020000189, Aspergillus wentii : Aspwe1_0559530 and Aspergillus sydowii : Aspsy1_0146522	
**AN4843.3**	**ANIA_04843**	**2.07**	agdG		Putative alpha-glucosidase with a predicted role in maltose metabolism	Alpha-amylase [G]
**AN4852.3**	**ANIA_04852**	**2.67**			Putative glucan 1,3-beta-glucosidase with a predicted role in glucan metabolism	
**AN4854.3**	**ANIA_04854**	**3.12**			Ortholog of A. fumigatus Af293 : Afu3g07570, A. niger CBS 513.88 : An02g13130, A. oryzae RIB40 : AO090020000139, Aspergillus wentii : Aspwe1_0050934 and Aspergillus sydowii : Aspsy1_0798750	
**AN4871.3**	**ANIA_04871**	**3.23**	chiB		Class V chitinase; glycoside hydrolase family 18 (GH18) protein with a role in the age-dependent autolysis	Chitinase [G]
**AN4897.3**	**ANIA_04897**	**2.68**			Ortholog(s) have role in asexual sporulation resulting in formation of a cellular spore, cellular response to heat and cell septum, cell surface localization	
**AN4921.3**	**ANIA_04921**	**2.52**			Ortholog of A. fumigatus Af293 : Afu3g10680, A. niger CBS 513.88 : An02g06340, A. oryzae RIB40 : AO090003000613, Aspergillus wentii : Aspwe1_0360214 and Aspergillus sydowii : Aspsy1_0056501	
**AN4950.3**	**ANIA_04950**	**2.14**			Ortholog of A. fumigatus Af293 : Afu3g10370, Neosartorya fischeri NRRL 181 : NFIA_066900, Aspergillus fumigatus A1163 : AFUB_038780 and Aspergillus clavatus NRRL 1 : ACLA_038270	
**AN4999.3**	**ANIA_04999**	**3.44**			Protein of unknown function	
**AN5033.3**	**ANIA_05033**	**2.81**			Has domain(s) with predicted substrate-specific transmembrane transporter activity, role in transmembrane transport and integral to membrane localization	
**AN5037.3**	**ANIA_05037**	**2.10**			Predicted glycosylphosphatidylinositol (GPI)-anchored protein	
**AN5057.3**	**ANIA_05057**	**3.57**	cdcA		Putative phosphoprotein phosphatase; reduced growth on AVICEL medium and reduced eglA and eglB expression	Protein tyrosine phosphatase CDC14 [V]
**AN5067.3**	**ANIA_05067**	**3.87**			Putative transporter of the major facilitator superfamily (MFS); expression reduced after exposure to farnesol	Predicted transporter (major facilitator superfamily) [R]
**AN5126.3**	**ANIA_05126**	**2.07**			Ortholog of A. fumigatus Af293 : Afu1g07490, Afu2g04420, A. niger CBS 513.88 : An11g03310, An07g10050, A. oryzae RIB40 : AO090012000999 and Aspergillus wentii : Aspwe1_0046371	Myosin class II heavy chain [Z]
**AN5135.3**	**ANIA_05135**	**2.66**			Ortholog of Aspergillus versicolor : Aspve1_0087700 and Aspergillus sydowii : Aspsy1_0047598	
**AN5167.3**	**ANIA_05167**	**6.39**			Has domain(s) with predicted phospholipid binding, zinc ion binding activity	
**AN5169.3**	**ANIA_05169**	**2.96**			Ortholog(s) have role in syncytium formation by plasma membrane fusion	
**AN5171.3**	**ANIA_05171**	**9.58**			Protein of unknown function	
**AN5201.3**	**ANIA_05201**	**2.37**			Ortholog of A. fumigatus Af293 : Afu6g07350, A. niger CBS 513.88 : An07g09090, Aspergillus wentii : Aspwe1_0109490, Aspergillus sydowii : Aspsy1_0059760 and Aspergillus terreus NIH2624 : ATET_03697	
**AN5213.3**	**ANIA_05213**	**4.18**			Ortholog(s) have Golgi apparatus, cell cortex of cell tip, cell division site localization	
**AN5221.3**	**ANIA_05221**	**2.80**			Ortholog of A. fumigatus Af293 : Afu2g04140, A. oryzae RIB40 : AO090005001543, Aspergillus wentii : Aspwe1_0172392, Aspergillus sydowii : Aspsy1_0047769 and Aspergillus terreus NIH2624 : ATET_03670	
**AN5228.3**	**ANIA_05228**	**2.69**			Putative NADH:flavin oxidoreductase/NADH oxidase; intracellular, menadione stress-induced protein	NADH:flavin oxidoreductase/12-oxophytodienoate reductase [C, R]
**AN5229.3**	**ANIA_05229**	**2.01**			Ortholog of A. fumigatus Af293 : Afu2g04050, A. niger CBS 513.88 : An07g08750, A. oryzae RIB40 : AO090005001534, Aspergillus wentii : Aspwe1_0515975 and Aspergillus sydowii : Aspsy1_0134382	TPR-containing nuclear phosphoprotein that regulates K(+) uptake [P]
**AN5235.3**	**ANIA_05235**	**3.28**			Ortholog(s) have role in transcription-coupled nucleotide-excision repair and Ddb1-Ckn1 complex, cytosol localization	K-coupled repair protein CSA, contains WD40 domain [K, L]

**AN5244.3**	**ANIA_05244**	**3.28**			Ortholog of A. nidulans FGSC A4 : AN5251, A. niger CBS 513.88 : An11g07550, An08g12150, A. oryzae RIB40 : AO090011000256, Aspergillus wentii : Aspwe1_0113272 and Aspergillus versicolor : Aspve1_0062915	
**AN5251.3**	**ANIA_05251**	**2.78**			Ortholog of A. nidulans FGSC A4 : AN5244, A. niger CBS 513.88 : An11g07550, An08g12150, A. oryzae RIB40 : AO090011000256, Aspergillus wentii : Aspwe1_0113272 and Aspergillus versicolor : Aspve1_0062915	
**AN5252.3**	**ANIA_05252**	**3.61**			Has domain(s) with predicted zinc ion binding activity and intracellular localization	
**AN5283.3**	**ANIA_05283**	**3.24**			Ortholog of A. nidulans FGSC A4 : AN2708, AN1572, AN7419, A. fumigatus Af293 : Afu6g14000, Afu8g05970, A. niger CBS 513.88 : An07g02380, An08g07270 and A. oryzae RIB40 : AO090026000716, AO090005000543	
**AN5312.3**	**ANIA_05312**	**4.38**			Ortholog of A. nidulans FGSC A4 : AN6413, AN6419, AN6946, AN5664, AN8328, AN3241, AN10369, AN1930, AN2386, AN1738, AN11159, AN8984, AN8951, AN1540, AN8971, AN9387, AN9266, AN9306, AN1317, AN10886, AN7232, AN7774, AN12202	
**AN5314.3**	**ANIA_05314**	**3.87**			Has domain(s) with predicted catalytic activity, pyridoxal phosphate binding activity and role in metabolic process	Molybdenum cofactor sulfurase [H]
**AN5318.3**	**ANIA_05318**	**2.39**			Putative non-ribosomal peptide synthetase; expression upregulated after exposure to farnesol	Non-ribosomal peptide synthetase/alpha-aminoadipate reductase and related enzymes [Q]
**AN5331.3**	**ANIA_05331**	**2.72**			Has domain(s) with predicted nucleotide binding, oxidoreductase activity and role in metabolic process	Predicted dehydrogenase [Q]
**AN5338.3**	**ANIA_05338**	**3.00**			Has domain(s) with predicted oxidoreductase activity and role in oxidation-reduction process	Flavin-containing monooxygenase [Q]
**AN5339.3**	**ANIA_05339**	**2.83**			Ortholog of A. oryzae RIB40 : AO090103000416, Aspergillus wentii : Aspwe1_0022264, Aspergillus sydowii : Aspsy1_0050291 and Aspergillus terreus NIH2624 : ATET_00936	
**AN5345.3**	**ANIA_05345**	**2.05**			Ortholog of A. fumigatus Af293 : Afu6g14190, A. niger CBS 513.88 : An06g01110, A. oryzae RIB40 : AO090103000511, Aspergillus wentii : Aspwe1_0099126 and Aspergillus sydowii : Aspsy1_0161355	
**AN5353.3**	**ANIA_05353**	**8.53**			Transcript repressed by light in developmentally competent mycelia	
**AN5365.3**	**ANIA_05365**	**2.12**			Protein of unknown function	
**AN5394.3**	**ANIA_05394**	**2.79**			Has domain(s) with predicted metal ion transmembrane transporter activity, role in metal ion transport, transmembrane transport and membrane localization	
**AN5402.3**	**ANIA_05402**	**2.20**			Has domain(s) with predicted role in lipid metabolic process	Intracellular membrane-bound Ca2+-independent phospholipase A2 [I]
**AN5408.3**	**ANIA_05408**	**3.13**			Has domain(s) with predicted RNA binding, ribonuclease III activity and role in RNA processing	
**AN5417.3**	**ANIA_05417**	**2.14**			Has domain(s) with predicted UDP-N-acetylmuramate dehydrogenase activity, flavin adenine dinucleotide binding activity and role in oxidation-reduction process	Proteins containing the FAD binding domain [C]
**AN5421.3**	**ANIA_05421**	**5.45**			Ortholog of A. nidulans FGSC A4 : AN4151, A. fumigatus Af293 : Afu5g07490, A. niger CBS 513.88 : An14g01660, An08g09760, An11g00210 and A. oryzae RIB40 : AO090012000034, AO090009000581, AO090012000374	Flavin-containing monooxygenase [Q]
**AN5422.3**	**ANIA_05422**	**4.76**			Putative beta-lactamase family protein; intracellular; protein abundance decreased by menadione stress	
**AN5423.3**	**ANIA_05423**	**2.61**			Ortholog of A. oryzae RIB40 : AO090003001412, Aspergillus glaucus : Aspgl1_0070324, Aspergillus flavus NRRL 3357 : AFL2T_01664 and Aspergillus kawachii : Aspka1_0182292	
**AN5453.3**	**ANIA_05453**	**2.15**	artG		Arrestin-like protein	
**AN5458.3**	**ANIA_05458**	**4.57**			Ortholog(s) have role in syncytium formation by plasma membrane fusion	
**AN5484.3**	**ANIA_05484**	**2.14**			Has domain(s) with predicted sterol binding activity	2-enoyl-CoA hydratase/3-hydroxyacyl-CoA dehydrogenase/Peroxisomal 3-ketoacyl-CoA-thiolase, sterol-binding domain and related enzymes [I]
**AN5487.3**	**ANIA_05487**	**4.46**			Ortholog of A. nidulans FGSC A4 : AN2619, A. niger CBS 513.88 : An11g05410, An12g09960, An12g02190 and Aspergillus wentii : Aspwe1_0167938	
**AN5488.3**	**ANIA_05488**	**3.45**			Protein of unknown function	
**AN5502.3**	**ANIA_05502**	**5.61**			Ortholog of Aspergillus sydowii : Aspsy1_0045941	
**AN5521.3**	**ANIA_05521**	**3.82**	alpA		Microtubule stabilizing, plus end-binding protein; Dis1/XMAP215 family, group 3; mutation causes polarity defects, hyphal curving, Spitzenkorper mislocalization, misplaced germ tube emergence, fewer and less dynamic cytoplasmic microtubules	Microtubule-associated protein [Z]
**AN5575.3**	**ANIA_05575**	**4.18**			Ortholog of A. fumigatus Af293 : Afu4g11602, A. oryzae RIB40 : AO090003001575, Aspergillus wentii : Aspwe1_0220437, Aspergillus sydowii : Aspsy1_0870778 and Aspergillus terreus NIH2624 : ATET_03918	
**AN5583.3**	**ANIA_05583**	**3.15**		Transcription factor	Has domain(s) with predicted nucleic acid binding, zinc ion binding activity and intracellular localization	C2H2-type Zn-finger protein [K]
**AN5597.3**	**ANIA_05597**	**2.15**			Putative ubiquinol-cytochrome-c reductase subunit with a predicted role in energy metabolism	
**AN5617.3**	**ANIA_05617**	**2.06**			Has domain(s) with predicted ATP binding, DNA binding, nucleoside-triphosphatase activity and role in DNA repair	ATPase related to the helicase subunit of the Holliday junction resolvase [L]
**AN5621.3**	**ANIA_05621**	**3.40**			Ortholog of A. fumigatus Af293 : Afu4g11130, A. niger CBS 513.88 : An04g05550, Aspergillus wentii : Aspwe1_0022788, Aspergillus sydowii : Aspsy1_0088403 and Aspergillus terreus NIH2624 : ATET_03967	
**AN5623.3**	**ANIA_05623**	**2.35**			Ortholog(s) have mitochondrion localization	ATP-dependent RNA helicase pitchoune [A]
**AN5637.3**	**ANIA_05637**	**2.11**			Has domain(s) with predicted nucleotide binding, oxidoreductase activity and role in metabolic process	Reductases with broad range of substrate specificities [R]
**AN5646.3**	**ANIA_05646**	**3.33**			Putative enzyme with a predicted role in fatty acid degradation	3-oxoacyl CoA thiolase [I]
**AN5649.3**	**ANIA_05649**	**2.62**			Ortholog of A. nidulans FGSC A4 : AN1674, AN0384, AN1268, AN7262, A. fumigatus Af293 : Afu1g01700, Afu2g17000, Afu4g08730, Afu4g13630, Afu1g10012 and A. niger CBS 513.88 : An01g09380, An01g06520, An04g09080, An04g03600	U4/U6-associated splicing factor PRP4 [A]
**AN5664.3**	**ANIA_05664**	**6.81**			Ortholog of A. nidulans FGSC A4 : AN6413, AN6419, AN6946, AN5312, AN8328, AN3241, AN10369, AN1930, AN2386, AN1738, AN11159, AN8984, AN8951, AN1540, AN8971, AN9387, AN9266, AN9306, AN1317, AN10886, AN7232, AN7774, AN12202	
**AN5665.3**	**ANIA_05665**	**2.31**	CYP531D2		Putative cytochrome P450	Cytochrome P450 CYP4/CYP19/CYP26 subfamilies [Q, I]
**AN5667.3**	**ANIA_05667**	**2.11**			Ortholog of A. fumigatus Af293 : Afu4g13730, A. oryzae RIB40 : AO090009000198, Aspergillus wentii : Aspwe1_0047212, Aspergillus sydowii : Aspsy1_0030299 and Aspergillus terreus NIH2624 : ATET_03253	
**AN5687.3**	**ANIA_05687**	**4.89**			Ortholog of A. fumigatus Af293 : Afu7g04200, A. niger CBS 513.88 : An13g00750, A. oryzae RIB40 : AO090005001653, Aspergillus wentii : Aspwe1_0038498 and Aspergillus sydowii : Aspsy1_0030277	
**AN5696.3**	**ANIA_05696**	**5.28**	ntpA		Nitric oxide-induced nitrosothionein involved in NO detoxification	
**AN5712.3**	**ANIA_05712**	**2.55**	casA		Putative metacaspase	Metacaspase involved in regulation of apoptosis [D, O]
**AN5714.3**	**ANIA_05714**	**2.20**			Ortholog of A. fumigatus Af293 : Afu1g06762, A. niger CBS 513.88 : An18g05800, A. oryzae RIB40 : AO090001000477, Aspergillus wentii : Aspwe1_0109415 and Aspergillus sydowii : Aspsy1_0150388	
**AN5716.3**	**ANIA_05716**	**2.19**			Putative inosine-5'-monophosphate dehydrogenase with a predicted role in purine metabolism	

**AN5733.3**	**ANIA_05733**	**2.19**		Ortholog of A. fumigatus Af293 : Afu6g06970, A. oryzae RIB40 : AO090003000079, Neosartorya fischeri NRRL 181 : NFIA_052620, Aspergillus wentii : Aspwe1_0050160 and Aspergillus versicolor : Aspve1_0132614	
**AN5759.3**	**ANIA_05759**	**2.08**	stk19	Ortholog(s) have role in ascospore formation, sporocarp development involved in sexual reproduction	Serine/threonine protein kinase [T]
**AN5762.3**	**ANIA_05762**	**3.63**		Predicted glycosylphosphatidylinositol (GPI)-anchored protein	
**AN5763.3**	**ANIA_05763**	**3.83**		Has domain(s) with predicted role in transmembrane transport and integral to membrane localization	Synaptic vesicle transporter SVOP and related transporters (major facilitator superfamily) [R]
**AN5768.3**	**ANIA_05768**	**3.39**		Ortholog(s) have role in actin cytoskeleton organization, inositol lipid-mediated signaling, vacuole organization and plasma membrane localization	
**AN5776.3**	**ANIA_05776**	**2.65**		Ortholog(s) have role in hypotonic response, intracellular transport, syncytium formation by plasma membrane fusion and cell septum localization	Thyroid hormone receptor-associated protein complex, subunit TRAP230 [K]
**AN5811.3**	**ANIA_05811**	**3.26**		Ortholog of A. fumigatus Af293 : Afu2g07510, A. niger CBS 513.88 : An05g00060, A. oryzae RIB40 : AO090011000941, Neosartorya fischeri NRRL 181 : NFIA_083250 and Aspergillus wentii : Aspwe1_0043750	
**AN5821.3**	**ANIA_05821**	**4.26**		Putative vacuolar H+/Ca++ exchanger; transcript induced in response to calcium dichloride in a CrzA-dependent manner	Ca2+/H+ antiporter VCX1 and related proteins [P]
**AN5831.3**	**ANIA_05831**	**3.07**		Ortholog of A. nidulans FGSC A4 : AN10273/gst3, A. fumigatus Af293 : Afu2g15770, A. niger CBS 513.88 : An15g06030, An05g00350 and A. oryzae RIB40 : AO090011000920	Predicted glutathione S-transferase [O]
**AN5834.3**	**ANIA_05834**	**3.35**		Predicted glycosylphosphatidylinositol (GPI)-anchored protein	
**AN5878.3**	**ANIA_05878**	**4.15**		Has domain(s) with predicted transferase activity, transferring acyl groups other than amino-acyl groups activity and role in metabolic process	3-oxoacyl CoA thiolase [I]
**AN5883.3**	**ANIA_05883**	**2.78**	metF	Putative methylenetetrahydrofolate reductase (NADPH) with predicted role in one-carbon metabolism; mutation causes methionine auxotrophy and decreased mycelial pigment production; expression induced by homocysteine and decreased by farnesol	5,10-methylenetetrahydrofolate reductase [E]
**AN5908.3**	**ANIA_05908**	**2.95**	tpiB	Putative triose-phosphate isomerase with a predicted role in glycolysis	Triosephosphate isomerase [G]
**AN5914.3**	**ANIA_05914**	**2.07**		Has domain(s) with predicted methyltransferase activity	
**AN5936.3**	**ANIA_05936**	**4.29**		Protein of unknown function	
**AN5940.3**	**ANIA_05940**	**5.33**		Has domain(s) with predicted carbon-sulfur lyase activity and role in metabolic process	
**AN5942.3**	**ANIA_05942**	**2.39**		Ortholog of Aspergillus versicolor : Aspve1_0148858 and Aspergillus sydowii : Aspsy1_0029071	
**AN5945.3**	**ANIA_05945**	**6.48**		Ortholog of A. fumigatus Af293 : Afu6g14630, Neosartorya fischeri NRRL 181 : NFIA_060670 and Aspergillus terreus NIH2624 : ATET_10017	Predicted ubiquitin-protein ligase/hyperplastic discs protein, HECT superfamily [O]
**AN5967.3**	**ANIA_05967**	**2.11**		Protein of unknown function	
**AN6013.3**	**ANIA_06013**	**3.80**		Ortholog(s) have S-adenosylmethionine-dependent methyltransferase activity, role in protein methylation and cytosol, nucleus localization	
**AN6016.3**	**ANIA_06016**	**4.09**		Protein of unknown function	
**AN6023.3**	**ANIA_06023**	**2.19**		Has domain(s) with predicted FMN binding, oxidoreductase activity and role in oxidation-reduction process	
**AN6046.3**	**ANIA_06046**	**3.61**		Putative p67phox regulatory subunit homolog with a predicted role in regulating hyphal reactive oxygen species (ROS) production; required for normal sexual and asexual development	
**AN6048.3**	**ANIA_06048**	**3.27**		Putative aspartate transaminase with a predicted role in amino acid metabolism; palA-dependent expression independent of pH; induced by carbon starvation-induced autophagy	Aspartate aminotransferase/Glutamic oxaloacetic transaminase AAT2/GOT1 [E]
**AN6050.3**	**ANIA_06050**	**2.82**		Ortholog of A. fumigatus Af293 : Afu2g09630, A. niger CBS 513.88 : An16g05640, An11g07160, A. oryzae RIB40 : AO090011000692, Aspergillus wentii : Aspwe1_0119323 and Aspergillus sydowii : Aspsy1_0057969	
**AN6088.3**	**ANIA_06088**	**2.45**		Predicted metal ion transmembrane transporter; upregulated in A. oryzae and A. nidulans under hypoxic growth conditions	Uncharacterized conserved protein [S]
**AN6125.3**	**ANIA_06125**	**2.90**	nudE	Protein with homology to Neurospora crassa RO11; multicopy suppressor of nudF mutation; physically interacts with NudF	LIS1-interacting protein NUDE [Z]
**AN6132.3**	**ANIA_06132**	**2.44**		Has domain(s) with predicted transferase activity, transferring phosphorus-containing groups activity	
**AN6168.3**	**ANIA_06168**	**2.12**	maeA	Putative malate dehydrogenase with a predicted role in oxidation of malate to pyruvate	NADP+-dependent malic enzyme [C]
**AN6177.3**	**ANIA_06177**	**2.16**	floA	Putative flotillin ortholog, involved in maintenance of sterol-rich plasma membrane domains; required for apical localization of cell-end marker proteins; mutation causes abnormal hyphal morphology	Flotillins [U, Z]
**AN6192.3**	**ANIA_06192**	**4.68**	ffkG	Has domain(s) with predicted ATP binding, protein tyrosine kinase activity and role in protein phosphorylation	MEKK and related serine/threonine protein kinases [T]
**AN6227.3**	**ANIA_06227**	**2.29**		Ortholog(s) have cytosol, nucleus localization	Cysteine desulfurase NFS1 [E]
**AN6241.3**	**ANIA_06241**	**2.79**		Ortholog of A. fumigatus Af293 : Afu2g13160, A. niger CBS 513.88 : An02g01890, A. oryzae RIB40 : AO090026000276, Aspergillus wentii : Aspwe1_0663843 and Aspergillus sydowii : Aspsy1_0151393	Vesicle coat complex COPII, subunit SEC31 [U]
**AN6259.3**	**ANIA_06259**	**4.00**		Ortholog of A. fumigatus Af293 : Afu3g15070, Neosartorya fischeri NRRL 181 : NFIA_062010, Aspergillus versicolor : Aspve1_0052545 and Aspergillus fumigatus A1163 : AFUB_034140	
**AN6324.3**	**ANIA_06324**	**2.76**	amyE	Putative alpha-amylase with a predicted role in starch metabolism; predicted glycosylphosphatidylinositol (GPI)-anchored protein	Alpha-amylase [G]
**AN6327.3**	**ANIA_06327**	**2.47**		Protein of unknown function	
**AN6340.3**	**ANIA_06340**	**2.00**	klpA	Kinesin-family protein with a role in the establishment of spindle pole bipolarity	Kinesin (KAR3 subfamily) [Z]
**AN6352.3**	**ANIA_06352**	**7.21**	abnB	Protein with arabinan endo-1,5-alpha-L-arabinosidase activity, involved in degradation of pectin	
**AN6370.3**	**ANIA_06370**	**5.97**		Ortholog of A. fumigatus Af293 : Afu2g14010, A. oryzae RIB40 : AO090026000159, Aspergillus wentii : Aspwe1_0696714, Aspergillus sydowii : Aspsy1_0031745 and Aspergillus terreus NIH2624 : ATET_01392	
**AN6371.3**	**ANIA_06371**	**3.33**		Ortholog of A. fumigatus Af293 : Afu2g14000, A. niger CBS 513.88 : An02g04960, A. oryzae RIB40 : AO090026000160, Aspergillus wentii : Aspwe1_0072469 and Aspergillus sydowii : Aspsy1_0150852	
**AN6373.3**	**ANIA_06373**	**6.80**		Ortholog of Aspergillus wentii : Aspwe1_0696581, Aspergillus zonatus : Aspzo1_0142023 and Aspergillus sydowii : Aspsy1_0999397	
**AN6374.3**	**ANIA_06374**	**2.03**		Ortholog(s) have ATP-dependent DNA helicase activity, ATP-dependent DNA/RNA helicase activity, ATP-dependent RNA helicase activity and role in maturation of LSU-rRNA from tricistronic rRNA transcript (SSU-rRNA, 5.8S rRNA, LSU-rRNA)	RNA helicase [A]
**AN6380.3**	**ANIA_06380**	**2.88**		Ortholog of A. fumigatus Af293 : Afu2g13930, A. niger CBS 513.88 : An02g05120, A. oryzae RIB40 : AO090026000179, Neosartorya fischeri NRRL 181 : NFIA_089090 and Aspergillus versicolor : Aspve1_0052622	
**AN6404.3**	**ANIA_06404**	**4.20**		Has domain(s) with predicted zinc ion binding activity	
**AN6412.3**	**ANIA_06412**	**4.20**		Has domain(s) with predicted transporter activity, role in transmembrane transport and integral to membrane, plasma membrane localization	Vesicular amine transporter [U]
**AN6421.3**	**ANIA_06421**	**2.09**		Has domain(s) with predicted metal ion binding, phosphoric diester hydrolase activity	

**AN6423.3**	**ANIA_06423**	**3.33**			Ortholog of A. fumigatus Af293 : Afu3g00960, A. niger CBS 513.88 : An16g08680, A. oryzae RIB40 : AO090009000250 and Aspergillus wentii : Aspwe1_0045294, Aspwe1_0109966	
**AN6446.3**	**ANIA_06446**	**6.09**	cicD		Predicted transcription factor; coregulated with the NRPS/AN6444; encoded within the cichorine gene cluster; possible transcriptional regulator of cluster	K factor, Myb superfamily [K]
**AN6447.3**	**ANIA_06447**	**4.38**	cicE		Predicted O-methyltransferase; coregulated with the PKS pkbA/AN6448; encoded within the cichorine gene cluster	Hydroxyindole-O-methyltransferase and related SAM-dependent methyltransferases [R]
**AN6470.3**	**ANIA_06470**	**7.27**			Protein with lysozyme activity, involved in carbohydrate catabolism	
**AN6476.3**	**ANIA_06476**	**3.35**			Protein of unknown function	
**AN6535.3**	**ANIA_06535**	**6.24**			Transcript induced in response to calcium dichloride in a CrzA-dependent manner	
**AN6541.3**	**ANIA_06541**	**2.10**			Putative ligase with a predicted role in purine metabolism	Glycinamide ribonucleotide synthetase (GARS)/Aminoimidazole ribonucleotide synthetase (AIRS) [F]
**AN6555.3**	**ANIA_06555**	**2.48**			Ortholog(s) have role in medium-chain fatty acid biosynthetic process	Alpha/beta hydrolase [R]
**AN6560.3**	**ANIA_06560**	**2.20**			Ortholog of A. fumigatus Af293 : Afu6g04600, A. niger CBS 513.88 : An15g00728, A. oryzae RIB40 : AO090701000086, Aspergillus wentii : Aspwe1_0324754 and Aspergillus sydowii : Aspsy1_0037161	
**AN6588.3**	**ANIA_06588**	**2.80**			Ortholog of A. fumigatus Af293 : Afu6g04300, A. niger CBS 513.88 : An15g01200, A. oryzae RIB40 : AO090701000121, Aspergillus wentii : Aspwe1_0048668 and Aspergillus sydowii : Aspsy1_0164200	
**AN6624.3**	**ANIA_06624**	**4.29**			Ortholog of A. niger CBS 513.88 : An15g01640, A. oryzae RIB40 : AO090701000161, Aspergillus wentii : Aspwe1_0048634, Aspergillus versicolor : Aspve1_0086150 and Aspergillus zonatus : Aspzo1_0135880	Kinesin light chain [Z]
**AN6625.3**	**ANIA_06625**	**4.98**			Ortholog of A. fumigatus Af293 : Afu6g03900, A. niger CBS 513.88 : An15g01620, A. oryzae RIB40 : AO090701000157, Aspergillus wentii : Aspwe1_0024331 and Aspergillus sydowii : Aspsy1_0092034	
**AN6645.3**	**ANIA_06645**	**2.27**	bioF		Putative 8-amino-7-oxononanoate synthase with a predicted role in Coenzyme A and pantothenate biosynthesis	5-aminolevulinate synthase [H]
**AN6658.3**	**ANIA_06658**	**2.29**			Has domain(s) with predicted oxidoreductase activity and role in oxidation-reduction process	Amine oxidase [Q]
**AN6691.3**	**ANIA_06691**	**2.22**			Ortholog of Aspergillus glaucus : Aspgl1_0117884, Neosartorya fischeri NRRL 181 : NFIA_005070, Aspergillus versicolor : Aspve1_0043949 and Aspergillus sydowii : Aspsy1_1018177	
**AN6696.3**	**ANIA_06696**	**3.77**			Ortholog(s) have chromatin binding activity, role in cellular response to DNA damage stimulus and Rpd3L-Expanded complex, mitochondrion localization	
**AN6703.3**	**ANIA_06703**	**3.08**			Putative major facilitator superfamily (MFS) permease	Predicted transporter (major facilitator superfamily) [R]
**AN6705.3**	**ANIA_06705**	**2.21**		Transcription factor	Ortholog(s) have DNA binding, DNA translocase activity	Chromatin remodeling factor subunit and related K factors [B]
**AN6718.3**	**ANIA_06718**	**2.63**			Has domain(s) with predicted ATP binding, nucleoside-triphosphatase activity	AAA+-type ATPase [O]
**AN6727.3**	**ANIA_06727**	**2.09**			Ortholog of A. nidulans FGSC A4 : AN5371, AN0386, A. fumigatus Af293 : Afu1g01610, Afu4g01060, Afu6g03180, Afu7g05880 and A. niger CBS 513.88 : An04g08650, An07g02000	
**AN6758.3**	**ANIA_06758**	**3.61**	ffkJ		Has domain(s) with predicted ATP binding, protein tyrosine kinase activity and role in protein phosphorylation	Tyrosine kinase specific for activated (GTP-bound) p21cdc42Hs [T]
**AN6769.3**	**ANIA_06769**	**2.40**			Has domain(s) with predicted metal ion transmembrane transporter activity, role in metal ion transport, transmembrane transport and membrane localization	
**AN6770.3**	**ANIA_06770**	**2.08**			Predicted amino acid transmembrane transporter	Amino acid transporters [E]
**AN6773.3**	**ANIA_06773**	**2.09**			Has domain(s) with predicted triglyceride lipase activity and role in lipid catabolic process	
**AN6797.3**	**ANIA_06797**	**3.09**			Protein of unknown function	
**AN6798.3**	**ANIA_06798**	**2.45**			Has domain(s) with predicted catalytic activity and role in metabolic process	Predicted L-carnitine dehydratase/alpha-methylacyl-CoA racemase [I]
**AN6802.3**	**ANIA_06802**	**5.80**			Protein of unknown function	
**AN6812.3**	**ANIA_06812**	**2.43**			Has domain(s) with predicted hydrolase activity, acting on carbon-nitrogen (but not peptide) bonds activity and role in nitrogen compound metabolic process	Carbon-nitrogen hydrolase [E]
**AN6818.3**	**ANIA_06818**	**2.77**			Ortholog of A. fumigatus Af293 : Afu8g00480, A. oryzae RIB40 : AO090010000026, Aspergillus flavus NRRL 3357 : AFL2T_11293 and Neosartorya fischeri NRRL 181 : NFIA_093990	
**AN6819.3**	**ANIA_06819**	**3.04**			Has domain(s) with predicted hydrolase activity, hydrolyzing O-glycosyl compounds activity and role in carbohydrate metabolic process	
**AN6825.3**	**ANIA_06825**	**2.42**			Ortholog(s) have Rab guanyl-nucleotide exchange factor activity, role in ER to Golgi vesicle-mediated transport and TRAPP complex, cytosol, nuclear envelope localization	Transport protein particle (TRAPP) complex subunit [U]
**AN6832.3**	**ANIA_06832**	**2.05**		Transcription factor	Has domain(s) with predicted DNA binding, sequence-specific DNA binding RNA polymerase II transcription factor activity, zinc ion binding activity and role in regulation of transcription, DNA-dependent, transcription, DNA-templated	
**AN6838.3**	**ANIA_06838**	**2.30**	tubC		Beta-tubulin, highly conserved component of microtubules; A. nidulans has two beta-tubulin genes, tubC and benA; expression of tubC increases during conidiation	Beta tubulin [Z]
**AN6839.3**	**ANIA_06839**	**3.64**			Protein of unknown function	
**AN6859.3**	**ANIA_06859**	**3.75**			Ortholog of A. fumigatus Af293 : Afu5g13070, A. niger CBS 513.88 : An14g06090, A. oryzae RIB40 : AO090120000432, Aspergillus wentii : Aspwe1_0107789 and Aspergillus sydowii : Aspsy1_0085559	
**AN6923.3**	**ANIA_06923**	**2.40**	hxtA		Putative high-affinity hexose transporter with a predicted role in hexose transport; induced upon starvation and during sexual development	Predicted transporter (major facilitator superfamily) [R]
**AN6929.3**	**ANIA_06929**	**3.46**			Putative alpha-L-rhamnosidase	
**AN6960.3**	**ANIA_06960**	**2.21**			Has domain(s) with predicted metal ion transmembrane transporter activity, role in metal ion transport, transmembrane transport and membrane localization	Notchless-like WD40 repeat-containing protein [S]
**AN6985.3**	**ANIA_06985**	**3.22**			Putative ribulokinase with a predicted role in ribulose metabolism	Ribulose kinase and related carbohydrate kinases [G]
**AN7026.3**	**ANIA_07026**	**2.11**			Ortholog of A. fumigatus Af293 : Afu4g04230, A. niger CBS 513.88 : An14g00600, A. oryzae RIB40 : AO090206000070, Aspergillus wentii : Aspwe1_0030288 and Aspergillus sydowii : Aspsy1_0092867	
**AN7055.3**	**ANIA_07055**	**2.68**			Has domain(s) with predicted oxidoreductase activity	Glycolate oxidase [C]
**AN7058.3**	**ANIA_07058**	**3.90**			Protein of unknown function	
**AN7059.3**	**ANIA_07059**	**2.84**			Ortholog of A. nidulans FGSC A4 : AN9338, A. niger CBS 513.88 : An12g03220, An01g01400, An12g02100, An09g02230 and A. oryzae RIB40 : AO090138000084	Putative translation initiation inhibitor UK114/IBM1 [J]
**AN7070.3**	**ANIA_07070**	**2.07**	pkgB		Beta-lactamase-type thioesterase; involved in production of alternariol and other secondary metabolites; predicted secondary metabolism gene cluster member; coregulated with the PKS pkgA/AN7071	Glyoxylase [R]
**AN7073.3**	**ANIA_07073**	**2.40**		Transcription factor	Putative transcription factor; predicted role in secondary metabolite production; predicted secondary metabolism gene cluster member; coregulated with the PKS pkgA/AN7071	
**AN7074.3**	**ANIA_07074**	**4.66**			Putative oxidoreductase; induced in pyrG mutant in response to excess uracil	Predicted dehydrogenase [Q]
**AN7075.3**	**ANIA_07075**	**11.70**			Has domain(s) with predicted UDP-N-acetylmuramate dehydrogenase activity, flavin adenine dinucleotide binding activity and role in oxidation-reduction process	Proteins containing the FAD binding domain [C]
**AN7081.3**	**ANIA_07081**	**2.15**			Predicted UDP-N-acetylmuramate dehydrogenase; predicted secondary metabolism gene cluster member	

**AN7126.3**	**ANIA_07126**	**2.29**			Ortholog of A. nidulans FGSC A4 : AN9262, A. fumigatus Af293 : Afu3g03140, Neosartorya fischeri NRRL 181 : NFIA_006010 and Aspergillus versicolor : Aspve1_0136655	
**AN7133.3**	**ANIA_07133**	**5.21**			Protein of unknown function	
**AN7148.3**	**ANIA_07148**	**3.78**			Ortholog of A. nidulans FGSC A4 : AN11171, A. fumigatus Af293 : Afu4g03595, A. niger CBS 513.88 : An02g01320, An14g01685, An03g06340 and A. oryzae RIB40 : AO090026000213, AO090026000142, AO090023000130	
**AN7154.3**	**ANIA_07154**	**2.30**			Has domain(s) with predicted nucleotide binding activity	
**AN7166.3**	**ANIA_07166**	**2.72**			Ortholog of A. nidulans FGSC A4 : AN3215, AN8609, A. fumigatus Af293 : Afu3g14210, Afu4g03360, Afu4g12510 and A. niger CBS 513.88 : An09g04200, An14g01840	
**AN7173.3**	**ANIA_07173**	**3.87**			Has domain(s) with predicted role in transmembrane transport and integral to membrane localization	Ca2+/H+ antiporter VCX1 and related proteins [P]
**AN7177.3**	**ANIA_07177**	**3.93**			Ortholog of A. fumigatus Af293 : Afu4g03322, A. niger CBS 513.88 : An14g01990, A. oryzae RIB40 : AO090011000139, Aspergillus sydowii : Aspsy1_0052594 and Aspergillus terreus NIH2624 : ATET_02136	
**AN7181.3**	**ANIA_07181**	**2.07**			Ortholog of A. fumigatus Af293 : Afu4g03200, A. oryzae RIB40 : AO090011000112, Neosartorya fischeri NRRL 181 : NFIA_030260 and Aspergillus versicolor : Aspve1_0140969	
**AN7183.3**	**ANIA_07183**	**4.26**			Protein of unknown function; transcript upregulated by nitrate limitation	
**AN7184.3**	**ANIA_07184**	**5.57**			Ortholog(s) have U2-type spliceosomal complex, cytosol localization	Uncharacterized conserved protein [S]
**AN7199.3**	**ANIA_07199**	**2.56**			Ortholog(s) have cytoplasm localization	Predicted NAD-dependent oxidoreductase [R]
**AN7207.3**	**ANIA_07207**	**2.05**			Protein of unknown function	
**AN7211.3**	**ANIA_07211**	**3.01**			Putative C-8,7 sterol isomerase with a predicted role in ergosterol biosynthesis	C-8,7 sterol isomerase [I]
**AN7213.3**	**ANIA_07213**	**2.68**			Ortholog of A. nidulans FGSC A4 : AN4040, A. fumigatus Af293 : Afu5g00960 and A. niger CBS 513.88 : An18g01180, An10g00910, An11g01220, An01g11560	
**AN7214.3**	**ANIA_07214**	**3.54**			Ortholog of A. nidulans FGSC A4 : AN8162, A. fumigatus Af293 : Afu5g01210, A. niger CBS 513.88 : An16g00660 and A. oryzae RIB40 : AO090102000383, AO090120000014, AO090001000406, AO090103000238	Amine oxidase [Q]
**AN7217.3**	**ANIA_07217**	**2.44**			Protein of unknown function	
**AN7231.3**	**ANIA_07231**	**2.36**			Ortholog(s) have serine-type endopeptidase activity and role in proteolysis	Hydrolytic enzymes of the alpha/beta hydrolase fold [O, R]
**AN7245.3**	**ANIA_07245**	**2.50**			Ortholog of A. niger CBS 513.88 : An01g06570, An06g00030, A. oryzae RIB40 : AO090023000010, AO090026000177, Neosartorya fischeri NRRL 181 : NFIA_055760 and Aspergillus wentii : Aspwe1_0032389, Aspwe1_0041672	
**AN7257.3**	**ANIA_07257**	**2.14**			Ortholog of A. oryzae RIB40 : AO090102000110, Aspergillus flavus NRRL 3357 : AFL2T_09560, Aspergillus wentii : Aspwe1_0182495 and Aspergillus versicolor : Aspve1_0050844	
**AN7258.3**	**ANIA_07258**	**3.42**			Ortholog(s) have cell cortex, peroxisome localization	Peroxisomal membrane protein MPV17 and related proteins [R]
**AN7263.3**	**ANIA_07263**	**2.27**			Ortholog of A. oryzae RIB40 : AO090012000392, AO090009000463, Aspergillus tubingensis : Asptu1_0036805, Aspergillus flavus NRRL 3357 : AFL2T_03298 and Aspergillus kawachii : Aspka1_0181434	
**AN7264.3**	**ANIA_07264**	**2.62**			Ortholog of A. fumigatus Af293 : Afu4g14460, A. niger CBS 513.88 : An06g02170, A. oryzae RIB40 : AO090001000622, Aspergillus wentii : Aspwe1_0233835 and Aspergillus sydowii : Aspsy1_0090692	Methyltransferase [R]
**AN7266.3**	**ANIA_07266**	**5.12**			Protein of unknown function	
**AN7268.3**	**ANIA_07268**	**3.06**			Has domain(s) with predicted nucleotide binding, oxidoreductase activity and role in metabolic process	Dehydrogenases with different specificities (related to short-chain alcohol dehydrogenases) [Q]
**AN7269.3**	**ANIA_07269**	**3.91**			Ortholog(s) have role in fumiquinazoline C biosynthetic process, secondary metabolite biosynthetic process	Proteins containing the FAD binding domain [C]
**AN7270.3**	**ANIA_07270**	**3.44**			Ortholog of A. oryzae RIB40 : AO090001000616	
**AN7313.3**	**ANIA_07313**	**2.45**			Putative beta-1,4-xylosidase	
**AN7323.3**	**ANIA_07323**	**3.36**			Covalently-bound cell wall protein; predicted glycosyl phosphatidylinositol (GPI)-anchor	
**AN7334.3**	**ANIA_07334**	**3.04**			Has domain(s) with predicted catalytic activity and role in metabolic process	Phospholipase D1 [I]
**AN7343.3**	**ANIA_07343**	**2.16**		Transcription factor	Putative Zn(II)2Cys6-domain containing transcription factor; transcript is induced by nitrate	
**AN7344.3**	**ANIA_07344**	**2.27**			Has domain(s) with predicted substrate-specific transmembrane transporter activity, role in transmembrane transport and integral to membrane localization	Predicted transporter (major facilitator superfamily) [R]
**AN7352.3**	**ANIA_07352**	**3.47**			Ortholog of A. fumigatus Af293 : Afu2g16390, A. niger CBS 513.88 : An15g06770, A. oryzae RIB40 : AO090102000224, Aspergillus wentii : Aspwe1_0039269 and Aspergillus sydowii : Aspsy1_0085045	
**AN7388.3**	**ANIA_07388**	**2.29**	cpeA		Putative catalase-peroxidase with a predicted role in gluconic acid and gluconate metabolism; protein also identified as laccase II which is expressed during sexual development	
**AN7407.3**	**ANIA_07407**	**3.33**			Ortholog of A. nidulans FGSC A4 : AN6148, A. fumigatus Af293 : Afu2g08500, Afu5g13710, A. niger CBS 513.88 : An02g14290, An12g03860 and A. oryzae RIB40 : AO090701000324, AO090011000852	Acetylcholinesterase/Butyrylcholin esterase [T]
**AN7418.3**	**ANIA_07418**	**3.39**			Has domain(s) with predicted oxidoreductase activity and role in metabolic process	
**AN7436.3**	**ANIA_07436**	**3.61**	pdiA		Putative protein disulfide isomerase; pdiA mRNA expression increased in the presence of farnesol	Protein disulfide isomerase (prolyl 4-hydroxylase beta subunit) [O]
**AN7477.3**	**ANIA_07477**	**2.19**			Ortholog of A. niger CBS 513.88 : An02g14040, Aspergillus versicolor : Aspve1_0044036, Aspergillus carbonarius ITEM 5010 : Acar5010_211768 and Aspergillus brasiliensis : Aspbr1_0191709	
**AN7510.3**	**ANIA_07510**	**4.43**			Has domain(s) with predicted role in transmembrane transport and integral to membrane localization	Ca2+/H+ antiporter VCX1 and related proteins [P]
**AN7517.3**	**ANIA_07517**	**2.41**			Ortholog of A. fumigatus Af293 : Afu5g03330, A. niger CBS 513.88 : An09g05650, A. oryzae RIB40 : AO090102000538, Aspergillus wentii : Aspwe1_0043027 and Aspergillus sydowii : Aspsy1_0146077	
**AN7518.3**	**ANIA_07518**	**4.62**			Has domain(s) with predicted oxidoreductase activity and role in oxidation-reduction process	Ferric reductase, NADH/NADPH oxidase and related proteins [P, Q]
**AN7519.3**	**ANIA_07519**	**3.04**			Protein of unknown function	
**AN7536.3**	**ANIA_07536**	**2.51**			Ortholog of A. nidulans FGSC A4 : AN2922, AN1515, A. niger CBS 513.88 : An12g10030, A. oryzae RIB40 : AO090701000305, AO090102000038, AO090124000043 and Aspergillus wentii : Aspwe1_0030521	
**AN7548.3**	**ANIA_07548**	**2.67**			Ortholog of A. fumigatus Af293 : Afu2g14740, A. niger CBS 513.88 : An15g03570, A. oryzae RIB40 : AO090026000805, Aspergillus wentii : Aspwe1_0118903 and Aspergillus sydowii : Aspsy1_0045980	Endoplasmic reticulum protein EP58, contains filamin rod domain and KDEL motif [R]
**AN7549.3**	**ANIA_07549**	**2.32**			Transcript induced in response to calcium dichloride in a CrzA-dependent manner	AAA+-type ATPase [O]
**AN7552.3**	**ANIA_07552**	**4.46**			Ortholog of A. fumigatus Af293 : Afu2g14790, A. oryzae RIB40 : AO090026000798, Aspergillus wentii : Aspwe1_0692743, Aspergillus sydowii : Aspsy1_0058559 and Aspergillus terreus NIH2624 : ATET_07821	Vesicle coat complex COPII, subunit SFB3 [U]
**AN7553.3**	**ANIA_07553**	**2.05**	devR	Transcription factor	Basic helix-loop-helix transcription factor required for normal conidiophore development; mutants are infertile in self-crosses	
**AN7580.3**	**ANIA_07580**	**2.80**			Ortholog of A. fumigatus Af293 : Afu2g15110, A. niger CBS 513.88 : An15g02960, A. oryzae RIB40 : AO090012000329, Aspergillus wentii : Aspwe1_0044605 and Aspergillus sydowii : Aspsy1_0046014	

**AN7583.3**	**ANIA_07583**	**6.89**			Ortholog of A. fumigatus Af293 : Afu2g15170, A. niger CBS 513.88 : An09g03380, Aspergillus wentii : Aspwe1_0117969, Aspergillus sydowii : Aspsy1_0152812 and Aspergillus terreus NIH2624 : ATET_07870	
**AN7592.3**	**ANIA_07592**	**7.06**		Transcription factor	Has domain(s) with predicted sequence-specific DNA binding RNA polymerase II transcription factor activity, zinc ion binding activity, role in regulation of transcription, DNA-dependent and nucleus localization	Phosphatidylethanolamine binding protein [R]
**AN7596.3**	**ANIA_07596**	**2.24**		Transcription factor	Has domain(s) with predicted sequence-specific DNA binding RNA polymerase II transcription factor activity, zinc ion binding activity, role in regulation of transcription, DNA-dependent and nucleus localization	
**AN7597.3**	**ANIA_07597**	**2.00**			Ortholog(s) have tetrapeptide transmembrane transporter activity and role in cellular response to drug, conjugation with cellular fusion, nitrogen utilization, tetrapeptide transmembrane transport	Sexual differentiation process protein ISP4 [T]
**AN7635.3**	**ANIA_07635**	**4.40**	binA		Transcript induced by exposure of A. nidulans to bafilomycin B1	
**AN7669.3**	**ANIA_07669**	**2.18**			Has domain(s) with predicted hydrolase activity, acting on carbon-nitrogen (but not peptide) bonds, in linear amidines, metal ion binding activity	Arginase family protein [E]
**AN7670.3**	**ANIA_07670**	**3.96**			Ortholog of A. fumigatus Af293 : Afu2g01430, Aspergillus wentii : Aspwe1_0114055, Aspergillus versicolor : Aspve1_0030702 and Aspergillus clavatus NRRL 1 : ACLA_093480	
**AN7680.3**	**ANIA_07680**	**3.38**			Ortholog of A. fumigatus Af293 : Afu2g01540, A. niger CBS 513.88 : An03g04930, A. oryzae RIB40 : AO090701000796, Aspergillus wentii : Aspwe1_0052178 and Aspergillus sydowii : Aspsy1_0090365	Myosin class II heavy chain [Z]
**AN7710.3**	**ANIA_07710**	**2.49**			Ortholog(s) have intracellular localization	Predicted haloacid-halidohydrolase and related hydrolases [R]
**AN7716.3**	**ANIA_07716**	**5.40**			Ortholog of A. fumigatus Af293 : Afu5g08180, A. oryzae RIB40 : AO090701000735, Aspergillus wentii : Aspwe1_0167587, Aspergillus sydowii : Aspsy1_0046259 and Aspergillus terreus NIH2624 : ATET_08308	
**AN7727.3**	**ANIA_07727**	**5.27**			Ortholog of A. fumigatus Af293 : Afu5g07980, Afu5g14920, A. niger CBS 513.88 : An03g03960, A. oryzae RIB40 : AO090701000712, Aspergillus wentii : Aspwe1_0271934 and Aspergillus sydowii : Aspsy1_0032303	
**AN7737.3**	**ANIA_07737**	**2.67**	mek1		Ortholog(s) have protein serine/threonine kinase activity, role in meiosis, meiotic recombination checkpoint, protein phosphorylation, regulation of linear element assembly and cytosol, linear element localization	Serine/threonine protein kinase Chk2 and related proteins [D]
**AN7745.3**	**ANIA_07745**	**2.55**			Has domain(s) with predicted role in phosphatidylinositol dephosphorylation, phosphatidylinositol phosphorylation	Inositol-1,4,5-triphosphate 5-phosphatase (synaptojanin), INP51/INP52/INP53 family [U]
**AN7772.3**	**ANIA_07772**	**2.23**	CYP5080B1		Putative cytochrome P450; transcript repressed by nitrate	Cytochrome P450 CYP2 subfamily [Q]
**AN7795.3**	**ANIA_07795**	**2.10**	gprK		Putative heterotrimeric G-protein coupled receptor component; contains both a 7-transmembrane domain and an RGS signaling domain	
**AN7804.3**	**ANIA_07804**	**6.61**	stcW		Putative FAD-containing monooxygenase with a predicted role in sterigmatocystin/aflatoxin biosynthesis; member of the sterigmatocystin biosynthesis gene cluster; expression upregulated after exposure to farnesol	Flavin-containing monooxygenase [Q]
**AN7805.3**	**ANIA_07805**	**3.90**	stcV		Putative norsolorinic acid reductase with a predicted role in sterigmatocystin/aflatoxin biosynthesis; member of the sterigmatocystin biosynthesis gene cluster	Voltage-gated shaker-like K+ channel, subunit beta/KCNAB [C]
**AN7807.3**	**ANIA_07807**	**2.44**	stcT		Putative translation elongation factor 1 gamma; member of the sterigmatocystin biosynthesis gene cluster	Glutathione S-transferase [O]
**AN7809.3**	**ANIA_07809**	**4.23**			Ortholog of Aspergillus versicolor : Aspve1_0126175 and Aspergillus terreus NIH2624 : ATET_01291	
**AN7810.3**	**ANIA_07810**	**3.57**	stcQ		Putative aflatoxin biosynthesis protein with a role in aflatoxin/sterigmatocystin biosynthesis; member of the sterigmatocystin biosynthesis gene cluster	
**AN7816.3**	**ANIA_07816**	**3.38**	stcI		Putative sterigmatocystin biosynthesis lipase/esterase with a predicted role in sterigmatocystin/aflatoxin biosynthesis; member of the sterigmatocystin biosynthesis gene cluster	Arylacetamide deacetylase [V]
**AN7818.3**	**ANIA_07818**	**2.33**	stcF		Putative sterigmatocystin biosynthesis P450 monooxygenase with a predicted role in sterigmatocystin/aflatoxin biosynthesis	Cytochrome P450 CYP4/CYP19/CYP26 subfamilies [Q, I]
**AN7821.3**	**ANIA_07821**	**8.89**	stcE		Putative norsolorinic acid reductase with a predicted role in sterigmatocystin/aflatoxin biosynthesis; member of the sterigmatocystin biosynthesis gene cluster	Predicted short chain-type dehydrogenase [R]
**AN7823.3**	**ANIA_07823**	**2.06**	stcC		Putative peroxidase; member of the sterigmatocystin biosynthesis gene cluster	
**AN7825.3**	**ANIA_07825**	**6.40**	stcA		Putative polyketide synthase with a predicted role in sterigmatocystin/aflatoxin biosynthesis; member of the sterigmatocystin biosynthesis gene cluster	Animal-type fatty acid synthase and related proteins [I]
**AN7836.3**	**ANIA_07836**	**3.49**			Ortholog of A. fumigatus Af293 : Afu7g01060, A. oryzae RIB40 : AO090003000833, Neosartorya fischeri NRRL 181 : NFIA_113770 and Aspergillus terreus NIH2624 : ATET_02590	
**AN7849.3**	**ANIA_07849**	**2.06**			Protein of unknown function	
**AN7873.3**	**ANIA_07873**	**4.42**			Putative multifunctional enzyme with a predicted role in cytosolic fatty acid formation; predicted secondary metabolism gene cluster member	Malonyl-CoA:ACP transacylase [I]
**AN7874.3**	**ANIA_07874**	**2.13**			Protein of unknown function; predicted secondary metabolism gene cluster member	Predicted thioesterase [R]
**AN7876.3**	**ANIA_07876**	**5.37**			Putative branched chain amino acid aminotransferase with a predicted role in branched chain amino acid biosynthesis; predicted secondary metabolism gene cluster member	Branched chain aminotransferase BCAT1, pyridoxal phosphate enzymes type IV superfamily [E]
**AN7877.3**	**ANIA_07877**	**2.00**			RTA-like protein; predicted secondary metabolism gene cluster member	
**AN7881.3**	**ANIA_07881**	**3.20**	CYP548C1		Putative cytochrome P450; predicted secondary metabolism gene cluster member	Cytochrome P450 CYP4/CYP19/CYP26 subfamilies [Q, I]
**AN7883.3**	**ANIA_07883**	**2.04**			Protein of unknown function; predicted secondary metabolism gene cluster member	
**AN7888.3**	**ANIA_07888**	**5.32**			Ortholog of A. fumigatus Af293 : Afu4g08450, Afu8g01980, A. niger CBS 513.88 : An15g05230, A. oryzae RIB40 : AO090023000157 and Aspergillus wentii : Aspwe1_0627621, Aspwe1_0672214	
**AN7894.3**	**ANIA_07894**	**2.03**			Protein of unknown function	
**AN7895.3**	**ANIA_07895**	**4.94**	cipB		Putative oxidoreductase; contains Zn-dependent alcohol dehydrogenase domain; protein expressed at increased levels during osmoadaptation	Zinc-binding oxidoreductase [C, R]
**AN7902.3**	**ANIA_07902**	**5.29**	dbaH		FAD-binding monooxygenase with a role in secondary metabolism; member of the dba gene cluster; dba cluster expression is coregulated	Kynurenine 3-monooxygenase and related flavoprotein monooxygenases [C, R]
**AN7911.3**	**ANIA_07911**	**2.58**	orsB		Putative amidohydrolase; member of the F9775 secondary metabolite gene cluster	Predicted metal-dependent hydrolase of the TIM-barrel fold [R]
**AN7912.3**	**ANIA_07912**	**2.96**	orsC		Putative tyrosinase; member of the F9775 secondary metabolite gene cluster	
**AN7914.3**	**ANIA_07914**	**7.73**			Putative alcohol dehydrogenase; member of the F9775 secondary metabolite gene cluster; induced by rapamycin-induced autophagy	Zinc-binding oxidoreductase [C, R]
**AN7939.3**	**ANIA_07939**	**2.14**			Ortholog(s) have intracellular localization	
**AN7941.3**	**ANIA_07941**	**3.15**			Ortholog of A. fumigatus Af293 : Afu2g12680, Afu7g00370, A. niger CBS 513.88 : An03g01770, Neosartorya fischeri NRRL 181 : NFIA_087830, NFIA_112920 and Aspergillus versicolor : Aspve1_0045447	
**AN7944.3**	**ANIA_07944**	**4.95**	ngn3		Putative GNAT-type acetyltransferase; expression upregulated after exposure to farnesol	
**AN7972.3**	**ANIA_07972**	**2.17**			Has domain(s) with predicted role in transmembrane transport and integral to membrane localization	Synaptic vesicle transporter SVOP and related transporters (major facilitator superfamily) [R]

**AN7997.3**	**ANIA_07997**	**2.50**			Ortholog(s) have chromatin insulator sequence binding, core RNA polymerase III binding transcription factor activity and role in transcription initiation from RNA polymerase III promoter	RNA polymerase III K factor (TF)IIIC subunit [K]
**AN7998.3**	**ANIA_07998**	**2.05**	xptC		GMC oxidoreductase; glucose oxidase-related protein; involved in a pathway leading to prenyl xanthone (shamixanthone and epishamixanthone) production	Glucose dehydrogenase/choline dehydrogenase/mandelonitrile lyase (GMC oxidoreductase family) [R]
**AN8004.3**	**ANIA_08004**	**3.64**	CYP541B1		Putative cytochrome P450	Cytochrome P450 CYP2 subfamily [Q]
**AN8060.3**	**ANIA_08060**	**4.16**			Has domain(s) with predicted NAD binding, oxidoreductase activity, acting on the aldehyde or oxo group of donors, NAD or NADP as acceptor activity and role in cellular amino acid metabolic process, oxidation-reduction process	
**AN8062.3**	**ANIA_08062**	**2.12**			Has domain(s) with predicted outer membrane localization	
**AN8069.3**	**ANIA_08069**	**4.65**			Has domain(s) with predicted membrane localization	Uncharacterized conserved protein [R]
**AN8093.3**	**ANIA_08093**	**4.13**			Ortholog of A. nidulans FGSC A4 : AN12197, A. fumigatus Af293 : Afu4g13910, Afu5g12760, A. niger CBS 513.88 : An15g06460, An13g02370 and A. oryzae RIB40 : AO090012000187	
**AN8094.3**	**ANIA_08094**	**2.23**			Protein of unknown function	
**AN8095.3**	**ANIA_08095**	**2.90**			Has domain(s) with predicted role in transmembrane transport and integral to membrane localization	Predicted transporter (major facilitator superfamily) [R]
**AN8098.3**	**ANIA_08098**	**4.26**			Protein of unknown function	
**AN8099.3**	**ANIA_08099**	**4.45**			Putative oxalate decarboxylase with a predicted role in oxalic acid metabolism	
**AN8102.3**	**ANIA_08102**	**3.66**	pepAc		Putative pepsin-like aspartic protease; predicted glycosyl phosphatidylinositol (GPI)-anchor	Aspartyl protease [O]
**AN8112.3**	**ANIA_08112**	**2.54**			Predicted ransmembrane transporter; predicted secondary metabolism gene cluster member	Predicted transporter (major facilitator superfamily) [R]
**AN8145.3**	**ANIA_08145**	**3.75**			Ortholog(s) have cytoplasm, nucleus localization	
**AN8152.3**	**ANIA_08152**	**2.44**			Has domain(s) with predicted UDP-N-acetylmuramate dehydrogenase activity, flavin adenine dinucleotide binding activity and role in oxidation-reduction process	Proteins containing the FAD binding domain [C]
**AN8153.3**	**ANIA_08153**	**2.52**			Has domain(s) with predicted ATP binding, nucleoside-triphosphatase activity	AAA+-type ATPase [O]
**AN8154.3**	**ANIA_08154**	**6.16**			Protein of unknown function	
**AN8157.3**	**ANIA_08157**	**3.37**			Ortholog of A. fumigatus Af293 : Afu1g01390 and Aspergillus fumigatus A1163 : AFUB_079400	
**AN8166.3**	**ANIA_08166**	**2.18**			Ortholog of A. niger CBS 513.88 : An09g04880, A. oryzae RIB40 : AO090102000486, Aspergillus wentii : Aspwe1_0042987, Aspergillus sydowii : Aspsy1_0161741 and Aspergillus terreus NIH2624 : ATET_09264	
**AN8174.3**	**ANIA_08174**	**2.41**			Ortholog of A. oryzae RIB40 : AO090102000187, AO090102000186, Aspergillus flavus NRRL 3357 : AFL2T_09624, Aspergillus kawachii : Aspka1_0182756 and Aspergillus versicolor : Aspve1_0089187	
**AN8175.3**	**ANIA_08175**	**6.93**			Has domain(s) with predicted viral capsid localization	
**AN8186.3**	**ANIA_08186**	**2.19**		Transcription factor	Predicted mariner transposon-related ORF	
**AN8218.3**	**ANIA_08218**	**5.07**	trxB		Putative thioredoxin reductase	
**AN8238.3**	**ANIA_08238**	**5.86**			Has domain(s) with predicted hydrolase activity, acting on carbon-nitrogen (but not peptide) bonds activity and role in nitrogen compound metabolic process	
**AN8266.3**	**ANIA_08266**	**2.39**			Ortholog of A. oryzae RIB40 : AO090138000120, Aspergillus flavus NRRL 3357 : AFL2T_08795, Neosartorya fischeri NRRL 181 : NFIA_062350 and Aspergillus versicolor : Aspve1_0047270	
**AN8278.3**	**ANIA_08278**	**2.07**			Ortholog of Neosartorya fischeri NRRL 181 : NFIA_037840, Aspergillus wentii : Aspwe1_0605622, Aspergillus versicolor : Aspve1_0047287 and Aspergillus clavatus NRRL 1 : ACLA_000900	
**AN8304.3**	**ANIA_08304**	**3.39**			Has domain(s) with predicted zinc ion binding activity	Ankyrin [M]
**AN8321.3**	**ANIA_08321**	**6.37**			Protein of unknown function	
**AN8323.3**	**ANIA_08323**	**4.49**			Protein of unknown function; transcript repressed by nitrate	
**AN8338.3**	**ANIA_08338**	**2.19**	CYP671A1		Putative cytochrome P450	Cytochrome P450 CYP4/CYP19/CYP26 subfamilies [Q, I]
**AN8351.3**	**ANIA_08351**	**2.33**			Ortholog of A. nidulans FGSC A4 : AN1611, A. fumigatus Af293 : Afu4g09220, A. niger CBS 513.88 : An04g04270, A. oryzae RIB40 : AO090023000614 and Aspergillus wentii : Aspwe1_0101354	Flavin-containing monooxygenase [Q]
**AN8360.3**	**ANIA_08360**	**2.28**			Has domain(s) with predicted oxidoreductase activity	
**AN8361.3**	**ANIA_08361**	**4.05**			Ortholog of A. fumigatus Af293 : Afu2g00370, A. niger CBS 513.88 : An12g10280, A. oryzae RIB40 : AO090701000625, Aspergillus wentii : Aspwe1_0168024 and Aspergillus sydowii : Aspsy1_0049880	
**AN8370.3**	**ANIA_08370**	**3.38**			Has domain(s) with predicted oxidoreductase activity and role in metabolic process	
**AN8376.3**	**ANIA_08376**	**2.64**			Ortholog of A. nidulans FGSC A4 : AN7265, AN8625 and A. fumigatus Af293 : Afu6g00680, Afu8g06420	
**AN8394.3**	**ANIA_08394**	**2.43**			Has domain(s) with predicted transmembrane transporter activity, role in transmembrane transport and integral to membrane localization	Predicted transporter (major facilitator superfamily) [R]
**AN8400.3**	**ANIA_08400**	**2.07**			Sugar transporter; transcriptionally induced by growth on xylose	Predicted transporter (major facilitator superfamily) [R]
**AN8433.3**	**ANIA_08433**	**7.60**			Has domain(s) with predicted catalytic activity and role in metabolic process	Acyl-CoA synthetase [I]
**AN8434.3**	**ANIA_08434**	**6.88**			Ortholog of A. fumigatus Af293 : Afu7g06200, Aspergillus wentii : Aspwe1_0053695, Aspergillus versicolor : Aspve1_0033713, Aspergillus zonatus : Aspzo1_0016352 and Aspergillus sydowii : Aspsy1_0061228	Ankyrin repeat and DHHC-type Zn-finger domain containing proteins [R]
**AN8435.3**	**ANIA_08435**	**3.12**			Protein of unknown function; transcript repressed by nitrate	Microtubule-associated protein TAU [Z]
**AN8438.3**	**ANIA_08438**	**3.29**	CYP658A1		Putative cytochrome P450	Cytochrome P450 [Q]
**AN8439.3**	**ANIA_08439**	**2.77**			Protein of unknown function; transcript is induced by nitrate; predicted NirA binding site	
**AN8448.3**	**ANIA_08448**	**2.15**			Has domain(s) with predicted substrate-specific transmembrane transporter activity, role in transmembrane transport and integral to membrane localization	Predicted transporter (major facilitator superfamily) [R]
**AN8461.3**	**ANIA_08461**	**4.28**			Ortholog(s) have IgE binding activity	
**AN8468.3**	**ANIA_08468**	**2.25**			Ortholog of A. nidulans FGSC A4 : AN2021, A. fumigatus Af293 : Afu7g00550 and A. niger CBS 513.88 : An15g05630, An11g03460, An11g07530, An04g03850, An09g00640	Notchless-like WD40 repeat-containing protein [S]
**AN8478.3**	**ANIA_08478**	**5.42**			Ortholog of A. fumigatus Af293 : Afu2g00870, A. oryzae RIB40 : AO090020000348, Neosartorya fischeri NRRL 181 : NFIA_099010, Aspergillus wentii : Aspwe1_0174900 and Aspergillus versicolor : Aspve1_0367643	
**AN8486.3**	**ANIA_08486**	**2.12**			Protein of unknown function	
**AN8489.3**	**ANIA_08489**	**4.43**	atrB		Putative plasma membrane ATP-binding cassette (ABC) transporter with a predicted role in multidrug resistance; transcript induced by the fungicide imazalil, by camptothecin and by farnesol	Pleiotropic drug resistance proteins (PDR1-15), ABC superfamily [Q]
**AN8490.3**	**ANIA_08490**	**2.39**			Ortholog of Aspergillus versicolor : Aspve1_0369297 and Aspergillus sydowii : Aspsy1_0471403	
**AN8492.3**	**ANIA_08492**	**3.48**			Protein of unknown function	
**AN8494.3**	**ANIA_08494**	**3.43**			Has domain(s) with predicted 2-dehydropantoate 2-reductase activity, NADP binding activity, role in oxidation-reduction process, pantothenate biosynthetic process and cytoplasm localization	
**AN8500.3**	**ANIA_08500**	**3.73**			Ortholog of A. niger CBS 513.88 : An13g02820, A. oryzae RIB40 : AO090009000060, Aspergillus wentii : Aspwe1_0122164, Aspergillus versicolor : Aspve1_0370452 and Aspergillus niger ATCC 1015 : 192087-mRNA	
**AN8505.3**	**ANIA_08505**	**2.10**			Ortholog of Aspergillus brasiliensis : Aspbr1_0111846, Aspergillus fumigatus A1163 : AFUB_075260, Aspergillus niger ATCC 1015 : 199457-mRNA and Aspergillus aculeatus ATCC16872 : Aacu16872_050877	WD40 repeat-containing protein [R]

**AN8513.3**	**ANIA_08513**	**2.38**	tdiA	Putative single-module nonribosomal peptide synthetase (NRPS); member of the tdi (terrequinone A biosynthesis) gene cluster; transcriptionally regulated by LaeA	Acyl-CoA synthetase [I]
**AN8514.3**	**ANIA_08514**	**3.04**	tdiB	Asterriquinone prenyltransferase; member of the tdi gene cluster; required for terrequinone A production; catalyzes the reverse prenylation event during terrequinone A biosynthesis; lacks canonical prenyl diphosphate binding motif (D/N)DXXD	
**AN8523.3**	**ANIA_08523**	**4.52**		Ortholog of A. fumigatus Af293 : Afu5g09810, A. niger CBS 513.88 : An13g02380, Aspergillus wentii : Aspwe1_0170432 and Aspergillus sydowii : Aspsy1_0049687	
**AN8528.3**	**ANIA_08528**	**3.05**		Ortholog of A. nidulans FGSC A4 : AN12291, A. fumigatus Af293 : Afu5g09790, A. niger CBS 513.88 : An15g05030, A. oryzae RIB40 : AO090010000330 and Aspergillus wentii : Aspwe1_0054537, Aspwe1_0170434	
**AN8536.3**	**ANIA_08536**	**2.61**		Has domain(s) with predicted metal ion transmembrane transporter activity, role in metal ion transport, transmembrane transport and membrane localization	
**AN8537.3**	**ANIA_08537**	**3.40**		Ortholog of A. nidulans FGSC A4 : AN6683, AN5260, A. fumigatus Af293 : Afu4g06200 and A. niger CBS 513.88 : An12g04370, An11g06510, An14g01260	Flavin-containing monooxygenase [Q]
**AN8540.3**	**ANIA_08540**	**5.66**	mirB	Siderophore iron transporter	Predicted transporter (major facilitator superfamily) [R]
**AN8556.3**	**ANIA_08556**	**2.25**		Has domain(s) with predicted amino acid transmembrane transporter activity, role in amino acid transmembrane transport and membrane localization	Amino acid transporters [E]
**AN8568.3**	**ANIA_08568**	**2.07**		Protein of unknown function	
**AN8569.3**	**ANIA_08569**	**2.77**		Has domain(s) with predicted O-methyltransferase activity	Hydroxyindole-O-methyltransferase and related SAM-dependent methyltransferases [R]
**AN8602.3**	**ANIA_08602**	**2.70**		Ortholog of A. fumigatus Af293 : Afu2g00500, Afu6g09865, A. oryzae RIB40 : AO090023000371, Neosartorya fischeri NRRL 181 : NFIA_032690, NFIA_055590 and Aspergillus versicolor : Aspve1_0031494	
**AN8609.3**	**ANIA_08609**	**2.34**		Predicted glycosylphosphatidylinositol (GPI)-anchored protein	
**AN8611.3**	**ANIA_08611**	**5.85**		Has domain(s) with predicted catalytic activity and role in nucleoside metabolic process	
**AN8618.3**	**ANIA_08618**	**2.18**		Ortholog of A. nidulans FGSC A4 : AN6953, Aspergillus wentii : Aspwe1_0023054, Aspergillus versicolor : Aspve1_0063482, Aspve1_0137255 and Aspergillus clavatus NRRL 1 : ACLA_066490	
**AN8619.3**	**ANIA_08619**	**4.99**		Has domain(s) with predicted nucleotide binding activity	
**AN8624.3**	**ANIA_08624**	**2.86**		Protein of unknown function	
**AN8625.3**	**ANIA_08625**	**4.41**		Ortholog of A. nidulans FGSC A4 : AN8376, AN7265 and A. fumigatus Af293 : Afu6g00680, Afu8g06420	
**AN8683.3**	**ANIA_08683**	**3.64**		Ortholog(s) have ferric-chelate reductase activity, role in copper ion import, iron ion transport and plasma membrane localization	Ferric reductase, NADH/NADPH oxidase and related proteins [P, Q]
**AN8688.3**	**ANIA_08688**	**2.62**		ORF that was absent from the original release of version 4 of the A. nidulans annotation, but present in a previous version; reinstated into version 4 in AspGD as of July 2009	
**AN8692.3**	**ANIA_08692**	**2.14**	prxA	Thioredoxin-dependent peroxidase; intracellular; PRX5-like domain; highly similar to the allergen Aspf3 from related fungi; menadione stress-repressed protein; osmoadaptation-induced protein; repressed by starvation-induced autophagy	Alkyl hydroperoxide reductase/peroxiredoxin [O]
**AN8716.3**	**ANIA_08716**	**3.92**		Has domain(s) with predicted oxidoreductase activity and role in metabolic process	Kynurenine 3-monooxygenase and related flavoprotein monooxygenases [C, R]
**AN8727.3**	**ANIA_08727**	**2.20**		Ortholog of A. nidulans FGSC A4 : AN3303, AN3346, AN3348, AN2044 and A. fumigatus Af293 : Afu4g10080, Afu7g00420, Afu8g06100, Afu4g01242	
**AN8764.3**	**ANIA_08764**	**3.07**		Protein of unknown function; upregulated in A. oryzae and A. nidulans under hypoxic growth conditions	
**AN8775.3**	**ANIA_08775**	**11.51**		Ortholog of Neosartorya fischeri NRRL 181 : NFIA_004310	
**AN8780.3**	**ANIA_08780**	**2.24**		Ortholog(s) have serine-type endopeptidase activity, role in glycoprotein metabolic process and mitochondrion localization	Predicted metalloprotease with chaperone activity (RNAse H/HSP70 fold) [O]
**AN8781.3**	**ANIA_08781**	**6.50**		Ortholog of A. niger CBS 513.88 : An07g03030, Neosartorya fischeri NRRL 181 : NFIA_026420, Aspergillus wentii : Aspwe1_0285981 and Aspergillus versicolor : Aspve1_0141259	
**AN8797.3**	**ANIA_08797**	**2.93**	mvlA	Protein involved in regulation of a cryptic orsellinic acid gene cluster	
**AN8801.3**	**ANIA_08801**	**2.15**		Ortholog(s) have glyoxysome localization	
**AN8802.3**	**ANIA_08802**	**5.33**		Ortholog of Aspergillus versicolor : Aspve1_0088238 and Aspergillus sydowii : Aspsy1_0035041	
**AN8813.3**	**ANIA_08813**	**3.05**		Has domain(s) with predicted ATP binding, ATPase activity, coupled to transmembrane movement of substances activity, role in transport and integral to membrane localization	Pleiotropic drug resistance proteins (PDR1-15), ABC superfamily [Q]
**AN8826.3**	**ANIA_08826**	**2.18**		Ortholog of A. niger CBS 513.88 : An17g01855, Neosartorya fischeri NRRL 181 : NFIA_037140, Aspergillus wentii : Aspwe1_0034873 and Aspergillus versicolor : Aspve1_0056457	
**AN8830.3**	**ANIA_08830**	**2.16**	halA	Predicted protein kinase involved in halotolerance; suppressor of molybdate sensitivity of pacC mutant; mutants are sensitive to NaCl and have a moderate growth defect	Checkpoint kinase and related serine/threonine protein kinases [D]
**AN8893.3**	**ANIA_08893**	**2.01**		Has domain(s) with predicted nucleotide binding activity	
**AN8896.3**	**ANIA_08896**	**2.68**		Has domain(s) with predicted nucleotide binding, oxidoreductase activity and role in metabolic process	Hydroxysteroid 17-beta dehydrogenase 11 [Q]
**AN8905.3**	**ANIA_08905**	**4.25**	CYP537B1	Putative cytochrome P450	Cytochrome P450 CYP4/CYP19/CYP26 subfamilies [Q, I]
**AN8908.3**	**ANIA_08908**	**2.10**		Ortholog of A. fumigatus Af293 : Afu8g00630, A. niger CBS 513.88 : An07g00510, A. oryzae RIB40 : AO090009000111 and Aspergillus sydowii : Aspsy1_0162791, Aspsy1_0212146	
**AN8914.3**	**ANIA_08914**	**2.10**		Protein of unknown function	
**AN8928.3**	**ANIA_08928**	**3.92**	atrA	Putative plasma membrane ATP-binding cassette (ABC) transporter with a predicted role in multidrug resistance; transcript induced by the fungicide imazalil	Pleiotropic drug resistance proteins (PDR1-15), ABC superfamily [Q]
**AN8940.3**	**ANIA_08940**	**3.10**		Ortholog of Aspergillus terreus NIH2624 : ATET_03481	
**AN8955.3**	**ANIA_08955**	**3.32**		Has domain(s) with predicted role in transmembrane transport and integral to membrane localization	Permease of the major facilitator superfamily [G]
**AN8969.3**	**ANIA_08969**	**4.14**		Has domain(s) with predicted cation binding, lysozyme activity and role in carbohydrate metabolic process, cell wall macromolecule catabolic process, peptidoglycan catabolic process	
**AN8994.3**	**ANIA_08994**	**3.47**		Ortholog of A. oryzae RIB40 : AO090009000566, Aspergillus zonatus : Aspzo1_0127856, Aspergillus sydowii : Aspsy1_0062118 and Aspergillus carbonarius ITEM 5010 : Acar5010_398349	
**AN8995.3**	**ANIA_08995**	**6.48**		Has domain(s) with predicted role in transmembrane transport and integral to membrane localization	Monocarboxylate transporter [G]
**AN9001.3**	**ANIA_09001**	**7.14**		Protein of unknown function	
**AN9003.3**	**ANIA_09003**	**2.41**		Has domain(s) with predicted zinc ion binding activity	Ankyrin [M]
**AN9005.3**	**ANIA_09005**	**5.40**		Putative polyketide synthase (PKS); predicted backbone enzyme of a secondary metabolism gene cluster	Animal-type fatty acid synthase and related proteins [I]
**AN9006.3**	**ANIA_09006**	**4.12**		Protein of unknown function; predicted secondary metabolism gene cluster member	
**AN9010.3**	**ANIA_09010**	**4.03**		Ortholog(s) have nicotinamide mononucleotide transmembrane transporter activity, role in nicotinamide mononucleotide transport and mitochondrion localization	Permease of the major facilitator superfamily [G]
**AN9011.3**	**ANIA_09011**	**4.49**		Putative aryl-alcohol oxidase-related protein	Glucose dehydrogenase/choline dehydrogenase/mandelonitrile lyase (GMC oxidoreductase family) [R]

**AN9023.3**	**ANIA_09023**	**2.08**			Has domain(s) with predicted oxidoreductase activity and role in oxidation-reduction process	Ferric reductase, NADH/NADPH oxidase and related proteins [P, Q]
**AN9026.3**	**ANIA_09026**	**3.07**			Has domain(s) with predicted DNA binding, sequence-specific DNA binding RNA polymerase II transcription factor activity, zinc ion binding activity and role in regulation of transcription, DNA-dependent, transcription, DNA-templated	
**AN9044.3**	**ANIA_09044**	**2.10**			Has domain(s) with predicted FMN binding, oxidoreductase activity and role in oxidation-reduction process	NADH:flavin oxidoreductase/12-oxophytodienoate reductase [C, R]
**AN9046.3**	**ANIA_09046**	**2.05**		Transcription factor	Predicted DDE1 transposon-related ORF	
**AN9069.3**	**ANIA_09069**	**6.84**			Ortholog of A. fumigatus Af293 : Afu7g02500, A. niger CBS 513.88 : An12g00100, A. oryzae RIB40 : AO090038000626, Aspergillus wentii : Aspwe1_0136265 and Aspergillus sydowii : Aspsy1_0061378	
**AN9072.3**	**ANIA_09072**	**2.11**	rabF		Ortholog(s) have cytosol, nucleus localization	GTPase Rab4, small G protein superfamily [U]
**AN9076.3**	**ANIA_09076**	**2.10**			Transcript repressed by light in developmentally competent mycelia	
**AN9128.3**	**ANIA_09128**	**3.28**			Has domain(s) with predicted catalytic activity and role in metabolic process	Enoyl-CoA hydratase [I]
**AN9139.3**	**ANIA_09139**	**3.35**			Protein of unknown function	
**AN9142.3**	**ANIA_09142**	**5.90**			Ortholog of A. fumigatus Af293 : Afu7g01570, Aspergillus wentii : Aspwe1_0029597, Aspergillus sydowii : Aspsy1_0093879 and Aspergillus terreus NIH2624 : ATET_01773	
**AN9152.3**	**ANIA_09152**	**2.19**			Has domain(s) with predicted catalytic activity, coenzyme binding, nucleotide binding activity and role in cellular metabolic process	
**AN9159.3**	**ANIA_09159**	**2.41**			Ortholog of A. nidulans FGSC A4 : AN7899/dbaE, AN2031, AN0529, AN1032/afoC, A. fumigatus Af293 : Afu8g00910 and A. niger CBS 513.88 : An03g01180, An09g01790, An03g01810	Phospholipase/carboxyhydrolase [E]
**AN9165.3**	**ANIA_09165**	**4.47**			Has domain(s) with predicted role in transmembrane transport and integral to membrane localization	Sugar transporter/spinster transmembrane protein [G]
**AN9174.3**	**ANIA_09174**	**3.49**			Has domain(s) with predicted amino acid transmembrane transporter activity, role in amino acid transmembrane transport and membrane localization	Amino acid transporters [E]
**AN9193.3**	**ANIA_09193**	**3.45**	llmJ		Putative LaeA-like methyltransferase	
**AN9197.3**	**ANIA_09197**	**4.40**			Ortholog of A. fumigatus Af293 : Afu1g16030, Afu5g06880, Afu5g14930, A. niger CBS 513.88 : An07g06380, An12g03140, Neosartorya fischeri NRRL 181 : NFIA_036250 and Aspergillus wentii : Aspwe1_0043988	
**AN9202.3**	**ANIA_09202**	**2.29**			Ortholog of Aspergillus glaucus : Aspgl1_0032628 and Aspergillus zonatus : Aspzo1_0078085	
**AN9206.3**	**ANIA_09206**	**2.90**			Transcript induced in response to calcium dichloride in a CrzA-dependent manner	
**AN9207.3**	**ANIA_09207**	**3.32**			Has domain(s) with predicted RNA binding, endoribonuclease activity	
**AN9210.3**	**ANIA_09210**	**2.12**	CYP662A1		Putative cytochrome P450	Cytochrome P450 CYP4/CYP19/CYP26 subfamilies [Q, I]
**AN9219.3**	**ANIA_09219**	**4.33**			Has domain(s) with predicted role in transmembrane transport and integral to membrane localization	Predicted transporter (major facilitator superfamily) [R]
**AN9220.3**	**ANIA_09220**	**11.73**			Ortholog of A. fumigatus Af293 : Afu5g10320, A. oryzae RIB40 : AO090001000034, Neosartorya fischeri NRRL 181 : NFIA_076940 and Aspergillus versicolor : Aspve1_0038692	
**AN9243.3**	**ANIA_09243**	**2.31**			Has domain(s) with predicted catalytic activity and role in metabolic process	Acyl-CoA synthetase [I]
**AN9252.3**	**ANIA_09252**	**4.13**			Ortholog of Aspergillus glaucus : Aspgl1_0083762, Neosartorya fischeri NRRL 181 : NFIA_101490, NFIA_101510, Aspergillus wentii : Aspwe1_0735355 and Aspergillus versicolor : Aspve1_0046577	
**AN9259.3**	**ANIA_09259**	**2.11**	ausN		Predicted prenyltransferase; required for austinol and dehydroaustinol biosynthesis; aus secondary metabolism gene cluster member	Para-hydroxybenzoate-polyprenyl transferase [H]
**AN9282.3**	**ANIA_09282**	**3.22**			Has domain(s) with predicted role in oxidation-reduction process	Flavin-containing monooxygenase [Q]
**AN9288.3**	**ANIA_09288**	**2.26**			Ortholog(s) have cytoplasm, nucleus localization	Sorbitol dehydrogenase [Q]
**AN9299.3**	**ANIA_09299**	**9.37**			Ortholog of Aspergillus glaucus : Aspgl1_0045484, Aspergillus versicolor : Aspve1_0045065, Aspve1_0176243, Aspergillus zonatus : Aspzo1_0054543 and Aspergillus sydowii : Aspsy1_0047825, Aspsy1_0208145	Glutathione S-transferase [O]
**AN9303.3**	**ANIA_09303**	**2.27**			Ortholog of A. nidulans FGSC A4 : AN1597, AN2561, A. fumigatus Af293 : Afu3g03290, Afu3g13140, Afu3g15010, Afu3g03295 and A. niger CBS 513.88 : An17g00490, An11g08500, An09g01100, An08g09870, An07g06460	
**AN9304.3**	**ANIA_09304**	**2.11**			Glutathione S-transferase; upregulated in A. oryzae and A. nidulans under hypoxic growth conditions	Glutathione S-transferase [O]
**AN9305.3**	**ANIA_09305**	**3.36**			Has domain(s) with predicted transferase activity, transferring acyl groups other than amino-acyl groups activity	
**AN9313.3**	**ANIA_09313**	**2.22**	CYP58D1		Putative cytochrome P450; secondary metabolism gene cluster member with AN9314	Cytochrome P450 CYP3/CYP5/CYP6/CYP9 subfamilies [Q]
**AN9314.3**	**ANIA_09314**	**2.91**			Protein with homology to entkaurene synthases; prediction backbone enzyme of a secondary metabolite biosynthesis gene cluster	
**AN9324.3**	**ANIA_09324**	**2.80**			Has domain(s) with predicted carbon-nitrogen ligase activity, with glutamine as amido-N-donor activity	Amidases [J]
**AN9339.3**	**ANIA_09339**	**2.83**	catB		Hyphal catalase with a predicted role in gluconic acid and gluconate metabolism	Catalase [P]
**AN9342.3**	**ANIA_09342**	**4.32**			Has domain(s) with predicted ATP binding, ATPase activity, coupled to transmembrane movement of substances activity, role in transmembrane transport and integral to membrane localization	Multidrug/pheromone exporter, ABC superfamily [Q]
**AN9373.3**	**ANIA_09373**	**2.02**			Has domain(s) with predicted sequence-specific DNA binding RNA polymerase II transcription factor activity, zinc ion binding activity, role in regulation of transcription, DNA-dependent and nucleus localization	
**AN9377.3**	**ANIA_09377**	**6.30**			Ortholog of A. fumigatus Af293 : Afu3g02940, A. niger CBS 513.88 : An01g00550, A. oryzae RIB40 : AO090120000006, Aspergillus wentii : Aspwe1_0112345 and Aspergillus sydowii : Aspsy1_0163833	
**AN9380.3**	**ANIA_09380**	**16.84**			Putative chitin deacetylase; catalyzes the conversion of chitin to chitosan by the deacetylation of N-acetyl-D-glucosamine residues	
**AN9390.3**	**ANIA_09390**	**11.33**	chiC		Putative endochitinase; glycoside hydrolase family 18 (GH18) protein with a predicted role in chitin hydrolysis	Chitinase [G]
**AN9392.3**	**ANIA_09392**	**2.41**			Has domain(s) with predicted role in transmembrane transport and integral to membrane localization	Permease of the major facilitator superfamily [G]
**AN9393.3**	**ANIA_09393**	**2.26**			Ortholog(s) have fungal-type vacuole membrane localization	Permease of the major facilitator superfamily [R]
**AN9397.3**	**ANIA_09397**	**2.46**	hacA	Transcription factor	Putative basic leucine zipper (bZIP) transcription factor that regulates the unfolded protein response; hacA mRNA expression increased in the presence of farnesol	
**AN9450.3**	**ANIA_09450**	**2.87**			Predicted glycosylphosphatidylinositol (GPI)-anchored protein	
**AN9456.3**	**ANIA_09456**	**2.07**			Has domain(s) with predicted role in transmembrane transport and integral to membrane localization	Permease of the major facilitator superfamily [G]
**AN9485.3**	**ANIA_09485**	**2.29**			Ortholog of A. fumigatus Af293 : Afu3g06570, A. niger CBS 513.88 : An11g09750, Aspergillus wentii : Aspwe1_0029351, Aspergillus sydowii : Aspsy1_0087842 and Aspergillus terreus NIH2624 : ATET_08761	
**AN9489.3**	**ANIA_09489**	**2.78**			Protein of unknown function	
**AN9492.3**	**ANIA_09492**	**2.27**	amdX	Transcription factor	C2H2 zinc-finger transcription factor involved in regulation of acetamide catabolism; regulates amdS transcription	C2H2-type Zn-finger protein [K]
**AN9505.3**	**ANIA_09505**	**2.02**		Transcription factor	Predicted DDE1 transposon-related ORF	
**AN9513.3**	**ANIA_09513**	**3.73**			Has domain(s) with predicted transferase activity, transferring acyl groups other than amino-acyl groups activity	

AN9529.3	ANIA_09529	2.39		Ortholog of A. fumigatus Af293 : Afu6g04410, A. niger CBS 513.88 : An15g00970, Neosartorya fischeri NRRL 181 : NFIA_050950 and Aspergillus wentii : Aspwe1_0024387	
AN10011.3	ANIA_10011	2.39		Has domain(s) with predicted oxidoreductase activity, acting on the aldehyde or oxo group of donors, NAD or NADP as acceptor activity and role in oxidation-reduction process	Aldehyde dehydrogenase [C]
AN10043.3	ANIA_10043	2.01		Has domain(s) with predicted ATP binding, DNA binding, helicase activity	Chromatin remodeling complex WSTF-ISWI, small subunit [K]
AN10059.3	ANIA_10059	2.02		Putative Zn(II)C6 domain containing transcription factor; predicted NirA binding site in promoter; transcript repressed by nitrate	
AN10071.3	ANIA_10071	3.72		Has domain(s) with predicted RNA binding activity	Translational repressor MPT5/PUF4 and related RNA-binding proteins (Puf superfamily) [J]
AN10082.3	ANIA_10082	2.50	srpkF	Serine/threonine kinase domain protein; transcript repressed by nitrate	Serine/threonine protein kinase [T]
AN10096.3	ANIA_10096	2.48		Has domain(s) with predicted hydrolase activity	
AN10133.3	ANIA_10133	2.65		Ortholog of A. fumigatus Af293 : Afu1g15380, A. niger CBS 513.88 : An01g13650, Aspergillus wentii : Aspwe1_0482639 and Aspergillus sydowii : Aspsy1_0191721	
AN10139.3	ANIA_10139	2.91		Protein expressed at increased levels in a hapX mutant versus wild-type	Filamentous baseplate protein Ligatin, contains PUA domain [J]
AN10141.3	ANIA_10141	2.61		Has domain(s) with predicted protein tyrosine/serine/threonine phosphatase activity and role in protein dephosphorylation	
AN10154.3	ANIA_10154	3.68		Has domain(s) with predicted DNA binding activity	
AN10156.3	ANIA_10156	2.10		Has domain(s) with predicted kinase activity and role in glycerophospholipid biosynthetic process	Ethanolamine kinase [I]
AN10160.3	ANIA_10160	2.24		Ortholog of A. nidulans FGSC A4 : AN2370, AN0323, A. fumigatus Af293 : Afu1g02440, Afu4g02760, A. niger CBS 513.88 : An01g05750, An12g09350, An13g03290 and A. oryzae RIB40 : AO090020000719, AO090005000852	Uncharacterized conserved protein [S]
AN10188.3	ANIA_10188	3.98		Putative guanylate kinase; expression reduced after exposure to farnesol; transcript is induced by nitrate; predicted NirA binding site in promoter	Guanylate kinase [F]
AN10199.3	ANIA_10199	2.41		Ortholog of Aspergillus versicolor : Aspve1_0037245 and Aspergillus sydowii : Aspsy1_0028755	
AN10220.3	ANIA_10220	3.32	ccp1	Putatice cytochrome c peroxidase; intracellular; protein abundance decreased by menadione stress	
AN10221.3	ANIA_10221	2.32		Has domain(s) with predicted transporter activity, role in transmembrane transport and integral to membrane, plasma membrane localization	Permease of the major facilitator superfamily [R]
AN10252.3	ANIA_10252	2.87		Ortholog of A. fumigatus Af293 : Afu4g10320, A. niger CBS 513.88 : An04g06600, A. oryzae RIB40 : AO090003001189, Aspergillus wentii : Aspwe1_0166765 and Aspergillus sydowii : Aspsy1_0084447	
AN10261.3	ANIA_10261	2.32		Ortholog(s) have role in syncytium formation by plasma membrane fusion	
AN10267.3	ANIA_10267	2.31		Has domain(s) with predicted role in transmembrane transport and integral to membrane localization	Permease of the major facilitator superfamily [G]
AN10285.3	ANIA_10285	2.58		Ortholog of A. fumigatus Af293 : Afu5g10660, A. niger CBS 513.88 : An14g04040, A. oryzae RIB40 : AO090010000472, Aspergillus wentii : Aspwe1_0050403 and Aspergillus terreus NIH2624 : ATET_07619	
AN10310.3	ANIA_10310	4.19		Protein of unknown function	
AN10326.3	ANIA_10326	5.80		Ortholog of Aspergillus versicolor : Aspve1_0081330 and Aspergillus sydowii : Aspsy1_0148173	
AN10342.3	ANIA_10342	2.11		Possible pseudogene	
AN10358.3	ANIA_10358	3.79		Has domain(s) with predicted nucleotide binding, oxidoreductase activity, zinc ion binding activity and role in oxidation-reduction process	Alcohol dehydrogenase, class V [Q]
AN10390.3	ANIA_10390	3.27		Ortholog of A. nidulans FGSC A4 : AN3286, A. fumigatus Af293 : Afu4g01280, Afu5g00830, A. niger CBS 513.88 : An12g00980, An11g04090, An06g00150 and A. oryzae RIB40 : AO090138000050, AO090010000179	
AN10392.3	ANIA_10392	3.47		Has domain(s) with predicted UDP-N-acetylmuramate dehydrogenase activity, flavin adenine dinucleotide binding activity and role in oxidation-reduction process	Proteins containing the FAD binding domain [C]
AN10399.3	ANIA_10399	4.73		Has domain(s) with predicted nucleotide binding, oxidoreductase activity and role in metabolic process	1-Acyl dihydroxyacetone phosphate reductase and related dehydrogenases [Q]
AN10403.3	ANIA_10403	2.17		Ortholog of Aspergillus sydowii : Aspsy1_0087592	
AN10415.3	ANIA_10415	2.02		Ortholog(s) have protein heterodimerization activity, role in DNA repair and Smc5-Smc6 complex, nucleus localization	DNA repair protein RAD18 (SMC family protein) [L]
AN10440.3	ANIA_10440	2.15		Ortholog(s) have membrane, mitochondrion localization	NADH:ubiquinone oxidoreductase, NDUFA9/39kDa subunit [C]
AN10442.3	ANIA_10442	2.65		Predicted guanine nucleotide exchange factor	Invasion-inducing protein TIAM1/CDC24 and related RhoGEF GTPases [T]
AN10448.3	ANIA_10448	3.15	tprA	Tetratricopeptide repeat (TPR)-containing protein; usvB-dependent transcript induction in response to camptothecin	Kinesin light chain [Z]
AN10461.3	ANIA_10461	3.39		Protein of unknown function	
AN10473.3	ANIA_10473	7.30		Protein of unknown function	
AN10479.3	ANIA_10479	2.08	CYP540B4	Putative cytochrome P450	Cytochrome P450 CYP4/CYP19/CYP26 subfamilies [Q, I]
AN10482.3	ANIA_10482	2.62	bglF	Putative beta-glucosidase	
AN10487.3	ANIA_10487	3.91		Has domain(s) with predicted role in transmembrane transport and integral to membrane localization	Permease of the major facilitator superfamily [G]
AN10509.3	ANIA_10509	2.71		Protein of unknown function	
AN10513.3	ANIA_10513	3.23		Has domain(s) with predicted calcium-dependent cysteine-type endopeptidase activity, role in proteolysis and intracellular localization	Cytosolic Ca2+-dependent cysteine protease (calpain), large subunit (EF-Hand protein superfamily) [O, T]
AN10518.3	ANIA_10518	2.30		Protein of unknown function; protein induced by farnesol	
AN10558.3	ANIA_10558	2.37		Ortholog of A. fumigatus Af293 : Afu4g07280/sok1, A. niger CBS 513.88 : An04g01670, A. oryzae RIB40 : AO090023000845, Aspergillus wentii : Aspwe1_0023238 and Aspergillus terreus NIH2624 : ATET_05483	SOK1 kinase belonging to the STE20/SPS1/GC kinase family [T]
AN10575.3	ANIA_10575	3.97		Predicted glycosylphosphatidylinositol (GPI)-anchored protein	
AN10580.3	ANIA_10580	2.31		Ortholog of A. fumigatus Af293 : Afu5g08850, Neosartorya fischeri NRRL 181 : NFIA_078450, Aspergillus wentii : Aspwe1_0278501 and Aspergillus clavatus NRRL 1 : ACLA_011690	
AN10581.3	ANIA_10581	4.24		Ortholog of A. fumigatus Af293 : Afu5g08640, A. niger CBS 513.88 : An07g04900, Neosartorya fischeri NRRL 181 : NFIA_078640 and Aspergillus versicolor : Aspve1_0042746	
AN10588.3	ANIA_10588	4.05		Ortholog of A. fumigatus Af293 : Afu5g08870, A. niger CBS 513.88 : An07g04510, A. oryzae RIB40 : AO090020000457, AO090012000441 and Neosartorya fischeri NRRL 181 : NFIA_078440	
AN10592.3	ANIA_10592	10.51		Ortholog of A. fumigatus Af293 : Afu5g08730, A. oryzae RIB40 : AO090020000434, Neosartorya fischeri NRRL 181 : NFIA_078560 and Aspergillus versicolor : Aspve1_0134596	
AN10598.3	ANIA_10598	2.17		Has domain(s) with predicted calcium ion binding, calcium-dependent phospholipid binding activity	Annexin [U]
AN10603.3	ANIA_10603	2.14		Ortholog of A. fumigatus Af293 : Afu3g07510, A. niger CBS 513.88 : An02g13190, A. oryzae RIB40 : AO090020000145, Neosartorya fischeri NRRL 181 : NFIA_069680 and Aspergillus wentii : Aspwe1_0029255	
AN10604.3	ANIA_10604	3.74		Ortholog of A. fumigatus Af293 : Afu2g17760, A. niger CBS 513.88 : An03g02870, Neosartorya fischeri NRRL 181 : NFIA_093160, Aspergillus wentii : Aspwe1_0141692 and Aspergillus versicolor : Aspve1_0070749	Glucose-6-phosphate/phosphate and phosphoenolpyruvate/phosphate antiporter [G, E]
AN10641.3	ANIA_10641	2.39		Has domain(s) with predicted protein disulfide oxidoreductase activity	

AN10643.3	ANIA_10643	2.57		Has domain(s) with predicted protein heterodimerization activity, sequence-specific DNA binding activity and intracellular localization	
AN10650.3	ANIA_10650	3.60		Ortholog of A. nidulans FGSC A4 : AN11203, A. fumigatus Af293 : Afu4g00520, A. niger CBS 513.88 : An12g05740 and Aspergillus wentii : Aspwe1_0072865, Aspwe1_0156452	
AN10662.3	ANIA_10662	4.72		Has domain(s) with predicted role in transmembrane transport and integral to membrane localization	Permease of the major facilitator superfamily [G]
AN10663.3	ANIA_10663	2.32		Has domain(s) with predicted hydrolase activity and role in nucleotide catabolic process	
AN10677.3	ANIA_10677	7.20		Ortholog(s) have role in DNA repair, ascospore formation, meiotic gene conversion, reciprocal meiotic recombination and nucleus, spindle localization	DNA repair protein, SNF2 family [L]
AN10700.3	ANIA_10700	2.02		Ortholog of S. cerevisiae : YER010C, A. fumigatus Af293 : Afu4g10940, A. niger CBS 513.88 : An04g05730, A. oryzae RIB40 : AO090003001122 and Aspergillus wentii : Aspwe1_0166704	
AN10733.3	ANIA_10733	2.05		Ortholog of A. fumigatus Af293 : Afu2g07650, Aspergillus glaucus : Aspgl1_0045891, Neosartorya fischeri NRRL 181 : NFIA_083380 and Aspergillus wentii : Aspwe1_0632268	
AN10736.3	ANIA_10736	4.36		Protein of unknown function	
AN10737.3	ANIA_10737	2.10		Ortholog of A. fumigatus Af293 : Afu2g07490, A. niger CBS 513.88 : An05g00040, Aspergillus wentii : Aspwe1_0175101, Aspergillus sydowii : Aspsy1_0044731 and Aspergillus terreus NIH2624 : ATET_06858	
AN10748.3	ANIA_10748	6.11		Protein of unknown function	
AN10752.3	ANIA_10752	5.57		Ortholog of A. fumigatus Af293 : Afu2g11470, A. niger CBS 513.88 : An02g03450, Neosartorya fischeri NRRL 181 : NFIA_086710, Aspergillus wentii : Aspwe1_0044288 and Aspergillus versicolor : Aspve1_0040617	
AN10753.3	ANIA_10753	3.50		Ortholog(s) have phosphoribosylglycinamide formyltransferase activity, role in 'de novo' IMP biosynthetic process, adenine biosynthetic process and cytoplasm, nucleus localization	5'-phosphoribosylglycinamide formyltransferase [G]
AN10773.3	ANIA_10773	2.01		Protein of unknown function	
AN10805.3	ANIA_10805	3.20		Has domain(s) with predicted hydrolase activity and role in metabolic process	Hormone-sensitive lipase HSL [I]
AN10814.3	ANIA_10814	2.14		Has domain(s) with predicted oxidoreductase activity and role in metabolic process	
AN10845.3	ANIA_10845	2.07		Ortholog(s) have plasma membrane localization	Predicted transporter (major facilitator superfamily) [R]
AN10863.3	ANIA_10863	3.03		Has domain(s) with predicted nucleotide binding, oxidoreductase activity, acting on the CH-NH2 group of donors, oxygen as acceptor activity and role in oxidation-reduction process	D-aspartate oxidase [E]
AN10869.3	ANIA_10869	3.89	ffkE	Has domain(s) with predicted ATP binding, protein kinase activity and role in protein phosphorylation	Serine/threonine protein kinase [T]
AN10876.3	ANIA_10876	2.47		Has domain(s) with predicted cation transmembrane transporter activity, role in cation transport, transmembrane transport and integral to membrane localization	Zn2+ transporter ZNT1 and related Cd2+/Zn2+ transporters (cation diffusion facilitator superfamily) [P]
AN10887.3	ANIA_10887	2.35	CYP5095B1	Putative cytochrome P450; predicted secondary metabolism gene cluster member	Cytochrome P450 CYP11/CYP12/CYP24/CYP27 subfamilies [Q]
AN10889.3	ANIA_10889	2.62		Predicted O-methyltransferase; predicted secondary metabolism gene cluster member; coregulated with the PKS pkgA/AN7071	Hydroxyindole-O-methyltransferase and related SAM-dependent methyltransferases [R]
AN10903.3	ANIA_10903	2.36		Ortholog(s) have guanine deaminase activity and cytosol localization	Atrazine chlorohydrolase/guanine deaminase [F, Q]
AN10909.3	ANIA_10909	3.57		Has domain(s) with predicted trans-aconitate 2-methyltransferase activity	
AN10945.3	ANIA_10945	2.96		Ortholog of A. fumigatus Af293 : Afu6g09950, A. niger CBS 513.88 : An11g08010, A. oryzae RIB40 : AO090001000628, Aspergillus wentii : Aspwe1_0121304 and Aspergillus sydowii : Aspsy1_0058487	Splicing factor SPF30 [A]
AN10949.3	ANIA_10949	2.23		Ortholog(s) have ATP binding, drug binding, fluconazole transporter activity, phospholipid-translocating ATPase activity, xenobiotic-transporting ATPase activity	Pleiotropic drug resistance proteins (PDR1-15), ABC superfamily [Q]
AN10951.3	ANIA_10951	2.55		Protein of unknown function; transcript repressed by nitrate	Synaptic vesicle transporter SVOP and related transporters (major facilitator superfamily) [R]
AN10960.3	ANIA_10960	3.98		Ortholog of Aspergillus versicolor : Aspve1_0135933	
AN10964.3	ANIA_10964	3.55		Has domain(s) with predicted methyltransferase activity and role in metabolic process	
AN10989.3	ANIA_10989	2.61		Ortholog of A. fumigatus Af293 : Afu2g01110, A. niger CBS 513.88 : An10g00450, A. oryzae RIB40 : AO090701000379, Neosartorya fischeri NRRL 181 : NFIA_033410 and Aspergillus clavatus NRRL 1 : ACLA_094420	
AN11013.3	ANIA_11013	6.93	stcL	Putative p450 monooxygenase required for desaturation of bisfuran in the sterigmatocystin biosynthesis pathway; member of the sterigmatocystin biosynthesis gene cluster	Cytochrome P450 CYP3/CYP5/CYP6/CYP9 subfamilies [Q]
AN11015.3	ANIA_11015	2.06		Has domain(s) with predicted S-(hydroxymethyl)glutathione synthase activity, zinc ion binding activity and role in formaldehyde catabolic process	
AN11028.3	ANIA_11028	4.50		Predicted nucleotide binding protein with oxidoreductase activity and a role in oxidation-reduction; predicted secondary metabolism gene cluster member	Dehydrogenases with different specificities (related to short-chain alcohol dehydrogenases) [Q]
AN11030.3	ANIA_11030	4.12		Putative NADP-coupled glycerol dehydrogenase; transcript is induced by nitrate	Aldo/keto reductase family proteins [R]
AN11038.3	ANIA_11038	4.44		Has domain(s) with predicted carbohydrate binding, carbon-oxygen lyase activity and role in amino sugar catabolic process, carbohydrate metabolic process	
AN11046.3	ANIA_11046	2.68		Protein of unknown function; transcript is induced by nitrate	
AN11061.3	ANIA_11061	2.53		Has domain(s) with predicted transferase activity, transferring acyl groups other than amino-acyl groups activity	
AN11062.3	ANIA_11062	3.06		Ortholog(s) have role in mitochondrial genome maintenance and cytosol, mitochondrial matrix, nucleus localization	Putative translation initiation inhibitor UK114/IBM1 [J]
AN11065.3	ANIA_11065	2.08		Putative oxidosqualene-lanosterol cyclase with a predicted role in ergosterol metabolism	Oxidosqualene-lanosterol cyclase and related proteins [I]
AN11066.3	ANIA_11066	3.65		Putative RNA maturation protein and telomerase elongation inhibitor; ortholog of S. cerevisiae Pxr1p; expression reduced after exposure to farnesol	Telomerase elongation inhibitor/RNA maturation protein PINX1 [A, D]
AN11068.3	ANIA_11068	2.14		Ortholog of A. fumigatus Af293 : Afu5g03810, A. niger CBS 513.88 : An09g06360, Aspergillus wentii : Aspwe1_0602984, Aspergillus sydowii : Aspsy1_0094439 and Aspergillus terreus NIH2624 : ATET_08030	
AN11076.3	ANIA_11076	3.21		Ortholog of A. fumigatus Af293 : Afu3g02220, A. niger CBS 513.88 : An01g02020, Neosartorya fischeri NRRL 181 : NFIA_003350, Aspergillus wentii : Aspwe1_0171305 and Aspergillus versicolor : Aspve1_0056749	
AN11079.3	ANIA_11079	7.47		Protein of unknown function	
AN11080.3	ANIA_11080	2.61		Putative dimethyl-allyl-tryptophan synthase (DMATS)-type aromatic prenyltransferase	
AN11091.3	ANIA_11091	2.11		Predicted DDE1 transposon-related ORF	
AN11107.3	ANIA_11107	2.35		Ortholog(s) have mitochondrion localization	Magnesium transporters: CorA family [P]
AN11149.3	ANIA_11149	2.55		Has domain(s) with predicted sulfuric ester hydrolase activity and role in metabolic process	Sulfatases [G]
AN11153.3	ANIA_11153	2.27		Ortholog(s) have endoplasmic reticulum localization	Permease of the major facilitator superfamily [G]
AN11154.3	ANIA_11154	2.85		Ortholog of A. niger CBS 513.88 : An05g01490, A. oryzae RIB40 : AO090009000214, Neosartorya fischeri NRRL 181 : NFIA_084670, Aspergillus wentii : Aspwe1_0119954 and Aspergillus versicolor : Aspve1_0144019	
AN11158.3	ANIA_11158	4.47		Protein of unknown function	
AN11200.3	ANIA_11200	2.28		Ortholog of A. fumigatus Af293 : Afu5g10300, Neosartorya fischeri NRRL 181 : NFIA_076960, Aspergillus fumigatus A1163 : AFUB_057880 and Aspergillus sydowii : Aspsy1_0036837	

**AN11203.3**	**ANIA_11203**	**2.66**		Ortholog of A. nidulans FGSC A4 : AN10650, A. fumigatus Af293 : Afu4g00520, A. niger CBS 513.88 : An12g05740 and Aspergillus wentii : Aspwe1_0072865, Aspwe1_0156452	
**AN11205.3**	**ANIA_11205**	**2.15**	ausK	Predicted oxidoreductase; required for austinol and dehydroaustinol biosynthesis	Voltage-gated shaker-like K+ channel, subunit beta/KCNAB [C]
**AN11210.3**	**ANIA_11210**	**3.35**		Protein of unknown function; transcript repressed by nitrate	
**AN11215.3**	**ANIA_11215**	**8.88**		Has domain(s) with predicted metal ion transmembrane transporter activity, role in metal ion transport, transmembrane transport and membrane localization	
**AN11217.3**	**ANIA_11217**	**3.10**		Predicted transmembrane transporter; located in the aus secondary metabolism gene cluster	Predicted transporter (major facilitator superfamily) [R]
**AN11228.3**	**ANIA_11228**	**5.79**		Has domain(s) with predicted DNA binding, sequence-specific DNA binding RNA polymerase II transcription factor activity, zinc ion binding activity and role in regulation of transcription, DNA-dependent, transcription, DNA-templated	
**AN11252.3**	**ANIA_11252**	**3.80**		Ortholog(s) have 3-hydroxyanthranilate 3,4-dioxygenase activity, role in 'de novo' NAD biosynthetic process from tryptophan and cytoplasm localization	3-hydroxyanthranilate oxygenase HAAO [E]
**AN11344.3**	**ANIA_11344**	**3.23**		Ortholog(s) have microtubule binding activity, role in mitosis, mitotic spindle organization in nucleus, regulation of microtubule polymerization or depolymerization and DASH complex, spindle localization	
**AN11489.3**	**ANIA_11489**	**2.19**		Protein of unknown function; transcript is induced by nitrate	
**AN11505.3**	**ANIA_11505**	**3.24**		Protein of unknown function	
**AN11551.3**	**ANIA_11551**	**3.45**		Protein of unknown function	
**AN11584.3**	**ANIA_11584**	**4.09**	dbaC	YCII domain protein with a role in secondary metabolism; member of the dba gene cluster; dba cluster expression is coregulated	
**AN11641.3**	**ANIA_11641**	**2.05**		Ortholog of Aspergillus versicolor : Aspve1_0117193	

Reference

1. AspGD (from The Board of Trustees, Leland Stanford Junior University and the Broad Institute)

: Chromosomal Feature Files - Last Update dates 2014-03-02

2. Transcription factor (DBD): Wilson *et al* ., 2008. *Nucleic Acids Res.* 36, D88–D92

3. KOGs

: Tatusov et al., 2003. BMC Bioinformatics 2003, 4:41

: Original PEDANT database name: p3_p130_Asp_nidul (PEDANT 3) - Calculation dates 2007-08-11

4. PEDANT 3: Walter *et al* ., 2009. Nucleic Acids Res. 37, Database issue

: Original PEDANT database name: p3_r40559_Asp_nidul (PEDANT 3) - Calculation dates 2013-09-19

Supplementary Table 1.2. Genes exhibited more than twofold reduction in the *ΔvosA* mutant conidia

: 1,104 genes

Systematic ID		Expession Ratio (*ΔvosA* /WT)	Description				Cell wall (GO Category from 'PEDANT3 - p3_r40559_Asp_nidul'[*4])												Sporulation (GO from 'PEDANT3 - p3_r40559_Asp_nidul'[*4])			
			Gene Name (AspGD[*1])	Transcription factor (DBD[*2])	Description (AspGD[*1])	KOGs[*3] Description & Category (PEDANT 3)	Cellular components GO:0005618 cell wall	Biological Process GO:0042546 cell wall biogenesis	Biological Process GO:0044036 cell wall macromolecule metabolic process	Biological Process GO:0034406 cell wall beta-glucan metabolic process	Biological Process GO:0006075 1,3-beta-D-glucan biosynthetic process	Biological Process GO:0006037 cell wall chitin metabolic process	Cellular compoment GO:0009277 fungal-type cell wall	Biological Process GO:0031505 fungal-type cell wall organization	Biological Process GO:0009272 fungal-type cell wall biogenesis	Cellular components GO:0031160 spore wall	Biological Process GO:0070590 spore wall biogenesis	Cellular compoment GO:0030446 hyphal cell wall	Biological Process GO:0009272 sporulation	Biological Process GO:0030436 asexual sporulation	Biological Process GO:0030293 sexual sporulation	Biological Process GO:0075259 spore-bearing organ development
**AN2752.3**	**ANIA_02752**	**0.38**			Ortholog(s) have alpha-1,2-mannosyltransferase activity	Glycolipid 2-alpha-mannosyltransferase (alpha-1,2-mannosyltransferase) [G]		cell wall biogenesis	cell wall macromolecule metabolic process					fungal-type cell wall organization								
**AN0287.3**	**ANIA_00287**	**0.27**			Ortholog(s) have cytosol localization	WD40 repeat protein [R]		cell wall biogenesis	cell wall macromolecule metabolic process													
**AN1620.3**	**ANIA_01620**	**0.29**			Ortholog of A. nidulans FGSC A4 : AN2626, A. fumigatus Af293 : Afu8g02040/och3, A. niger CBS 513.88 : An03g01090/hocA, An05g02320 and A. oryzae RIB40 : AO090010000615			cell wall biogenesis	cell wall macromolecule metabolic process													
**AN2359.3**	**ANIA_02359**	**0.37**	xlnD		Beta-xylosidase, involved in degradation of xylans; transcriptionally induced by growth on xylose				cell wall macromolecule metabolic process													
**AN7847.3**	**ANIA_07847**	**0.46**			Has domain(s) with predicted role in cell wall macromolecule catabolic process				cell wall macromolecule metabolic process													
**AN9035.3**	**ANIA_09035**	**0.31**	aglG		Putative alpha-galactosidase with a predicted role in galactose and galactitol metabolism and in degradation of mannans				cell wall macromolecule metabolic process													
**AN1545.3**	**ANIA_01545**	**0.47**			Ortholog(s) have protein serine/threonine phosphatase activity	Serine/threonine protein phosphatase 2A, regulatory subunit [T]		cell wall biogenesis							fungal-type cell wall biogenesis							
**AN2968.3**	**ANIA_02968**	**0.42**	ippA		Putative inorganic diphosphatase with a predicted role in energy metabolism; intracellular; protein abundance decreased by menadione stress; protein expressed at increased levels during osmoadaptation	Inorganic pyrophosphatase/Nucleosome remodeling factor, subunit NURF38 [C]	cell wall						fungal-type cell wall					hyphal cell wall				
**AN6900.3**	**ANIA_06900**	**0.13**	tpiA		Putative triose-phosphate isomerase with a predicted role in gluconeogenesis and glycolysis; protein induced by farnesol	Triosephosphate isomerase [G]	cell wall						fungal-type cell wall									
**AN7916.3**	**ANIA_07916**	**0.31**			Putative amino acid transporter; adjacent to the F9775 secondary metabolite gene cluster	Amino acid transporters [E]	cell wall						fungal-type cell wall									
**AN2875.3**	**ANIA_02875**	**0.28**	fbaA		Putative fructose-bisphosphate aldolase with a predicted role in gluconeogenesis and glycolysis; intracellular, menadione stress-induced protein; palA-dependent expression independent of pH	Fructose 1,6-bisphosphate aldolase [G]	cell wall											hyphal cell wall				
**AN2977.3**	**ANIA_02977**	**0.42**			Putative transporter with a predicted role in small molecule transport; putative mitochondrial phosphate carrier protein; expression upregulated after exposure to farnesol	Mitochondrial phosphate carrier protein [C]	cell wall															
**AN4860.3**	**ANIA_04860**	**0.43**	pmeB		Putative pectin methyl esterase		cell wall															
**AN6679.3**	**ANIA_06679**	**0.49**			Ortholog(s) have SSU rRNA binding, structural constituent of ribosome activity and role in maturation of SSU-rRNA from tricistronic rRNA transcript (SSU-rRNA, 5.8S rRNA, LSU-rRNA)	40S ribosomal protein S13 [J]	cell wall															
**AN8581.3**	**ANIA_08581**	**0.26**			Putative laccase related protein	Multicopper oxidases [Q]	cell wall															
**AN11220.3**	**ANIA_11220**	**0.38**	CYP687A1		Putative cytochrome P450	Cytochrome P450 CYP2 subfamily [Q]	cell wall															
**AN1246.3**	**ANIA_01246**	**0.32**	pgkA		Putative phosphoglycerate kinase with a predicted role in gluconeogenesis and glycolysis; intracellular, menadione stress-induced protein; promoter activity is greater on gluconeogenic than on glycolytic carbon sources	3-phosphoglycerate kinase [G]							fungal-type cell wall					hyphal cell wall				
**AN1867.3**	**ANIA_01867**	**0.41**	phoB		Putative cyclin-dependent protein kinase; lethal in combination with mutations in phoA	Protein kinase PCTAIRE and related kinases [R]								fungal-type cell wall organization					sporulation	asexual sporulation		spore-bearing organ development
**AN6305.3**	**ANIA_06305**	**0.47**	pkaA		cAMP-dependent protein kinase catalytic subunit; cAMP signaling during conidial germination; overlapping functions with SchA; induced by osmotic stress; mutant growth defect remediated by sucrose and NaCl; reduced growth on AVICEL medium	cAMP-dependent protein kinase catalytic subunit (PKA) [T]								fungal-type cell wall organization					sporulation	asexual sporulation		
**AN4615.3**	**ANIA_04615**	**0.47**	surG		Transmembrane domain-containing protein with homology to S. cerevisiae Sur7p; SurG-GFP localizes perinuclearly and to patches in the periphery of spores and to the vacuole and endosomes of hyphae									fungal-type cell wall organization					sporulation		sexual sporulation	
**AN2740.3**	**ANIA_02740**	**0.48**	pdeB		High-affinity cAMP phosphodiesterase	Cyclic nucleotide phosphodiesterase [T]								fungal-type cell wall organization					sporulation			spore-bearing organ development
**AN10756.3**	**ANIA_10756**	**0.25**			Ortholog(s) have role in ascospore formation, mitotic G1 cell cycle arrest in response to nitrogen starvation, regulation of conjugation with cellular fusion and TORC2 complex, cell division site, cytosol, nucleus localization	Protein required for meiosis [D]								fungal-type cell wall organization								spore-bearing organ development
**AN3441.3**	**ANIA_03441**	**0.28**	orlA		Trehalose 6-phosphate phosphatase, predicted subunit of the trehalose-6-phosphate synthase/phosphatase complex; involved in chitin synthesis at elevated temperatures	Trehalose-6-phosphate synthase component TPS1 and related subunits [G]								fungal-type cell wall organization								
**AN6487.3**	**ANIA_06487**	**0.30**			Putative aspartyl protease; ortholog of S. cerevisiae BAR1; expression reduced after exposure to farnesol	Aspartyl protease [O]								fungal-type cell wall organization								
**AN8868.3**	**ANIA_08868**	**0.26**	rhbA		Ortholog(s) have GDP binding, GTP binding, GTPase activity	Ras-related GTPase [R]								fungal-type cell wall organization								
**AN5296.3**	**ANIA_05296**	**0.33**	tcsA		Two-component signaling protein with histidine kinase and response regulator domains; involved in control of conidiation	Sensory transduction histidine kinase [T]													sporulation	asexual sporulation	sexual sporulation	
**AN6197.3**	**ANIA_06197**	**0.49**	nudF		Nuclear migration protein; mutants defective in asexual and sexual sporulation; similar to human LIS-1; interacts directly with NudC; spindle-pole body-localized; may facilitate minus-end-directed dynein movement	WD40 repeat-containing protein [S]													sporulation	asexual sporulation	sexual sporulation	
**AN2094.3**	**ANIA_02094**	**0.41**	dopA		Putative leucine-zipper transcription factor, involved in conidiophore morphogenesis and sexual development	Dopey and related predicted leucine zipper K factors [K]													sporulation	asexual sporulation		spore-bearing organ development
**AN1876.3**	**ANIA_01876**	**0.49**			Ortholog(s) have role in cellular manganese ion homeostasis, mitochondrion organization and fungal-type vacuole membrane localization	Predicted membrane protein, contains two CBS domains [S]													sporulation	asexual sporulation		
**AN3144.3**	**ANIA_03144**	**0.11**	vps19		Protein with predicted role in Golgi to endosome transport, Golgi to vacuole transport, vacuole inheritance, vesicle docking involved in exocytosis and vesicle fusion; locus contains conserved upstream open reading frame (uORF) AN3144-uORF	FYVE finger-containing protein [R]													sporulation	asexual sporulation		
**AN4706.3**	**ANIA_04706**	**0.07**	myoB		Myosin II; required for normal conidiation, septum formation and for correct chitin deposition	Myosin class II heavy chain [Z]													sporulation	asexual sporulation		
**AN2458.3**	**ANIA_02458**	**0.48**	candA-C		C-terminal subunit of Cand1; which is split into two subunits (candA-N and candA-C) that bind to each other and to unneddylated cullin-RING complexes; role in sexual development, secondary metabolism and light control of asexual development	TATA-binding protein-interacting protein [R]													sporulation		sexual sporulation	spore-bearing organ development
**AN0243.3**	**ANIA_00243**	**0.35**			Putative phosphatidylinositol transfer protein	Phosphatidylinositol transfer protein SEC14 and related proteins [I]													sporulation		sexual sporulation	
**AN1527.3**	**ANIA_01527**	**0.35**			Ortholog(s) have role in ascospore formation and cytosol, fungal-type vacuole, nucleus localization														sporulation		sexual sporulation	
**AN3152.3**	**ANIA_03152**	**0.37**	nsdD		Predicted GATA-type zinc-finger transcription factor required for sexual development; transcript induced by nitrositive stress														sporulation		sexual sporulation	
**AN3522.3**	**ANIA_03522**	**0.17**	fhbB		Flavohemoprotein, involved (with FhbA) in sterigmatocystin production	Hemoglobin-like flavoprotein [C]													sporulation		sexual sporulation	
**AN3814.3**	**ANIA_03814**	**0.36**	cypA		Putative peptidyl-prolyl cis-trans isomerase (PPIase); cyclophilin	Peptidyl-prolyl cis-trans isomerase [O]													sporulation		sexual sporulation	
**AN5195.3**	**ANIA_05195**	**0.45**			Ortholog(s) have role in ER to Golgi vesicle-mediated transport, ascospore formation, axial cellular bud site selection and ER to Golgi transport vesicle, endoplasmic reticulum membrane localization	ER vesicle integral membrane protein involved in establishing cell polarity, signaling and protein degradation [O, U, T]													sporulation		sexual sporulation	
**AN5523.3**	**ANIA_05523**	**0.26**	tpsA		Putative alpha,alpha-trehalose-phosphate synthase (UDP-forming) with a role in trehalose biosynthesis; transcriptionally induced during spore germination and exponential growth; required for viability of conidia during prolonged storage	Trehalose-6-phosphate synthase component TPS1 and related subunits [G]													sporulation		sexual sporulation	
**AN8605.3**	**ANIA_08605**	**0.47**	cyp1		Putative peptidyl-prolyl cis-trans isomerase (PPIase); cyclophilin	HSP90 co-chaperone CPR7/Cyclophilin [O]													sporulation		sexual sporulation	
**AN10902.3**	**ANIA_10902**	**0.33**			Ortholog(s) have water channel activity and role in ascospore formation, cellular response to freezing, cellular response to osmotic stress, single-species biofilm formation on inanimate substrate, water transport	Aquaporin (major intrinsic protein family) [G]													sporulation		sexual sporulation	
**AN5643.3**	**ANIA_05643**	**0.40**			Ortholog(s) have DNA translocase activity, chromatin binding, single-stranded DNA binding activity	Chromatin remodeling complex WSTF-ISWI, small subunit [K]													sporulation			
**AN0387.3**	**ANIA_00387**	**0.48**	cryA		Cryptochrome, nuclearly-localized protein that senses UVA and blue light and represses sexual development by regulating other regulators such as VeA, NsdD, and RosA; member of the class I cyclobutane pyrimidine dimer (CPD) photolyase family	Deoxyribodipyrimidine photolyase/cryptochrome [L, T]																spore-bearing organ development
**AN1553.3**	**ANIA_01553**	**0.20**			Has domain(s) with predicted role in fruiting body development, hemolysis by symbiont of host erythrocytes																	spore-bearing organ development

**AN3449.3**	**ANIA_03449**	**0.48**		Ortholog(s) have ubiquitin-specific protease activity, role in protein deubiquitination, regulation of transcription, DNA-dependent and cytosol, nucleus localization	OTU-like cysteine protease [T, O]	spore-bearing organ development
**AN7743.3**	**ANIA_07743**	**0.41**	preA	Predicted G-protein coupled pheromone receptor; similar to S. cerevisiae Ste3; induced by growth on 1% glucose		spore-bearing organ development
**AN10788.3**	**ANIA_10788**	**0.21**		Ortholog(s) have ubiquitin-specific protease activity	Predicted signal transduction protein [R]	spore-bearing organ development
**AN0020.3**	**ANIA_00020**	**0.35**		Ortholog of A. fumigatus Af293 : Afu2g00190, A. niger CBS 513.88 : An15g07480, Aspergillus versicolor : Aspve1_0365574, Aspergillus niger ATCC 1015 : 40853-mRNA and Aspergillus zonatus : Aspzo1_0077270		
**AN0027.3**	**ANIA_00027**	**0.38**		Ortholog of A. nidulans FGSC A4 : AN12088, A. fumigatus Af293 : Afu2g09400, A. niger CBS 513.88 : An12g05000 and A. oryzae RIB40 : AO090011000294, AO090026000051, AO090011000747, AO090011000330	Flavin-containing monooxygenase [Q]	
**AN0040.3**	**ANIA_00040**	**0.45**		Ortholog(s) have 3'-5' exonuclease activity, DNA-directed DNA polymerase activity, role in mitochondrial DNA replication and mitochondrion localization	Mitochondrial DNA polymerase gamma, catalytic subunit [L]	
**AN0041.3**	**ANIA_00041**	**0.34**		Protein of unknown function		
**AN0045.3**	**ANIA_00045**	**0.24**		Transcript induced by light in in developmentally competent mycelia		
**AN0070.3**	**ANIA_00070**	**0.43**	uaW	2-oxo-4-hydroxy-4-carboxy-5-ureidoimidazoline (OHCU) decarboxylase; involved in purine utilization; inducible by uric acid and repressible by ammonium; under the control of the UaY protein		
**AN0094.3**	**ANIA_00094**	**0.24**		Has domain(s) with predicted DNA binding, zinc ion binding activity, role in transcription, DNA-templated and nucleus localization		
**AN0097.3**	**ANIA_00097**	**0.49**		Ortholog(s) have role in DNA recombination, double-strand break repair via nonhomologous end joining	ATP-dependent DNA ligase IV [L]	
**AN0120.3**	**ANIA_00120**	**0.31**	tapA	Putative SitA phosphatase inhibitor protein; involved in the TOR signaling pathway	Protein phosphatase 2A-associated protein [T]	
**AN0121.3**	**ANIA_00121**	**0.47**	hemC	porphobilinogen deaminase; heme biosynthesis enzyme that facilitates growth in reactive nitrogen species conditions; transcriptionally induced by reactive nitrogen species	Porphobilinogen deaminase [H]	
**AN0126.3**	**ANIA_00126**	**0.45**		Ortholog(s) have ATP binding, ATPase activity, dinucleotide insertion or deletion binding, loop DNA binding, single-stranded DNA binding activity	DNA mismatch repair protein - MLH1 family [L]	
**AN0131.3**	**ANIA_00131**	**0.40**		Ortholog(s) have Golgi apparatus, endoplasmic reticulum localization		
**AN0132.3**	**ANIA_00132**	**0.49**		Ortholog of A. fumigatus Af293 : Afu5g11630, A. niger CBS 513.88 : An18g03110, A. oryzae RIB40 : AO090026000663, Aspergillus wentii : Aspwe1_0170695 and Aspergillus sydowii : Aspsy1_0195219		
**AN0140.3**	**ANIA_00140**	**0.47**		Protein with a predicted role in actin assembly; similar to Saccharomyces cerevisiae Arp3p	Actin-related protein Arp2/3 complex, subunit Arp3 [Z]	
**AN0166.3**	**ANIA_00166**	**0.26**		Ortholog(s) have protein homodimerization activity and role in attachment of spindle microtubules to kinetochore involved in homologous chromosome segregation, mitotic sister chromatid segregation, mitotic spindle assembly checkpoint	Centromere-associated protein NUF2 [D]	
**AN0173.3**	**ANIA_00173**	**0.43**		Ortholog of A. niger CBS 513.88 : An01g02440, A. oryzae RIB40 : AO090026000850, Aspergillus wentii : Aspwe1_0442639, Aspergillus sydowii : Aspsy1_0027713 and Aspergillus terreus NIH2624 : ATET_07718		
**AN0174.3**	**ANIA_00174**	**0.23**		Ortholog(s) have D-amino-acid oxidase activity and role in D-alanine metabolic process, D-valine metabolic process	D-aspartate oxidase [E]	
**AN0178.3**	**ANIA_00178**	**0.26**		Protein of unknown function		
**AN0184.3**	**ANIA_00184**	**0.34**		Ortholog(s) have Arp2/3 complex binding activity, role in actin cortical patch assembly, actin filament debranching, negative regulation of Arp2/3 complex-mediated actin nucleation and actin cortical patch, cytosol, nucleus localization	Glia maturation factor beta [W]	
**AN0197.3**	**ANIA_00197**	**0.17**		Has domain(s) with predicted sequence-specific DNA binding RNA polymerase II transcription factor activity, zinc ion binding activity, role in regulation of transcription, DNA-dependent and nucleus localization		
**AN0198.3**	**ANIA_00198**	**0.44**		Ortholog of A. fumigatus Af293 : Afu5g14750, A. niger CBS 513.88 : An14g07220, Aspergillus wentii : Aspwe1_0043650, Aspwe1_0044769 and Aspergillus sydowii : Aspsy1_0040680		
**AN0205.3**	**ANIA_00205**	**0.43**	pantoA	Putative pantoate-beta-alanine ligase with a predicted role in Coenzyme A and pantothenate biosynthesis	Panthothenate synthetase [H]	
**AN0206.3**	**ANIA_00206**	**0.43**		Has domain(s) with predicted glycerol-3-phosphate O-acyltransferase activity, role in phospholipid biosynthetic process and membrane localization	Mitochondrial glycerol-3-phosphate acyltransferase GPAT [I]	
**AN0212.3**	**ANIA_00212**	**0.46**		Has domain(s) with predicted FMN binding, oxidoreductase activity and role in oxidation-reduction process	Uncharacterized conserved protein [S]	
**AN0216.3**	**ANIA_00216**	**0.17**		Has domain(s) with predicted nucleotide binding, oxidoreductase activity and role in metabolic process	1-Acyl dihydroxyacetone phosphate reductase and related dehydrogenases [Q]	
**AN0221.3**	**ANIA_00221**	**0.43**		Putative chitinase; glycoside hydrolase family 18 (GH18) protein with a predicted role in chitin hydrolysis	Chitinase [G]	
**AN0240.3**	**ANIA_00240**	**0.28**	pppA	Putative transaldolase with a predicted role in the pentose-phosphate shunt; intracellular, menadione stress-induced protein; protein induced by farnesol	Transaldolase [G]	
**AN0246.3**	**ANIA_00246**	**0.35**		Ortholog of A. fumigatus Af293 : Afu1g05300, A. niger CBS 513.88 : An01g04570, A. oryzae RIB40 : AO090003000826, Aspergillus wentii : Aspwe1_0049630 and Aspergillus sydowii : Aspsy1_0142918		
**AN0249.3**	**ANIA_00249**	**0.46**		Putative F-box protein		
**AN0285.3**	**ANIA_00285**	**0.30**		Putative 6-phosphogluconolactonase with a predicted role in gluconic acid and gluconate metabolism; expression upregulated after exposure to farnesol	6-phosphogluconolactonase - like protein [G]	
**AN0289.3**	**ANIA_00289**	**0.44**		Ortholog(s) have U2-type spliceosomal complex localization	Uncharacterized conserved protein [R]	
**AN0294.3**	**ANIA_00294**	**0.42**		Ortholog(s) have cytoplasm localization		
**AN0295.3**	**ANIA_00295**	**0.48**		Ortholog(s) have role in ER-associated misfolded protein catabolic process and cytoplasm-associated proteasomal ubiquitin-dependent protein catabolic process, more	Nuclear pore complex, rNpl4 component (sc Npl4) [Y, U]	
**AN0297.3**	**ANIA_00297**	**0.43**		Putative protoplast secreted protein 2; intracellular, menadione stress-induced protein	1,4-benzoquinone reductase-like; Trp repressor binding protein-like/protoplast-secreted protein [R]	
**AN0311.3**	**ANIA_00311**	**0.26**		Ortholog of A. fumigatus Af293 : Afu1g02600, A. niger CBS 513.88 : An01g05600, A. oryzae RIB40 : AO090005000834, Aspergillus wentii : Aspwe1_0169640 and Aspergillus sydowii : Aspsy1_0027873		
**AN0326.3**	**ANIA_00326**	**0.30**		Ortholog of A. fumigatus Af293 : Afu1g02400, A. niger CBS 513.88 : An01g05790, A. oryzae RIB40 : AO090005000857, Aspergillus wentii : Aspwe1_0025630 and Aspergillus terreus NIH2624 : ATET_04850		
**AN0330.3**	**ANIA_00330**	**0.48**		NADH:flavin oxidoreductase; upregulated in A. oryzae and A. nidulans under hypoxic growth conditions	NADH:flavin oxidoreductase/12-oxophytodienoate reductase [C, R]	
**AN0335.3**	**ANIA_00335**	**0.49**		Ortholog of A. fumigatus Af293 : Afu1g02330, A. niger CBS 513.88 : An01g05850, A. oryzae RIB40 : AO090005000867, Aspergillus wentii : Aspwe1_0103633 and Aspergillus sydowii : Aspsy1_0633416		
**AN0337.3**	**ANIA_00337**	**0.38**		Ortholog of A. fumigatus Af293 : Afu1g07195, A. niger CBS 513.88 : An07g09780, A. oryzae RIB40 : AO090012000973, Aspergillus wentii : Aspwe1_0028307 and Aspergillus sydowii : Aspsy1_0047732		
**AN0346.3**	**ANIA_00346**	**0.47**		Ortholog(s) have cytosol, nucleus, plasma membrane localization	Mitochondrial carrier protein - Rim2p/Mrs12p [C]	
**AN0353.3**	**ANIA_00353**	**0.16**		Putative F-box protein		
**AN0376.3**	**ANIA_00376**	**0.27**		Has domain(s) with predicted RNA binding, RNA-directed DNA polymerase activity, role in RNA-dependent DNA replication and nucleus localization		

AN0381.3	ANIA_00381	0.44		Ortholog(s) have unfolded protein binding activity, role in protein folding and chaperonin-containing T-complex, nucleus localization	Chaperonin complex component, TCP-1 beta subunit (CCT2) [O]
AN0396.3	ANIA_00396	0.11		Has domain(s) with predicted protein dimerization activity	
AN0397.3	ANIA_00397	0.17		Protein of unknown function	
AN0400.3	ANIA_00400	0.47		Ortholog of A. fumigatus Af293 : Afu4g09610, A. niger CBS 513.88 : An04g07500, A. oryzae RIB40 : AO090003001281, Neosartorya fischeri NRRL 181 : NFIA_106400 and Aspergillus wentii : Aspwe1_0022929	
AN0411.3	ANIA_00411	0.49		Putative GTPase with a predicted role in ER to Golgi transport	Vesicle coat complex COPII, GTPase subunit SAR1 [U]
AN0421.3	ANIA_00421	0.41		Ortholog(s) have rRNA primary transcript binding activity	RNA-binding protein (RRM superfamily) [R]
AN0429.3	ANIA_00429	0.45		Ortholog(s) have protein transporter activity, unfolded protein binding activity and role in protein import into mitochondrial inner membrane, protein import into mitochondrial outer membrane	Mitochondrial import inner membrane translocase, subunits TIM10/TIM12 [U]
AN0433.3	ANIA_00433	0.47		Ortholog(s) have cell surface, cytosol, nucleolus localization	60S ribosomal protein L18A [J]
AN0434.3	ANIA_00434	0.36		Ortholog(s) have cytosol, nucleus localization	K-associated recombination protein - Thp1p [D]
AN0436.3	ANIA_00436	0.49		Ortholog(s) have cytosol, nucleus localization	Predicted E3 ubiquitin ligase [O]
AN0447.3	ANIA_00447	0.40		Ortholog(s) have mitochondrion localization	NifU-like domain-containing proteins [O]
AN0460.3	ANIA_00460	0.42		Putative F-box protein	
AN0463.3	ANIA_00463	0.48		Predicted Rac guanine nucleotide exchange factor (Rac GEF)	Signaling protein DOCK180 [T]
AN0470.3	ANIA_00470	0.43		Has domain(s) with predicted structural constituent of ribosome activity, role in translation and ribosome localization	Ribosomal protein S7 [J]
AN0471.3	ANIA_00471	0.44	vcxA	Putative vacuolar H+/Ca2+ exchanger	Ca2+/H+ antiporter VCX1 and related proteins [P]
AN0473.3	ANIA_00473	0.42		Has domain(s) with predicted role in transmembrane transport and integral to membrane localization	Permease of the major facilitator superfamily [G]
AN0476.3	ANIA_00476	0.37		Ortholog(s) have mitochondrion localization	Mitochondrial ribosomal protein L27 [J]
AN0478.3	ANIA_00478	0.29		Protein of unknown function	
AN0483.3	ANIA_00483	0.42		Ortholog of Neosartorya fischeri NRRL 181 : NFIA_058180, Aspergillus versicolor : Aspve1_0158177, Aspergillus clavatus NRRL 1 : ACLA_085420 and Aspergillus zonatus : Aspzo1_0018005	
AN0487.3	ANIA_00487	0.49		Has domain(s) with predicted transferase activity, transferring hexosyl groups activity and role in lipid glycosylation	UDP-glucuronosyl and UDP-glucosyl transferase [G, C]
AN0518.3	ANIA_00518	0.47		Ortholog of A. nidulans FGSC A4 : AN6482, A. fumigatus Af293 : Afu1g15540, A. niger CBS 513.88 : An12g09790, A. oryzae RIB40 : AO090011000265 and Neosartorya fischeri NRRL 181 : NFIA_009780	
AN0532.3	ANIA_00532	0.14		Predicted DDE1 transposon-related ORF	
AN0550.3	ANIA_00550	0.22		Putative glucan 1,3-beta-glucosidase with a predicted role in glucan metabolism	
AN0559.3	ANIA_00559	0.41		Ortholog(s) have mitochondrion targeting sequence binding, protein channel activity	Translocase of outer mitochondrial membrane complex, subunit TOM20 [U]
AN0586.3	ANIA_00586	0.25		Protein of unknown function	Flavin-containing monooxygenase [Q]
AN0594.3	ANIA_00594	0.13		Ortholog(s) have role in actin filament reorganization involved in cell cycle, budding cell apical bud growth, cell morphogenesis, cellular response to biotic stimulus and cellular response to drug, more	Fry-like conserved proteins [R]
AN0596.3	ANIA_00596	0.45	ddbA	Putative DNA damage binding protein involved in the DNA damage response; homolog of human DDB1; NeddH-associated protein	Damage-specific DNA binding complex, subunit DDB1 [L]
AN0604.3	ANIA_00604	0.34		Ortholog(s) have role in pyrimidine dimer repair by nucleotide-excision repair, response to UV	
AN0624.3	ANIA_00624	0.44		Ortholog(s) have mitochondrion, nucleus localization	
AN0634.3	ANIA_00634	0.40		Predicted ADP ribosylation factor GTPase	GTP-binding ADP-ribosylation factor-like protein yARL3 [U]
AN0636.3	ANIA_00636	0.40		Ortholog(s) have acetylglucosaminyltransferase activity and role in GPI anchor biosynthetic process, conidium formation, establishment or maintenance of cell polarity, fungal-type cell wall biogenesis, regulation of growth rate	N-acetylglucosaminyltransferase complex, subunit PIG-A/SPT14, required for phosphatidylinositol biosynthesis/Sulfolipid synthase [M, O, I]
AN0639.3	ANIA_00639	0.44		Ortholog of A. fumigatus Af293 : Afu1g16860, A. niger CBS 513.88 : An01g10020, Neosartorya fischeri NRRL 181 : NFIA_008500, Aspergillus wentii : Aspwe1_0027440 and Aspergillus versicolor : Aspve1_0252372	
AN0658.3	ANIA_00658	0.11		Has domain(s) with predicted DNA binding, copper ion binding, sequence-specific DNA binding transcription factor activity, role in regulation of transcription, DNA-dependent and nucleus localization	
AN0659.3	ANIA_00659	0.49		Ortholog of A. fumigatus Af293 : Afu1g13200, A. niger CBS 513.88 : An08g06230, A. oryzae RIB40 : AO090012001040, Aspergillus wentii : Aspwe1_0051321 and Aspergillus sydowii : Aspsy1_0065853	
AN0660.3	ANIA_00660	0.49	furA	Putative allantoin transporter; transcript is induced by allantoin and by uric acid and is repressed by ammonium	Uridine permease/thiamine transporter/allantoin transport [F, H]
AN0681.3	ANIA_00681	0.36		Has domain(s) with predicted catalytic activity, pyridoxal phosphate binding activity	Cystathionine beta-lyases/cystathionine gamma-synthases [E]
AN0690.3	ANIA_00690	0.46		Ortholog(s) have cytosol, nucleus localization	N-methyltransferase [R]
AN0694.3	ANIA_00694	0.31		Ortholog(s) have intracellular localization	
AN0712.3	ANIA_00712	0.25	bglB	Protein with beta-glucosidase activity, involved in degradation of glucans	
AN0715.3	ANIA_00715	0.37		Has domain(s) with predicted intracellular localization	Apoptosis inhibitor IAP1 and related BIR domain proteins [D, R]
AN0756.3	ANIA_00756	0.42	lacA	Beta-galactosidase with a predicted role in lactose metabolism	Beta-galactosidase [G]
AN0757.3	ANIA_00757	0.49		Ortholog(s) have tRNA (cytosine-5-)-methyltransferase activity, role in tRNA methylation and nucleus localization	tRNA cytosine-5-methylases and related enzymes of the NOL1/NOP2/sun superfamily [J]
AN0768.3	ANIA_00768	0.30		Ortholog of A. fumigatus Af293 : Afu1g14300, A. oryzae RIB40 : AO090011000381, Neosartorya fischeri NRRL 181 : NFIA_011110 and Aspergillus versicolor : Aspve1_0067752	Fasciclin and related adhesion glycoproteins [M, W]
AN0775.3	ANIA_00775	0.19		Has domain(s) with predicted NAD binding, oxidoreductase activity, acting on the CH-OH group of donors, NAD or NADP as acceptor activity and role in oxidation-reduction process	Glyoxylate/hydroxypyruvate reductase (D-isomer-specific 2-hydroxy acid dehydrogenase superfamily) [C]
AN0803.3	ANIA_00803	0.50		Ortholog of A. fumigatus Af293 : Afu1g14620, A. niger CBS 513.88 : An01g12620, A. oryzae RIB40 : AO090003001568, Aspergillus wentii : Aspwe1_0027658 and Aspergillus sydowii : Aspsy1_0139476	Myosin class II heavy chain [Z]
AN0806.3	ANIA_00806	0.36		Ortholog(s) have role in single-species biofilm formation on inanimate substrate and cytosol, nucleus localization	WD40 repeat protein [R]
AN0821.3	ANIA_00821	0.44		Ortholog(s) have role in establishment or maintenance of cell polarity, mitochondrial genome maintenance, mitochondrion inheritance, phospholipid transport, protein import into mitochondrial outer membrane	
AN0836.3	ANIA_00836	0.30		Ortholog(s) have D-arabinono-1,4-lactone oxidase activity	D-arabinono-1, 4-lactone oxidase [V]
AN0839.3	ANIA_00839	0.41		Has domain(s) with predicted oxidoreductase activity and role in carotenoid biosynthetic process, chlorophyll biosynthetic process, oxidation-reduction process, photosynthesis	
AN0871.3	ANIA_00871	0.49		Ortholog(s) have GPI-anchor transamidase activity, role in attachment of GPI anchor to protein and GPI-anchor transamidase complex localization	Gpi-anchor transamidase [O]
AN0876.3	ANIA_00876	0.23		Has domain(s) with predicted FMN binding, riboflavin reductase (NADPH) activity and role in oxidation-reduction process	
AN0886.3	ANIA_00886	0.17	lamB	Protein with a predicted role in nitrogen metabolism; involved in the utilization of 2-pyrrolidinone as a nitrogen source	
AN0887.3	ANIA_00887	0.15	lamA	Putative urea amidolyase with a predicted role in nitrogen metabolism; required for the utilization of lactams such as 2-pyrrolidinone	3-Methylcrotonyl-CoA carboxylase, biotin-containing subunit/Propionyl-CoA carboxylase, alpha chain/Acetyl-CoA carboxylase, biotin carboxylase subunit [I, E]
AN0895.3	ANIA_00895	0.30		Predicted NAD-dependent oxidoreductase; intracellular; expression upregulated after exposure to farnesol	Predicted NAD-dependent oxidoreductase [R]
AN0907.3	ANIA_00907	0.33		Ortholog(s) have structural constituent of ribosome activity and cytosolic small ribosomal subunit, nucleus localization	40S ribosomal protein S15/S22 [J]

**AN0926.3**	**ANIA_00926**	**0.44**	kapC	Non-essential nuclear transport receptor importin-beta2 subunit; karyopherin superfamily; expression reduced after exposure to farnesol	Nuclear transport receptor Karyopherin-beta2/Transportin (importin beta superfamily) [Y, U]
**AN0937.3**	**ANIA_00937**	**0.43**		Has domain(s) with predicted DNA binding, sequence-specific DNA binding RNA polymerase II transcription factor activity, zinc ion binding activity and role in regulation of transcription, DNA-dependent, transcription, DNA-templated	
**AN0943.3**	**ANIA_00943**	**0.45**	atp20	Putative mitochondrial F1F0-ATP synthase subunit g; ortholog of S. cerevisiae Atp20p; expression reduced after exposure to farnesol	Mitochondrial F1F0-ATP synthase, subunit g/ATP20 [C]
**AN0950.3**	**ANIA_00950**	**0.19**		Ortholog of Aspergillus versicolor : Aspve1_0036555 and Aspergillus sydowii : Aspsy1_0038433	
**AN0972.3**	**ANIA_00972**	**0.44**		Protein of unknown function	
**AN1027.3**	**ANIA_01027**	**0.37**		Ortholog of A. fumigatus Af293 : Afu1g12660, A. niger CBS 513.88 : An08g05410, A. oryzae RIB40 : AO090012000716, Aspergillus wentii : Aspwe1_0040406 and Aspergillus sydowii : Aspsy1_0038316	
**AN1039.3**	**ANIA_01039**	**0.37**		Ortholog(s) have DNA binding activity	Ankyrin repeat and DHHC-type Zn-finger domain containing proteins [R]
**AN1040.3**	**ANIA_01040**	**0.47**		Ortholog of A. oryzae RIB40 : AO090038000523, Aspergillus flavus NRRL 3357 : AFL2T_07855 and Aspergillus sydowii : Aspsy1_0160459, Aspsy1_0165518	
**AN1056.3**	**ANIA_01056**	**0.25**		Ortholog(s) have cytosol, nucleus localization	WD40 repeat-containing protein [R]
**AN1074.3**	**ANIA_01074**	**0.42**		Ortholog(s) have glycine dehydrogenase (decarboxylating) activity, role in glycine catabolic process, one-carbon metabolic process, protein lipoylation and mitochondrion localization	Glycine cleavage system H protein (lipoate-binding) [E]
**AN1075.3**	**ANIA_01075**	**0.44**		Ortholog(s) have ubiquitin-protein ligase activity and role in protein ubiquitination, ubiquitin-dependent protein catabolic process via the multivesicular body sorting pathway	Predicted E3 ubiquitin ligase [O]
**AN1076.3**	**ANIA_01076**	**0.46**		Ortholog(s) have zinc ion transmembrane transporter activity, role in cellular zinc ion homeostasis, vesicle-mediated transport, zinc ion transport and Golgi membrane, cytosol, nucleus localization	Putative Zn2+ transporter MSC2 (cation diffusion facilitator superfamily) [P]
**AN1081.3**	**ANIA_01081**	**0.32**		Ortholog of A. fumigatus Af293 : Afu1g12140, A. niger CBS 513.88 : An08g04440, A. oryzae RIB40 : AO090001000330, Aspergillus wentii : Aspwe1_0503520 and Aspergillus sydowii : Aspsy1_0053292	
**AN1085.3**	**ANIA_01085**	**0.41**		Ortholog(s) have GTPase activity, role in cellular response to drug, endoplasmic reticulum inheritance, endoplasmic reticulum membrane fusion, hexose transport, pathogenesis and cortical endoplasmic reticulum, cytosol localization	GTP-binding protein [R]
**AN1099.3**	**ANIA_01099**	**0.36**	teaC	SH3 domain protein that localizes to hyphal tips and septa; related to Schizosaccharomyces pombe Tea4; required for selection of initial polarity of germ tube emergence; interacts with TeaA at hyphal tips and SepA at hyphal tips and septa	
**AN1103.3**	**ANIA_01103**	**0.36**		Has domain(s) with predicted acetoacetate-CoA ligase activity and role in lipid metabolic process	Acyl-CoA synthetase [I]
**AN1105.3**	**ANIA_01105**	**0.48**		Ortholog(s) have mitochondrion targeting sequence binding, protein channel activity	Translocase of outer mitochondrial membrane complex, subunit TOM22 [U]
**AN1107.3**	**ANIA_01107**	**0.38**		Protein of unknown function	
**AN1109.3**	**ANIA_01109**	**0.45**		Has domain(s) with predicted substrate-specific transmembrane transporter activity, role in transmembrane transport and integral to membrane localization	Predicted transporter (major facilitator superfamily) [R]
**AN1110.3**	**ANIA_01110**	**0.47**		Has domain(s) with predicted role in transmembrane transport and integral to membrane localization	Permease of the major facilitator superfamily [G]
**AN1113.3**	**ANIA_01113**	**0.22**		Protein of unknown function	
**AN1114.3**	**ANIA_01114**	**0.47**		Has domain(s) with predicted protein dimerization activity	
**AN1115.3**	**ANIA_01115**	**0.40**		Putative purine nucleosidase with a predicted role in nucleotide salvage pathways	Predicted inosine-uridine preferring nucleoside hydrolase [F]
**AN1121.3**	**ANIA_01121**	**0.44**		Ortholog of A. fumigatus Af293 : Afu1g11700, A. niger CBS 513.88 : An08g03920, A. oryzae RIB40 : AO090038000248, Aspergillus wentii : Aspwe1_0068693 and Aspergillus sydowii : Aspsy1_0038144	
**AN1122.3**	**ANIA_01122**	**0.43**		Ortholog(s) have cytosol localization	60S ribosomal protein L10A [J]
**AN1132.3**	**ANIA_01132**	**0.49**	qutR	Negative regulator of qut gene expression; mutants constitutively express enzymes of the quinic acid utilization pathway	
**AN1133.3**	**ANIA_01133**	**0.43**		Ortholog(s) have Golgi apparatus, fungal-type vacuole membrane localization	N-methyl-D-aspartate receptor glutamate-binding subunit [T]
**AN1157.3**	**ANIA_01157**	**0.34**	gle1	Putative nuclear pore complex protein	Nuclear-export-signal (NES)-containing protein/polyadenylated-RNA export factor [A]
**AN1162.3**	**ANIA_01162**	**0.41**		Ortholog(s) have guanyl-nucleotide exchange factor activity, translation elongation factor activity, role in translational elongation and cell surface, cytosol, eukaryotic translation elongation factor 1 complex, ribosome localization	Elongation factor 1 beta/delta chain [K]
**AN1166.3**	**ANIA_01166**	**0.44**		Ortholog(s) have RNA binding, structural constituent of ribosome activity	60s ribosomal protein L6 [J]
**AN1179.3**	**ANIA_01179**	**0.46**		Protein with a predicted role in negative regulation of gluconeogenesis and protein targeting to vacuole; protein is translationally repressed by the conserved upstream open reading frame (uORF) AN1179-uORF	Vacuolar import and degradation protein [U]
**AN1191.3**	**ANIA_01191**	**0.49**	sumO	Small ubiquitin-like modifier (SUMO) protein; mutant is viable, slow growing with pleiotropic phenotypes; punctate nucleoplasmic localization at interphase; deletion causes dramatic increase in asperthecin; camptothecin induced transcript	Ubiquitin-like proteins [O]
**AN1192.3**	**ANIA_01192**	**0.42**		Ortholog of A. fumigatus Af293 : Afu1g10840, A. oryzae RIB40 : AO090038000328, Aspergillus sydowii : Aspsy1_0236946 and Aspergillus terreus NIH2624 : ATET_00276	
**AN1228.3**	**ANIA_01228**	**0.44**		Ortholog(s) have role in maturation of LSU-rRNA from tricistronic rRNA transcript (SSU-rRNA, 5.8S rRNA, LSU-rRNA) and cytosol, nucleolus, preribosome, large subunit precursor localization	60S ribosomal protein L35 [J]
**AN1230.3**	**ANIA_01230**	**0.49**		Ortholog(s) have unfolded protein binding activity, role in mitochondrial proton-transporting ATP synthase complex assembly and integral to mitochondrial membrane, mitochondrial inner membrane localization	Inner membrane protein required for assembly of the F0 sector of ATP synthase [O]
**AN1238.3**	**ANIA_01238**	**0.43**	nup120	Putative nuclear pore complex protein with low homology to Saccharomyces cerevisiae Nup120p; synthetically lethal with ndc1 mutation	Nuclear pore complex, Nup160 component [Y, U]
**AN1243.3**	**ANIA_01243**	**0.40**		Has domain(s) with predicted role in transmembrane transport and integral to membrane localization	Synaptic vesicle transporter SVOP and related transporters (major facilitator superfamily) [R]
**AN1244.3**	**ANIA_01244**	**0.42**		Ortholog(s) have cytosol, nucleus localization	Uncharacterized conserved protein [S]
**AN1274.3**	**ANIA_01274**	**0.32**		Ortholog(s) have alditol:NADP+ 1-oxidoreductase activity, glycerol dehydrogenase [NAD(P)+] activity, mRNA binding activity and role in D-xylose catabolic process, arabinose catabolic process, cellular response to oxidative stress	Aldo/keto reductase family proteins [R]
**AN1290.3**	**ANIA_01290**	**0.35**		Has domain(s) with predicted solute:hydrogen antiporter activity, role in cation transport, transmembrane transport and integral to membrane localization	Predicted K+/H+-antiporter [P]
**AN1304.3**	**ANIA_01304**	**0.46**		Protein of unknown function	
**AN1317.3**	**ANIA_01317**	**0.42**		Ortholog of A. nidulans FGSC A4 : AN6413, AN6419, AN6946, AN5312, AN5664, AN8328, AN3241, AN10369, AN1930, AN2386, AN1738, AN11159, AN8984, AN8951, AN1540, AN8971, AN9387, AN9266, AN9306, AN10886, AN7232, AN7774, AN12202	
**AN1334.3**	**ANIA_01334**	**0.43**		Ortholog(s) have mRNA binding, small GTPase regulator activity	Component of vacuolar transporter chaperone (Vtc) involved in vacuole fusion [U, O]

AN1336.3	ANIA_01336	0.48		Ortholog of A. fumigatus Af293 : Afu1g09570, A. niger CBS 513.88 : An08g01160, A. oryzae RIB40 : AO090012000913, Aspergillus wentii : Aspwe1_0024569 and Aspergillus sydowii : Aspsy1_0037865	
AN1351.3	ANIA_01351	0.50		Ortholog of A. fumigatus Af293 : Afu1g09370, A. niger CBS 513.88 : An08g00910, Aspergillus wentii : Aspwe1_0048832, Aspergillus sydowii : Aspsy1_0053020 and Aspergillus terreus NIH2624 : ATET_08561	
AN1364.3	ANIA_01364	0.24		Ortholog(s) have cytosol, nucleus localization	Thioredoxin-like protein [O]
AN1381.3	ANIA_01381	0.42		Ortholog of A. fumigatus Af293 : Afu1g09000, A. oryzae RIB40 : AO090005001625, Aspergillus wentii : Aspwe1_0103608 and Aspergillus sydowii : Aspsy1_0140811	Uncharacterized conserved protein [S]
AN1399.3	ANIA_01399	0.49		Has domain(s) with predicted role in meiotic chromosome segregation and chromosome, centromeric region, nucleus localization	
AN1412.3	ANIA_01412	0.50		Ortholog of A. fumigatus Af293 : Afu8g04030, A. niger CBS 513.88 : An16g09150, A. oryzae RIB40 : AO090103000016, Aspergillus wentii : Aspwe1_0117975 and Aspergillus sydowii : Aspsy1_0144289	
AN1423.3	ANIA_01423	0.40		Has domain(s) with predicted transporter activity, role in transmembrane transport and integral to membrane, plasma membrane localization	Permease of the major facilitator superfamily [R]
AN1474.3	ANIA_01474	0.48		Ortholog(s) have endoplasmic reticulum localization	RNA 3'-terminal phosphate cyclase [A]
AN1486.3	ANIA_01486	0.35		Has domain(s) with predicted ribonuclease activity and role in tRNA processing	Protein subunit of nuclear ribonuclease P (RNase P) [J]
AN1503.3	ANIA_01503	0.44		Protein expressed at increased levels during osmoadaptation; contains dihydrodipicolinate synthase family DHDPS-like conserved domain	
AN1542.3	ANIA_01542	0.41	xgcA	Protein with oligoxyloglucan reducing end-specific cellobiohydrolase activity, involved in degradation of xyloglucans	
AN1547.3	ANIA_01547	0.18	coaT	Acyl-CoA:carboxylate CoA-transferase involved in detoxification of propionyl-CoA ; predicted role in acetate, acetaldehyde, and ethanol (two-carbon) metabolism; expression upregulated after exposure to farnesol	Acetyl-CoA hydrolase [C]
AN1557.3	ANIA_01557	0.18		Protein of unknown function	
AN1574.3	ANIA_01574	0.43		Protein of unknown function; transcript repressed by nitrate	
AN1580.3	ANIA_01580	0.42		Has domain(s) with predicted ADP binding, microtubule motor activity, role in nucleoside metabolic process and kinesin complex localization	Kinesin light chain [Z]
AN1595.3	ANIA_01595	0.39		Putative elongation factor 1-gamma; member of the PbcR-activated diterpene cluster	Glutathione S-transferase [O]
AN1598.3	ANIA_01598	0.49	CYP503B1	Putative cytochrome P450; member of the PbcR-activated diterpene cluster	Cytochrome P450 CYP4/CYP19/CYP26 subfamilies [Q, I]
AN1603.3	ANIA_01603	0.20		Ortholog of A. fumigatus Af293 : Afu8g05985, Neosartorya fischeri NRRL 181 : NFIA_098760, NFIA_099140, Aspergillus wentii : Aspwe1_0689083 and Aspergillus versicolor : Aspve1_0037404	
AN1605.3	ANIA_01605	0.09		Ortholog of Aspergillus sydowii : Aspsy1_0679421	
AN1616.3	ANIA_01616	0.37		Ortholog of A. nidulans FGSC A4 : AN5887, AN4831, A. fumigatus Af293 : Afu2g11250, A. niger CBS 513.88 : An08g09700 and A. oryzae RIB40 : AO090026000525, AO090005000474, AO090113000193	Voltage-gated shaker-like K+ channel, subunit beta/KCNAB [C]
AN1626.3	ANIA_01626	0.09		Ortholog of Aspergillus sydowii : Aspsy1_0053088	
AN1631.3	ANIA_01631	0.39		Ortholog(s) have L-methionine secondary active transmembrane transporter activity and role in cysteine transport, methionine import	Amino acid transporters [E]
AN1648.3	ANIA_01648	0.31		Has domain(s) with predicted GTP binding, GTPase activity	Vacuolar sorting protein VPS1, dynamin, and related proteins [U, R]
AN1651.3	ANIA_01651	0.08		Ortholog of A. nidulans FGSC A4 : AN8144, A. fumigatus Af293 : Afu5g14600, Afu6g00460, Afu6g13950, A. niger CBS 513.88 : An11g02490, An01g11420 and A. oryzae RIB40 : AO090001000384, AO090011000740	
AN1654.3	ANIA_01654	0.31		Ortholog of A. fumigatus Af293 : Afu4g08750, A. niger CBS 513.88 : An04g03610, Aspergillus wentii : Aspwe1_0034423 and Aspergillus sydowii : Aspsy1_0084030	
AN1662.3	ANIA_01662	0.35		Ortholog(s) have cytosol, nucleus, ribosome localization	Uncharacterized conserved protein [S]
AN1665.3	ANIA_01665	0.38	stk51	Has domain(s) with predicted ATP binding, protein tyrosine kinase activity and role in protein phosphorylation	Ca2+/calmodulin-dependent protein kinase, EF-Hand protein superfamily [T]
AN1670.3	ANIA_01670	0.24		Ortholog of A. niger CBS 513.88 : An04g03830, Aspergillus wentii : Aspwe1_0047606, Aspergillus versicolor : Aspve1_0079774 and Aspergillus niger ATCC 1015 : 124618-mRNA	Vesicle coat complex COPII, subunit SFB3 [U]
AN1689.3	ANIA_01689	0.25		Putative oxidoreductase; putative salicylaldehyde dehydrogenase with a predicted role in naphthalene degradation; upregulated under hypoxic growth conditions	Aldehyde dehydrogenase [C]
AN1695.3	ANIA_01695	0.43		Ortholog(s) have Golgi apparatus, cytosol, nucleus localization	Conserved WD40 repeat-containing protein AN11 [S]
AN1702.3	ANIA_01702	0.41		Protein of unknown function	Amino acid transporters [E]
AN1712.3	ANIA_01712	0.43		Ortholog of A. fumigatus Af293 : Afu4g08460, A. oryzae RIB40 : AO090023000716, Neosartorya fischeri NRRL 181 : NFIA_107710, Aspergillus wentii : Aspwe1_0102930 and Aspergillus versicolor : Aspve1_0079819	
AN1714.3	ANIA_01714	0.44		Ortholog of A. nidulans FGSC A4 : AN1718, A. fumigatus Af293 : Afu4g08380, Afu4g08420, A. niger CBS 513.88 : An04g03190, An04g03210 and A. oryzae RIB40 : AO090023000718	
AN1734.3	ANIA_01734	0.34		Ortholog of A. fumigatus Af293 : Afu6g08740, A. niger CBS 513.88 : An11g06130, A. oryzae RIB40 : AO090001000548, Aspergillus wentii : Aspwe1_0120879 and Aspergillus sydowii : Aspsy1_0289054	
AN1740.3	ANIA_01740	0.29		Protein of unknown function	
AN1769.3	ANIA_01769	0.38		Putative 3'-5' bisphosphate nucleotidase with a predicted role in cysteine metabolism; protein expressed at increased levels during osmoadaptation	Salt-sensitive 3'-phosphoadenosine-5'-phosphatase HAL2/SAL1 [F, P]
AN1778.3	ANIA_01778	0.36	pantoB	Putative ketopentoate hydroxymethyl transferase with a predicted role in Coenzyme A and pantothenate biosynthesis	Ketopantoate hydroxymethyltransferase [H]
AN1782.3	ANIA_01782	0.43		Has domain(s) with predicted NAD+ binding activity	Sirtuin 5 and related class III sirtuins (SIR2 family) [B, K]
AN1794.3	ANIA_01794	0.44	CYP682D1	Putative cytochrome P450; expression reduced after exposure to farnesol	Cytochrome P450 CYP3/CYP5/CYP6/CYP9 subfamilies [Q]
AN1796.3	ANIA_01796	0.43		Ortholog(s) have role in DNA repair, actin cytoskeleton organization, cellular manganese ion homeostasis, dephosphorylation, hyphal growth and endoplasmic reticulum localization	Cell division control protein/predicted DNA repair exonuclease [L]
AN1824.3	ANIA_01824	0.41		Has domain(s) with predicted DNA binding, sequence-specific DNA binding RNA polymerase II transcription factor activity, zinc ion binding activity and role in regulation of transcription, DNA-dependent, transcription, DNA-templated	
AN1829.3	ANIA_01829	0.28		Has domain(s) with predicted sequence-specific DNA binding RNA polymerase II transcription factor activity, zinc ion binding activity, role in regulation of transcription, DNA-dependent and nucleus localization	
AN1856.3	ANIA_01856	0.32		Ortholog of A. fumigatus Af293 : Afu4g09880, A. niger CBS 513.88 : An04g07170, Aspergillus wentii : Aspwe1_0034217, Aspergillus sydowii : Aspsy1_0026276 and Aspergillus terreus NIH2624 : ATET_00756	
AN1859.3	ANIA_01859	0.49		Ortholog(s) have role in DNA repair, cellular response to cadmium ion, chromatin silencing at telomere, detoxification of cadmium ion, histone exchange and NuA4 histone acetyltransferase complex, Swr1 complex, cytosol localization	K initiation factor IIF, auxiliary subunit [K]
AN1864.3	ANIA_01864	0.38		Ortholog of A. fumigatus Af293 : Afu2g04535, A. niger CBS 513.88 : An11g03760, A. oryzae RIB40 : AO090003000246, Aspergillus wentii : Aspwe1_0055095 and Aspergillus sydowii : Aspsy1_0084294	

**AN1868.3**	**ANIA_01868**	**0.50**		Putative glycerol dehydrogenase with a predicted role in glycerol metabolism; protein expressed at decreased levels in a hapX mutant versus wild-type	Alcohol dehydrogenase, class IV [C]
**AN1904.3**	**ANIA_01904**	**0.46**		Ortholog(s) have chaperonin-containing T-complex localization	Chaperonin complex component, TCP-1 epsilon subunit (CCT5) [O]
**AN1915.3**	**ANIA_01915**	**0.29**		Ortholog(s) have electron carrier activity, oxidoreductase activity, acting on NAD(P)H, heme protein as acceptor activity and role in electron transport chain, heme a biosynthetic process	Cytochrome oxidase assembly factor COX15 [O]
**AN1930.3**	**ANIA_01930**	**0.18**		Ortholog of A. nidulans FGSC A4 : AN6413, AN6419, AN6946, AN5312, AN5664, AN8328, AN3241, AN10369, AN2386, AN1738, AN11159, AN8984, AN8951, AN1540, AN8971, AN9387, AN9266, AN9306, AN1317, AN10886, AN7232, AN7774, AN12202	
**AN1932.3**	**ANIA_01932**	**0.42**	msrB	Putative methionine sulfoxide reductase with a predicted role in the reduction of methionine sulfoxide to methionine	Predicted pilin-like K factor [O]
**AN1945.3**	**ANIA_01945**	**0.39**		Ortholog(s) have cytoplasm, nucleus localization	
**AN1960.3**	**ANIA_01960**	**0.31**		Ortholog of A. fumigatus Af293 : Afu4g10850, A. oryzae RIB40 : AO090003001126, Aspergillus wentii : Aspwe1_0022807, Aspergillus sydowii : Aspsy1_0026393 and Aspergillus terreus NIH2624 : ATET_03985	
**AN1964.3**	**ANIA_01964**	**0.33**		Ortholog of S. cerevisiae RPS6B and RPS6A; palA-dependent expression independent of pH	40S ribosomal protein S6 [J]
**AN1977.3**	**ANIA_01977**	**0.34**		Ortholog of A. fumigatus Af293 : Afu4g10650, A. niger CBS 513.88 : An04g06050, A. oryzae RIB40 : AO090003001149, Neosartorya fischeri NRRL 181 : NFIA_105430 and Aspergillus clavatus NRRL 1 : ACLA_049840	Uncharacterized conserved protein [S]
**AN1986.3**	**ANIA_01986**	**0.45**		Ortholog(s) have ferrous iron binding, ferroxidase activity, iron chaperone activity	Mitochondrial matrix protein frataxin, involved in Fe/S protein biosynthesis [P]
**AN1987.3**	**ANIA_01987**	**0.37**		Ortholog of A. fumigatus Af293 : Afu4g10500, A. niger CBS 513.88 : An04g06170, A. oryzae RIB40 : AO090003001161, Aspergillus wentii : Aspwe1_0047460 and Aspergillus sydowii : Aspsy1_0316623	
**AN2005.3**	**ANIA_02005**	**0.38**	ypdA	Histidine-containing phosphotransfer protein, part of a two-component signal transducer involved in the HOG signaling pathway that regulates osmotic stress response	Two-component phosphorelay intermediate involved in MAP kinase cascade regulation [T]
**AN2017.3**	**ANIA_02017**	**0.29**	agdA	Putative alpha-glucosidase with a predicted role in maltose metabolism; transcriptionally induced by isomaltose	Maltase glucoamylase and related hydrolases, glycosyl hydrolase family 31 [G]
**AN2023.3**	**ANIA_02023**	**0.22**		Ortholog of A. niger CBS 513.88 : An11g07220, An18g00750, An16g04060, A. oryzae RIB40 : AO090001000252 and Aspergillus wentii : Aspwe1_0105181, Aspwe1_0181876	
**AN2032.3**	**ANIA_02032**	**0.32**	pkhA	Putative polyketide synthase; involved in secondary metabolite production	Animal-type fatty acid synthase and related proteins [I]
**AN2052.3**	**ANIA_02052**	**0.29**		Ortholog(s) have oligopeptide-transporting ATPase activity, role in oligopeptide export from mitochondrion and mitochondrial inner membrane localization	Peptide exporter, ABC superfamily [U]
**AN2078.3**	**ANIA_02078**	**0.45**		Has domain(s) with predicted RNA polymerase II transcription cofactor activity, role in regulation of transcription from RNA polymerase II promoter and mediator complex localization	
**AN2084.3**	**ANIA_02084**	**0.48**		Ortholog of Aspergillus tubingensis : Asptu1_0931510, Aspergillus glaucus : Aspgl1_0073071, Aspergillus acidus : Aspfo1_0057156 and Aspergillus wentii : Aspwe1_0055136	
**AN2086.3**	**ANIA_02086**	**0.41**	nup159	Putative nuclear pore complex protein; localizes to the nuclear periphery during interphase and disperses throughout the cell during mitosis	Nuclear pore complex, Nup214/CAN component [Y, U]
**AN2107.3**	**ANIA_02107**	**0.42**		Has domain(s) with predicted GTP binding, GTPase activity	Predicted translation elongation factor [J]
**AN2108.3**	**ANIA_02108**	**0.30**		Ortholog of Aspergillus wentii : Aspwe1_0149185, Aspergillus versicolor : Aspve1_0123190, Aspve1_0187353, Aspergillus niger ATCC 1015 : 178726-mRNA and Aspergillus sydowii : Aspsy1_0039267, Aspsy1_0391823	
**AN2114.3**	**ANIA_02114**	**0.27**		Has domain(s) with predicted oxidoreductase activity and role in metabolic process	Kynurenine 3-monooxygenase and related flavoprotein monooxygenases [C, R]
**AN2124.3**	**ANIA_02124**	**0.38**		Putative diacylglycerol pyrophosphate phosphatase with a predicted role in phospholipid metabolism	Lipid phosphate phosphatase and related enzymes of the PAP2 family [I]
**AN2128.3**	**ANIA_02128**	**0.34**		Ortholog(s) have role in cellular protein localization, cytokinesis checkpoint, dynein-driven meiotic oscillatory nuclear movement, microtubule bundle formation and mitotic spindle elongation, more	Microtubule-associated protein essential for anaphase spindle elongation [D, Z]
**AN2142.3**	**ANIA_02142**	**0.37**	kapA	Karyopherin (importin) alpha, involved in protein import into nucleus	Karyopherin (importin) alpha [U]
**AN2143.3**	**ANIA_02143**	**0.47**		Ortholog(s) have role in mitotic sister chromatid cohesion and mating-type region heterochromatin, mitotic cohesin complex, nuclear centromeric heterochromatin, telomeric heterochromatin localization	Sister chromatid cohesion complex Cohesin, subunit STAG/IRR1/SCC3 [D]
**AN2162.3**	**ANIA_02162**	**0.37**		Has domain(s) with predicted zinc ion binding activity	Predicted E3 ubiquitin ligase [O]
**AN2165.3**	**ANIA_02165**	**0.47**	llmA	Putative LaeA-like methyltransferase	
**AN2194.3**	**ANIA_02194**	**0.39**		Has domain(s) with predicted serine-type endopeptidase activity and role in proteolysis	
**AN2199.3**	**ANIA_02199**	**0.48**		Possible pseudogene	
**AN2200.3**	**ANIA_02200**	**0.28**		Ortholog(s) have sulfonate dioxygenase activity and role in sulfur compound catabolic process	
**AN2221.3**	**ANIA_02221**	**0.16**		Ortholog of A. niger CBS 513.88 : An13g02070, Aspergillus versicolor : Aspve1_0050620, Aspergillus niger ATCC 1015 : 44818-mRNA and Aspergillus sydowii : Aspsy1_0039729	
**AN2227.3**	**ANIA_02227**	**0.39**	bglI	Putative beta-glucosidase with a predicted role in degradation of glucans	
**AN2234.3**	**ANIA_02234**	**0.43**		Ortholog of A. fumigatus Af293 : Afu5g07265, A. niger CBS 513.88 : An17g00700, A. oryzae RIB40 : AO090701000229, Aspergillus wentii : Aspwe1_0035000 and Aspergillus sydowii : Aspsy1_0039713	
**AN2255.3**	**ANIA_02255**	**0.21**		Has domain(s) with predicted ATP binding, DNA binding, helicase activity	K regulator XNP/ATRX, DEAD-box superfamily [K]
**AN2256.3**	**ANIA_02256**	**0.12**		Has domain(s) with predicted ATP binding, DNA binding, helicase activity, ubiquitin-protein ligase activity, zinc ion binding activity, role in protein ubiquitination and ubiquitin ligase complex localization	Helicase-like K factor HLTF/DNA helicase RAD5, DEAD-box superfamily [K, L]
**AN2275.3**	**ANIA_02275**	**0.40**		Ortholog(s) have structural constituent of ribosome activity, role in cytoplasmic translation and cytosolic large ribosomal subunit, nucleus localization	60s ribosomal protein L2/L8 [J]
**AN2294.3**	**ANIA_02294**	**0.43**	elp3	Putative GNAT-type acetyltransferase	RNA polymerase II elongator complex, subunit ELP3/histone acetyltransferase [B, K]
**AN2301.3**	**ANIA_02301**	**0.49**		Has domain(s) with predicted structural constituent of ribosome activity, role in translation and ribosome localization	60s ribosomal protein L39 [J]
**AN2306.3**	**ANIA_02306**	**0.33**		Putative ubiquinol-cytochrome-c reductase subunit with a predicted role in energy metabolism; intracellular, menadione stress-induced protein	Ubiquinol cytochrome c reductase, subunit RIP1 [C]
**AN2316.3**	**ANIA_02316**	**0.38**		Putative cytochrome c oxidase subunit with a predicted role in energy metabolism	Cytochrome c oxidase, subunit IV/COX5b [C]
**AN2321.3**	**ANIA_02321**	**0.24**		Has domain(s) with predicted role in transmembrane transport and integral to membrane localization	Permease of the major facilitator superfamily [G]
**AN2322.3**	**ANIA_02322**	**0.38**		Ortholog of Neosartorya fischeri NRRL 181 : NFIA_005120	
**AN2327.3**	**ANIA_02327**	**0.48**	cnxF	Protein with a role in molybdopterin cofactor biosynthesis; molybdopterin cofactor required for the activity of nitrate reductase	Molybdopterin synthase sulfurylase [H]
**AN2333.3**	**ANIA_02333**	**0.48**		Has domain(s) with predicted hydrolase activity, acting on carbon-nitrogen (but not peptide) bonds, in linear amides activity, role in ceramide metabolic process and integral to membrane localization	Alkaline ceramidase [I]
**AN2339.3**	**ANIA_02339**	**0.37**		Protein of unknown function	
**AN2404.3**	**ANIA_02404**	**0.15**		Ortholog of A. fumigatus Af293 : Afu8g05810, A. niger CBS 513.88 : An15g04670, A. oryzae RIB40 : AO090005000570, Aspergillus wentii : Aspwe1_0118486 and Aspergillus sydowii : Aspsy1_0039439	Predicted steroid reductase [R]
**AN2406.3**	**ANIA_02406**	**0.38**		Ortholog(s) have intracellular localization	

**AN2407.3**	**ANIA_02407**	**0.21**		Has domain(s) with predicted role in isoprenoid biosynthetic process	Geranylgeranyl pyrophosphate synthase/Polyprenyl synthetase [H]
**AN2410.3**	**ANIA_02410**	**0.40**		Has domain(s) with predicted hydrolase activity and role in cellular metabolic process	Predicted translation initiation factor related to eIF-2B alpha/beta/delta subunits (CIG2/IDI2) [J]
**AN2414.3**	**ANIA_02414**	**0.49**		Putative NADH dehydrogenase (ubiquinone) with a predicted role in energy metabolism	NADH:ubiquinone oxidoreductase, NDUFS2/49 kDa subunit [C]
**AN2432.3**	**ANIA_02432**	**0.42**		Ortholog(s) have chaperone binding, unfolded protein binding activity, role in protein refolding and mitochondrial matrix localization	Mitochondrial chaperonin [O]
**AN2438.3**	**ANIA_02438**	**0.38**		Ortholog(s) have role in RNA polymerase II complex localization to nucleus, RNA polymerase III complex localization to nucleus, establishment of mitotic sister chromatid cohesion and cytosol, nucleus localization	Predicted GTPase [R]
**AN2450.3**	**ANIA_02450**	**0.46**	ubaB	SumO activating (E1) enzyme	SMT3/SUMO-activating complex, catalytic component UBA2 [O]
**AN2453.3**	**ANIA_02453**	**0.48**	cyp5	Putative peptidyl-prolyl cis-trans isomerase (PPIase)	Similar to cyclophilin-type peptidyl-prolyl cis-trans isomerase [O]
**AN2455.3**	**ANIA_02455**	**0.31**		Ortholog(s) have cytosol, nucleus localization	C4-type Zn-finger protein [R]
**AN2463.3**	**ANIA_02463**	**0.43**	lacF	Putative beta-galactosidase with a predicted role in lactose metabolism	Predicted beta-mannosidase [G]
**AN2466.3**	**ANIA_02466**	**0.42**		Has domain(s) with predicted substrate-specific transmembrane transporter activity, role in transmembrane transport and integral to membrane localization	Predicted transporter (major facilitator superfamily) [R]
**AN2470.3**	**ANIA_02470**	**0.39**		Ortholog(s) have Golgi apparatus localization	Sorbitol dehydrogenase [Q]
**AN2472.3**	**ANIA_02472**	**0.46**		Has domain(s) with predicted catalytic activity	Serine/threonine protein phosphatase [T]
**AN2478.3**	**ANIA_02478**	**0.40**		Ortholog of A. fumigatus Af293 : Afu6g00240, A. niger CBS 513.88 : An13g02290, A. oryzae RIB40 : AO090103000492 and Aspergillus wentii : Aspwe1_0179466	
**AN2479.3**	**ANIA_02479**	**0.34**	ngn16	Putative GNAT-type acetyltransferase	Diamine acetyltransferase [E]
**AN2485.3**	**ANIA_02485**	**0.47**		Ortholog(s) have role in chromatin remodeling and Ino80 complex, cytoplasm localization	Uncharacterized conserved protein [S]
**AN2503.3**	**ANIA_02503**	**0.37**		Ortholog(s) have role in response to endoplasmic reticulum stress	Metacaspase involved in regulation of apoptosis [D, O]
**AN2504.3**	**ANIA_02504**	**0.41**		Has domain(s) with predicted oxidoreductase activity and role in oxidation-reduction process	
**AN2509.3**	**ANIA_02509**	**0.35**		Putative tryptophan 2,3-dioxygenase with a predicted role in aromatic amino acid biosynthesis	
**AN2513.3**	**ANIA_02513**	**0.44**	pipA	Putative serine/threonine protein kinase, interacts with PtkA, cyclin-dependent kinase, in metulae, phialides and conidia; essential; involved in hyphal growth and asexual development	Serine/threonine protein kinase [R]
**AN2515.3**	**ANIA_02515**	**0.33**		Has domain(s) with predicted oxidoreductase activity and role in oxidation-reduction process	Aldo/keto reductase family proteins [R]
**AN2535.3**	**ANIA_02535**	**0.20**		Ortholog of A. fumigatus Af293 : Afu3g14660, A. niger CBS 513.88 : An09g01230, Aspergillus wentii : Aspwe1_0107397, Aspwe1_0171355 and Aspergillus sydowii : Aspsy1_0087029	
**AN2553.3**	**ANIA_02553**	**0.45**		Has domain(s) with predicted DNA binding, sequence-specific DNA binding RNA polymerase II transcription factor activity, zinc ion binding activity and role in regulation of transcription, DNA-dependent, transcription, DNA-templated	
**AN2558.3**	**ANIA_02558**	**0.40**		Ortholog of A. fumigatus Af293 : Afu3g15050, A. niger CBS 513.88 : An14g03430, A. oryzae RIB40 : AO090701000428, Aspergillus wentii : Aspwe1_0175480 and Aspergillus sydowii : Aspsy1_0055978	Flavin-containing monooxygenase [Q]
**AN2564.3**	**ANIA_02564**	**0.49**		Has domain(s) with predicted catalytic activity, pyridoxal phosphate binding activity and role in biosynthetic process	1-aminocyclopropane-1-carboxylate synthase, and related proteins [T]
**AN2570.3**	**ANIA_02570**	**0.04**		Ortholog of Aspergillus versicolor : Aspve1_0039071 and Aspergillus sydowii : Aspsy1_0146617	
**AN2571.3**	**ANIA_02571**	**0.22**		Protein of unknown function	
**AN2574.3**	**ANIA_02574**	**0.33**		Has domain(s) with predicted UDP-N-acetylmuramate dehydrogenase activity, flavin adenine dinucleotide binding activity and role in oxidation-reduction process	Proteins containing the FAD binding domain [C]
**AN2595.3**	**ANIA_02595**	**0.45**		Protein of unknown function	
**AN2613.3**	**ANIA_02613**	**0.24**		Has domain(s) with predicted role in transmembrane transport and integral to membrane localization	Synaptic vesicle transporter SVOP and related transporters (major facilitator superfamily) [R]
**AN2615.3**	**ANIA_02615**	**0.49**		Has domain(s) with predicted sequence-specific DNA binding RNA polymerase II transcription factor activity, zinc ion binding activity, role in regulation of transcription, DNA-dependent and nucleus localization	
**AN2616.3**	**ANIA_02616**	**0.37**		Has domain(s) with predicted RNA binding, RNA-directed DNA polymerase activity, role in DNA integration, RNA-dependent DNA replication and nucleus localization	Heterochromatin-associated protein HP1 and related CHROMO domain proteins [B]
**AN2637.3**	**ANIA_02637**	**0.49**		Ortholog of A. fumigatus Af293 : Afu2g13650, A. oryzae RIB40 : AO090001000291, AO090005001139, AO090026000243, Aspergillus wentii : Aspwe1_0045467 and Aspergillus sydowii : Aspsy1_0035381, Aspsy1_0047966	Predicted Mg2+-dependent phosphodiesterase TTRAP [T]
**AN2638.3**	**ANIA_02638**	**0.40**	hxkB	Putative hexokinase with a predicted role in carbohydrate metabolism	Hexokinase [G]
**AN2639.3**	**ANIA_02639**	**0.22**		Has domain(s) with predicted hydrolase activity	
**AN2640.3**	**ANIA_02640**	**0.45**		Ortholog of A. fumigatus Af293 : Afu5g14080, A. niger CBS 513.88 : An12g05640, Neosartorya fischeri NRRL 181 : NFIA_072900 and Aspergillus versicolor : Aspve1_0040725	
**AN2666.3**	**ANIA_02666**	**0.41**		Putative oxidoreductase with a predicted role in carbohydrate or amino acid metabolism; putative D-xylulose reductase	Sorbitol dehydrogenase [Q]
**AN2689.3**	**ANIA_02689**	**0.23**	ulpA	SUMO-specific isopeptidase	Protease, Ulp1 family [O]
**AN2710.3**	**ANIA_02710**	**0.41**		Ortholog of A. nidulans FGSC A4 : AN6951, AN6833, A. fumigatus Af293 : Afu1g01520, A. oryzae RIB40 : AO090038000445, Neosartorya fischeri NRRL 181 : NFIA_023140 and Aspergillus wentii : Aspwe1_0116569	
**AN2720.3**	**ANIA_02720**	**0.06**		Has domain(s) with predicted catalytic activity	Predicted hydrolase/acyltransferase (alpha/beta hydrolase superfamily) [R]
**AN2728.3**	**ANIA_02728**	**0.24**		Has domain(s) with predicted lyase activity and role in metabolic process	
**AN2729.3**	**ANIA_02729**	**0.50**		Has domain(s) with predicted role in transmembrane transport and integral to membrane localization	Permease of the major facilitator superfamily [G]
**AN2733.3**	**ANIA_02733**	**0.47**		Ortholog(s) have uroporphyrinogen decarboxylase activity, role in heme biosynthetic process and cytosol, nucleus localization	Uroporphyrinogen decarboxylase [H]
**AN2741.3**	**ANIA_02741**	**0.35**		Has domain(s) with predicted selenium binding activity and role in cell redox homeostasis	
**AN2751.3**	**ANIA_02751**	**0.39**	pat1	Putative ortholog of PAT1, a topoisomerase II-associated protein; transcript upregulated in response to camptothecin	Uncharacterized conserved protein [S]
**AN2763.3**	**ANIA_02763**	**0.49**		Ortholog(s) have role in filamentous growth of a population of unicellular organisms in response to biotic stimulus and growth of unicellular organism as a thread of attached cells, more	
**AN2772.3**	**ANIA_02772**	**0.23**	bimE	Component of the Anaphase-Promoting Complex/Cyclosome (APC/C), which is a ubiquitin ligase required for cell cycle progression; negative regulator of mitosis	Anaphase-promoting complex (APC), subunit 1 (meiotic check point regulator/Tsg24) [D, O]
**AN2785.3**	**ANIA_02785**	**0.33**		Has domain(s) with predicted sequence-specific DNA binding RNA polymerase II transcription factor activity, zinc ion binding activity, role in regulation of transcription, DNA-dependent and nucleus localization	
**AN2798.3**	**ANIA_02798**	**0.19**		Has domain(s) with predicted role in response to stress and integral to membrane localization	
**AN2801.3**	**ANIA_02801**	**0.28**		Has domain(s) with predicted oxidoreductase activity and role in oxidation-reduction process	Aldo/keto reductase family proteins [R]
**AN2823.3**	**ANIA_02823**	**0.44**		Has domain(s) with predicted ion channel activity, role in transport and integral to membrane localization	
**AN2827.3**	**ANIA_02827**	**0.36**		Putative potassium-transporting ATPase with a predicted role in energy metabolism	Calcium transporting ATPase [P]

**AN2838.3**	**ANIA_02838**	**0.19**		Ortholog of Aspergillus glaucus : Aspgl1_0163409, Neosartorya fischeri NRRL 181 : NFIA_097910, Aspergillus clavatus NRRL 1 : ACLA_058550 and Aspergillus zonatus : Aspzo1_0133076	
**AN2850.3**	**ANIA_02850**	**0.47**		Has domain(s) with predicted transferase activity, transferring phosphorus-containing groups, zinc ion binding activity	Chromatin remodeling protein, contains PHD Zn-finger [B]
**AN2863.3**	**ANIA_02863**	**0.28**		Ortholog of A. fumigatus Af293 : Afu3g11850, A. niger CBS 513.88 : An02g07670, A. oryzae RIB40 : AO090003000748, Neosartorya fischeri NRRL 181 : NFIA_065470 and Aspergillus wentii : Aspwe1_0037077	
**AN2877.3**	**ANIA_02877**	**0.40**		Ortholog(s) have oxysterol binding, phosphatidic acid binding, phosphatidylinositol-4,5-bisphosphate binding, phosphatidylinositol-4-phosphate binding, sterol transporter activity	Oxysterol-binding protein [T]
**AN2884.3**	**ANIA_02884**	**0.42**		Ortholog of A. fumigatus Af293 : Afu3g11620, A. niger CBS 513.88 : An02g07410, Aspergillus wentii : Aspwe1_0736090, Aspergillus sydowii : Aspsy1_0776302 and Aspergillus terreus NIH2624 : ATET_04698	
**AN2894.3**	**ANIA_02894**	**0.39**		Ortholog of A. fumigatus Af293 : Afu3g11550, A. niger CBS 513.88 : An02g07350, A. oryzae RIB40 : AO090003000710, Aspergillus wentii : Aspwe1_0025131 and Aspergillus sydowii : Aspsy1_0775782	
**AN2933.3**	**ANIA_02933**	**0.49**		Ortholog of A. fumigatus Af293 : Afu3g08170, A. niger CBS 513.88 : An02g11690, A. oryzae RIB40 : AO090005001460, Aspergillus wentii : Aspwe1_0113955 and Aspergillus sydowii : Aspsy1_0029366	Phosphatidylserine-specific receptor PtdSerR, contains JmjC domain [B, T]
**AN2936.3**	**ANIA_02936**	**0.44**		Putative Class 2C alpha-mannosidase with a predicted role in mannose polymer metabolism	Alpha-mannosidase [G]
**AN2973.3**	**ANIA_02973**	**0.47**		Ortholog(s) have DNA binding, DNA-dependent ATPase activity, chromatin binding activity	SNF2 family DNA-dependent ATPase [B]
**AN2974.3**	**ANIA_02974**	**0.35**		Has domain(s) with predicted substrate-specific transmembrane transporter activity, role in transmembrane transport and integral to membrane localization	Predicted transporter (major facilitator superfamily) [R]
**AN2980.3**	**ANIA_02980**	**0.37**		Ortholog(s) have structural constituent of ribosome activity, role in cytoplasmic translation and cytosolic large ribosomal subunit, nucleolus localization	60S ribosomal protein L35A/L37 [J]
**AN3006.3**	**ANIA_03006**	**0.26**		Ortholog of A. fumigatus Af293 : Afu4g13070, Neosartorya fischeri NRRL 181 : NFIA_103280, Aspergillus versicolor : Aspve1_0032308 and Aspergillus niger ATCC 1015 : 52618-mRNA	Predicted hydrolase/acyltransferase (alpha/beta hydrolase superfamily) [R]
**AN3023.3**	**ANIA_03023**	**0.39**		Ortholog of A. fumigatus Af293 : Afu3g08870, A. niger CBS 513.88 : An16g02440, A. oryzae RIB40 : AO090005001347, Neosartorya fischeri NRRL 181 : NFIA_068250 and Aspergillus wentii : Aspwe1_0041800	
**AN3034.3**	**ANIA_03034**	**0.49**	suAprgA1	Suppressor of prgA1 mutation; involved in regulation of penicillin biosynthesis; null mutation results in decreased expression of ipnA and acvA, and reduced penicillin production	MAM33, mitochondrial matrix glycoprotein [C]
**AN3038.3**	**ANIA_03038**	**0.49**		Ortholog(s) have nucleus localization	
**AN3057.3**	**ANIA_03057**	**0.50**		Has domain(s) with predicted oxidoreductase activity, acting on a sulfur group of donors, oxygen as acceptor activity and role in oxidation-reduction process, prenylcysteine catabolic process	Amine oxidase [Q]
**AN3059.3**	**ANIA_03059**	**0.48**	pgmA	Putative phosphoglycerate mutase with a predicted role in gluconeogenesis and glycolysis; expression upregulated after exposure to farnesol	Phosphoglycerate mutase [G]
**AN3070.3**	**ANIA_03070**	**0.39**		Ortholog(s) have chaperonin-containing T-complex, nucleus localization	Chaperonin complex component, TCP-1 zeta subunit (CCT6) [O]
**AN3092.3**	**ANIA_03092**	**0.38**		Has domain(s) with predicted guanyl-nucleotide exchange factor activity, role in regulation of small GTPase mediated signal transduction, small GTPase mediated signal transduction and intracellular localization	cAMP-regulated guanine nucleotide exchange factor [T]
**AN3096.3**	**ANIA_03096**	**0.49**	rmtB	Putative transferase with a predicted role in histidine metabolism; arginine methyltransferase for histones H4, H3, and H2A	Protein arginine N-methyltransferase PRMT1 and related enzymes [O, K, T]
**AN3104.3**	**ANIA_03104**	**0.46**		Putative allantoicase with a predicted role in purine metabolism	Allantoicase [F]
**AN3156.3**	**ANIA_03156**	**0.32**		Ortholog(s) have role in mitotic cell cycle checkpoint, regulation of translational initiation in response to osmotic stress, stress granule disassembly	Translation initiation factor 2, alpha subunit (eIF-2alpha) [J]
**AN3171.3**	**ANIA_03171**	**0.42**		Has domain(s) with predicted GTP binding, GTPase activity, role in GTP catabolic process, protein transport, small GTPase mediated signal transduction and intracellular, membrane localization	Ras-related small GTPase, Rho type [R]
**AN3172.3**	**ANIA_03172**	**0.46**		Ortholog of S. cerevisiae RPS0A and RPS0B; expression reduced after exposure to farnesol	40S ribosomal protein SA (P40)/Laminin receptor 1 [J]
**AN3174.3**	**ANIA_03174**	**0.40**		Ortholog of Aspergillus sydowii : Aspsy1_0087161	
**AN3190.3**	**ANIA_03190**	**0.37**		Ortholog of A. fumigatus Af293 : Afu4g02920, A. niger CBS 513.88 : An14g02990, A. oryzae RIB40 : AO090001000192, Aspergillus wentii : Aspwe1_0061505 and Aspergillus sydowii : Aspsy1_1159424	Predicted membrane protein [S]
**AN3200.3**	**ANIA_03200**	**0.38**		Putative beta-glucuronidase with a predicted role in polysaccharide degradation; the predicted pseudogene AN10383 and predicted ORF AN3200 occupy overlapping chromosomal locations in version 4 of the A. nidulans annotation	Beta-Glucuronidase GUSB (glycosylhydrolase superfamily 2) [G]
**AN3201.3**	**ANIA_03201**	**0.50**	lacD	Putative beta-galactosidase with a predicted role in lactose metabolism	
**AN3214.3**	**ANIA_03214**	**0.09**	hk-8-5	Putative histidine-containing phosphotransfer protein	Sensory transduction histidine kinase [T]
**AN3219.3**	**ANIA_03219**	**0.16**		Protein of unknown function	
**AN3224.3**	**ANIA_03224**	**0.46**		Ortholog(s) have activating transcription factor binding, core RNA polymerase II recruiting transcription factor activity, structural molecule activity	
**AN3233.3**	**ANIA_03233**	**0.47**		Has domain(s) with predicted substrate-specific transmembrane transporter activity, role in transmembrane transport and integral to membrane localization	Predicted transporter (major facilitator superfamily) [R]
**AN3243.3**	**ANIA_03243**	**0.35**		Ortholog of A. niger CBS 513.88 : An11g08150, Neosartorya fischeri NRRL 181 : NFIA_040880, Aspergillus wentii : Aspwe1_0036368 and Aspergillus versicolor : Aspve1_0033884	
**AN3247.3**	**ANIA_03247**	**0.34**		Has domain(s) with predicted ATP binding, ATPase activity, coupled to transmembrane movement of substances activity, role in transport and integral to membrane localization	Pleiotropic drug resistance proteins (PDR1-15), ABC superfamily [Q]
**AN3249.3**	**ANIA_03249**	**0.21**		Has domain(s) with predicted nucleotide binding, oxidoreductase activity and role in metabolic process	Hydroxysteroid 17-beta dehydrogenase 11 [Q]
**AN3281.3**	**ANIA_03281**	**0.47**	CYP567D1	Putative cytochrome P450	Cytochrome P450 CYP3/CYP5/CYP6/CYP9 subfamilies [Q]
**AN3301.3**	**ANIA_03301**	**0.37**		Has domain(s) with predicted role in transmembrane transport and integral to membrane localization	Predicted transporter (major facilitator superfamily) [R]
**AN3314.3**	**ANIA_03314**	**0.28**		Has domain(s) with predicted carbon-sulfur lyase activity and role in metabolic process	
**AN3322.3**	**ANIA_03322**	**0.08**		Ortholog of A. fumigatus Af293 : Afu5g14570, Neosartorya fischeri NRRL 181 : NFIA_072390 and Aspergillus fumigatus A1163 : AFUB_062250	
**AN3326.3**	**ANIA_03326**	**0.45**		Putative enodomannanase	
**AN3327.3**	**ANIA_03327**	**0.31**		Protein of unknown function	
**AN3334.3**	**ANIA_03334**	**0.25**		Has domain(s) with predicted nucleotide binding activity	
**AN3361.3**	**ANIA_03361**	**0.44**	nopA	Bacterial rhodopsin family G-protein coupled receptor-like protein	
**AN3383.3**	**ANIA_03383**	**0.40**		Has domain(s) with predicted role in transmembrane transport and integral to membrane localization	Predicted transporter (major facilitator superfamily) [R]
**AN3386.3**	**ANIA_03386**	**0.14**		Putative polyketide synthase; involved in secondary metabolite production	Animal-type fatty acid synthase and related proteins [I]
**AN3392.3**	**ANIA_03392**	**0.38**		Has domain(s) with predicted nucleotide binding, oxidoreductase activity and role in metabolic process	Kynurenine 3-monooxygenase and related flavoprotein monooxygenases [C, R]
**AN3425.3**	**ANIA_03425**	**0.23**		Ortholog(s) have cytosol localization	Interferon-related protein PC4 like [Z]

**AN3437.3**	**ANIA_03437**	**0.20**	apsB	Coiled-coil protein of the spindle pole body involved in distribution of nuclei in hyphae and conidiophores; required for entry of nuclei into sterigmata; mutants show uneven distribution and chaotic movement of nuclei; mutants self-sterile	Myosin class II heavy chain [Z]
**AN3474.3**	**ANIA_03474**	**0.29**		Ortholog of A. oryzae RIB40 : AO090003001515, Aspergillus flavus NRRL 3357 : AFL2T_01577 and Aspergillus wentii : Aspwe1_0071034	
**AN3477.3**	**ANIA_03477**	**0.39**		Ortholog of Aspergillus niger ATCC 1015 : 136740-mRNA	
**AN3480.3**	**ANIA_03480**	**0.09**		Ortholog of A. niger CBS 513.88 : An11g08860, Neosartorya fischeri NRRL 181 : NFIA_008010, Aspergillus niger ATCC 1015 : 48080-mRNA and Aspergillus sydowii : Aspsy1_0166071	
**AN3484.3**	**ANIA_03484**	**0.21**		Ortholog of Aspergillus acidus : Aspfo1_0160947	
**AN3485.3**	**ANIA_03485**	**0.17**		Ortholog of Aspergillus acidus : Aspfo1_0060189	
**AN3486.3**	**ANIA_03486**	**0.47**		Ortholog of A. nidulans FGSC A4 : AN7279, A. fumigatus Af293 : Afu1g01220, Afu1g01300, A. niger CBS 513.88 : An09g02890, An01g01360 and A. oryzae RIB40 : AO090102000097, AO090010000040/aoiA	
**AN3498.3**	**ANIA_03498**	**0.50**		Has domain(s) with predicted substrate-specific transmembrane transporter activity, role in transmembrane transport and integral to membrane localization	Predicted transporter (major facilitator superfamily) [R]
**AN3501.3**	**ANIA_03501**	**0.49**		Putative transcription factor; predicted role in secondary metabolite production	
**AN3512.3**	**ANIA_03512**	**0.37**		Ortholog of Aspergillus brasiliensis : Aspbr1_0049104, Aspergillus glaucus : Aspgl1_0060098, Aspergillus flavus NRRL 3357 : AFL2T_08754 and Neosartorya fischeri NRRL 181 : NFIA_073230	
**AN3523.3**	**ANIA_03523**	**0.46**		Ortholog of A. nidulans FGSC A4 : AN10421, A. fumigatus Af293 : Afu1g01460, Afu4g01090, Afu6g13470, Afu8g06620 and A. niger CBS 513.88 : An08g03030	Predicted Zn-dependent hydrolase (beta-lactamase superfamily) [R]
**AN3528.3**	**ANIA_03528**	**0.37**		Ortholog of A. nidulans FGSC A4 : AN7149, A. fumigatus Af293 : Afu4g03620, Afu6g02580, A. niger CBS 513.88 : An14g01630, An04g09610 and A. oryzae RIB40 : AO090113000188, AO090011000275, AO090023000922	
**AN3539.3**	**ANIA_03539**	**0.18**		Ortholog of A. nidulans FGSC A4 : AN3540, A. fumigatus Af293 : Afu8g00120, A. oryzae RIB40 : AO090038000188, AO090026000095 and Neosartorya fischeri NRRL 181 : NFIA_032270	
**AN3540.3**	**ANIA_03540**	**0.26**		Ortholog of A. nidulans FGSC A4 : AN3539, A. fumigatus Af293 : Afu8g00120, A. oryzae RIB40 : AO090038000188, AO090026000095 and Neosartorya fischeri NRRL 181 : NFIA_032270	
**AN3549.3**	**ANIA_03549**	**0.22**		Protein of unknown function; transcript repressed by nitrate	HMG-box K factor [K]
**AN3565.3**	**ANIA_03565**	**0.42**		Has domain(s) with predicted hydrolase activity	
**AN3566.3**	**ANIA_03566**	**0.24**	mns1C	Putative alpha-mannosidase with a predicted role in mannose polymer metabolism	1, 2-alpha-mannosidase [G]
**AN3584.3**	**ANIA_03584**	**0.34**		Ortholog(s) have phosphatidylinositol-3-phosphate binding activity and role in CVT pathway, early endosome to Golgi transport, mitochondrion degradation, piecemeal microautophagy of nucleus	Membrane coat complex Retromer, subunit VPS5/SNX1, Sorting nexins, and related PX domain-containing proteins [U]
**AN3591.3**	**ANIA_03591**	**0.19**		Protein with similarity to bacterial propionyl-CoA-yielding methylmalonate semialdehyde dehydrogenase; may be involved in isoleucine and valine catabolism	Aldehyde dehydrogenase [C]
**AN3593.3**	**ANIA_03593**	**0.45**		Ortholog(s) have methylthioribulose 1-phosphate dehydratase activity, role in L-methionine salvage from methylthioadenosine and cytoplasm localization	Class II aldolase/adducin N-terminal domain protein [G]
**AN3601.3**	**ANIA_03601**	**0.44**		Has domain(s) with predicted catalytic activity and role in metabolic process	Predicted metal-dependent hydrolase of the TIM-barrel fold [R]
**AN3643.3**	**ANIA_03643**	**0.25**		Ortholog(s) have guanyl-nucleotide exchange factor activity, role in Golgi to endosome transport, endocytosis, protein targeting to vacuole and Golgi apparatus, cytosol, endosome, extrinsic to membrane, trans-Golgi network localization	Uncharacterized conserved protein [S]
**AN3658.3**	**ANIA_03658**	**0.16**		Has domain(s) with predicted role in transmembrane transport and integral to membrane localization	Monocarboxylate transporter [G]
**AN3677.3**	**ANIA_03677**	**0.36**		Ortholog(s) have cytosol, nucleus localization	Asparagine synthase [E]
**AN3679.3**	**ANIA_03679**	**0.34**		Ortholog(s) have cytosol, nucleus localization	Reductases with broad range of substrate specificities [R]
**AN3684.3**	**ANIA_03684**	**0.47**		Has domain(s) with predicted DNA binding, sequence-specific DNA binding RNA polymerase II transcription factor activity, zinc ion binding activity and role in regulation of transcription, DNA-dependent, transcription, DNA-templated	
**AN3703.3**	**ANIA_03703**	**0.14**		Ortholog of A. fumigatus Af293 : Afu3g02990, Neosartorya fischeri NRRL 181 : NFIA_004810, Aspergillus wentii : Aspwe1_0643479 and Aspergillus versicolor : Aspve1_0042072	
**AN3704.3**	**ANIA_03704**	**0.49**		Has domain(s) with predicted catalytic activity, pyridoxal phosphate binding activity and role in biosynthetic process	1-aminocyclopropane-1-carboxylate synthase, and related proteins [T]
**AN3708.3**	**ANIA_03708**	**0.44**		Ortholog(s) have hydrolase activity, role in nucleotide metabolic process and cytoplasm, nucleus localization	Zinc-binding protein of the histidine triad (HIT) family [T]
**AN3736.3**	**ANIA_03736**	**0.34**		Ortholog of A. fumigatus Af293 : Afu6g12320, A. niger CBS 513.88 : An06g01470, A. oryzae RIB40 : AO090009000187, Aspergillus wentii : Aspwe1_0144945 and Aspergillus sydowii : Aspsy1_0044136	
**AN3757.3**	**ANIA_03757**	**0.34**		Ortholog(s) have role in establishment of cell polarity, mycelium development, spore germination and endoplasmic reticulum, hyphal tip, plasma membrane localization	Predicted alpha/beta hydrolase BEM46 [R]
**AN3771.3**	**ANIA_03771**	**0.49**		Ortholog(s) have cytoplasm localization	Predicted coiled-coil protein [R]
**AN3776.3**	**ANIA_03776**	**0.47**		Has domain(s) with predicted role in transmembrane transport and integral to membrane localization	Permease of the major facilitator superfamily [G]
**AN3779.3**	**ANIA_03779**	**0.28**		Ortholog(s) have exopolyphosphatase activity, role in polyphosphate catabolic process and cytosol, nucleus localization	Exopolyphosphatases and related proteins [C]
**AN3781.3**	**ANIA_03781**	**0.15**		Has domain(s) with predicted inorganic phosphate transmembrane transporter activity, role in phosphate ion transport and membrane localization	Na+/Pi symporter [P]
**AN3782.3**	**ANIA_03782**	**0.35**		Has domain(s) with predicted oleate hydratase activity and role in fatty acid metabolic process	
**AN3810.3**	**ANIA_03810**	**0.44**		Ortholog(s) have CTD phosphatase activity, protein tyrosine phosphatase activity	Protein involved in K start site selection [K]
**AN3828.3**	**ANIA_03828**	**0.31**		Has domain(s) with predicted zinc ion binding activity and intracellular localization	
**AN3839.3**	**ANIA_03839**	**0.44**	swoF	Putative glycylpeptide N-tetradecanoyltransferase involved in control of polar growth; predicted role in protein, peptide, and amino acid metabolism	N-myristoyl transferase [I]
**AN3864.3**	**ANIA_03864**	**0.45**	nxtA	Putative homologue of the human NTF2-like export factor 1, p15/NXT1,	RNA export factor NXT1 [A]
**AN3870.3**	**ANIA_03870**	**0.49**		Has domain(s) with predicted oxidoreductase activity and role in oxidation-reduction process	FAD-dependent oxidoreductase [R]
**AN3886.3**	**ANIA_03886**	**0.46**		Protein of unknown function	
**AN3898.3**	**ANIA_03898**	**0.50**		Ortholog of A. fumigatus Af293 : Afu3g14180, A. niger CBS 513.88 : An09g04380, A. oryzae RIB40 : AO090012000661 and Aspergillus wentii : Aspwe1_0038053	
**AN3905.3**	**ANIA_03905**	**0.37**		Ortholog(s) have cytosol, nucleus localization	Phosphotyrosyl phosphatase activator [D, T]
**AN3910.3**	**ANIA_03910**	**0.41**		Ortholog(s) have role in attachment of telomeric heterochromatin to nuclear envelope, maintenance of rDNA, mitotic sister chromatid segregation, nuclear envelope organization and endoplasmic reticulum, nuclear envelope localization	
**AN3920.3**	**ANIA_03920**	**0.50**		Ortholog(s) have cytosol, nucleus localization	Protoporphyrinogen oxidase [H]
**AN3922.3**	**ANIA_03922**	**0.37**		Ortholog(s) have mitochondrion, plasma membrane localization	Ubiquinol cytochrome c oxidoreductase, subunit QCR9 [C]

**AN3952.3**	**ANIA_03952**	**0.31**		Has domain(s) with predicted ATP binding, ATPase activity, coupled to transmembrane movement of substances activity, role in transport and integral to membrane localization	Pleiotropic drug resistance proteins (PDR1-15), ABC superfamily [Q]
**AN3955.3**	**ANIA_03955**	**0.43**		Ortholog(s) have role in RNA metabolic process and cytoplasmic stress granule, nucleus, ribosome localization	Uncharacterized conserved protein [S]
**AN3965.3**	**ANIA_03965**	**0.30**		Has domain(s) with predicted hydrolase activity, acting on carbon-nitrogen (but not peptide) bonds, in linear amidines, metal ion binding activity	
**AN3966.3**	**ANIA_03966**	**0.42**		Protein of unknown function	
**AN3968.3**	**ANIA_03968**	**0.14**		Protein of unknown function; transcript is induced by nitrate	
**AN3973.3**	**ANIA_03973**	**0.26**		Putative peroxiredoxin	Alkyl hydroperoxide reductase, thiol specific antioxidant and related enzymes [O]
**AN3976.3**	**ANIA_03976**	**0.44**		Has domain(s) with predicted nucleotide binding activity	
**AN3992.3**	**ANIA_03992**	**0.27**		Ortholog of A. nidulans FGSC A4 : AN8424, AN7089, A. fumigatus Af293 : Afu7g05085, A. oryzae RIB40 : AO090005000321 and Neosartorya fischeri NRRL 181 : NFIA_026200	
**AN3993.3**	**ANIA_03993**	**0.50**		Ortholog of A. nidulans FGSC A4 : AN11049, A. fumigatus Af293 : Afu3g01790, Afu6g11770, A. niger CBS 513.88 : An02g08800 and A. oryzae RIB40 : AO090166000059, AO090010000315	
**AN3994.3**	**ANIA_03994**	**0.18**		Ortholog of A. nidulans FGSC A4 : AN5182, AN2782, A. fumigatus Af293 : Afu1g01650, Afu2g00470, Afu6g07130, Afu7g00770, A. niger CBS 513.88 : An15g02620 and A. oryzae RIB40 : AO090005000527, AO090012000936	
**AN4005.3**	**ANIA_04005**	**0.15**		Ortholog of A. fumigatus Af293 : Afu3g05800 and Aspergillus fumigatus A1163 : AFUB_043230	
**AN4011.3**	**ANIA_04011**	**0.44**		Putative 5'-phosphoribosyl-4-(N-succinocarboxamide)-5-aminoimidazole lyase with a predicted role in purine metabolism	Adenylosuccinate lyase [F]
**AN4018.3**	**ANIA_04018**	**0.41**		Ortholog(s) have role in protein targeting to vacuole	Transferrin receptor and related proteins containing the protease-associated (PA) domain [O, P, R]
**AN4037.3**	**ANIA_04037**	**0.47**		Ortholog(s) have role in response to temperature stimulus	Splicing factor 3b, subunit 3 [A]
**AN4039.3**	**ANIA_04039**	**0.48**		Ortholog(s) have endoplasmic reticulum, mitochondrial outer membrane localization	Rhodanese-related sulfurtransferase [P]
**AN4040.3**	**ANIA_04040**	**0.46**		Ortholog of A. nidulans FGSC A4 : AN7213, A. fumigatus Af293 : Afu5g00960 and A. niger CBS 513.88 : An18g01180, An10g00910, An11g01220, An01g11560	
**AN4058.3**	**ANIA_04058**	**0.49**		Putative dihydroxy acid dehydratase with a predicted role in branched-chain amino acid biosynthesis	Dihydroxy-acid dehydratase [E]
**AN4066.3**	**ANIA_04066**	**0.39**		Ortholog(s) have role in cellular response to drug	Putative GTPase activating proteins (GAPs) [T]
**AN4085.3**	**ANIA_04085**	**0.46**		Ortholog(s) have role in asymmetric protein localization to old mitotic spindle pole body, establishment or maintenance of cytoskeleton polarity, negative regulation of septation initiation signaling	Protein phosphatase 2A regulatory subunit A and related proteins [T]
**AN4100.3**	**ANIA_04100**	**0.44**		Ortholog of A. nidulans FGSC A4 : AN10579, A. fumigatus Af293 : Afu8g06630, A. niger CBS 513.88 : An05g01150, Aspergillus wentii : Aspwe1_0169274 and Aspergillus sydowii : Aspsy1_0065818, Aspsy1_0089096	
**AN4104.3**	**ANIA_04104**	**0.34**		Ortholog of A. fumigatus Af293 : Afu1g05860, A. niger CBS 513.88 : An18g03590, A. oryzae RIB40 : AO090009000354, Aspergillus wentii : Aspwe1_0026045 and Aspergillus sydowii : Aspsy1_0089091	
**AN4106.3**	**ANIA_04106**	**0.34**		Has domain(s) with predicted role in transmembrane transport and integral to membrane localization	Permease of the major facilitator superfamily [G]
**AN4112.3**	**ANIA_04112**	**0.18**		Ortholog of A. fumigatus Af293 : Afu1g05840, A. niger CBS 513.88 : An18g03610, A. oryzae RIB40 : AO090009000350, Aspergillus wentii : Aspwe1_0038192 and Aspergillus sydowii : Aspsy1_0089097	
**AN4115.3**	**ANIA_04115**	**0.18**		Ortholog of Aspergillus versicolor : Aspve1_0037421, Aspergillus sydowii : Aspsy1_0060570 and Aspergillus aculeatus ATCC16872 : Aacu16872_055617	
**AN4116.3**	**ANIA_04116**	**0.10**		Protein of unknown function	
**AN4123.3**	**ANIA_04123**	**0.48**		Has domain(s) with predicted sequence-specific DNA binding RNA polymerase II transcription factor activity, zinc ion binding activity, role in regulation of transcription, DNA-dependent and nucleus localization	
**AN4129.3**	**ANIA_04129**	**0.37**		Has domain(s) with predicted role in transmembrane transport and integral to membrane localization	Permease of the major facilitator superfamily [G]
**AN4133.3**	**ANIA_04133**	**0.48**		Has domain(s) with predicted nucleic acid binding, zinc ion binding activity	
**AN4154.3**	**ANIA_04154**	**0.44**		Has domain(s) with predicted 1-alkyl-2-acetylglycerophosphocholine esterase activity and role in lipid catabolic process	Phospholipase A2 (platelet-activating factor acetylhydrolase in humans) [I]
**AN4159.3**	**ANIA_04159**	**0.21**	glnA	Putative glutamate-ammonia ligase with a predicted role in glutamate and glutamine metabolism; intracellular; transcript upregulated by nitrate limitation; protein abundance decreased by menadione stress and induced by farnesol	Glutamine synthetase [E]
**AN4164.3**	**ANIA_04164**	**0.33**		Ortholog(s) have mitochondrion localization	Uncharacterized conserved protein [S]
**AN4174.3**	**ANIA_04174**	**0.26**		Putative lactoylglutathione lyase with a predicted role in the methylglyoxal bypass	Glyoxalase [G]
**AN4176.3**	**ANIA_04176**	**0.38**		Ortholog of A. niger CBS 513.88 : An15g01870, An19g00110, An12g05110, A. oryzae RIB40 : AO090009000510 and Aspergillus wentii : Aspwe1_0033361, Aspwe1_0034402	
**AN4177.3**	**ANIA_04177**	**0.22**		Has domain(s) with predicted catalytic activity, coenzyme binding, nucleotide binding activity and role in cellular metabolic process	
**AN4202.3**	**ANIA_04202**	**0.46**	rpl16a	Predicted ribosomal protein of the large (60S) ribosomal subunit; differentially expressed during sexual development	60S ribosomal protein L13a [J]
**AN4209.3**	**ANIA_04209**	**0.47**		Has domain(s) with predicted sugar:hydrogen symporter activity, role in carbohydrate transport and Golgi membrane, integral to membrane localization	Predicted integral membrane protein [R]
**AN4222.3**	**ANIA_04222**	**0.49**		Ortholog(s) have cytosol, nucleus, small-subunit processome localization	60S ribosomal protein L27 [J]
**AN4226.3**	**ANIA_04226**	**0.43**		Ortholog(s) have role in maturation of SSU-rRNA from tricistronic rRNA transcript (SSU-rRNA, 5.8S rRNA, LSU-rRNA) and 90S preribosome, nucleolus, small-subunit processome, spindle localization	Sof1-like rRNA processing protein (contains WD40 repeats) [A]
**AN4276.3**	**ANIA_04276**	**0.28**		Protein of unknown function	
**AN4277.3**	**ANIA_04277**	**0.28**		Ortholog(s) have glucose transmembrane transporter activity, role in cellular response to drug and plasma membrane localization	Predicted transporter (major facilitator superfamily) [R]
**AN4284.3**	**ANIA_04284**	**0.27**		Ortholog of A. fumigatus Af293 : Afu5g04336, A. niger CBS 513.88 : An09g06820, Neosartorya fischeri NRRL 181 : NFIA_037740 and Aspergillus versicolor : Aspve1_0047298	
**AN4292.3**	**ANIA_04292**	**0.41**		Possible pseudogene	
**AN4297.3**	**ANIA_04297**	**0.28**		Ortholog(s) have role in iron-sulfur cluster assembly, mitochondrial respiratory chain complex I assembly and mitochondrial respiratory chain complex I, peripheral segment, plasma membrane localization	NADH-ubiquinone oxidoreductase, NUFS7/PSST/20 kDa subunit [C]
**AN4299.3**	**ANIA_04299**	**0.20**		Putative clock-controlled and temperature-regulated gene; transcript induced by light in in developmentally competent mycelia	
**AN4300.3**	**ANIA_04300**	**0.42**		NADH-ubiquinone oxidoreductase 21.3 kDa subunit; protein levels decrease in response to farnesol	
**AN4304.3**	**ANIA_04304**	**0.48**		Ortholog(s) have disulfide oxidoreductase activity, role in cellular response to oxidative stress, response to osmotic stress and mitochondrial matrix localization	Glutaredoxin-related protein [O]
**AN4315.3**	**ANIA_04315**	**0.32**		Ortholog(s) have role in regulation of translational elongation and cytosolic ribosome localization	ATPase component of ABC transporters with duplicated ATPase domains/Translation elongation factor EF-3b [E, J]
**AN4352.3**	**ANIA_04352**	**0.28**		Ortholog(s) have mitochondrion, plasma membrane localization	Mitochondrial carnitine-acylcarnitine carrier protein [C]

**AN4365.3**	**ANIA_04365**	**0.40**		Ortholog(s) have role in meiotic mismatch repair, reciprocal meiotic recombination and MutLbeta complex localization	DNA mismatch repair protein - MLH3 family [L]
**AN4388.3**	**ANIA_04388**	**0.34**		Putative ubiquinol-cytochrome-c reductase subunit with a predicted role in energy metabolism	Ubiquinol cytochrome c reductase, subunit QCR7 [C]
**AN4428.3**	**ANIA_04428**	**0.50**		Ortholog(s) have endoplasmic reticulum localization	Amino acid transporter protein [E]
**AN4444.3**	**ANIA_04444**	**0.41**		Protein of unknown function	
**AN4449.3**	**ANIA_04449**	**0.49**		Ortholog(s) have endopeptidase activator activity and role in proteasomal ubiquitin-independent protein catabolic process, proteasome-mediated ubiquitin-dependent protein catabolic process	20S proteasome, regulatory subunit beta type PSMB3/PUP3 [O]
**AN4471.3**	**ANIA_04471**	**0.40**		Ortholog(s) have role in maturation of 5.8S rRNA from tricistronic rRNA transcript (SSU-rRNA, 5.8S rRNA, LSU-rRNA), maturation of LSU-rRNA from tricistronic rRNA transcript (SSU-rRNA, 5.8S rRNA, LSU-rRNA), ribosomal large subunit assembly	WD40 repeat-containing protein [S]
**AN4473.3**	**ANIA_04473**	**0.26**		Ortholog of A. nidulans FGSC A4 : AN5608, A. fumigatus Af293 : Afu4g07720, A. niger CBS 513.88 : An14g07030, An04g02100 and A. oryzae RIB40 : AO090023000800, AO090009000070	Carboxylesterase and related proteins [R]
**AN4474.3**	**ANIA_04474**	**0.49**		Ortholog of A. niger CBS 513.88 : An04g02110, A. oryzae RIB40 : AO090023000799, Neosartorya fischeri NRRL 181 : NFIA_108450 and Aspergillus versicolor : Aspve1_0133959	
**AN4486.3**	**ANIA_04486**	**0.47**	leuB	Putative transcriptional activator with a predicted role in leucine biosynthesis; locus contains the conserved upstream open reading frame (uORF) AN4486-uORF	
**AN4510.3**	**ANIA_04510**	**0.29**	fbxA	F-box protein; mutant shows increased resistance to 2-deoxyglucose; BLAST indicates no apparent homologs in higher eukaryotes	Cyclic nucleotide-gated cation channel CNGA1-3 and related proteins [P, T]
**AN4548.3**	**ANIA_04548**	**0.43**		Ortholog(s) have mitochondrion localization	
**AN4553.3**	**ANIA_04553**	**0.34**		Ortholog(s) have mitochondrial outer membrane, plasma membrane localization	Uncharacterized conserved protein [S]
**AN4561.3**	**ANIA_04561**	**0.49**	phnB	Phosducin-like protein	Conserved phosducin-like protein [T]
**AN4594.3**	**ANIA_04594**	**0.37**		Ortholog(s) have role in maturation of SSU-rRNA from tricistronic rRNA transcript (SSU-rRNA, 5.8S rRNA, LSU-rRNA) and cytosolic small ribosomal subunit localization	40S ribosomal protein S20 [J]
**AN4606.3**	**ANIA_04606**	**0.47**		Has domain(s) with predicted sequence-specific DNA binding RNA polymerase II transcription factor activity, zinc ion binding activity, role in regulation of transcription, DNA-dependent and nucleus localization	
**AN4614.3**	**ANIA_04614**	**0.41**		Ortholog(s) have role in cellular response to cadmium ion, cellular response to calcium ion, detoxification of cadmium ion, response to drug and RAVE complex, cytosol, nucleus localization	
**AN4619.3**	**ANIA_04619**	**0.38**		Ortholog of Aspergillus versicolor : Aspve1_0073054 and Aspergillus sydowii : Aspsy1_0070056	
**AN4629.3**	**ANIA_04629**	**0.42**		Putative unsaturated glucuronyl hydrolase	
**AN4630.3**	**ANIA_04630**	**0.46**		Has domain(s) with predicted substrate-specific transmembrane transporter activity, role in transmembrane transport and integral to membrane localization	Predicted transporter (major facilitator superfamily) [R]
**AN4636.3**	**ANIA_04636**	**0.43**		Ortholog(s) have lipid particle localization	
**AN4645.3**	**ANIA_04645**	**0.35**		Protein of unknown function	
**AN4646.3**	**ANIA_04646**	**0.41**		Ortholog of A. niger CBS 513.88 : An07g05420, Aspergillus versicolor : Aspve1_0042823, Aspergillus niger ATCC 1015 : 39924-mRNA and Aspergillus sydowii : Aspsy1_0155641	
**AN4661.3**	**ANIA_04661**	**0.30**		Protein of unknown function	Acyl-CoA synthetase [I]
**AN4670.3**	**ANIA_04670**	**0.46**	acdX	Subunit of the SAGA transcriptional regulatory complex; mutations abolish acetate repression; WD repeat protein; locus contains the conserved upstream open reading frame (uORF) AN4670-uORF	WD40 repeat-containing protein [R]
**AN4672.3**	**ANIA_04672**	**0.48**		Ortholog(s) have U4/U6 x U5 tri-snRNP complex, cytosol localization	Predicted PRP38-like splicing factor [S]
**AN4682.3**	**ANIA_04682**	**0.43**		Ortholog(s) have ubiquinone binding activity	
**AN4685.3**	**ANIA_04685**	**0.46**		Predicted Ras GTPase	Ras-related GTPase [R]
**AN4689.3**	**ANIA_04689**	**0.39**		Ortholog of A. fumigatus Af293 : Afu5g08920, A. niger CBS 513.88 : An07g04290, A. oryzae RIB40 : AO090020000494, Aspergillus wentii : Aspwe1_0167662 and Aspergillus sydowii : Aspsy1_0091330	
**AN4704.3**	**ANIA_04704**	**0.29**		Protein of unknown function	
**AN4757.3**	**ANIA_04757**	**0.42**		Ortholog(s) have role in cellular response to cadmium ion, detoxification of cadmium ion, ubiquinone biosynthetic process and mitochondrial inner membrane localization	Protein involved in ubiquinone biosynthesis [H]
**AN4762.3**	**ANIA_04762**	**0.49**		Ortholog(s) have mitochondrion localization	
**AN4774.3**	**ANIA_04774**	**0.44**		Ortholog(s) have uroporphyrin-III C-methyltransferase activity and role in cellular response to cadmium ion, cellular response to drug, detoxification of cadmium ion, methionine biosynthetic process, siroheme biosynthetic process	Uroporphyrin III methyltransferase [H]
**AN4777.3**	**ANIA_04777**	**0.39**		Ortholog(s) have structural constituent of ribosome activity	40s ribosomal protein S27 [J]
**AN4780.3**	**ANIA_04780**	**0.32**		Has domain(s) with predicted FMN binding, pyridoxamine-phosphate oxidase activity and role in oxidation-reduction process	
**AN4786.3**	**ANIA_04786**	**0.10**		Ortholog of A. fumigatus Af293 : Afu3g06750, A. niger CBS 513.88 : An11g09580, A. oryzae RIB40 : AO090020000322, Aspergillus wentii : Aspwe1_0562759 and Aspergillus sydowii : Aspsy1_0147721	
**AN4788.3**	**ANIA_04788**	**0.22**		Has domain(s) with predicted nucleic acid binding, zinc ion binding activity	RNA splicing factor - Slu7p [A]
**AN4802.3**	**ANIA_04802**	**0.31**		60S ribosomal protein L21; ortholog of S. cerevisiae Rpl21Ap; expression reduced after exposure to farnesol	60S ribosomal protein L21 [J]
**AN4829.3**	**ANIA_04829**	**0.39**		Putative reductase with a predicted role in carbohydrate metabolism	Aldo/keto reductase family proteins [R]
**AN4830.3**	**ANIA_04830**	**0.36**		Ortholog(s) have role in coenzyme A biosynthetic process and cytosol, nucleus localization	Uncharacterized conserved protein with similarity to phosphopantothenoylcysteine synthetase/decarboxylase [R]
**AN4832.3**	**ANIA_04832**	**0.33**		Ortholog of A. fumigatus Af293 : Afu3g07240, A. niger CBS 513.88 : An02g13480/atg29, A. oryzae RIB40 : AO090020000200, Aspergillus wentii : Aspwe1_0042020 and Aspergillus sydowii : Aspsy1_0148063	
**AN4857.3**	**ANIA_04857**	**0.47**		Has domain(s) with predicted transferase activity, transferring phosphorus-containing groups activity	Predicted unusual protein kinase [R]
**AN4861.3**	**ANIA_04861**	**0.39**		Ortholog(s) have RNA polymerase II core binding, RNA polymerase II regulatory region DNA binding, core RNA polymerase II recruiting transcription factor activity	K elongation factor TFIIS/Cofactor of enhancer-binding protein Sp1 [K]
**AN4862.3**	**ANIA_04862**	**0.47**	ranGAP	Putative Ran GTPase activating protein (GAP) with homology to Saccharomyces cerevisiae Rna1p; endogenously tagged RanGAP is excluded from nuclei during interphase and disperses throughout the cell at mitosis	Ran GTPase-activating protein [A, Y, T]
**AN4865.3**	**ANIA_04865**	**0.33**		Has domain(s) with predicted nucleic acid binding, nucleotide binding activity	Apoptosis-promoting RNA-binding protein TIA-1/TIAR (RRM superfamily) [A, J]
**AN4868.3**	**ANIA_04868**	**0.47**		Ortholog of A. fumigatus Af293 : Afu3g11310, A. niger CBS 513.88 : An02g07050, Aspergillus wentii : Aspwe1_0037013, Aspergillus sydowii : Aspsy1_0029674 and Aspergillus glaucus : Aspgl1_0045568	
**AN4883.3**	**ANIA_04883**	**0.44**		Ortholog(s) have nucleus localization	ATP-dependent DNA ligase I [L]
**AN4901.3**	**ANIA_04901**	**0.43**		Putative glutaminase A with a predicted role in glutamate and glutamine metabolism	
**AN4905.3**	**ANIA_04905**	**0.45**	gstA	theta class glutathione S-transferase; involved in resistance to a variety of xenobiotics and metals; confers susceptibility to the systemic fungicide carboxin	Glutathione S-transferase [O]
**AN4908.3**	**ANIA_04908**	**0.48**		Putative eukaryotic translation initiation factor subunit; PalA-dependent expression independent of pH	Uncharacterized protein CLU1/cluA/TIF31 involved in mitochondrial morphology/distribution, also found associated with eIF-3 [R]
**AN4913.3**	**ANIA_04913**	**0.31**	phk	Putative phosphoketolase with a predicted role in the phosphoketolase pathway	Transketolase [G]

**AN4940.3**	**ANIA_04940**	**0.24**		Ortholog(s) have role in cellular response to salt stress, reciprocal meiotic recombination and endoplasmic reticulum, mitochondrion, plasma membrane localization	
**AN4941.3**	**ANIA_04941**	**0.36**		Ortholog of A. fumigatus Af293 : Afu3g10470, A. niger CBS 513.88 : An16g04410, A. oryzae RIB40 : AO090003000593, Aspergillus wentii : Aspwe1_0169039 and Aspergillus sydowii : Aspsy1_0788739	
**AN4943.3**	**ANIA_04943**	**0.50**		Has domain(s) with predicted RNA binding activity and role in RNA processing	Predicted splicing regulator, contains RRM, SWAP and RPR domains [R]
**AN4946.3**	**ANIA_04946**	**0.35**		Ortholog of A. fumigatus Af293 : Afu3g10410, A. niger CBS 513.88 : An16g04340, A. oryzae RIB40 : AO090003000590, Aspergillus wentii : Aspwe1_0169033 and Aspergillus sydowii : Aspsy1_0087645	
**AN4966.3**	**ANIA_04966**	**0.46**		Ortholog(s) have endoplasmic reticulum localization	Uncharacterized conserved protein [S]
**AN4977.3**	**ANIA_04977**	**0.43**		Ortholog(s) have mitochondrion localization	Electron transfer flavoprotein ubiquinone oxidoreductase [C]
**AN4979.3**	**ANIA_04979**	**0.35**	folA	Predicted dihydroneopterin aldolase; predicted role in folic acid-containing compound metabolism; extragenic suppressor of a crzA null mutation; role in nuclear division cycle	Dihydropteroate synthase/7,8-dihydro-6-hydroxymethylpterin-pyrophosphokinase/Dihydroneopterin aldolase [H]
**AN4987.3**	**ANIA_04987**	**0.31**	pkaR	Putative protein kinase A (PKA) regulatory subunit	cAMP-dependent protein kinase types I and II, regulatory subunit [T]
**AN4988.3**	**ANIA_04988**	**0.32**		Ortholog(s) have intracellular localization	
**AN4993.3**	**ANIA_04993**	**0.45**	nnaC	Putative GNAT-type acetyltransferase	N-acetyltransferase [R]
**AN5004.3**	**ANIA_05004**	**0.19**		Transcript induced by light in in developmentally competent mycelia	
**AN5015.3**	**ANIA_05015**	**0.21**	conJ	Putative conidiation gene; transcript induced by light in in developmentally competent mycelia; double conF and conJ deletion results in increased cellular glycerol or erythritol leading to delayed germination and desiccation resistance	
**AN5022.3**	**ANIA_05022**	**0.41**		Dynactin p25 subunit; required for dynein/dynactin localization to the hyphal tip region and for interactions between dynein and the early endosome; contains several hexapeptide repeats	Dynactin, subunit p25 [Z]
**AN5029.3**	**ANIA_05029**	**0.49**		Has domain(s) with predicted substrate-specific transmembrane transporter activity, role in transmembrane transport and integral to membrane localization	Predicted transporter (major facilitator superfamily) [R]
**AN5039.3**	**ANIA_05039**	**0.37**		Ortholog of A. nidulans FGSC A4 : AN10386, A. niger CBS 513.88 : An09g05600, An13g01270 and A. oryzae RIB40 : AO090026000484, AO090005000917, AO090103000243, AO090005001067	
**AN5049.3**	**ANIA_05049**	**0.30**		Has domain(s) with predicted cation transmembrane transporter activity, role in cation transport, transmembrane transport and integral to membrane localization	Mitochondrial Fe2+ transporter MMT1 and related transporters (cation diffusion facilitator superfamily) [P]
**AN5063.3**	**ANIA_05063**	**0.31**		Ortholog of A. niger CBS 513.88 : An09g03770, Neosartorya fischeri NRRL 181 : NFIA_074500, Aspergillus sydowii : Aspsy1_0038892 and Aspergillus terreus NIH2624 : ATET_07074	
**AN5069.3**	**ANIA_05069**	**0.13**		Ortholog of A. nidulans FGSC A4 : AN2683, A. fumigatus Af293 : Afu5g02860, Afu5g14320, A. niger CBS 513.88 : An09g04830, An14g05730 and A. oryzae RIB40 : AO090102000482, AO090001000197	
**AN5071.3**	**ANIA_05071**	**0.34**		Protein of unknown function	
**AN5081.3**	**ANIA_05081**	**0.47**		Protein of unknown function	
**AN5083.3**	**ANIA_05083**	**0.26**		Has domain(s) with predicted hydrogen ion transmembrane transporter activity, role in ATP hydrolysis coupled proton transport and vacuolar proton-transporting V-type ATPase, V0 domain localization	Vacuolar H+-ATPase V0 sector, subunit a [C]
**AN5086.3**	**ANIA_05086**	**0.48**	SpoC1-C1D	Conidium-specific RNA of unknown function; member of the SpoC1 gene cluster	
**AN5088.3**	**ANIA_05088**	**0.19**		Putative potassium-transporting ATPase with a predicted role in energy metabolism	Calcium transporting ATPase [P]
**AN5089.3**	**ANIA_05089**	**0.25**		Ortholog of A. oryzae RIB40 : AO090103000089, Aspergillus flavus NRRL 3357 : AFL2T_12280, Aspergillus terreus NIH2624 : ATET_03032 and Aspergillus aculeatus ATCC16872 : Aacu16872_034601	
**AN5109.3**	**ANIA_05109**	**0.33**		Ortholog(s) have alditol:NADP+ 1-oxidoreductase activity, alpha-keto amide reductase activity, alpha-keto ester reductase activity and indole-3-acetaldehyde reductase (NADH) activity, more	Aldo/keto reductase family proteins [R]
**AN5117.3**	**ANIA_05117**	**0.11**		Protein of unknown function	
**AN5120.3**	**ANIA_05120**	**0.41**		Protein of unknown function; transcript is induced by nitrate; predicted NirA binding site in promoter	
**AN5122.3**	**ANIA_05122**	**0.42**		Putative kinase with a predicted role in nucleotide salvage pathways	Adenylate kinase [F]
**AN5123.3**	**ANIA_05123**	**0.32**		Has domain(s) with predicted nucleotide binding, oxidoreductase activity	Dimeric dihydrodiol dehydrogenase [G, Q]
**AN5141.3**	**ANIA_05141**	**0.38**		Ortholog of A. fumigatus Af293 : Afu1g07280, A. niger CBS 513.88 : An07g09830, A. oryzae RIB40 : AO090012000982, Aspergillus wentii : Aspwe1_0172497 and Aspergillus sydowii : Aspsy1_0033440	H/ACA small nucleolar RNP component GAR1 [J]
**AN5144.3**	**ANIA_05144**	**0.48**	pfkZ	Putative 6-phosphofructo-2-kinase with a predicted role in mannose/mannitol, fructose, and sorbose/sorbitol metabolism	Fructose-6-phosphate 2-kinase/fructose-2,6-biphosphatase [G]
**AN5154.3**	**ANIA_05154**	**0.31**		Ortholog of A. fumigatus Af293 : Afu1g07090, A. niger CBS 513.88 : An07g09710, A. oryzae RIB40 : AO090012000961, Aspergillus wentii : Aspwe1_0155743 and Aspergillus sydowii : Aspsy1_0134266	Nuclear protein, contains WD40 repeats [R]
**AN5157.3**	**ANIA_05157**	**0.42**		Ortholog of A. fumigatus Af293 : Afu1g07050, A. niger CBS 513.88 : An07g09610, A. oryzae RIB40 : AO090012000957, Aspergillus wentii : Aspwe1_0051655 and Aspergillus sydowii : Aspsy1_0091619	Proteins containing armadillo/beta-catenin-like repeat [R]
**AN5160.3**	**ANIA_05160**	**0.39**		Protein of unknown function	
**AN5168.3**	**ANIA_05168**	**0.50**		Has domain(s) with predicted nucleoside-triphosphatase activity, nucleotide binding activity	Ankyrin [M]
**AN5200.3**	**ANIA_05200**	**0.38**		Putative kynurenine 3-monooxygenase with a predicted role in aromatic amino acid biosynthesis	Kynurenine 3-monooxygenase and related flavoprotein monooxygenases [C, R]
**AN5207.3**	**ANIA_05207**	**0.36**		Ortholog(s) have cytosol, nucleus localization	
**AN5211.3**	**ANIA_05211**	**0.47**		Ortholog(s) have 1-phosphatidylinositol-3-phosphate 5-kinase activity, phosphatidylinositol-3-phosphate binding activity	Phosphatidylinositol-4-phosphate 5-kinase and related FYVE finger-containing proteins [T]
**AN5216.3**	**ANIA_05216**	**0.41**		Ortholog(s) have role in nucleotide-excision repair involved in interstrand cross-link repair and nucleus localization	5'-3' exonuclease [L]
**AN5222.3**	**ANIA_05222**	**0.40**		Ortholog(s) have structural constituent of ribosome activity and role in maturation of SSU-rRNA from tricistronic rRNA transcript (SSU-rRNA, 5.8S rRNA, LSU-rRNA), ribosomal small subunit assembly	40S ribosomal protein S11 [J]
**AN5258.3**	**ANIA_05258**	**0.48**		Ortholog(s) have role in fumiquinazoline C biosynthetic process, secondary metabolite biosynthetic process	
**AN5259.3**	**ANIA_05259**	**0.31**		Has domain(s) with predicted sequence-specific DNA binding RNA polymerase II transcription factor activity, zinc ion binding activity, role in regulation of transcription, DNA-dependent and nucleus localization	
**AN5263.3**	**ANIA_05263**	**0.47**		Protein of unknown function	Predicted transporter (major facilitator superfamily) [R]
**AN5268.3**	**ANIA_05268**	**0.45**		Has domain(s) with predicted nucleic acid binding, nucleotide binding activity	
**AN5273.3**	**ANIA_05273**	**0.28**	hlyA	Bifunctional enzyme with 3-methylglutaconyl-CoA hydratase and 3 hydroxy-3-methylglutaryl-CoA lyase activity; involved in conversion of leucine to acetyl-CoA and acetoacetate	Hydroxymethylglutaryl-CoA lyase [C, E]
**AN5274.3**	**ANIA_05274**	**0.45**		Ortholog(s) have sequence-specific DNA binding activity	
**AN5295.3**	**ANIA_05295**	**0.28**		Has domain(s) with predicted carbon-carbon lyase activity and role in cellular aromatic compound metabolic process	
**AN5297.3**	**ANIA_05297**	**0.44**		Has domain(s) with predicted catalytic activity and role in metabolic process	
**AN5300.3**	**ANIA_05300**	**0.29**		Putative aconitate hydratase with a predicted role in the TCA cycle	Aconitase/homoaconitase (aconitase superfamily) [C, E]
**AN5308.3**	**ANIA_05308**	**0.35**		Protein of unknown function	

AN5319.3	ANIA_05319	0.23		Ortholog of Aspergillus versicolor : Aspve1_0074620, Aspve1_0893571 and Aspergillus sydowii : Aspsy1_0094036, Aspsy1_0136394	
AN5321.3	ANIA_05321	0.48		Putative triacylglycerol lipase with a predicted role in glycerolipid metabolism	Acetylcholinesterase/Butyrylcholinesterase [T]
AN5323.3	ANIA_05323	0.17		Has domain(s) with predicted substrate-specific transmembrane transporter activity, role in transmembrane transport and integral to membrane localization	Predicted transporter (major facilitator superfamily) [R]
AN5325.3	ANIA_05325	0.29		Protein of unknown function	
AN5332.3	ANIA_05332	0.34		Ortholog(s) have intracellular localization	
AN5346.3	ANIA_05346	0.45		Ortholog(s) have role in establishment or maintenance of cell polarity and endoplasmic reticulum localization	Beta-1,4-mannosyltransferase [O]
AN5355.3	ANIA_05355	0.23		Has domain(s) with predicted nucleotide binding, oxidoreductase activity, transferase activity, transferring acyl groups other than amino-acyl groups, zinc ion binding activity and role in oxidation-reduction process	Alcohol dehydrogenase, class V [Q]
AN5357.3	ANIA_05357	0.19		Predicted glycosylphosphatidylinositol (GPI)-anchored protein	
AN5359.3	ANIA_05359	0.28		Has domain(s) with predicted trichodiene synthase activity and role in sesquiterpenoid biosynthetic process	
AN5360.3	ANIA_05360	0.31	CYP58C1	Putative cytochrome P450	Cytochrome P450 CYP2 subfamily [Q]
AN5362.3	ANIA_05362	0.44		Ortholog of Aspergillus niger ATCC 1015 : 41393-mRNA	
AN5363.3	ANIA_05363	0.47		Protein of unknown function	
AN5371.3	ANIA_05371	0.31		Ortholog of A. nidulans FGSC A4 : AN6727, AN0386, A. fumigatus Af293 : Afu1g01610, Afu4g01060, Afu6g03180, Afu7g05880 and A. niger CBS 513.88 : An04g08650, An07g02000	
AN5387.3	ANIA_05387	0.33		Ortholog of A. fumigatus Af293 : Afu6g00360, A. niger CBS 513.88 : An13g01480, An02g11060, An18g00050 and A. oryzae RIB40 : AO090023000455, AO090003001393, AO090001000640	
AN5391.3	ANIA_05391	0.40		Has domain(s) with predicted zinc ion binding activity	
AN5401.3	ANIA_05401	0.21		Ortholog of A. fumigatus Af293 : Afu6g13860, A. niger CBS 513.88 : An08g08500, A. oryzae RIB40 : AO090103000362, Aspergillus sydowii : Aspsy1_0046494 and Aspergillus terreus NIH2624 : ATET_03448	
AN5431.3	ANIA_05431	0.23		Has domain(s) with predicted DNA binding, sequence-specific DNA binding RNA polymerase II transcription factor activity, zinc ion binding activity and role in regulation of transcription, DNA-dependent, transcription, DNA-templated	
AN5448.3	ANIA_05448	0.38		Ortholog of A. fumigatus Af293 : Afu6g13460, A. niger CBS 513.88 : An08g08850, Neosartorya fischeri NRRL 181 : NFIA_059420, Aspergillus wentii : Aspwe1_0194392 and Aspergillus versicolor : Aspve1_0053254	
AN5461.3	ANIA_05461	0.41		Ortholog of A. fumigatus Af293 : Afu5g00130, A. niger CBS 513.88 : An01g00570, Aspergillus wentii : Aspwe1_0022994, Aspergillus versicolor : Aspve1_0039792 and Aspergillus clavatus NRRL 1 : ACLA_024300	
AN5467.3	ANIA_05467	0.29		Ortholog of Aspergillus terreus NIH2624 : ATET_07601	
AN5480.3	ANIA_05480	0.28		Putative RNA binding protein; menadione stress-induced protein	
AN5503.3	ANIA_05503	0.29		Protein of unknown function	
AN5517.3	ANIA_05517	0.30	fbxB	Putative F-box protein	Cdc4 and related F-box and WD-40 proteins [R]
AN5520.3	ANIA_05520	0.42		Has domain(s) with predicted role in ribosome biogenesis and ribonucleoprotein complex localization	60S ribosomal protein L7A [J]
AN5535.3	ANIA_05535	0.21		Ortholog(s) have U2-type spliceosomal complex localization	Uncharacterized conserved protein [S]
AN5547.3	ANIA_05547	0.48		Has domain(s) with predicted nucleotide binding, oxidoreductase activity and role in metabolic process	Reductases with broad range of substrate specificities [R]
AN5550.3	ANIA_05550	0.31		Has domain(s) with predicted UDP-N-acetylmuramate dehydrogenase activity, flavin adenine dinucleotide binding activity and role in oxidation-reduction process	Proteins containing the FAD binding domain [C]
AN5553.3	ANIA_05553	0.27	CYP5080D1	Putative cytochrome P450	Cytochrome P450 CYP4/CYP19/CYP26 subfamilies [Q, I]
AN5556.3	ANIA_05556	0.49		Has domain(s) with predicted nucleotide binding, oxidoreductase activity and role in metabolic process	Predicted dehydrogenase [Q]
AN5576.3	ANIA_05576	0.40		Ortholog of A. fumigatus Af293 : Afu4g11590, A. niger CBS 513.88 : An04g04860, A. oryzae RIB40 : AO090003001061, Aspergillus wentii : Aspwe1_0166635 and Aspergillus sydowii : Aspsy1_0149581	
AN5577.3	ANIA_05577	0.24	sodB	Manganese-superoxide dismutase	Manganese superoxide dismutase [P]
AN5600.3	ANIA_05600	0.44		Has domain(s) with predicted transferase activity, transferring alkyl or aryl (other than methyl) groups activity	Cis-prenyltransferase [I]
AN5610.3	ANIA_05610	0.13		Putative L-aminoadipate-semialdehyde dehydrogenase with a predicted role in lysine metabolism	Non-ribosomal peptide synthetase/alpha-aminoadipate reductase and related enzymes [Q]
AN5613.3	ANIA_05613	0.35	hxA	Xanthine dehydrogenase (purine hydroxylase I) involved in purine catabolism; requires a molybdenum cofactor for function; induction by uric acid requires the product of the uaY regulatory gene	Xanthine dehydrogenase [F]
AN5624.3	ANIA_05624	0.28		Ortholog(s) have cytosol, endoplasmic reticulum, mitotic spindle pole body, nucleus localization	Predicted Ca2+-dependent phospholipid-binding protein [R]
AN5630.3	ANIA_05630	0.47		Ortholog(s) have protein-lysine N-methyltransferase activity, role in peptidyl-lysine methylation and cytosol, nucleus localization	N-methyltransferase [R]
AN5634.3	ANIA_05634	0.48	acuD	Isocitrate lyase, required for utilization of acetate and fatty acids as carbon sources; transcriptional induction in response to acetate is mediated by FacB; transcriptional induction in response to long-chain fatty acids mediated by FarA	Isocitrate lyase [C]
AN5654.3	ANIA_05654	0.08		Protein of unknown function	
AN5657.3	ANIA_05657	0.38		Ortholog of A. fumigatus Af293 : Afu4g13590, A. niger CBS 513.88 : An01g09400, A. oryzae RIB40 : AO090009000210, Aspergillus wentii : Aspwe1_0047218 and Aspergillus sydowii : Aspsy1_0199958	
AN5671.3	ANIA_05671	0.50		Has domain(s) with predicted phospholipid binding activity	
AN5681.3	ANIA_05681	0.40		Ortholog(s) have ubiquitin-protein ligase activity, role in protein import into peroxisome matrix, protein ubiquitination and peroxisomal membrane localization	Predicted E3 ubiquitin ligase, integral peroxisomal membrane protein [O]
AN5703.3	ANIA_05703	0.48		Putative electron-transferring-flavoprotein dehydrogenase with a predicted role in energy metabolism	NADH:ubiquinone oxidoreductase, NDUFS8/23 kDa subunit [C]
AN5704.3	ANIA_05704	0.41		Putative Type II fatty acid synthase with a predicted role in mitochondrial fatty acid formation	Acyl carrier protein/NADH-ubiquinone oxidoreductase, NDUFAB1/SDAP subunit [C, I, Q]
AN5709.3	ANIA_05709	0.43		Ortholog of A. fumigatus Af293 : Afu1g06670, A. niger CBS 513.88 : An18g05730, A. oryzae RIB40 : AO090001000467, Aspergillus wentii : Aspwe1_0038290 and Aspergillus sydowii : Aspsy1_0056984	
AN5718.3	ANIA_05718	0.40		Putative fumarate hydratase	
AN5721.3	ANIA_05721	0.46		Ortholog(s) have cytosol localization	Uncharacterized conserved protein [S]
AN5738.3	ANIA_05738	0.35		Ortholog(s) have methionyl-tRNA formyltransferase activity, role in conversion of methionyl-tRNA to N-formyl-methionyl-tRNA and mitochondrion localization	Methionyl-tRNA formyltransferase [J]
AN5764.3	ANIA_05764	0.36		Transcript induced by light in in developmentally competent mycelia	
AN5778.3	ANIA_05778	0.42		Ortholog(s) have role in Arp2/3 complex-mediated actin nucleation, cellular response to biotic stimulus and cellular response to starvation, more	Actin-related protein Arp2/3 complex, subunit ARPC1/p41-ARC [Z]
AN5784.3	ANIA_05784	0.47		Ortholog(s) have role in proteasomal ubiquitin-independent protein catabolic process, proteasome-mediated ubiquitin-dependent protein catabolic process and cytosol, nucleus, proteasome core complex, beta-subunit complex localization	20S proteasome, regulatory subunit beta type PSMB1/PRE7 [O]
AN5797.3	ANIA_05797	0.36		Ortholog(s) have role in cortical actin cytoskeleton organization, establishment or maintenance of actin cytoskeleton polarity and cytosol, nucleus localization	Arp2/3 complex-interacting protein VIP1/Asp1, involved in regulation of actin Z [Z]

**AN5808.3**	**ANIA_05808**	**0.37**	clrA	Zn(II)2Cys6 transcription factor; predicted regulator of cellulose degradation; mutant deficient for growth on cellobiose and for xylanase and cellulase activity when pregrown on glucose and shifted to Avicel medium	
**AN5814.3**	**ANIA_05814**	**0.35**		Putative TapA inhibitor protein; component of the TOR signaling pathway; rapamycin-resistant mutant displays enhanced growth on threonine	Uncharacterized conserved protein [S]
**AN5816.3**	**ANIA_05816**	**0.26**		Ortholog(s) have role in mitochondrial protein processing, mitochondrial proton-transporting ATP synthase complex assembly and mitochondrial inner membrane, mitochondrial intermembrane space localization	Ku70-binding protein [L]
**AN5827.3**	**ANIA_05827**	**0.28**		Has domain(s) with predicted ATP binding, ATP-dependent helicase activity, nucleic acid binding activity	mRNA splicing factor ATP-dependent RNA helicase [A]
**AN5843.3**	**ANIA_05843**	**0.28**	pdkA	Putative pyruvate, water dikinase	
**AN5847.3**	**ANIA_05847**	**0.46**		Ortholog of A. fumigatus Af293 : Afu2g08000, A. niger CBS 513.88 : An05g00550, Neosartorya fischeri NRRL 181 : NFIA_083670 and Aspergillus wentii : Aspwe1_0175059, Aspwe1_0670697	
**AN5866.3**	**ANIA_05866**	**0.47**	ngn20	Putative aralkylamine N-acetyltransferase; ortholog of S. cerevisiae Paa1p; expression reduced after exposure to farnesol	Arylalkylamine N-acetyltransferase [R]
**AN5873.3**	**ANIA_05873**	**0.40**	gcpB	Gamma-tubulin complex protein 2	Gamma-tubulin complex, DGRIP84/SPC97 component [Z]
**AN5917.3**	**ANIA_05917**	**0.48**		Ortholog(s) have alpha-glucoside:hydrogen symporter activity, maltose:hydrogen symporter activity, trehalose transmembrane transporter activity and role in disaccharide catabolic process, maltose transport, trehalose transport	Predicted transporter (major facilitator superfamily) [R]
**AN5935.3**	**ANIA_05935**	**0.28**		Has domain(s) with predicted transmembrane transporter activity, role in transmembrane transport and integral to membrane localization	Inorganic phosphate transporter [P]
**AN5939.3**	**ANIA_05939**	**0.22**		Putative 5'-nucleotidase with a predicted role in nucleotide salvage pathways; predicted glycosylphosphatidylinositol (GPI)-anchored protein	5' nucleotidase [F]
**AN5941.3**	**ANIA_05941**	**0.39**		Protein of unknown function	
**AN5947.3**	**ANIA_05947**	**0.25**		Ortholog of A. niger CBS 513.88 : An11g05770, Neosartorya fischeri NRRL 181 : NFIA_007650, Aspergillus versicolor : Aspve1_0047552 and Aspergillus zonatus : Aspzo1_0020862	
**AN5952.3**	**ANIA_05952**	**0.29**		Putative kynureninase with a predicted role in aromatic amino acid biosynthesis	L-kynurenine hydrolase [E]
**AN5955.3**	**ANIA_05955**	**0.29**		Has domain(s) with predicted DNA binding, sequence-specific DNA binding RNA polymerase II transcription factor activity, zinc ion binding activity and role in regulation of transcription, DNA-dependent, transcription, DNA-templated	
**AN5956.3**	**ANIA_05956**	**0.14**		Has domain(s) with predicted DNA binding, transposase activity and role in DNA integration, transposition, DNA-mediated	
**AN5970.3**	**ANIA_05970**	**0.39**		Ortholog(s) have Golgi apparatus localization	Thioredoxin/protein disulfide isomerase [O]
**AN5971.3**	**ANIA_05971**	**0.34**		Putative NADH-ubiquinone oxidoreductase	
**AN5994.3**	**ANIA_05994**	**0.37**		Ortholog(s) have ATP-dependent NAD(P)H-hydrate dehydratase activity, role in nicotinamide nucleotide metabolic process and cytosol localization	Predicted sugar kinase [G]
**AN6005.3**	**ANIA_06005**	**0.45**		Ortholog of A. fumigatus Af293 : Afu2g10020, A. niger CBS 513.88 : An16g05030, Neosartorya fischeri NRRL 181 : NFIA_085440, Aspergillus wentii : Aspwe1_0176252 and Aspergillus versicolor : Aspve1_0040814	
**AN6036.3**	**ANIA_06036**	**0.46**		Ortholog(s) have U1 snRNP, cytosol localization	U1 snRNP complex, subunit SNU71 and related PWI-motif proteins [A]
**AN6053.3**	**ANIA_06053**	**0.46**		Ortholog(s) have role in cell volume homeostasis, cellular calcium ion homeostasis, regulation of resting membrane potential and cortical endoplasmic reticulum localization	Predicted mechanosensitive ion channel [M]
**AN6066.3**	**ANIA_06066**	**0.48**		Has domain(s) with predicted catalytic activity and role in metabolic process	Mitochondrial associated endoribonuclease MAR1 (isochorismatase superfamily) [R]
**AN6077.3**	**ANIA_06077**	**0.38**		Putative NADH dehydrogenase (ubiquinone) with a predicted role in energy metabolism	NADH:ubiquinone oxidoreductase, NDUFV2/24 kD subunit [C]
**AN6105.3**	**ANIA_06105**	**0.49**		Has domain(s) with predicted carbon-sulfur lyase activity and role in metabolic process	
**AN6111.3**	**ANIA_06111**	**0.47**		Ortholog(s) have cytosol, exosome (RNase complex), nucleus localization	Exosomal 3'-5' exoribonuclease complex, subunit Rrp45 [J]
**AN6115.3**	**ANIA_06115**	**0.50**		Ortholog(s) have high-affinity nickel cation transmembrane transporter activity, role in nickel cation transmembrane transport, regulation of urea catabolic process and endoplasmic reticulum, plasma membrane localization	
**AN6129.3**	**ANIA_06129**	**0.41**		Ortholog(s) have role in cellular response to drug, filamentous growth of a population of unicellular organisms and nitrogen utilization, more	
**AN6138.3**	**ANIA_06138**	**0.49**	bimA	Component of the Anaphase-Promoting Complex/Cyclosome (APC/C), which is a ubiquitin ligase required for cell cycle progression; mutations lead to DNA damage and block cytokinesis; tetratricopeptide repeat (TPR) gene family member	DNA-binding cell division cycle control protein [D]
**AN6172.3**	**ANIA_06172**	**0.48**		Ortholog of A. fumigatus Af293 : Afu2g08340, A. niger CBS 513.88 : An05g00970, A. oryzae RIB40 : AO090011000871, Aspergillus wentii : Aspwe1_0176098 and Aspergillus sydowii : Aspsy1_0151670	
**AN6178.3**	**ANIA_06178**	**0.34**		Has domain(s) with predicted role in regulation of transcription, DNA-dependent and integral to membrane, nucleus localization	
**AN6183.3**	**ANIA_06183**	**0.34**		Putative F-box protein	
**AN6188.3**	**ANIA_06188**	**0.37**		Ortholog(s) have cytosol, nucleus localization	Predicted hydrolases of HD superfamily [R]
**AN6190.3**	**ANIA_06190**	**0.40**		Ortholog(s) have role in sporocarp development involved in sexual reproduction, syncytium formation by plasma membrane fusion	Cell cycle-associated protein [D]
**AN6201.3**	**ANIA_06201**	**0.40**		Ortholog of A. fumigatus Af293 : Afu2g11830, A. oryzae RIB40 : AO090026000461, Neosartorya fischeri NRRL 181 : NFIA_087060, Aspergillus wentii : Aspwe1_0175601 and Aspergillus versicolor : Aspve1_0052426	
**AN6202.3**	**ANIA_06202**	**0.48**	rpl3	Putative ribosomal protein L3	60S ribosomal protein L3 and related proteins [J]
**AN6207.3**	**ANIA_06207**	**0.45**	pkpC	Putative protein kinase; reduced growth on AVICEL medium	Dehydrogenase kinase [T]
**AN6210.3**	**ANIA_06210**	**0.50**		Ortholog(s) have GTP-Rho binding, phosphatidylinositol-4,5-bisphosphate binding activity and role in Golgi to plasma membrane transport, Rho protein signal transduction, exocyst assembly, exocyst localization	Exocyst component protein and related proteins [U]
**AN6246.3**	**ANIA_06246**	**0.43**	cycA	Cytochrome C	
**AN6251.3**	**ANIA_06251**	**0.33**	ndxD	Predicted hydrolase	Diadenosine and diphosphoinositol polyphosphate phosphohydrolase [T]
**AN6253.3**	**ANIA_06253**	**0.46**	podG	Putative phenylalanine tRNA synthetase alpha subunit	Phenylalanyl-tRNA synthetase [J]
**AN6255.3**	**ANIA_06255**	**0.39**		Putative cytochrome c oxidase subunit with a predicted role in energy metabolism	Cytochrome c oxidase, subunit VIb/COX12 [C]
**AN6261.3**	**ANIA_06261**	**0.41**		Ortholog(s) have role in double-strand break repair, replication fork processing and nucleus localization	
**AN6266.3**	**ANIA_06266**	**0.43**		Ortholog(s) have nucleus localization	Uncharacterized conserved protein [S]
**AN6274.3**	**ANIA_06274**	**0.21**		Has domain(s) with predicted nucleotide binding, oxidoreductase activity and role in metabolic process	Reductases with broad range of substrate specificities [R]
**AN6310.3**	**ANIA_06310**	**0.35**		Ortholog(s) have role in rRNA processing, ribosomal large subunit assembly and mitochondrion, mitotic spindle pole body, nucleoplasm localization	AAA ATPase containing von Willebrand factor type A (vWA) domain [R]
**AN6311.3**	**ANIA_06311**	**0.43**		Has domain(s) with predicted voltage-gated chloride channel activity, role in chloride transport, transmembrane transport and membrane localization	Cl- channel CLC-3 and related proteins (CLC superfamily) [P]

**AN6319.3**	**ANIA_06319**	**0.45**		Ortholog of A. nidulans FGSC A4 : AN0638, A. fumigatus Af293 : Afu1g16870, A. oryzae RIB40 : AO090005000988, Aspergillus wentii : Aspwe1_0038696 and Aspergillus sydowii : Aspsy1_0129541, Aspsy1_0995740	
**AN6321.3**	**ANIA_06321**	**0.46**	CYP656A1	Putative cytochrome P450	Cytochrome P450 CYP4/CYP19/CYP26 subfamilies [Q, I]
**AN6335.3**	**ANIA_06335**	**0.31**		Ortholog(s) have role in meiotic chromosome segregation	
**AN6349.3**	**ANIA_06349**	**0.26**		Ortholog of A. fumigatus Af293 : Afu2g14180, A. niger CBS 513.88 : An02g01370, A. oryzae RIB40 : AO090023000134, Aspergillus wentii : Aspwe1_0697288 and Aspergillus sydowii : Aspsy1_0178307	Cell cycle-associated protein [D]
**AN6360.3**	**ANIA_06360**	**0.34**		Has domain(s) with predicted role in autophagy	
**AN6381.3**	**ANIA_06381**	**0.40**		Has domain(s) with predicted role in transmembrane transport and integral to membrane localization	Permease of the major facilitator superfamily [G]
**AN6390.3**	**ANIA_06390**	**0.48**		Has domain(s) with predicted homogentisate 1,2-dioxygenase activity and role in L-phenylalanine catabolic process, oxidation-reduction process, tyrosine metabolic process	Homogentisate 1,2-dioxygenase [E]
**AN6392.3**	**ANIA_06392**	**0.28**		Has domain(s) with predicted sequence-specific DNA binding RNA polymerase II transcription factor activity, zinc ion binding activity, role in regulation of transcription, DNA-dependent and nucleus localization	
**AN6393.3**	**ANIA_06393**	**0.40**		Has domain(s) with predicted acetyltransferase activity	Serine O-acetyltransferase [E]
**AN6397.3**	**ANIA_06397**	**0.37**		Has domain(s) with predicted ATP binding, nucleoside-triphosphatase activity	AAA+-type ATPase [O]
**AN6398.3**	**ANIA_06398**	**0.28**		Ortholog(s) have intracellular localization	Flavonol reductase/cinnamoyl-CoA reductase [V]
**AN6410.3**	**ANIA_06410**	**0.29**		Protein of unknown function; transcript is induced by nitrate	Dosage compensation complex, subunit MLE [K]
**AN6439.3**	**ANIA_06439**	**0.49**		Ortholog(s) have intracellular localization	Zinc-binding oxidoreductase [C, R]
**AN6458.3**	**ANIA_06458**	**0.36**		Ortholog of A. fumigatus Af293 : Afu3g07420, A. niger CBS 513.88 : An02g10960, An01g01420, An12g05390, A. oryzae RIB40 : AO090020000161, AO090005000921, AO090003001427 and Aspergillus sydowii : Aspsy1_0086152	
**AN6461.3**	**ANIA_06461**	**0.40**		Has domain(s) with predicted role in transmembrane transport and integral to membrane localization	Permease of the major facilitator superfamily [G]
**AN6469.3**	**ANIA_06469**	**0.15**		Protein of unknown function	
**AN6485.3**	**ANIA_06485**	**0.36**	CYP678A1	Putative cytochrome P450	Cytochrome P450 CYP3/CYP5/CYP6/CYP9 subfamilies [Q]
**AN6512.3**	**ANIA_06512**	**0.36**		Ortholog(s) have cytosol, nucleus localization	Predicted ABC-type transport, ATPase component/CCR4 associated factor [R, K]
**AN6523.3**	**ANIA_06523**	**0.29**	sepA	Formin; FH1/2 domain protein; colocalizes with TeaA at cell apex, localization is actin-dependent; required for the formation of actin rings at sites of septation; mutant spores exhibit a random pattern of germ tube emergence	RhoA GTPase effector DIA/Diaphanous [T, Z]
**AN6525.3**	**ANIA_06525**	**0.43**	aciA	Putative formate dehydrogenase with a predicted role in oxalic acid metabolism; intracellular; protein abundance decreased by menadione stress; inducible by acetate; expression reduced after exposure to farnesol	Glyoxylate/hydroxypyruvate reductase (D-isomer-specific 2-hydroxy acid dehydrogenase superfamily) [C]
**AN6543.3**	**ANIA_06543**	**0.45**		Ortholog(s) have adenyl-nucleotide exchange factor activity, role in cytoplasm-associated proteasomal ubiquitin-dependent protein catabolic process, cytoplasmic translation and cytosolic ribosome, nucleus localization	Armadillo/beta-catenin-like repeat-containing protein [O]
**AN6547.3**	**ANIA_06547**	**0.48**		Ortholog(s) have role in proteasomal ubiquitin-independent protein catabolic process, proteasome-mediated ubiquitin-dependent protein catabolic process	20S proteasome, regulatory subunit alpha type PSMA1/PRE5 [O]
**AN6550.3**	**ANIA_06550**	**0.33**	pabaA	Para-aminobenzoic acid synthase, enzyme of folic acid biosynthesis	Para-aminobenzoate (PABA) synthase ABZ1 [J]
**AN6557.3**	**ANIA_06557**	**0.39**		Ortholog(s) have mitochondrial respiratory chain complex I, peripheral segment localization	NADH:ubiquinone oxidoreductase, NDUFA6/B14 subunit [C]
**AN6569.3**	**ANIA_06569**	**0.39**		Ortholog(s) have chromatin DNA binding activity and role in positive regulation of transcription from RNA polymerase II promoter, telomere maintenance, threonylcarbamoyladenosine metabolic process	Predicted metalloprotease with chaperone activity (RNAse H/HSP70 fold) [O]
**AN6571.3**	**ANIA_06571**	**0.41**		Ortholog(s) have alpha-1,2-mannosyltransferase activity, role in mannan biosynthetic process, protein mannosylation and Golgi apparatus localization	
**AN6574.3**	**ANIA_06574**	**0.28**		Ortholog(s) have ubiquitin-protein ligase activity	Myosin class II heavy chain [Z]
**AN6577.3**	**ANIA_06577**	**0.27**		Ortholog(s) have role in mitochondrial tRNA wobble uridine modification and mitochondrion localization	NAD/FAD-utilizing protein possibly involved in translation [J]
**AN6580.3**	**ANIA_06580**	**0.32**		Putative cardiolipin synthase with a predicted role in phospholipid metabolism	CDP-alcohol phosphatidyltransferase/Phosphatidylglycerol-phosphate synthase [I]
**AN6589.3**	**ANIA_06589**	**0.41**		Ortholog(s) have mannose-ethanolamine phosphotransferase activity	Glycosylphosphatidylinositol anchor synthesis protein [T]
**AN6590.3**	**ANIA_06590**	**0.28**		Ortholog(s) have role in cellular protein localization, cellular response to nitrogen starvation, negative regulation of protein binding and positive regulation of GTPase activity, more	Tuberin - Rap/ran-GTPase-activating protein [D, T]
**AN6593.3**	**ANIA_06593**	**0.41**		Ortholog(s) have unfolded protein binding activity, role in aerobic respiration, mitochondrial respiratory chain complex IV assembly and integral to mitochondrial inner membrane, plasma membrane localization	
**AN6594.3**	**ANIA_06594**	**0.48**		Ortholog(s) have role in ascospore formation, regulation of mitotic cell cycle, regulation of translation and cytosol, nucleus localization	Uncharacterized conserved protein [S]
**AN6602.3**	**ANIA_06602**	**0.47**		Has domain(s) with predicted nucleotide binding, oxidoreductase activity and role in metabolic process	Predicted dehydrogenase [Q]
**AN6604.3**	**ANIA_06604**	**0.27**		Ortholog(s) have ATPase activity, tRNA binding activity, role in tRNA modification and Elongator holoenzyme complex, cytosol, nucleus localization	
**AN6609.3**	**ANIA_06609**	**0.26**		Ortholog(s) have cytosol, nucleus localization	Predicted E3 ubiquitin ligase [O]
**AN6618.3**	**ANIA_06618**	**0.40**		Ortholog(s) have cytosol, nucleus localization	Ypt/Rab-specific GTPase-activating protein GYP7 and related proteins [T]
**AN6640.3**	**ANIA_06640**	**0.27**		Has domain(s) with predicted hydrolase activity and role in metabolic process	
**AN6642.3**	**ANIA_06642**	**0.16**	enaA	Putative potassium-transporting ATPase with a predicted role in energy metabolism; locus contains the conserved upstream open reading frames (uORFs) AN6642-uORF and AN6642-uORF.2	Ca2+ transporting ATPase [P]
**AN6647.3**	**ANIA_06647**	**0.50**		Has domain(s) with predicted oxidoreductase activity and role in oxidation-reduction process	FAD-dependent oxidoreductase [R]
**AN6654.3**	**ANIA_06654**	**0.32**		Has domain(s) with predicted glutamate-ammonia ligase activity and role in glutamine biosynthetic process	
**AN6695.3**	**ANIA_06695**	**0.44**	nmdA	Protein involved in pre-mRNA stability and nonsense-mediated mRNA decay (NMD) pathway	Nonsense-mediated mRNA decay 2 protein [A]
**AN6704.3**	**ANIA_06704**	**0.27**		Protein of unknown function	
**AN6737.3**	**ANIA_06737**	**0.43**		Ortholog(s) have mitochondrion localization	
**AN6750.3**	**ANIA_06750**	**0.48**		Has domain(s) with predicted oxidoreductase activity and role in metabolic process	Kynurenine 3-monooxygenase and related flavoprotein monooxygenases [C, R]
**AN6792.3**	**ANIA_06792**	**0.40**	gfdB	Putative NAD+ dependent glycerol 3-phosphate dehydrogenase with a predicted role in glycerol metabolism	Glycerol-3-phosphate dehydrogenase/dihydroxyacetone 3-phosphate reductase [C]
**AN6834.3**	**ANIA_06834**	**0.36**		Has domain(s) with predicted substrate-specific transmembrane transporter activity, role in transmembrane transport and integral to membrane localization	Predicted transporter (major facilitator superfamily) [R]
**AN6856.3**	**ANIA_06856**	**0.19**		Ortholog of A. fumigatus Af293 : Afu5g13100, A. niger CBS 513.88 : An14g06050, A. oryzae RIB40 : AO090120000435, Aspergillus wentii : Aspwe1_0107169 and Aspergillus sydowii : Aspsy1_0040420	
**AN6857.3**	**ANIA_06857**	**0.23**		Ortholog(s) have alpha-1,2-mannosyltransferase activity, role in protein glycosylation and Golgi apparatus localization	
**AN6862.3**	**ANIA_06862**	**0.12**		Ortholog of A. niger CBS 513.88 : An03g01640, Neosartorya fischeri NRRL 181 : NFIA_074040, Aspergillus wentii : Aspwe1_0170839 and Aspergillus versicolor : Aspve1_0035273	
**AN6892.3**	**ANIA_06892**	**0.22**	ptcA	Putative protein serine/threonine phosphatase; reduced growth on AVICEL medium and reduced eglA and eglB expression	Serine/threonine protein phosphatase [T]

**AN6924.3**	**ANIA_06924**	**0.43**		Ortholog of A. nidulans FGSC A4 : AN5943, AN8512, AN8548, AN0857, AN8661, AN4642 and A. fumigatus Af293 : Afu1g15290, Afu3g00850, Afu4g08850, Afu7g00920	
**AN6930.3**	**ANIA_06930**	**0.30**		Has domain(s) with predicted pyridoxal phosphate binding, transaminase activity	Alanine-glyoxylate aminotransferase AGT2 [E]
**AN6933.3**	**ANIA_06933**	**0.29**	maeB	Putative malate dehydrogenase with a predicted role in oxidation of malate to pyruvate; transcript is induced by nitrate	NADP+-dependent malic enzyme [C]
**AN6943.3**	**ANIA_06943**	**0.48**		Putative glycerate kinase with a predicted role in glycerate (three-carbon) metabolism	
**AN6962.3**	**ANIA_06962**	**0.25**		Ortholog of the non-ribosomal peptide synthetase (NRPS) of A. fumigatus, nrps14/Afu8g00540; predicted secondary metabolism gene cluster member; coregulated with AN6961	
**AN6963.3**	**ANIA_06963**	**0.22**		Ortholog(s) have flavin-linked sulfhydryl oxidase activity and role in oxidation-reduction process	
**AN6964.3**	**ANIA_06964**	**0.31**		Ortholog of Aspergillus glaucus : Aspgl1_0086825 and Aspergillus versicolor : Aspve1_0093575	
**AN6968.3**	**ANIA_06968**	**0.50**		Has domain(s) with predicted RNA binding, RNA-directed DNA polymerase activity and role in RNA-dependent DNA replication	
**AN6992.3**	**ANIA_06992**	**0.49**		Ortholog of Neosartorya fischeri NRRL 181 : NFIA_028800, Aspergillus wentii : Aspwe1_0043211, Aspergillus versicolor : Aspve1_0154813 and Aspergillus clavatus NRRL 1 : ACLA_053800	Dolichol-phosphate mannosyltransferase, subunit 3 [O, T]
**AN6993.3**	**ANIA_06993**	**0.37**		Putative phosphoribosyltransferase with a predicted role in nucleotide salvage pathways	
**AN7020.3**	**ANIA_07020**	**0.23**		Protein of unknown function	
**AN7025.3**	**ANIA_07025**	**0.48**		Ortholog of A. fumigatus Af293 : Afu4g04220, A. oryzae RIB40 : AO090206000072, Aspergillus wentii : Aspwe1_0114911, Aspergillus sydowii : Aspsy1_0060635 and Aspergillus terreus NIH2624 : ATET_10114	
**AN7038.3**	**ANIA_07038**	**0.33**		Ortholog of A. fumigatus Af293 : Afu4g04090, A. niger CBS 513.88 : An14g00760, A. oryzae RIB40 : AO090206000113, Aspergillus wentii : Aspwe1_0613920 and Aspergillus sydowii : Aspsy1_0158081	
**AN7053.3**	**ANIA_07053**	**0.50**		Ortholog of Aspergillus versicolor : Aspve1_0054863	Dosage compensation complex, subunit MLE [K]
**AN7076.3**	**ANIA_07076**	**0.41**		Protein of unknown function	
**AN7084.3**	**ANIA_07084**	**0.48**		Putative polyketide synthase (PKS)-like enzyme; predicted secondary metabolism gene cluster member	Animal-type fatty acid synthase and related proteins [I]
**AN7088.3**	**ANIA_07088**	**0.29**		Has domain(s) with predicted transmembrane transporter activity, role in transmembrane transport and integral to membrane localization	Predicted transporter (major facilitator superfamily) [R]
**AN7102.3**	**ANIA_07102**	**0.05**		Ortholog of A. fumigatus Af293 : Afu4g03830, A. niger CBS 513.88 : An14g01068, A. oryzae RIB40 : AO090011000343, Aspergillus wentii : Aspwe1_0030332 and Aspergillus sydowii : Aspsy1_0060599	
**AN7106.3**	**ANIA_07106**	**0.43**		Ortholog(s) have role in meiotic telomere clustering and negative regulation of transcription from RNA polymerase II promoter by glucose, more	RNA polymerase II K factor complex subunit [K]
**AN7115.3**	**ANIA_07115**	**0.42**		Has domain(s) with predicted role in guanosine tetraphosphate metabolic process	
**AN7135.3**	**ANIA_07135**	**0.28**	rglA	Protein with rhamnogalacturonan lyase activity, involved in degradation of pectin	
**AN7144.3**	**ANIA_07144**	**0.50**		Ortholog(s) have protein binding involved in protein folding activity, role in mitochondrial respiratory chain complex III assembly and mitochondrial matrix localization	
**AN7185.3**	**ANIA_07185**	**0.46**	srpkA	Putative protein serine/threonine kinase; mutants are sensitive to NaCl and have a moderate growth defect	Serine/threonine protein kinase [T]
**AN7240.3**	**ANIA_07240**	**0.36**		Has domain(s) with predicted role in transmembrane transport and integral to membrane localization	
**AN7255.3**	**ANIA_07255**	**0.31**		Ortholog of A. oryzae RIB40 : AO090102000108, Aspergillus wentii : Aspwe1_0171112, Aspergillus versicolor : Aspve1_0038807 and Aspergillus niger ATCC 1015 : 194648-mRNA	
**AN7273.3**	**ANIA_07273**	**0.36**		Ortholog of A. fumigatus Af293 : Afu2g16990, A. oryzae RIB40 : AO090102000119, Aspergillus wentii : Aspwe1_0182487, Aspergillus sydowii : Aspsy1_0027170 and Aspergillus terreus NIH2624 : ATET_10043	
**AN7274.3**	**ANIA_07274**	**0.41**		Ortholog(s) have role in fumiquinazoline C biosynthetic process, secondary metabolite biosynthetic process	Proteins containing the FAD binding domain [C]
**AN7278.3**	**ANIA_07278**	**0.06**		Putative glutamate decarboxylase with a predicted role in the 4-aminobutyrate (GABA) shunt	Glutamate decarboxylase/sphingosine phosphate lyase [E]
**AN7279.3**	**ANIA_07279**	**0.32**		Has domain(s) with predicted poly(beta-D-mannuronate) lyase activity, role in alginic acid catabolic process and periplasmic space localization	
**AN7314.3**	**ANIA_07314**	**0.50**		Ortholog(s) have phosphatidylinositol-3,5-bisphosphate 5-phosphatase activity, role in phosphatidylinositol dephosphorylation and PAS complex, cytosol, extrinsic to membrane, fungal-type vacuole membrane localization	Putative phosphoinositide phosphatase [I]
**AN7315.3**	**ANIA_07315**	**0.35**		Putative succinate-semialdehyde dehydrogenase [NAD(P)+] with a predicted role in the 4-aminobutyrate (GABA) shunt	Aldehyde dehydrogenase [C]
**AN7321.3**	**ANIA_07321**	**0.48**	hriA	Ortholog(s) have eukaryotic translation initiation factor 2alpha kinase activity, role in negative regulation of translational initiation in response to osmotic stress, protein phosphorylation and cytosol localization	eIF-2alpha kinase GCN2 [J]
**AN7328.3**	**ANIA_07328**	**0.45**		Ortholog of A. fumigatus Af293 : Afu2g16550, A. niger CBS 513.88 : An03g03580, A. oryzae RIB40 : AO090102000183, Aspergillus wentii : Aspwe1_0039308 and Aspergillus sydowii : Aspsy1_0140287	
**AN7331.3**	**ANIA_07331**	**0.18**		Has domain(s) with predicted DNA binding, cyanate hydratase activity and role in cyanate metabolic process	
**AN7345.3**	**ANIA_07345**	**0.40**	agdC	Protein with glucosidase activity, involved in degradation of glucans; predicted role in maltose metabolism	Maltase glucoamylase and related hydrolases, glycosyl hydrolase family 31 [G]
**AN7353.3**	**ANIA_07353**	**0.43**		Putative galactose 1-dehydrogenase with a predicted role in galactonic acid and galactonate metabolism	Predicted oxidoreductase [R]
**AN7376.3**	**ANIA_07376**	**0.43**		Has domain(s) with predicted sequence-specific DNA binding RNA polymerase II transcription factor activity, zinc ion binding activity, role in regulation of transcription, DNA-dependent and nucleus localization	
**AN7387.3**	**ANIA_07387**	**0.32**	pcrA	Putative pyrroline-5-carboxylate reductase with a predicted role in proline metabolism	Pyrroline-5-carboxylate reductase [E]
**AN7395.3**	**ANIA_07395**	**0.35**		Ortholog of A. nidulans FGSC A4 : AN5639, AN2587, AN9444, A. fumigatus Af293 : Afu5g00840, A. niger CBS 513.88 : An03g01000 and A. oryzae RIB40 : AO090102000018	
**AN7425.3**	**ANIA_07425**	**0.43**		Has domain(s) with predicted lipid transporter activity, role in lipid transport and integral to membrane localization	Nuclear division RFT1 protein [D]
**AN7437.3**	**ANIA_07437**	**0.48**		Ortholog(s) have role in amino acid transmembrane export from vacuole, cellular response to nitrogen starvation and integral to fungal-type vacuolar membrane localization	
**AN7456.3**	**ANIA_07456**	**0.44**		Ortholog(s) have U2 snRNP localization	Predicted RNA-binding protein (RRM superfamily) [R]
**AN7460.3**	**ANIA_07460**	**0.34**		Ortholog of Aspergillus versicolor : Aspve1_0030998 and Aspergillus sydowii : Aspsy1_0203499	
**AN7471.3**	**ANIA_07471**	**0.28**		Ortholog(s) have cytoplasm localization	Predicted carbohydrate kinase, contains PfkB domain [R]
**AN7497.3**	**ANIA_07497**	**0.42**		Ortholog(s) have oxidoreductase activity and mitochondrion, plasma membrane localization	NADH:ubiquinone oxidoreductase, NDUFS4/18 kDa subunit [C]
**AN7498.3**	**ANIA_07498**	**0.40**		Ortholog(s) have deoxyhypusine monooxygenase activity	HEAT repeat-containing protein [R]
**AN7502.3**	**ANIA_07502**	**0.29**		Putative uridine kinase with a predicted role in pyrimidine metabolism; expression reduced after exposure to farnesol	Armadillo/beta-Catenin/plakoglobin [T, Z]
**AN7560.3**	**ANIA_07560**	**0.42**	palC	Protein involved in a signaling pathway that activates PacC transcription factor in response to alkaline ambient pH	

**AN7590.3**	**ANIA_07590**	**0.46**		Putative reductase with a predicted role in carbohydrate metabolism; mannitol 2-dehydrogenase; intracellular, menadione stress-induced protein; HapX-regulated; protein induced by farnesol	Reductases with broad range of substrate specificities [R]
**AN7591.3**	**ANIA_07591**	**0.36**		Ortholog of A. nidulans FGSC A4 : AN7437, A. fumigatus Af293 : Afu2g06170, Afu2g15370, A. niger CBS 513.88 : An09g03630, An02g14810/atg22 and A. oryzae RIB40 : AO090012000289, AO090001000734	
**AN7595.3**	**ANIA_07595**	**0.28**		Ortholog of A. fumigatus Af293 : Afu2g15270, A. niger CBS 513.88 : An09g03530, Neosartorya fischeri NRRL 181 : NFIA_090510 and Aspergillus versicolor : Aspve1_0030808	
**AN7607.3**	**ANIA_07607**	**0.40**		Ortholog of A. fumigatus Af293 : Afu2g15480, A. niger CBS 513.88 : An15g05550, An12g07620, A. oryzae RIB40 : AO090012000281, AO090020000707 and Aspergillus wentii : Aspwe1_0025841, Aspwe1_0037842, Aspwe1_0044672	
**AN7609.3**	**ANIA_07609**	**0.45**		Ortholog(s) have mitochondrial RNA polymerase regulatory region sequence-specific DNA binding activity and cytosol, mitochondrion localization	Mitochondrial/chloroplast DNA-directed RNA polymerase RPO41, provides primers for DNA replication-initiation [K, L]
**AN7614.3**	**ANIA_07614**	**0.29**		Protein of unknown function	
**AN7629.3**	**ANIA_07629**	**0.36**		Has domain(s) with predicted NADH dehydrogenase (ubiquinone) activity and mitochondrion localization	
**AN7633.3**	**ANIA_07633**	**0.36**		Putative malate dehydrogenase	
**AN7636.3**	**ANIA_07636**	**0.32**		Has domain(s) with predicted sulfuric ester hydrolase activity and role in metabolic process	Zinc-binding oxidoreductase [C, R]
**AN7645.3**	**ANIA_07645**	**0.35**		Protein of unknown function	
**AN7683.3**	**ANIA_07683**	**0.40**		Protein associated with the nonclassical protein export pathway; ortholog of S. cerevisiae Nce102p	
**AN7691.3**	**ANIA_07691**	**0.48**	plcB	Putative phospholipase	
**AN7771.3**	**ANIA_07771**	**0.50**		Ortholog of A. fumigatus Af293 : Afu5g07480, A. niger CBS 513.88 : An15g02850, A. oryzae RIB40 : AO090701000657, Aspergillus wentii : Aspwe1_0035055 and Aspergillus sydowii : Aspsy1_0046323	
**AN7777.3**	**ANIA_07777**	**0.28**		Ortholog of A. fumigatus Af293 : Afu2g00310, A. niger CBS 513.88 : An09g03250, A. oryzae RIB40 : AO090701000622, Aspergillus sydowii : Aspsy1_0153492 and Aspergillus terreus NIH2624 : ATET_08410	Amino acid transporters [E]
**AN7782.3**	**ANIA_07782**	**0.26**		Has domain(s) with predicted oxidoreductase activity, acting on paired donors, with incorporation or reduction of molecular oxygen, 2-oxoglutarate as one donor, and incorporation of one atom each of oxygen into both donors activity	
**AN7796.3**	**ANIA_07796**	**0.22**		Has domain(s) with predicted role in transmembrane transport and integral to membrane localization	Synaptic vesicle transporter SVOP and related transporters (major facilitator superfamily) [R]
**AN7798.3**	**ANIA_07798**	**0.15**		Ortholog of A. nidulans FGSC A4 : AN6764, AN2660, A. fumigatus Af293 : Afu8g01960 and A. niger CBS 513.88 : An15g04120, An11g05430, An01g12050, An11g03480, An01g01140	
**AN7799.3**	**ANIA_07799**	**0.35**		Protein expressed at increased levels during osmoadaptation; short-chain alcohol dehydrogenase domain	Dehydrogenases with different specificities (related to short-chain alcohol dehydrogenases) [Q]
**AN7850.3**	**ANIA_07850**	**0.31**		Ortholog of A. fumigatus Af293 : Afu4g14030, A. oryzae RIB40 : AO090001000391, Neosartorya fischeri NRRL 181 : NFIA_102290, Aspergillus wentii : Aspwe1_0046564 and Aspergillus terreus NIH2624 : ATET_02187	
**AN7868.3**	**ANIA_07868**	**0.23**		Ortholog of A. nidulans FGSC A4 : AN1866, A. fumigatus Af293 : Afu2g04540, A. niger CBS 513.88 : An04g07800, A. oryzae RIB40 : AO090003000256 and Neosartorya fischeri NRRL 181 : NFIA_057620, NFIA_081290	
**AN7890.3**	**ANIA_07890**	**0.42**		Putative glucose oxidase with a predicted role in gluconic acid and gluconate metabolism	Glucose dehydrogenase/choline dehydrogenase/mandelonitrile lyase (GMC oxidoreductase family) [R]
**AN7904.3**	**ANIA_07904**	**0.14**		Has domain(s) with predicted methyltransferase activity and role in metabolic process	Predicted protein carboxyl methylase [R]
**AN7907.3**	**ANIA_07907**	**0.13**		Putative glyoxylate-bleomycin resistance protein; member of the F9775 secondary metabolite gene cluster	
**AN7928.3**	**ANIA_07928**	**0.34**		Possible pseudogene	
**AN7940.3**	**ANIA_07940**	**0.40**		Has domain(s) with predicted 2-methylcitrate dehydratase activity and role in propionate catabolic process	
**AN7959.3**	**ANIA_07959**	**0.27**		Ortholog of A. fumigatus Af293 : Afu5g12830, A. oryzae RIB40 : AO090120000409, Neosartorya fischeri NRRL 181 : NFIA_074340, Aspergillus wentii : Aspwe1_0449236 and Aspergillus versicolor : Aspve1_0175438	
**AN7988.3**	**ANIA_07988**	**0.19**		Protein of unknown function; transcript is induced by nitrate	Uncharacterized conserved protein [S]
**AN7989.3**	**ANIA_07989**	**0.14**		Putative phosphatidylserine decarboxylase with a predicted role in phospholipid metabolism	Phosphatidylserine decarboxylase [I]
**AN8018.3**	**ANIA_08018**	**0.23**		Transcript induced by light in in developmentally competent mycelia	Predicted membrane protein [S]
**AN8032.3**	**ANIA_08032**	**0.39**		Putative C-3 sterol dehydrogenase; expression upregulated after exposure to farnesol	C-3 sterol dehydrogenase/3-beta-hydroxysteroid dehydrogenase and related dehydrogenases [I, E]
**AN8036.3**	**ANIA_08036**	**0.37**		Ortholog of A. fumigatus Af293 : Afu5g01910, A. niger CBS 513.88 : An16g01770, A. oryzae RIB40 : AO090003001328, Aspergillus wentii : Aspwe1_0042781 and Aspergillus sydowii : Aspsy1_0047861	Aldo/keto reductase family proteins [R]
**AN8037.3**	**ANIA_08037**	**0.14**		Protein of unknown function	
**AN8050.3**	**ANIA_08050**	**0.31**		Ortholog(s) have role in meiotic chromosome segregation and cytosol, nucleus localization	Uncharacterized conserved protein [S]
**AN8054.3**	**ANIA_08054**	**0.39**		Predicted alpha 4 subunit of the 20S core proteasome; involved in N-myristoylation and cell morphogenesis; menadione stress-induced protein	20S proteasome, regulatory subunit alpha type PSMA7/PRE6 [O]
**AN8059.3**	**ANIA_08059**	**0.40**		Ortholog(s) have mitochondrion targeting sequence binding, protein channel activity, role in protein import into mitochondrial inner membrane and mitochondrial inner membrane protein insertion complex, plasma membrane localization	Mitochondrial import inner membrane translocase, subunit TIM22 [U]
**AN8074.3**	**ANIA_08074**	**0.30**		Has domain(s) with predicted ubiquitin thiolesterase activity, zinc ion binding activity and role in ubiquitin-dependent protein catabolic process	Ubiquitin carboxyl-terminal hydrolase [O]
**AN8110.3**	**ANIA_08110**	**0.45**		Putative short-chain dehydrogenase/reductase family protein; predicted secondary metabolism gene cluster member	Reductases with broad range of substrate specificities [R]
**AN8122.3**	**ANIA_08122**	**0.08**		Ortholog(s) have role in cellular response to drug, hexose transport, pathogenesis	Synaptic vesicle transporter SVOP and related transporters (major facilitator superfamily) [R]
**AN8124.3**	**ANIA_08124**	**0.28**		Ortholog of A. oryzae RIB40 : AO090012001039, Aspergillus glaucus : Aspgl1_0152256, Aspergillus sydowii : Aspsy1_0211409 and Aspergillus terreus NIH2624 : ATET_09335	Uncharacterized conserved protein [S]
**AN8132.3**	**ANIA_08132**	**0.45**		Has domain(s) with predicted metallopeptidase activity, zinc ion binding activity and role in proteolysis	
**AN8134.3**	**ANIA_08134**	**0.13**		Has domain(s) with predicted transferase activity, transferring acyl groups other than amino-acyl groups activity	
**AN8142.3**	**ANIA_08142**	**0.41**		Putative UbiA-like prenyltransferase; transcript repressed by nitrate	Para-hydroxybenzoate-polyprenyl transferase [H]
**AN8167.3**	**ANIA_08167**	**0.45**		Ortholog of A. fumigatus Af293 : Afu5g02950, A. niger CBS 513.88 : An09g05040, A. oryzae RIB40 : AO090102000494, Aspergillus wentii : Aspwe1_0030018 and Aspergillus sydowii : Aspsy1_0061808	
**AN8172.3**	**ANIA_08172**	**0.42**		Has domain(s) with predicted catalytic activity, pyridoxal phosphate binding activity and role in biosynthetic process	Aromatic amino acid aminotransferase and related proteins [E]
**AN8188.3**	**ANIA_08188**	**0.48**		Putative GTP cyclohydrolase I with a predicted role in folate biosynthesis	GTP cyclohydrolase I [H]
**AN8191.3**	**ANIA_08191**	**0.45**		Has domain(s) with predicted translation initiation factor activity, role in translational initiation and cytoplasm localization	Predicted mRNA cap-binding protein related to eIF-4E [J]
**AN8201.3**	**ANIA_08201**	**0.49**		Putative DNA helicase; transcript upregulated in response to camptothecin	RNA polymerase II K initiation/nucleotide excision repair factor TFIIH, 3'-5' helicase subunit SSL2 [K, L]

**AN8202.3**	**ANIA_08202**	**0.45**		Ortholog of A. fumigatus Af293 : Afu5g03340, A. niger CBS 513.88 : An09g05655, A. oryzae RIB40 : AO090102000539, Aspergillus wentii : Aspwe1_0115517 and Aspergillus sydowii : Aspsy1_0161584	
**AN8203.3**	**ANIA_08203**	**0.48**		Ortholog(s) have NAD+ synthase (glutamine-hydrolyzing) activity, hydrolase activity, acting on carbon-nitrogen (but not peptide) bonds, in linear amides activity, role in NAD biosynthetic process and cytosol, nucleus localization	Predicted NAD synthase, contains CN hydrolase domain [H, R]
**AN8204.3**	**ANIA_08204**	**0.47**	ndxC	Putative nudix hydrolase	NADH pyrophosphatase I of the Nudix family of hydrolases [L]
**AN8212.3**	**ANIA_08212**	**0.32**		Ortholog(s) have 5'-flap endonuclease activity, role in DNA-dependent DNA replication and Slx1-Slx4 complex localization	GIY-YIG type nuclease [R]
**AN8213.3**	**ANIA_08213**	**0.38**		Putative dTMP kinase with a predicted role in pyrimidine metabolism	Thymidylate kinase/adenylate kinase [F]
**AN8219.3**	**ANIA_08219**	**0.33**		Putative plasma membrane ATPase with a predicted role in energy metabolism	Plasma membrane H+-transporting ATPase [P]
**AN8232.3**	**ANIA_08232**	**0.45**		Subunit of the SAGA transcriptional regulatory complex	K initiation factor TFIID, subunit TAF6 (also component of histone acetyltransferase SAGA) [K]
**AN8246.3**	**ANIA_08246**	**0.50**	srpA	Putative signal recognition particle protein	Signal recognition particle, subunit Srp54 [U]
**AN8249.3**	**ANIA_08249**	**0.12**		Putative 2,3-oxidosqualene-lanosterol cyclase with a predicted role in sterol metabolism	Oxidosqualene-lanosterol cyclase and related proteins [I]
**AN8258.3**	**ANIA_08258**	**0.40**		Putative ubiquitin-conjugating enzyme; ortholog of S. cerevisiae Ubc7p	Ubiquitin-protein ligase [O]
**AN8268.3**	**ANIA_08268**	**0.39**		Putative RasGAP SH3 binding protein, contains NTF2 and RRM domains; expression reduced after exposure to farnesol	RasGAP SH3 binding protein rasputin, contains NTF2 and RRM domains [T]
**AN8275.3**	**ANIA_08275**	**0.37**	citA	Mitochondrial citrate synthase with a predicted role in the TCA cycle	Citrate synthase [C]
**AN8301.3**	**ANIA_08301**	**0.47**		Protein of unknown function	Uncharacterized conserved protein [S]
**AN8306.3**	**ANIA_08306**	**0.49**		Protein of unknown function	
**AN8309.3**	**ANIA_08309**	**0.35**	CYP682B1	Putative cytochrome P450	Cytochrome P450 CYP3/CYP5/CYP6/CYP9 subfamilies [Q]
**AN8315.3**	**ANIA_08315**	**0.34**		Protein of unknown function	
**AN8335.3**	**ANIA_08335**	**0.08**		Ortholog of A. oryzae RIB40 : AO090038000479, AO090009000246, Aspergillus flavus NRRL 3357 : AFL2T_10422, Aspergillus versicolor : Aspve1_0046662 and Aspergillus sydowii : Aspsy1_0136121	
**AN8355.3**	**ANIA_08355**	**0.20**		Has domain(s) with predicted DNA binding, sequence-specific DNA binding RNA polymerase II transcription factor activity, zinc ion binding activity and role in regulation of transcription, DNA-dependent, transcription, DNA-templated	
**AN8356.3**	**ANIA_08356**	**0.26**		Has domain(s) with predicted nucleotide binding, oxidoreductase activity, transferase activity, transferring acyl groups other than amino-acyl groups, zinc ion binding activity and role in oxidation-reduction process	Alcohol dehydrogenase, class III [Q]
**AN8357.3**	**ANIA_08357**	**0.20**		Has domain(s) with predicted DNA binding, sequence-specific DNA binding RNA polymerase II transcription factor activity, zinc ion binding activity and role in regulation of transcription, DNA-dependent, transcription, DNA-templated	
**AN8372.3**	**ANIA_08372**	**0.41**		Protein of unknown function	
**AN8390.3**	**ANIA_08390**	**0.30**		GPR1/FUN34/YaaH family member; ethanol- and ethylacetate-induced gene	
**AN8397.3**	**ANIA_08397**	**0.39**		Has domain(s) with predicted oxidoreductase activity, acting on CH-OH group of donors activity and role in oxidation-reduction process	Glucose dehydrogenase/choline dehydrogenase/mandelonitrile lyase (GMC oxidoreductase family) [R]
**AN8398.3**	**ANIA_08398**	**0.21**		Has domain(s) with predicted nucleotide binding, oxidoreductase activity and role in metabolic process	Predicted dehydrogenase [Q]
**AN8411.3**	**ANIA_08411**	**0.36**	apdE	Putative cytochrome P450 monooxygenase; member of the aspyridone (apd) gene cluster	Cytochrome P450 CYP4/CYP19/CYP26 subfamilies [Q, I]
**AN8431.3**	**ANIA_08431**	**0.49**		Has domain(s) with predicted sequence-specific DNA binding RNA polymerase II transcription factor activity, zinc ion binding activity, role in regulation of transcription, DNA-dependent and nucleus localization	
**AN8432.3**	**ANIA_08432**	**0.38**		Has domain(s) with predicted catalytic activity	
**AN8441.3**	**ANIA_08441**	**0.48**		Has domain(s) with predicted DNA binding, sequence-specific DNA binding RNA polymerase II transcription factor activity, zinc ion binding activity and role in regulation of transcription, DNA-dependent, transcription, DNA-templated	
**AN8449.3**	**ANIA_08449**	**0.15**		Has domain(s) with predicted electron carrier activity, heme binding, molybdenum ion binding, oxidoreductase activity and role in oxidation-reduction process	Sulfite oxidase, molybdopterin-binding component [C]
**AN8465.3**	**ANIA_08465**	**0.05**	rhaF	Putative alpha-L-rhamnosidase; expression upregulated after exposure to farnesol	
**AN8472.3**	**ANIA_08472**	**0.45**		Putative alpha-L-arabinofuranosidase	
**AN8473.3**	**ANIA_08473**	**0.45**		Has domain(s) with predicted DNA binding, sequence-specific DNA binding RNA polymerase II transcription factor activity, zinc ion binding activity and role in regulation of transcription, DNA-dependent, transcription, DNA-templated	
**AN8481.3**	**ANIA_08481**	**0.31**		Putative chitinase; glycoside hydrolase family 18 (GH18) protein with a predicted role in chitin	Chitinase [G]
**AN8495.3**	**ANIA_08495**	**0.35**		Ortholog(s) have endoplasmic reticulum localization	
**AN8497.3**	**ANIA_08497**	**0.45**		Has domain(s) with predicted carbon-sulfur lyase activity and role in metabolic process	
**AN8503.3**	**ANIA_08503**	**0.40**		Ortholog of A. niger CBS 513.88 : An13g03080, Aspergillus wentii : Aspwe1_0177501, Aspergillus versicolor : Aspve1_0046851 and Aspergillus sydowii : Aspsy1_0159981	
**AN8506.3**	**ANIA_08506**	**0.36**		Putative transcription factor; predicted role in secondary metabolite production	
**AN8509.3**	**ANIA_08509**	**0.36**		Putative transcription factor; predicted role in secondary metabolite production	
**AN8521.3**	**ANIA_08521**	**0.31**		Gene adjacent to tdi (terrequinone biosynthesis) gene cluster; terrequinone A production normal in null mutant	Kynurenine 3-monooxygenase and related flavoprotein monooxygenases [C, R]
**AN8526.3**	**ANIA_08526**	**0.44**		Ortholog(s) have role in ergot alkaloid biosynthetic process	
**AN8532.3**	**ANIA_08532**	**0.31**		Predicted glycosylphosphatidylinositol (GPI)-anchored protein	
**AN8548.3**	**ANIA_08548**	**0.48**		Ortholog of A. nidulans FGSC A4 : AN6924, AN5943, AN8512, AN0857, AN8661, AN4642 and A. fumigatus Af293 : Afu1g15290, Afu3g00850, Afu4g08850, Afu7g00920	
**AN8553.3**	**ANIA_08553**	**0.20**		Putative catalase with a predicted role in gluconic acid and gluconate metabolism	Catalase [P]
**AN8561.3**	**ANIA_08561**	**0.34**		Has domain(s) with predicted nucleotide binding, oxidoreductase activity and role in metabolic process	Reductases with broad range of substrate specificities [R]
**AN8579.3**	**ANIA_08579**	**0.44**		Has domain(s) with predicted oxidoreductase activity and role in oxidation-reduction process	
**AN8592.3**	**ANIA_08592**	**0.19**		Has domain(s) with predicted oxidoreductase activity and role in metabolic process	Kynurenine 3-monooxygenase and related flavoprotein monooxygenases [C, R]
**AN8607.3**	**ANIA_08607**	**0.16**		Ortholog of A. nidulans FGSC A4 : AN1754, A. fumigatus Af293 : Afu3g02050, A. niger CBS 513.88 : An11g06690, An18g01930 and A. oryzae RIB40 : AO090001000144	
**AN8631.3**	**ANIA_08631**	**0.42**	FKBP2	Protein with similarity to FK506 binding proteins (FKBPs)	FKBP-type peptidyl-prolyl cis-trans isomerase [O]
**AN8633.3**	**ANIA_08633**	**0.49**		Protein of unknown function	
**AN8634.3**	**ANIA_08634**	**0.30**		Has domain(s) with predicted nucleotide binding activity	
**AN8638.3**	**ANIA_08638**	**0.23**	cetJ	Transcript enriched in dormant conidia; induced by light in in developmentally competent mycelia	
**AN8639.3**	**ANIA_08639**	**0.38**		Putative alpha,alpha-trehalose-phosphate synthase (UDP-forming) with a predicted role in trehalose biosynthesis	Trehalose-6-phosphate synthase component TPS1 and related subunits [G]
**AN8640.3**	**ANIA_08640**	**0.10**	conF	Ortholog of N. crassa conF, light-induced transcript expressed during conidiation in N. crassa; double conF conJ deletion results in increased cellular glycerol or erythritol leading to delayed germination and desiccation resistance	
**AN8641.3**	**ANIA_08641**	**0.41**		Transcript induced by light in in developmentally competent mycelia	
**AN8642.3**	**ANIA_08642**	**0.19**		Has domain(s) with predicted nucleotide binding, oxidoreductase activity	Dimeric dihydrodiol dehydrogenase [G, Q]

**AN8643.3**	**ANIA_08643**	**0.14**		Ortholog(s) have role in cellular response to heat, cellular response to hydrogen peroxide	
**AN8646.3**	**ANIA_08646**	**0.37**		Has domain(s) with predicted oxidoreductase activity, zinc ion binding activity and role in oxidation-reduction process	
**AN8652.3**	**ANIA_08652**	**0.43**		Protein of unknown function	
**AN8664.3**	**ANIA_08664**	**0.50**		Ortholog(s) have cytosol, nucleus localization	Predicted hydrolase related to dienelactone hydrolase [R]
**AN8666.3**	**ANIA_08666**	**0.47**		Has domain(s) with predicted sequence-specific DNA binding RNA polymerase II transcription factor activity, zinc ion binding activity, role in regulation of transcription, DNA-dependent and nucleus localization	
**AN8681.3**	**ANIA_08681**	**0.48**		Has domain(s) with predicted aspartic-type endopeptidase activity and integral to membrane localization	Uncharacterized conserved protein [S]
**AN8708.3**	**ANIA_08708**	**0.39**		Protein of unknown function	
**AN8709.3**	**ANIA_08709**	**0.48**		Putative aspartate transaminase with a predicted role in alanine and aspartate metabolism	Aspartate aminotransferase/Glutamic oxaloacetic transaminase AAT2/GOT1 [E]
**AN8714.3**	**ANIA_08714**	**0.29**		Protein of unknown function	
**AN8728.3**	**ANIA_08728**	**0.26**		Ortholog of A. oryzae RIB40 : AO090012000831, Aspergillus brasiliensis : Aspbr1_0201195, Aspergillus glaucus : Aspgl1_0037582 and Aspergillus versicolor : Aspve1_0192004	
**AN8735.3**	**ANIA_08735**	**0.43**		Has domain(s) with predicted DNA binding, sequence-specific DNA binding RNA polymerase II transcription factor activity, zinc ion binding activity and role in regulation of transcription, DNA-dependent, transcription, DNA-templated	
**AN8777.3**	**ANIA_08777**	**0.45**	amdS	Acetamidase, produces ammonium and acetate from acetamide, allowing utilization of acetamide as sole carbon or nitrogen source; transcript induced under low nitrogen conditions	Amidases [J, I, T]
**AN8793.3**	**ANIA_08793**	**0.46**	carC	Putative succinate dehydrogenase (ubiquinone) with a predicted role in the TCA cycle; palA-dependent expression independent of pH	Succinate dehydrogenase, cytochrome b subunit [G]
**AN8807.3**	**ANIA_08807**	**0.46**	rtt109	Putative Rtt109-type acetyltransferase	Uncharacterized conserved protein [S]
**AN8812.3**	**ANIA_08812**	**0.28**		Has domain(s) with predicted ATP binding, ATPase activity	Lipid exporter ABCA1 and related proteins, ABC superfamily [I, R]
**AN8815.3**	**ANIA_08815**	**0.48**		Putative isoflavone reductase family protein; intracellular, menadione stress-induced protein	
**AN8822.3**	**ANIA_08822**	**0.48**		Ortholog(s) have cytosol, nucleus localization	Lysophospholipase [I]
**AN8824.3**	**ANIA_08824**	**0.48**		Ortholog(s) have ribosomal large subunit binding activity	Translation initiation factor 6 (eIF-6) [J]
**AN8829.3**	**ANIA_08829**	**0.14**		Ortholog of A. oryzae RIB40 : AO090009000665, Aspergillus wentii : Aspwe1_0054722, Aspergillus sydowii : Aspsy1_0117087 and Aspergillus terreus NIH2624 : ATET_02810, ATET_09435	
**AN8843.3**	**ANIA_08843**	**0.42**		Putative homoserine kinase with a predicted role in glycine, serine, and threonine metabolism	Homoserine kinase [E]
**AN8854.3**	**ANIA_08854**	**0.28**		Ortholog(s) have L-glutamine transmembrane transporter activity, L-isoleucine transmembrane transporter activity, L-tyrosine transmembrane transporter activity and role in amino acid transport, vacuolar transport	Amino acid transporters [E]
**AN8861.3**	**ANIA_08861**	**0.48**		Ortholog(s) have GTP diphosphatase activity, ITP diphosphatase activity, UTP diphosphatase activity, XTP diphosphatase activity, dATP pyrophosphohydrolase activity and dCTP diphosphatase activity, more	Inosine triphosphate pyrophosphatase [F]
**AN8866.3**	**ANIA_08866**	**0.46**		Putative phosphoglycerate dehydrogenase with a predicted role in glycine, serine, and threonine metabolism	D-3-phosphoglycerate dehydrogenase, D-isomer-specific 2-hydroxy acid dehydrogenase superfamily [E]
**AN8869.3**	**ANIA_08869**	**0.49**		Putative uracil phosphoribosyltransferase with a predicted role in pyrimidine metabolism	Armadillo/beta-Catenin/plakoglobin [T, Z]
**AN8874.3**	**ANIA_08874**	**0.42**		Ortholog(s) have GTPase activity, protein homodimerization activity	Vacuolar sorting protein VPS1, dynamin, and related proteins [U, R]
**AN8877.3**	**ANIA_08877**	**0.47**		Predicted Rac guanine nucleotide exchange factor (Rac GEF)	Regulator of Rac1, required for phagocytosis and cell migration [T]
**AN8891.3**	**ANIA_08891**	**0.31**	pgxB	Putative exopolygalacturonase	
**AN8910.3**	**ANIA_08910**	**0.37**		Putative polyketide synthase (PKS)	Animal-type fatty acid synthase and related proteins [I]
**AN8918.3**	**ANIA_08918**	**0.17**		Putative transcription factor; predicted role in secondary metabolite production	
**AN8919.3**	**ANIA_08919**	**0.08**	CYP540A2	Putative cytochrome P450 monooxygenase	Cytochrome P450 CYP4/CYP19/CYP26 subfamilies [Q, I]
**AN8920.3**	**ANIA_08920**	**0.17**		Putative cytochrome b5; ortholog of A. fumigatus Afu5g10060	NADH-cytochrome b-5 reductase [H, C]
**AN8922.3**	**ANIA_08922**	**0.33**		Protein of unknown function	
**AN8927.3**	**ANIA_08927**	**0.37**		Ortholog of A. fumigatus Af293 : Afu5g01120, A. oryzae RIB40 : AO090003001298, Aspergillus flavus NRRL 3357 : AFL2T_01773 and Neosartorya fischeri NRRL 181 : NFIA_041050	
**AN8936.3**	**ANIA_08936**	**0.44**		Has domain(s) with predicted role in transmembrane transport and integral to membrane localization	Permease of the major facilitator superfamily [G]
**AN8937.3**	**ANIA_08937**	**0.49**		Putative 3-oxoacyl-(acyl-carrier-protein) reductase 2	Reductases with broad range of substrate specificities [R]
**AN8943.3**	**ANIA_08943**	**0.26**		Ortholog of A. fumigatus Af293 : Afu2g00900, Afu6g11560, A. niger CBS 513.88 : An01g11690, An03g01300, A. oryzae RIB40 : AO090113000175, AO090023000422 and Aspergillus wentii : Aspwe1_0024673, Aspwe1_0108934	
**AN8950.3**	**ANIA_08950**	**0.35**		Protein of unknown function	
**AN8951.3**	**ANIA_08951**	**0.49**		Ortholog of A. nidulans FGSC A4 : AN6413, AN6419, AN6946, AN5312, AN5664, AN8328, AN3241, AN10369, AN1930, AN2386, AN1738, AN11159, AN8984, AN1540, AN8971, AN9387, AN9266, AN9306, AN1317, AN10886, AN7232, AN7774, AN12202	
**AN8956.3**	**ANIA_08956**	**0.26**		Has domain(s) with predicted inorganic phosphate transmembrane transporter activity, role in phosphate ion transport and membrane localization	Na+/Pi symporter [P]
**AN8961.3**	**ANIA_08961**	**0.40**		Protein of unknown function	
**AN8962.3**	**ANIA_08962**	**0.43**		Has domain(s) with predicted solute:hydrogen antiporter activity, role in cation transport, transmembrane transport and integral to membrane localization	
**AN8966.3**	**ANIA_08966**	**0.30**		Has domain(s) with predicted neurotransmitter:sodium symporter activity, role in neurotransmitter transport and integral to membrane localization	Sodium-neurotransmitter symporter [T]
**AN8973.3**	**ANIA_08973**	**0.48**		Has domain(s) with predicted sequence-specific DNA binding RNA polymerase II transcription factor activity, zinc ion binding activity, role in regulation of transcription, DNA-dependent and nucleus localization	
**AN8974.3**	**ANIA_08974**	**0.28**		Protein of unknown function	
**AN8975.3**	**ANIA_08975**	**0.24**		Ortholog of Aspergillus versicolor : Aspve1_0045995 and Aspergillus sydowii : Aspsy1_0060497	Predicted glutamine synthetase [F]
**AN8978.3**	**ANIA_08978**	**0.45**	alcR	Transcription factor involved in positive regulation of the ethanol regulon; contains Zn(II)2Cys6 DNA-binding domain	
**AN8997.3**	**ANIA_08997**	**0.10**		Protein of unknown function	
**AN9002.3**	**ANIA_09002**	**0.36**		Has domain(s) with predicted nucleotide binding, oxidoreductase activity and role in metabolic process	Reductases with broad range of substrate specificities [R]
**AN9015.3**	**ANIA_09015**	**0.27**		Protein of unknown function	
**AN9027.3**	**ANIA_09027**	**0.36**		Has domain(s) with predicted hydrolase activity	
**AN9028.3**	**ANIA_09028**	**0.29**		Has domain(s) with predicted nucleotide binding activity	
**AN9034.3**	**ANIA_09034**	**0.05**		Putative aldehyde dehydrogenase with a predicted role in two-carbon compound metabolism or penicillin biosynthesis	Aldehyde dehydrogenase [C]
**AN9038.3**	**ANIA_09038**	**0.16**		Has domain(s) with predicted role in Mo-molybdopterin cofactor biosynthetic process	Molybdopterin biosynthesis protein [H]
**AN9048.3**	**ANIA_09048**	**0.21**	hk-8-7	Putative histidine-containing phosphotransfer protein	Sensory transduction histidine kinase [T]
**AN9057.3**	**ANIA_09057**	**0.49**		Ortholog of A. fumigatus Af293 : Afu7g02600, A. niger CBS 513.88 : An09g03040, A. oryzae RIB40 : AO090311000001, Aspergillus wentii : Aspwe1_0052567 and Aspergillus sydowii : Aspsy1_0049967	

AN9060.3	ANIA_09060	0.48		Ortholog(s) have ATPase activator activity and role in endocytosis, rRNA processing, regulation of cell size, ribosomal large subunit biogenesis, ribosomal subunit export from nucleus	Molecular chaperone (DnaJ superfamily) [O]
AN9063.3	ANIA_09063	0.33		Ortholog(s) have cytosol localization	Oxysterol-binding protein [I]
AN9064.3	ANIA_09064	0.32		Xylitol dehydrogenase with a predicted role in amino acid or carbohydrate metabolism; putative D-xylulose reductase; transcriptionally induced by growth on xylose	Sorbitol dehydrogenase [Q]
AN9074.3	ANIA_09074	0.41		Ortholog of A. fumigatus Af293 : Afu7g02440, A. oryzae RIB40 : AO090038000622, Aspergillus wentii : Aspwe1_0052578, Aspergillus sydowii : Aspsy1_0093793 and Aspergillus terreus NIH2624 : ATET_01872	
AN9078.3	ANIA_09078	0.47		Has domain(s) with predicted NADH dehydrogenase (ubiquinone) activity and mitochondrion localization	
AN9085.3	ANIA_09085	0.42		Ortholog(s) have U5 snRNP, spliceosomal complex localization	U5 snRNP-specific protein-like factor and related proteins [A]
AN9093.3	ANIA_09093	0.32		Ortholog(s) have mRNA binding, single-stranded DNA binding, translation regulator activity and role in cellular response to cold, cellular response to drug, cellular response to osmotic stress, positive regulation of translation	E3 ubiquitin ligase interacting with arginine methyltransferase [O]
AN9113.3	ANIA_09113	0.20		Putative F-box protein	Molecular co-chaperone STI1 [O]
AN9115.3	ANIA_09115	0.50		Ortholog of A. niger CBS 513.88 : An10g00220, A. oryzae RIB40 : AO090010000382, Aspergillus wentii : Aspwe1_0182832, Aspergillus versicolor : Aspve1_0180171 and Aspergillus niger ATCC 1015 : 192862-mRNA	
AN9116.3	ANIA_09116	0.41		Ortholog(s) have nicotinate phosphoribosyltransferase activity and role in cell-abiotic substrate adhesion, chromatin silencing at rDNA, chromatin silencing at telomere, nicotinate nucleotide salvage, replicative cell aging	Nicotinic acid phosphoribosyltransferase [H]
AN9117.3	ANIA_09117	0.32		Has domain(s) with predicted DNA binding, zinc ion binding activity, role in transcription, DNA-templated and nucleus localization	
AN9123.3	ANIA_09123	0.43		Ortholog of Aspergillus brasiliensis : Aspbr1_0040215, Aspbr1_0678615, Aspergillus wentii : Aspwe1_0041389, Aspergillus versicolor : Aspve1_0140141 and Aspergillus niger ATCC 1015 : 183284-mRNA	Putative translation initiation inhibitor UK114/IBM1 [J]
AN9125.3	ANIA_09125	0.48		RNA polymerase III, subunit C34; locus contains the conserved upstream open reading frame (uORF) AN9125-uORF	RNA polymerase III, subunit C34 [K]
AN9140.3	ANIA_09140	0.44		Putative MFS-type amino acid transporter; transcript repressed by nitrogen limitation	
AN9143.3	ANIA_09143	0.48	cnxG	Putative molybdopterin synthase small subunit involved in molybdenum cofactor biosynthesis; molybdopterin cofactor required for the activity of nitrate reductase	
AN9151.3	ANIA_09151	0.33		Ortholog(s) have role in Group I intron splicing and mitochondrion localization	Exosomal 3'-5' exoribonuclease complex, subunit Rrp44/Dis3 [J]
AN9163.3	ANIA_09163	0.09		Ortholog of A. fumigatus Af293 : Afu8g07190, A. niger CBS 513.88 : An14g07150, A. oryzae RIB40 : AO090138000059, Aspergillus wentii : Aspwe1_0078799 and Aspergillus sydowii : Aspsy1_0061494	
AN9164.3	ANIA_09164	0.49		Has domain(s) with predicted nucleotide binding, oxidoreductase activity	
AN9179.3	ANIA_09179	0.29		Ortholog(s) have cytoplasm localization	Voltage-gated shaker-like K+ channel, subunit beta/KCNAB [C]
AN9194.3	ANIA_09194	0.43	cetL	Transcript enriched in dormant conidia	
AN9195.3	ANIA_09195	0.46		Ortholog of A. fumigatus Af293 : Afu3g11890, A. niger CBS 513.88 : An01g14950, A. oryzae RIB40 : AO090010000214, Neosartorya fischeri NRRL 181 : NFIA_065430 and Aspergillus wentii : Aspwe1_0130022	
AN9241.3	ANIA_09241	0.41		Has domain(s) with predicted zinc ion binding activity and role in lipid metabolic process	
AN9242.3	ANIA_09242	0.28		Protein of unknown function; transcript is induced by nitrate	Flavin-containing monooxygenase [Q]
AN9267.3	ANIA_09267	0.42		Ortholog(s) have peroxisome localization	
AN9272.3	ANIA_09272	0.31		Ortholog of A. nidulans FGSC A4 : AN0698, A. fumigatus Af293 : Afu1g13610, Afu4g09370, A. niger CBS 513.88 : An08g06690, An07g08670 and A. oryzae RIB40 : AO090012000513	
AN9284.3	ANIA_09284	0.20		Has domain(s) with predicted nucleotide binding activity	Reductases with broad range of substrate specificities [R]
AN9289.3	ANIA_09289	0.48		Has domain(s) with predicted role in transmembrane transport and integral to membrane localization	Synaptic vesicle transporter SV2 (major facilitator superfamily) [R]
AN9322.3	ANIA_09322	0.23		Has domain(s) with predicted nucleotide binding activity	
AN9330.3	ANIA_09330	0.29		Ortholog(s) have lipase activity	Arylacetamide deacetylase [V]
AN9350.3	ANIA_09350	0.18		Protein of unknown function	Iron/ascorbate family oxidoreductases [Q, R]
AN9352.3	ANIA_09352	0.44		Ortholog of A. nidulans FGSC A4 : AN4485, AN11792, A. fumigatus Af293 : Afu3g00140, Afu4g03310, Afu8g01440 and A. niger CBS 513.88 : An12g02490, An13g02300, An09g03710, An12g06070, An12g02990, An14g01970	
AN9357.3	ANIA_09357	0.36		Ortholog(s) have cytosol, nucleus localization	Predicted dehydrogenase [Q]
AN9370.3	ANIA_09370	0.42		Ortholog(s) have endoplasmic reticulum localization	Predicted transporter (major facilitator superfamily) [R]
AN9378.3	ANIA_09378	0.46		Has domain(s) with predicted nucleotide binding, oxidoreductase activity and role in metabolic process	Predicted dehydrogenase [Q]
AN9386.3	ANIA_09386	0.29		Ortholog of Aspergillus brasiliensis : Aspbr1_0139979, Aspergillus kawachii : Aspka1_0182863, Aspergillus wentii : Aspwe1_0051750 and Aspergillus versicolor : Aspve1_0057888	
AN9387.3	ANIA_09387	0.50		Ortholog of A. nidulans FGSC A4 : AN6413, AN6419, AN6946, AN5312, AN5664, AN8328, AN3241, AN10369, AN1930, AN2386, AN1738, AN11159, AN8984, AN8951, AN1540, AN8971, AN9266, AN9306, AN1317, AN10886, AN7232, AN7774, AN12202	
AN9401.3	ANIA_09401	0.33		Ortholog(s) have mitochondrion localization	Zn2+-binding dehydrogenase (nuclear receptor binding factor-1) [K, C]
AN9412.3	ANIA_09412	0.45		Protein of unknown function	
AN9420.3	ANIA_09420	0.46	cyp4	Putative peptidyl-prolyl cis-trans isomerase H (PPIase)	U-snRNP-associated cyclophilin type peptidyl-prolyl cis-trans isomerase [O]
AN9423.3	ANIA_09423	0.44		Ortholog of A. fumigatus Af293 : Afu8g03910, A. niger CBS 513.88 : An16g09290, Neosartorya fischeri NRRL 181 : NFIA_096620, Aspergillus wentii : Aspwe1_0053600 and Aspergillus versicolor : Aspve1_0422328	
AN9446.3	ANIA_09446	0.39	panK	Putative pantothenate kinase with a predicted role in Coenzyme A and pantothenate biosynthesis	Pantothenate kinase PanK and related proteins [H]
AN9462.3	ANIA_09462	0.43		Ortholog of A. fumigatus Af293 : Afu2g03630, A. niger CBS 513.88 : An07g08200, Neosartorya fischeri NRRL 181 : NFIA_035990 and Aspergillus wentii : Aspwe1_0110313	
AN9465.3	ANIA_09465	0.40		Ortholog(s) have structural constituent of ribosome activity, role in cytoplasmic translation and cytosolic large ribosomal subunit, nucleus localization	60S ribosomal protein L9 [J]
AN9515.3	ANIA_09515	0.31		Has domain(s) with predicted phosphatidylinositol binding activity and role in cell communication	
AN10006.3	ANIA_10006	0.34		Has domain(s) with predicted zinc ion binding activity	Predicted E3 ubiquitin ligase, integral peroxisomal membrane protein [O]
AN10012.3	ANIA_10012	0.45		Ortholog(s) have role in iron-sulfur cluster assembly and mitochondrial matrix localization	K factor, component of CCR4 Kal complex [K]
AN10020.3	ANIA_10020	0.50		Ortholog(s) have role in proteasome-mediated ubiquitin-dependent protein catabolic process and cytosol, nucleus localization	26S proteasome regulatory complex, subunit RPN1/PSMD2 [O]
AN10021.3	ANIA_10021	0.20	mdpA	Regulatory protein; member of the monodictyphenone secondary metabolite biosynthesis gene cluster; similar to aflatoxin coactivator aflJ and O-methyltransferases; required for biosynthesis of monodictyphenone, a prenyl xanthone precursor	

**AN10025.3**	**ANIA_10025**	**0.37**		Ortholog of A. fumigatus Af293 : Afu5g11280, A. niger CBS 513.88 : An01g02420, A. oryzae RIB40 : AO090026000715, Neosartorya fischeri NRRL 181 : NFIA_075990 and Aspergillus clavatus NRRL 1 : ACLA_014360	
**AN10030.3**	**ANIA_10030**	**0.48**		Putative alkaline serine protease; predicted glycosylphosphatidylinositol (GPI)-anchored protein	Subtilisin-related protease/Vacuolar protease B [O]
**AN10032.3**	**ANIA_10032**	**0.39**		Ortholog(s) have proton-transporting ATP synthase activity, rotational mechanism, proton-transporting ATPase activity, rotational mechanism activity and role in ATP synthesis coupled proton transport	Mitochondrial F1F0-ATP synthase, subunit delta/ATP16 [C]
**AN10039.3**	**ANIA_10039**	**0.40**		Putative histidine acid phosphatase	Lysosomal & prostatic acid phosphatases [I]
**AN10040.3**	**ANIA_10040**	**0.10**		Ortholog of A. fumigatus Af293 : Afu5g09180, A. niger CBS 513.88 : An07g03930, A. oryzae RIB40 : AO090020000514, Aspergillus wentii : Aspwe1_0035291 and Aspergillus terreus NIH2624 : ATET_06551	
**AN10079.3**	**ANIA_10079**	**0.45**	ureB	Urease, involved in utilization of urea as a nitrogen source	
**AN10088.3**	**ANIA_10088**	**0.47**		Ortholog(s) have protein phosphatase type 1 activator activity, role in positive regulation of mitotic sister chromatid separation, regulation of mitotic metaphase/anaphase transition and cytosol, nucleus localization	Protein phosphatase 1, regulatory subunit, and related proteins [T]
**AN10098.3**	**ANIA_10098**	**0.27**		Putative zinc-binding oxidoreductase; intracellular, menadione stress-induced protein	Zinc-binding oxidoreductase [C, R]
**AN10111.3**	**ANIA_10111**	**0.47**		Ortholog(s) have peptidyl-prolyl cis-trans isomerase activity, protein phosphatase type 2A regulator activity	Phosphotyrosyl phosphatase activator [D, T]
**AN10122.3**	**ANIA_10122**	**0.42**		Has domain(s) with predicted nucleotide binding, oxidoreductase activity and role in metabolic process	Reductases with broad range of substrate specificities [R]
**AN10130.3**	**ANIA_10130**	**0.46**		Ortholog(s) have structural constituent of ribosome activity and cytosolic small ribosomal subunit localization	40S ribosomal protein S25 [J]
**AN10131.3**	**ANIA_10131**	**0.24**	pyrD	Dihydroorotase, catalyzes the third step in pyrimidine biosynthesis	Dihydroorotase [F]
**AN10151.3**	**ANIA_10151**	**0.41**		Possible pseudogene, similar to Zn(II)2Cys6 transcription factor	
**AN10159.3**	**ANIA_10159**	**0.45**		Ortholog of Aspergillus wentii : Aspwe1_0172079, Aspergillus clavatus NRRL 1 : ACLA_023190, Aspergillus zonatus : Aspzo1_0012961 and Aspergillus carbonarius ITEM 5010 : Acar5010_000620	
**AN10169.3**	**ANIA_10169**	**0.44**		Short-chain dehydrogenase; transcriptionally induced by growth on xylose	Reductases with broad range of substrate specificities [R]
**AN10170.3**	**ANIA_10170**	**0.49**		Ortholog(s) have ATPase activity, role in negative regulation of G2/M transition of mitotic cell cycle and cytosol, nucleus localization	Predicted GTP-binding protein (ODN superfamily) [R]
**AN10174.3**	**ANIA_10174**	**0.25**		Ortholog of A. fumigatus Af293 : Afu1g10090, A. oryzae RIB40 : AO090038000427, Neosartorya fischeri NRRL 181 : NFIA_015560 and Aspergillus wentii : Aspwe1_0168735	
**AN10179.3**	**ANIA_10179**	**0.38**		Ortholog of A. fumigatus Af293 : Afu1g09650, A. oryzae RIB40 : AO090012000878, Aspergillus wentii : Aspwe1_0036407, Aspergillus clavatus NRRL 1 : ACLA_025850 and Aspergillus niger ATCC 1015 : 38576-mRNA	
**AN10209.3**	**ANIA_10209**	**0.42**		Ortholog of A. oryzae RIB40 : AO090005000644, Neosartorya fischeri NRRL 181 : NFIA_097790, Aspergillus kawachii : Aspka1_0179507 and Aspergillus clavatus NRRL 1 : ACLA_058380	
**AN10222.3**	**ANIA_10222**	**0.33**		Ortholog(s) have glucose-6-phosphate 1-epimerase activity and cytoplasm, nucleus localization	Uncharacterized enzymes related to aldose 1-epimerase [G]
**AN10232.3**	**ANIA_10232**	**0.36**		Ortholog(s) have nucleolus localization	
**AN10256.3**	**ANIA_10256**	**0.41**		Has domain(s) with predicted RNA binding, RNA-directed DNA polymerase activity, role in DNA integration, RNA-dependent DNA replication and nucleus localization	
**AN10264.3**	**ANIA_10264**	**0.37**		Ortholog of A. oryzae RIB40 : AO090003000305, Aspergillus wentii : Aspwe1_0046218, Aspergillus versicolor : Aspve1_0080260 and Aspergillus clavatus NRRL 1 : ACLA_089630	
**AN10287.3**	**ANIA_10287**	**0.31**		Has domain(s) with predicted oleate hydratase activity and role in fatty acid metabolic process	
**AN10292.3**	**ANIA_10292**	**0.29**		Protein of unknown function	
**AN10297.3**	**ANIA_10297**	**0.38**		Protein with similarity to nonribosomal peptide synthases (NRPS-like); putative acyl CoA synthetase with an NAD-binding domain	Acyl-CoA synthetase [I]
**AN10315.3**	**ANIA_10315**	**0.47**		Ortholog(s) have GTPase activity, ribosome binding activity, role in positive regulation of translation and mitochondrial matrix, mitochondrial ribosome localization	Elongation factor-type GTP-binding protein [J]
**AN10332.3**	**ANIA_10332**	**0.34**		Has domain(s) with predicted role in transmembrane transport and integral to membrane localization	Uncharacterized conserved protein [S]
**AN10334.3**	**ANIA_10334**	**0.45**		Has domain(s) with predicted sequence-specific DNA binding, sequence-specific DNA binding RNA polymerase II transcription factor activity, zinc ion binding activity and role in regulation of transcription, DNA-dependent	K factor MEIS1 and related HOX domain proteins [K]
**AN10343.3**	**ANIA_10343**	**0.36**		Ortholog(s) have sodium:inorganic phosphate symporter activity, role in phosphate ion transmembrane transport and plasma membrane localization	Na+/Pi symporter [P]
**AN10369.3**	**ANIA_10369**	**0.14**		Ortholog of A. nidulans FGSC A4 : AN6413, AN6419, AN6946, AN5312, AN5664, AN8328, AN3241, AN1930, AN2386, AN1738, AN11159, AN8984, AN8951, AN1540, AN8971, AN9387, AN9266, AN9306, AN1317, AN10886, AN7232, AN7774, AN12202	
**AN10370.3**	**ANIA_10370**	**0.37**		Ortholog of A. fumigatus Af293 : Afu3g07980, A. niger CBS 513.88 : An16g01990, A. oryzae RIB40 : AO090005001498, Aspergillus wentii : Aspwe1_0173462 and Aspergillus sydowii : Aspsy1_0042585	
**AN10383.3**	**ANIA_10383**	**0.20**		Possible pseudogene; the predicted pseudogene AN10383 and predicted ORF AN3200 occupy overlapping chromosomal locations in version 4 of the A. nidulans annotation	
**AN10385.3**	**ANIA_10385**	**0.13**		Protein of unknown function	
**AN10408.3**	**ANIA_10408**	**0.49**		Ortholog of A. fumigatus Af293 : Afu3g05850, A. niger CBS 513.88 : An11g10750, A. oryzae RIB40 : AO090020000054, Neosartorya fischeri NRRL 181 : NFIA_071380 and Aspergillus clavatus NRRL 1 : ACLA_033820	
**AN10416.3**	**ANIA_10416**	**0.49**		Putative 60s ribosomal protein similar to subunits L15 and L27; ortholog of S. cerevisiae RPL28; expression reduced after exposure to farnesol; palA-dependent expression independent of pH	60s ribosomal protein L15/L27 [J]
**AN10421.3**	**ANIA_10421**	**0.29**		Ortholog of A. nidulans FGSC A4 : AN3523, A. fumigatus Af293 : Afu1g01460, Afu4g01090, Afu6g13470, Afu8g06620 and A. niger CBS 513.88 : An08g03030	Predicted Zn-dependent hydrolase (beta-lactamase superfamily) [R]
**AN10428.3**	**ANIA_10428**	**0.21**		Has domain(s) with predicted role in transmembrane transport and integral to membrane localization	Permease of the major facilitator superfamily [G]
**AN10441.3**	**ANIA_10441**	**0.46**		Ortholog(s) have cell division site, cytosol, nucleus localization	Uncharacterized conserved protein [S]
**AN10447.3**	**ANIA_10447**	**0.43**		Has domain(s) with predicted transferase activity, transferring acyl groups activity	Carnitine O-acyltransferase CRAT [I]
**AN10463.3**	**ANIA_10463**	**0.49**		Has domain(s) with predicted DNA binding, sequence-specific DNA binding RNA polymerase II transcription factor activity, zinc ion binding activity and role in regulation of transcription, DNA-dependent, transcription, DNA-templated	
**AN10468.3**	**ANIA_10468**	**0.44**		Ortholog(s) have role in cadmium ion transport, zinc ion transport	Fe2+/Zn2+ regulated transporter [P]
**AN10472.3**	**ANIA_10472**	**0.48**		Ortholog(s) have cytosol localization	
**AN10494.3**	**ANIA_10494**	**0.37**		Has domain(s) with predicted nucleotide binding, oxidoreductase activity and role in metabolic process	Dehydrogenases with different specificities (related to short-chain alcohol dehydrogenases) [Q]
**AN10499.3**	**ANIA_10499**	**0.43**		Has domain(s) with predicted CoA-transferase activity and role in ketone body catabolic process	Aldo/keto reductase family proteins [R]
**AN10501.3**	**ANIA_10501**	**0.45**		Ortholog(s) have cytosol, nucleus localization	

**AN10504.3**	**ANIA_10504**	**0.35**		Ortholog(s) have role in conidiophore development, hyphal growth, sporocarp development involved in sexual reproduction, syncytium formation by plasma membrane fusion	
**AN10506.3**	**ANIA_10506**	**0.37**		Ortholog of A. fumigatus Af293 : Afu5g14780, A. niger CBS 513.88 : An18g01640, A. oryzae RIB40 : AO090103000417, Aspergillus versicolor : Aspve1_0173600 and Aspergillus niger ATCC 1015 : 42671-mRNA	
**AN10507.3**	**ANIA_10507**	**0.45**	hsp20	Ortholog(s) have mRNA binding, unfolded protein binding activity, role in cellular response to unfolded protein, mRNA export from nucleus in response to heat stress, protein folding and cytosol, nucleus localization	Molecular chaperone (small heat-shock protein Hsp26/Hsp42) [O]
**AN10514.3**	**ANIA_10514**	**0.25**		Has domain(s) with predicted nucleotide binding, oxidoreductase activity and role in metabolic process	Dehydrogenases with different specificities (related to short-chain alcohol dehydrogenases) [Q]
**AN10519.3**	**ANIA_10519**	**0.50**		Ortholog(s) have cytosol, nucleus localization	26S proteasome regulatory complex, subunit RPN6/PSMD11 [O]
**AN10539.3**	**ANIA_10539**	**0.25**		Ortholog(s) have cytoplasm localization	Putative N2,N2-dimethylguanosine tRNA methyltransferase [A]
**AN10551.3**	**ANIA_10551**	**0.39**		Ortholog(s) have role in regulation of transcription from RNA polymerase II promoter, tRNA wobble uridine modification and Elongator holoenzyme complex, cytosol, nucleus localization	IkappaB kinase complex, IKAP component [K]
**AN10552.3**	**ANIA_10552**	**0.33**		Ortholog of A. fumigatus Af293 : Afu4g07060, A. niger CBS 513.88 : An04g01460, A. oryzae RIB40 : AO090023000870, Aspergillus wentii : Aspwe1_0167183 and Aspergillus sydowii : Aspsy1_0094979	Acyl-CoA thioesterase [I]
**AN10559.3**	**ANIA_10559**	**0.41**		Ortholog(s) have endoplasmic reticulum localization	Steroid reductase [I]
**AN10562.3**	**ANIA_10562**	**0.44**	msrA	Putative methionine sulfoxide reductase with a predicted role in the reduction of methionine sulfoxide to methionine	Peptide methionine sulfoxide reductase [O]
**AN10590.3**	**ANIA_10590**	**0.35**		Ortholog of A. fumigatus Af293 : Afu3g06380, A. niger CBS 513.88 : An11g09960, A. oryzae RIB40 : AO090020000113, Aspergillus sydowii : Aspsy1_0087862 and Aspergillus terreus NIH2624 : ATET_08788	DNA-binding protein C1D involved in regulation of double-strand break repair [L]
**AN10599.3**	**ANIA_10599**	**0.47**		Has domain(s) with predicted catalytic activity and role in cellular amino acid catabolic process	
**AN10609.3**	**ANIA_10609**	**0.44**		Ortholog(s) have role in mitochondrial respiratory chain complex III assembly and integral to mitochondrial inner membrane localization	Inhibitor of type V adenylyl cyclases/Neuronal presynaptic protein Highwire/PAM/RPM-1 [T]
**AN10611.3**	**ANIA_10611**	**0.27**		Ortholog(s) have role in GPI anchor biosynthetic process, mitotic cytokinesis and endoplasmic reticulum localization	N-acetylglucosaminyltransferase complex, subunit PIG-Q/GPI1, required for phosphatidylinositol biosynthesis [M, O]
**AN10619.3**	**ANIA_10619**	**0.31**		Has domain(s) with predicted carboxy-lyase activity, pyridoxal phosphate binding activity and role in carboxylic acid metabolic process	Glutamate decarboxylase and related proteins [E]
**AN10622.3**	**ANIA_10622**	**0.43**		Ortholog(s) have role in mitochondrial respiratory chain complex assembly and cytosol, mitochondrial intermembrane space, nucleus localization	Uncharacterized conserved protein [S]
**AN10629.3**	**ANIA_10629**	**0.30**		Has domain(s) with predicted role in lipid metabolic process	
**AN10630.3**	**ANIA_10630**	**0.08**		Has domain(s) with predicted integral to membrane localization	Predicted membrane proteins, contain hemolysin III domain [R, T]
**AN10631.3**	**ANIA_10631**	**0.25**		Ortholog(s) have cytosol, nucleus localization	
**AN10634.3**	**ANIA_10634**	**0.44**		Putative ortholog of S. cerevisiae Yme2p which has role in mitochondrial genome maintenance; expression upregulated after exposure to farnesol	
**AN10637.3**	**ANIA_10637**	**0.41**		Ortholog(s) have role in microautophagy, polyphosphate metabolic process, protein localization, vacuolar transport, vacuole fusion, non-autophagic	Protein involved in vacuolar polyphosphate accumulation, contains SPX domain [P]
**AN10649.3**	**ANIA_10649**	**0.47**		Has domain(s) with predicted RNA binding, RNA-directed DNA polymerase activity, role in DNA integration, RNA-dependent DNA replication and nucleus localization	
**AN10654.3**	**ANIA_10654**	**0.47**		Protein of unknown function	
**AN10670.3**	**ANIA_10670**	**0.33**		Has domain(s) with predicted DNA binding, sequence-specific DNA binding RNA polymerase II transcription factor activity, zinc ion binding activity and role in regulation of transcription, DNA-dependent, transcription, DNA-templated	
**AN10695.3**	**ANIA_10695**	**0.35**		Ortholog of A. niger CBS 513.88 : An01g07100	Glutathione S-transferase [O]
**AN10704.3**	**ANIA_10704**	**0.37**	CYP59C1	Putative cytochrome P450	Cytochrome P450 CYP4/CYP19/CYP26 subfamilies [Q, I]
**AN10707.3**	**ANIA_10707**	**0.37**		Ortholog(s) have nucleus localization	DEAD box-containing helicase-like K factor/DNA repair protein [L]
**AN10718.3**	**ANIA_10718**	**0.45**	riboG	6,7-mimethyl-8-ribityl-lumazine synthase; intracellular; protein abundance decreased by menadione stress	6,7-dimethyl-8-ribityllumazine synthase [H]
**AN10742.3**	**ANIA_10742**	**0.31**		Protein of unknown function	
**AN10784.3**	**ANIA_10784**	**0.41**		Ortholog of A. fumigatus Af293 : Afu2g12010, A. niger CBS 513.88 : An02g04070, A. oryzae RIB40 : AO090026000446, Aspergillus wentii : Aspwe1_0053968 and Aspergillus sydowii : Aspsy1_0150883	
**AN10791.3**	**ANIA_10791**	**0.49**	lsb6	Putative 1-phosphatidylinositol 4-kinase; mutants have a strong growth defect	Phosphatidylinositol 4-kinase [T]
**AN10803.3**	**ANIA_10803**	**0.33**		Ortholog of Aspergillus wentii : Aspwe1_0175695, Aspergillus versicolor : Aspve1_0130960, Aspergillus zonatus : Aspzo1_0015856 and Aspergillus sydowii : Aspsy1_0150723	
**AN10806.3**	**ANIA_10806**	**0.25**		Ortholog(s) have cytoplasm localization	Uncharacterized conserved protein [S]
**AN10811.3**	**ANIA_10811**	**0.38**	CYP65U1	Putative cytochrome P450	Cytochrome P450 CYP2 subfamily [Q]
**AN10813.3**	**ANIA_10813**	**0.50**		Ortholog(s) have role in ergot alkaloid biosynthetic process	
**AN10820.3**	**ANIA_10820**	**0.38**		Has domain(s) with predicted carbohydrate binding, catalytic activity and role in carbohydrate catabolic process	
**AN10824.3**	**ANIA_10824**	**0.47**		Ortholog(s) have cytosol localization	Fructose-6-phosphate 2-kinase/fructose-2,6-biphosphatase [G]
**AN10868.3**	**ANIA_10868**	**0.46**		Ortholog of A. niger CBS 513.88 : An14g01330, An02g11890, An14g01620 and A. oryzae RIB40 : AO090012000045, AO090011000315, AO090701000905	
**AN10874.3**	**ANIA_10874**	**0.44**		Ortholog(s) have ubiquitin-protein ligase activity, role in ER-associated ubiquitin-dependent protein catabolic process and endoplasmic reticulum membrane localization	Ubiquitin-protein ligase [O]
**AN10883.3**	**ANIA_10883**	**0.42**		Ortholog of A. nidulans FGSC A4 : AN9032, A. fumigatus Af293 : Afu7g06950, Afu8g02470, A. niger CBS 513.88 : An03g06330, An03g00350, An05g02580 and A. oryzae RIB40 : AO090010000660	
**AN10891.3**	**ANIA_10891**	**0.16**		Ortholog(s) have role in D-xylose transport, glucose import	Predicted transporter (major facilitator superfamily) [R]
**AN10896.3**	**ANIA_10896**	**0.38**		Has domain(s) with predicted nucleotide binding activity	
**AN10897.3**	**ANIA_10897**	**0.36**		Has domain(s) with predicted sequence-specific DNA binding RNA polymerase II transcription factor activity, zinc ion binding activity, role in regulation of transcription, DNA-dependent and nucleus localization	
**AN10898.3**	**ANIA_10898**	**0.29**		Protein of unknown function	
**AN10914.3**	**ANIA_10914**	**0.26**		Has domain(s) with predicted glutamate-1-semialdehyde 2,1-aminomutase activity, pyridoxal phosphate binding, transaminase activity and role in tetrapyrrole biosynthetic process	
**AN10919.3**	**ANIA_10919**	**0.25**		Putative beta-1,4-xylosidase	
**AN10921.3**	**ANIA_10921**	**0.48**		Has domain(s) with predicted hydrolase activity, acting on ester bonds activity, role in GPI anchor metabolic process, intracellular protein transport and intrinsic to endoplasmic reticulum membrane localization	Uncharacterized conserved protein [S]
**AN10928.3**	**ANIA_10928**	**0.50**		Protein of unknown function	
**AN10961.3**	**ANIA_10961**	**0.43**		Has domain(s) with predicted DNA binding, sequence-specific DNA binding RNA polymerase II transcription factor activity, zinc ion binding activity and role in regulation of transcription, DNA-dependent, transcription, DNA-templated	
**AN10967.3**	**ANIA_10967**	**0.36**		Ortholog of Aspergillus versicolor : Aspve1_0096649	Predicted Yippee-type zinc-binding protein [R]
**AN10982.3**	**ANIA_10982**	**0.28**	enaC	Putative P-type ATPase sodium pump	Ca2+ transporting ATPase [P]

**AN10991.3**	**ANIA_10991**	**0.28**	Ortholog(s) have 4-amino-4-deoxychorismate lyase activity, role in folic acid biosynthetic process and cytosol, nucleus localization	
**AN11001.3**	**ANIA_11001**	**0.32**	Ortholog(s) have cytosol, nucleus localization	Uncharacterized conserved protein [S]
**AN11005.3**	**ANIA_11005**	**0.48**	Ortholog(s) have cytosol, nucleus localization	Xaa-Pro aminopeptidase [E]
**AN11016.3**	**ANIA_11016**	**0.42**	Has domain(s) with predicted role in transmembrane transport and integral to membrane localization	
**AN11069.3**	**ANIA_11069**	**0.47**	Ortholog of A. fumigatus Af293 : Afu5g03860, A. niger CBS 513.88 : An09g06330, Neosartorya fischeri NRRL 181 : NFIA_038250 and Aspergillus wentii : Aspwe1_0115239	
**AN11087.3**	**ANIA_11087**	**0.50**	Has domain(s) with predicted role in transmembrane transport and integral to membrane localization	Predicted transporter (major facilitator superfamily) [R]
**AN11105.3**	**ANIA_11105**	**0.31**	Has domain(s) with predicted nucleotide binding, oxidoreductase activity and role in metabolic process	Dehydrogenases with different specificities (related to short-chain alcohol dehydrogenases) [Q]
**AN11111.3**	**ANIA_11111**	**0.46**	Ortholog(s) have 3-phytase activity, acid phosphatase activity, protein tyrosine phosphatase activity and role in cellular response to vitamin B1 starvation, dephosphorylation, thiamine biosynthetic process	Multiple inositol polyphosphate phosphatase [R]
**AN11122.3**	**ANIA_11122**	**0.20**	Has domain(s) with predicted nucleotide binding, oxidoreductase activity and role in metabolic process	Dehydrogenases with different specificities (related to short-chain alcohol dehydrogenases) [Q]
**AN11131.3**	**ANIA_11131**	**0.45**	Ortholog(s) have RNA polymerase II core binding activity, role in regulation of transcription by chromatin organization, transcription elongation from RNA polymerase II promoter and transcription elongation factor complex localization	Uncharacterized Zn ribbon-containing protein [S]
**AN11138.3**	**ANIA_11138**	**0.43**	Ortholog(s) have cytoplasm localization	
**AN11144.3**	**ANIA_11144**	**0.33**	Has domain(s) with predicted transferase activity, transferring glycosyl groups activity and membrane localization	Galactosyltransferases [G]
**AN11161.3**	**ANIA_11161**	**0.41**	Has domain(s) with predicted phosphatidylserine decarboxylase activity and role in phospholipid biosynthetic process	Phosphatidylserine decarboxylase [I]
**AN11162.3**	**ANIA_11162**	**0.44**	Ortholog(s) have nucleolus localization	WD40 repeat protein [R]
**AN11169.3**	**ANIA_11169**	**0.25**	Has domain(s) with predicted DNA binding, sequence-specific DNA binding RNA polymerase II transcription factor activity, zinc ion binding activity and role in regulation of transcription, DNA-dependent, transcription, DNA-templated	
**AN11182.3**	**ANIA_11182**	**0.27**	Ortholog of Aspergillus sydowii : Aspsy1_0061393	
**AN11184.3**	**ANIA_11184**	**0.48**	Has domain(s) with predicted DNA binding, sequence-specific DNA binding RNA polymerase II transcription factor activity, zinc ion binding activity and role in regulation of transcription, DNA-dependent, transcription, DNA-templated	
**AN11197.3**	**ANIA_11197**	**0.35**	Has domain(s) with predicted DNA binding, zinc ion binding activity, role in transcription, DNA-templated and nucleus localization	
**AN11202.3**	**ANIA_11202**	**0.37**	Putative dimethyl-allyl-tryptophan synthase (DMATS) type aromatic prenyltransferase; coregulated member of a DMATS type aromatic prenyltransferase- and NRPS-containing gene cluster	
**AN11207.3**	**ANIA_11207**	**0.05**	Has domain(s) with predicted nucleotide binding, oxidoreductase activity and role in metabolic process	Reductases with broad range of substrate specificities [R]
**AN11212.3**	**ANIA_11212**	**0.14**	Has domain(s) with predicted DNA binding, zinc ion binding activity, role in transcription, DNA-templated and nucleus localization	
**AN11244.3**	**ANIA_11244**	**0.36**	Ortholog(s) have fungal-type vacuole membrane localization	Tumor differentially expressed (TDE) protein [S]
**AN11287.3**	**ANIA_11287**	**0.40**	Protein of unknown function	
**AN11317.3**	**ANIA_11317**	**0.33**	Ortholog of A. niger CBS 513.88 : An04g07180, Neosartorya fischeri NRRL 181 : NFIA_106180, Aspergillus wentii : Aspwe1_0034219 and Aspergillus niger ATCC 1015 : 204621-mRNA	
**AN11337.3**	**ANIA_11337**	**0.20**	Ortholog of A. niger CBS 513.88 : An18g01250, Aspergillus tubingensis : Asptu1_0114551, Aspergillus brasiliensis : Aspbr1_0322983 and Aspergillus kawachii : Aspka1_0173401	
**AN11364.3**	**ANIA_11364**	**0.18**	Protein of unknown function	
**AN11378.3**	**ANIA_11378**	**0.09**	Ortholog of A. oryzae RIB40 : AO090001000134, Aspergillus glaucus : Aspgl1_0071332, Aspergillus wentii : Aspwe1_0171393 and Aspergillus versicolor : Aspve1_0087914	
**AN11387.3**	**ANIA_11387**	**0.49**	Protein of unknown function	
**AN11487.3**	**ANIA_11487**	**0.50**	Ortholog(s) have NADH dehydrogenase (ubiquinone) activity and role in mitochondrial respiratory chain complex I assembly, programmed cell death	
**AN11533.3**	**ANIA_11533**	**0.41**	Ortholog of A. fumigatus Af293 : Afu8g06974, A. niger CBS 513.88 : An01g03010, Neosartorya fischeri NRRL 181 : NFIA_099710 and Aspergillus kawachii : Aspka1_0178966	
**AN11574.3**	**ANIA_11574**	**0.13**	Protein of unknown function	
**AN11585.3**	**ANIA_11585**	**0.46**	Protein of unknown function	

Reference

1. AspGD (from The Board of Trustees, Leland Stanford Junior University and the Broad Institute)
: Chromosomal Feature Files - Last Update dates 2014-03-02

2. Transcription factor (DBD): Wilson*et al*., 2008. *Nucleic Acids Res.* 36, D88–D92

3. KOGs
: Tatusov et al., 2003. BMC Bioinformatics 2003, 4:41
: Original PEDANT database name: p3_p130_Asp_nidul (PEDANT 3) - Calculation dates 2007-08-11

4. PEDANT 3: Walter *et al*., 2009. Nucleic Acids Res. 37, Database issue
: Original PEDANT database name: p3_r40559_Asp_nidul (PEDANT 3) - Calculation dates 2013-09-19

Supplementary Table 1.3. Functional categoried of genes differentially regulated by *vosA* in conidia

: The genes were functionally categorized based on KOGs description.

KOGs Category	Description	genes in up-cluster			genes in down-cluster			Random Occurrence Value*3
		%*1	P-value	fold*2	%*1	P-value	fold*2	
**Information storage and processing**								
J	Translation, ribosomal structure and biogenesis	1.256	<0.0001	0.406	3.807	0.5376	1.232	3.090
A	RNA processing and modification	0.739	0.0002	0.346	0.921	0.0016	0.431	2.136
K	Transcription	1.354	<0.0001	0.515	1.485	0.0347	0.565	2.627
L	Replication, recombination and repair	0.949	0.0006	0.540	1.116	0.0183	0.635	1.758
B	Chromatin structure and dynamics	0.443	0.0163	0.551	0.558	0.3210	0.695	0.803
**Cellular processes and signaling**								
D	Cell cycle control, cell division, chromosome partitioning	0.882	0.1716	0.546	0.929	0.7367	0.575	1.616
Y	Nuclear structure	0.071	0.0066	0.277	0.248	0.9597	0.974	0.255
V	Defense mechanisms	0.724	0.0058	1.393	0.323	0.0037	0.621	0.520
T	Signal transduction mechanisms	2.763	0.1432	0.769	4.188	0.1916	1.166	3.591
M	Cell wall/membrane/envelope biogenesis	1.073	0.1545	1.305	0.552	0.0063	0.671	0.822
N	Cell motility	0.000	-	0.000	0.000	-	0.000	0.038
Z	Cytoskeleton	1.790	0.7762	1.121	1.213	0.0483	0.760	1.597
W	Extracellular structures	0.000	-	0.000	0.262	0.1017	3.471	0.076
U	Intracellular trafficking, secretion, and vesicular transport	1.989	0.0003	0.638	1.249	0.0176	0.401	3.119
O	Posttranslational modification, protein turnover, chaperones	3.010	0.0005	0.701	4.992	0.2040	1.163	4.291
**Metabolism**								
C	Energy production and conversion	2.843	0.0376	0.748	5.746	0.0208	1.512	3.799
G	Carbohydrate transport and metabolism	4.367	0.0417	1.313	4.593	0.0067	1.381	3.327
E	Amino acid transport and metabolism	2.911	0.0003	0.865	2.653	0.0756	0.789	3.365
F	Nucleotide transport and metabolism	0.387	0.0785	0.460	0.891	0.7363	1.059	0.841
H	Coenzyme transport and metabolism	0.673	0.0151	0.685	1.439	0.0336	1.464	0.983
I	Lipid transport and metabolism	4.900	0.0014	1.271	3.393	0.0837	0.880	3.856
P	Inorganic ion transport and metabolism	1.415	0.3456	1.077	2.361	0.0017	1.797	1.314
Q	Secondary metabolites biosynthesis, transport and catabolism	6.203	0.0004	1.662	4.409	0.1255	1.181	3.733
**Poorly characterized**								
R	General function prediction only	9.014	0.6020	0.920	11.468	0.0056	1.170	9.801
S	Function unknown	1.889	0.0169	0.651	3.615	0.0016	1.246	2.901

*1. %: relative average portion of regulated genes in the cluster, regulated gene in certain category / total regulated genes × 100

*2. fold: the ratio of [relative portion of genes in the each cluster] to [Random Occurrence Value]

*3. random occurrence rate for each KOG functional category in the whole *A. nidulans* genome

Supplementary Table 1.4. Functional categoried of genes differentially regulated by *vosA* in conidia

: The genes were functionally categorized based on GO description.

GO Category		GO Description	genes in up-cluster			genes in down-cluster			Random Occurrence Value*3
			%*1	P-value	fold*2	%*1	P-value	fold*2	
GO:0005618	Cellular components	**cell wall**	2.603	0.0266	2.040	0.777	0.0097	0.609	1.276
GO:0042546	Biological Process	cell wall biogenesis	2.685	<0.0001	2.429	0.185	0.0009	0.167	1.106
GO:0044036	Biological Process	cell wall macromolecule metabolic process	1.961	0.0002	1.572	0.245	0.0032	0.196	1.248
GO:0034406	Biological Process	cell wall beta-glucan metabolic process	0.443	0.0266	3.345	0.000	-	0.000	0.132
GO:0006075	Biological Process	1,3-beta-D-glucan biosynthetic process	0.354	0.2194	3.743	0.000	-	0.000	0.095
GO:0006037	Biological Process	cell wall chitin metabolic process	0.724	0.0002	3.482	0.000	-	0.000	0.208
GO:0009277	Cellular compoment	**fungal-type cell wall**	1.930	0.0250	2.295	0.457	0.0107	0.544	0.841
GO:0031505	Biological Process	fungal-type cell wall organization	2.582	<0.0001	1.910	0.905	0.0006	0.669	1.351
GO:0009272	Biological Process	fungal-type cell wall biogenesis	1.769	0.0004	2.369	0.094	0.0003	0.126	0.747
GO:0031160	Cellular components	**spore wall**	0.230	0.0169	1.523	0.000	-	0.000	0.151
GO:0070590	Biological Process	spore wall biogenesis	1.111	0.0085	3.093	0.000	-	0.000	0.359
GO:0030446	Cellular compoment	**hyphal cell wall**	0.513	0.1196	1.940	0.323	0.1480	1.220	0.265

*1. %: relative average portion of regulated genes in the cluster, regulated gene in certain category / total regulated genes × 100

*2. fold: the ratio of [relative portion of genes in the each cluster] to [Random Occurrence Value]

*3. random occurrence rate for each GO functional category in the whole *A. nidulans* genome
